# Supplementary material for: Hematological and Serum Biochemical Reference Intervals for Alphaxalone Sedated Common Marmosets (Callithrix jacchus)
Source: Animals (Basel). 2024 Mar 3;14(5):790. doi: 10.3390/ani14050790 (PMC10930924; doi:10.3390/ani14050790)
Supplement: Supplementary file 1 [file animals-14-00790-s001.zip › Figure S1.pdf]

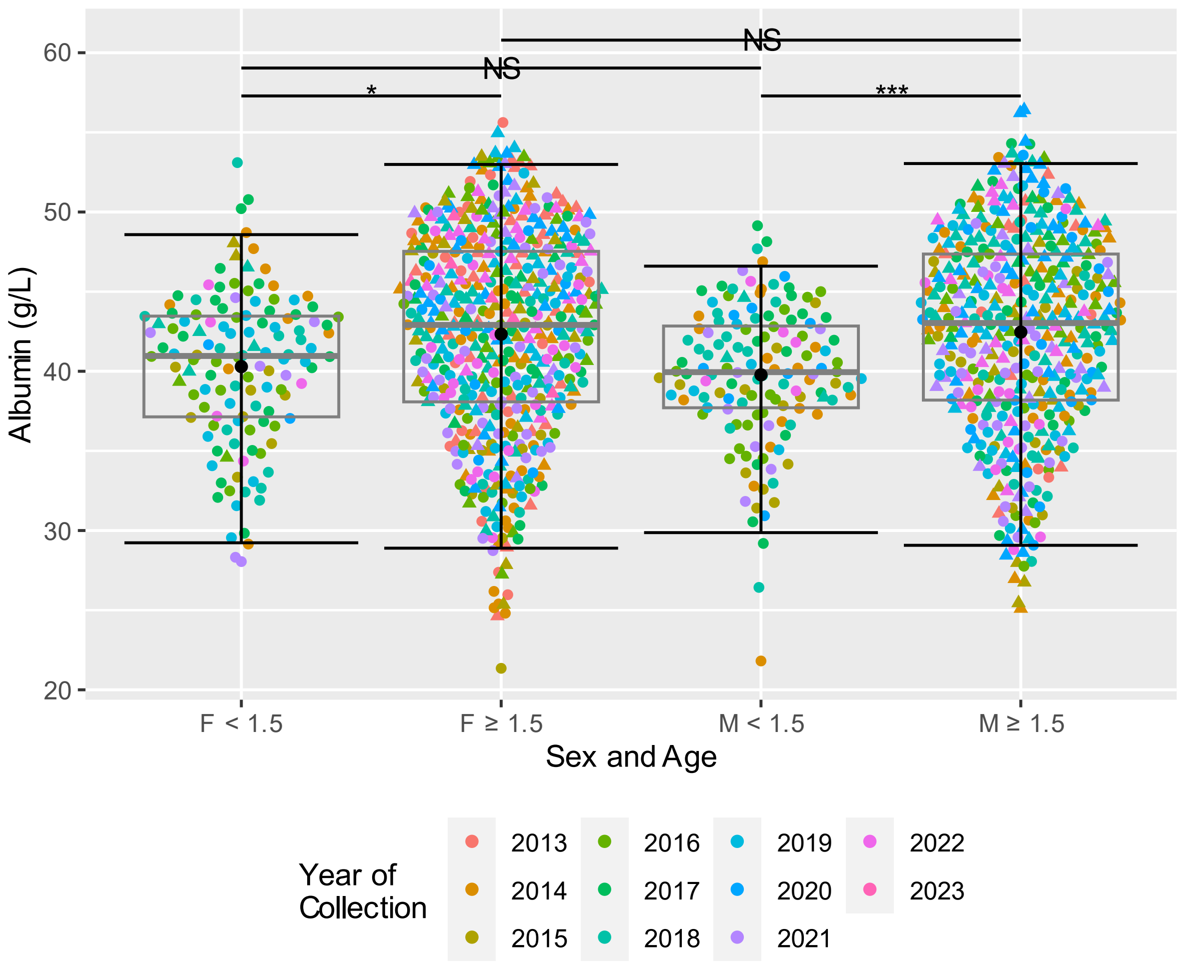

Figure S1 1. Albumin levels according to sex and age in common marmosets. Boxes indicate median and quartile ranges; black point indicates arithmetic mean and error bars indicate the normal range. Symbol colours indicate year of collection, round symbols indicate animals with outdoor access and triangles indicate indoor housed animals; NS = not significant, asterisks indicate statistical significance levels * < 0.001, ** < 0.0001, *** < 0.00001, **** < 0.000001

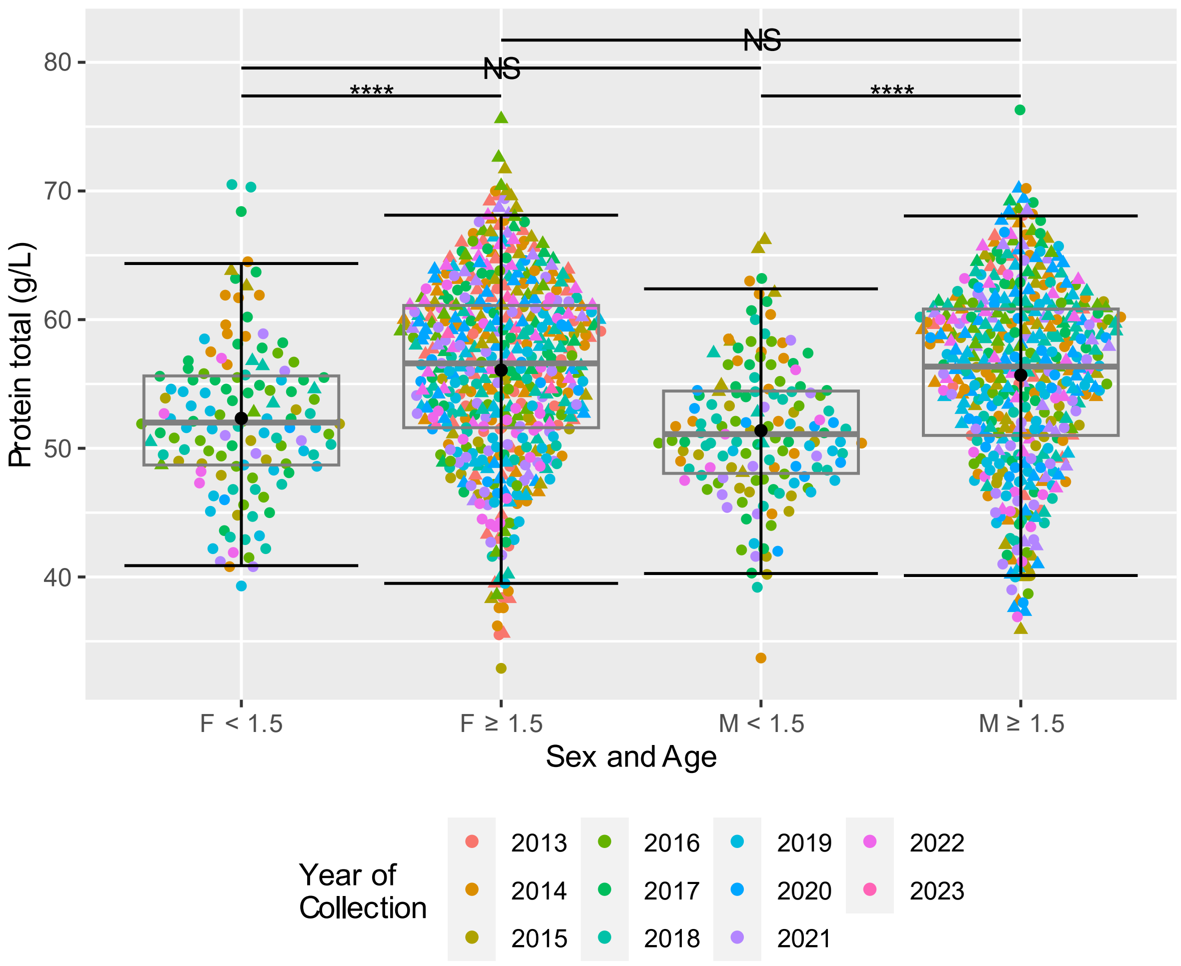

Figure S1 2. Total Protein levels according to sex and age in common marmosets. Boxes indicate median and quartile ranges; black point indicates arithmetic mean and error bars indicate the normal range. Symbol colours indicate year of collection, round symbols indicate animals with outdoor access and triangles indicate indoor housed animals; NS = not significant, asterisks indicate statistical significance levels * < 0.001, ** < 0.0001, *** < 0.00001, **** < 0.000001

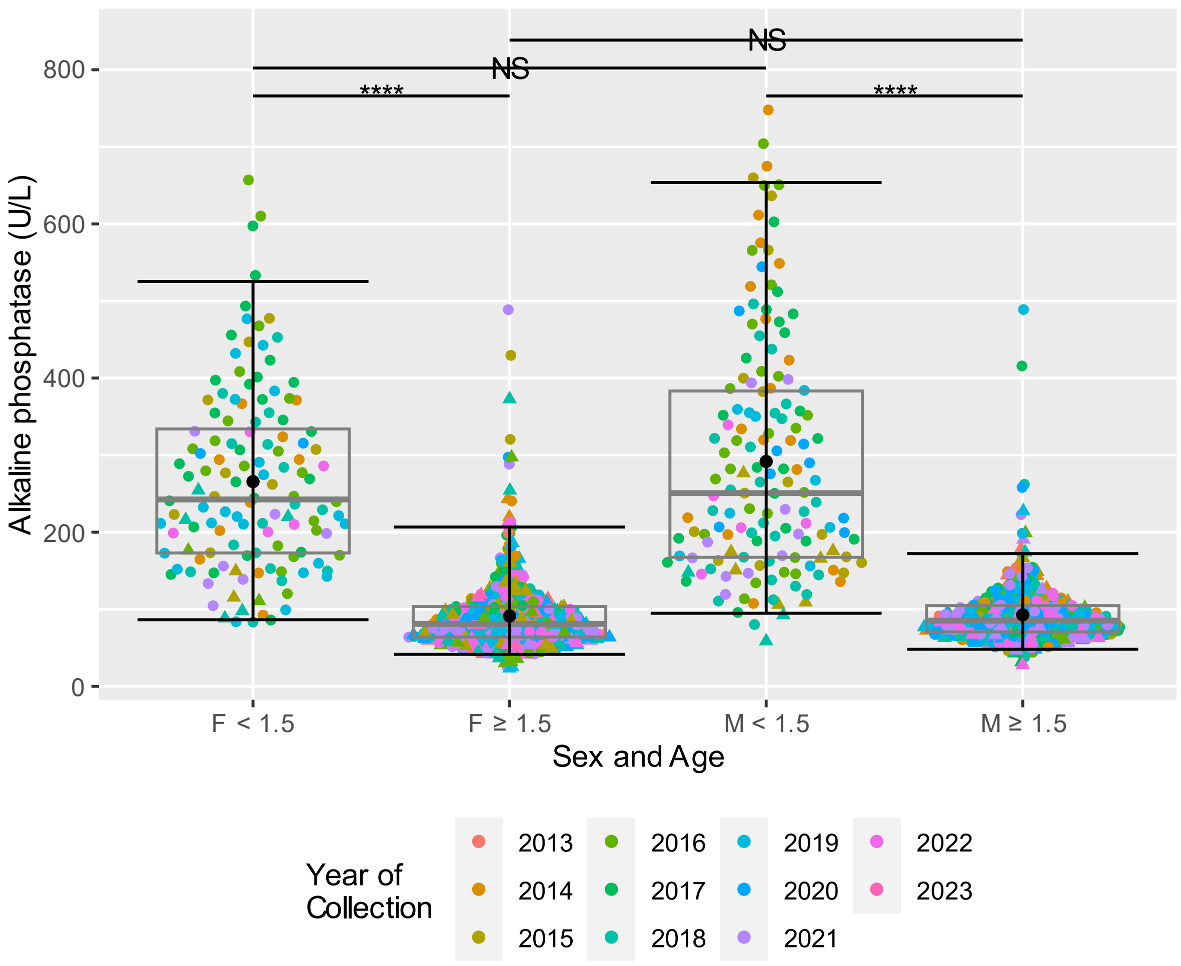

Figure S1 3. Alkaline Phosphatase levels according to sex and age in common marmosets. Boxes indicate median and quartile ranges; black point indicates arithmetic mean and error bars indicate the normal range. Symbol colours indicate year of collection, round symbols indicate animals with outdoor access and triangles indicate indoor housed animals; NS = not significant, asterisks indicate statistical significance levels * < 0.001, ** < 0.0001, *** < 0.00001, **** < 0.000001

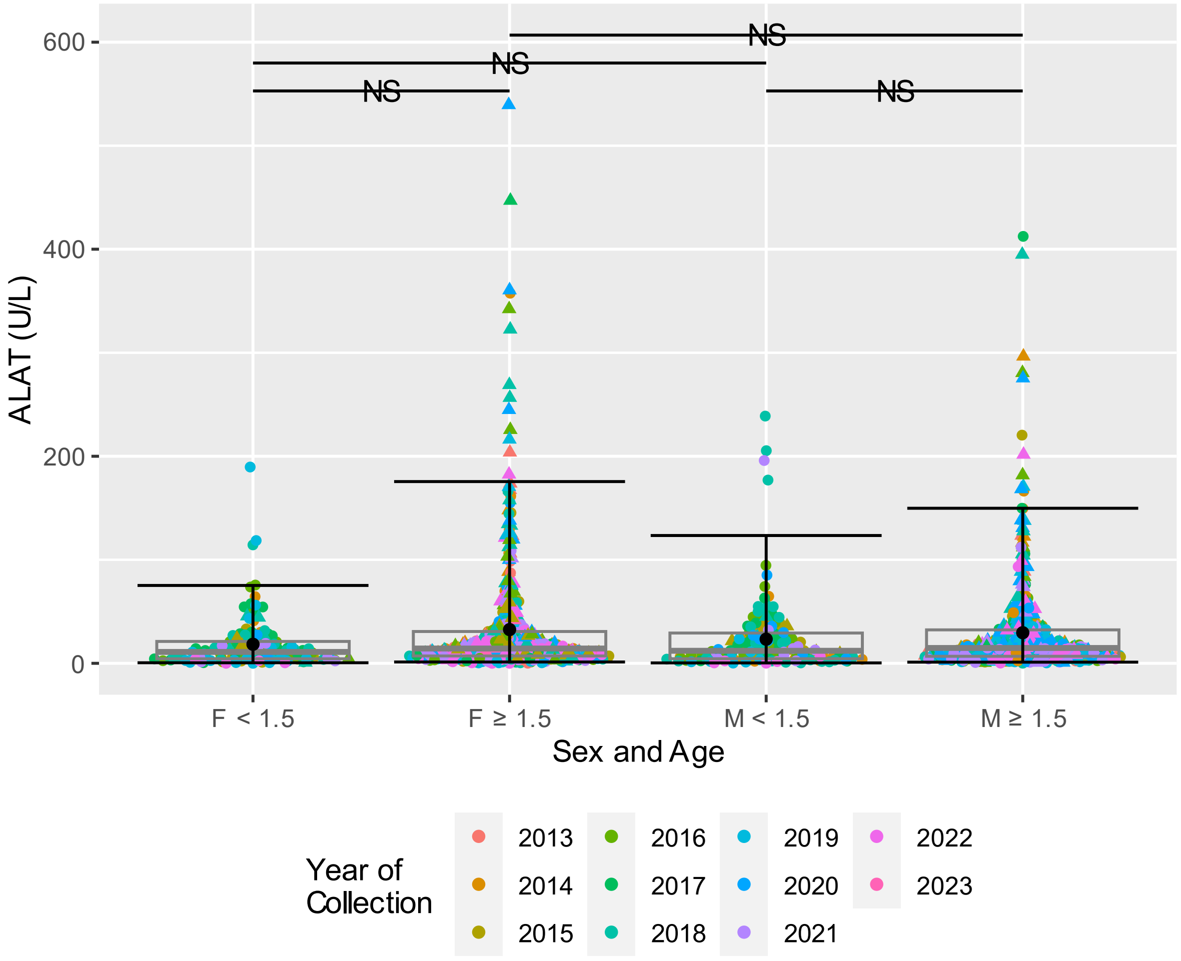

Figure S1 4. ALAT levels according to sex and age in common marmosets. Boxes indicate median and quartile ranges; black point indicates arithmetic mean and error bars indicate the normal range. Symbol colours indicate year of collection, round symbols indicate animals with outdoor access and triangles indicate indoor housed animals; NS = not significant, asterisks indicate statistical significance levels * < 0.001, ** < 0.0001, *** < 0.00001, **** < 0.000001

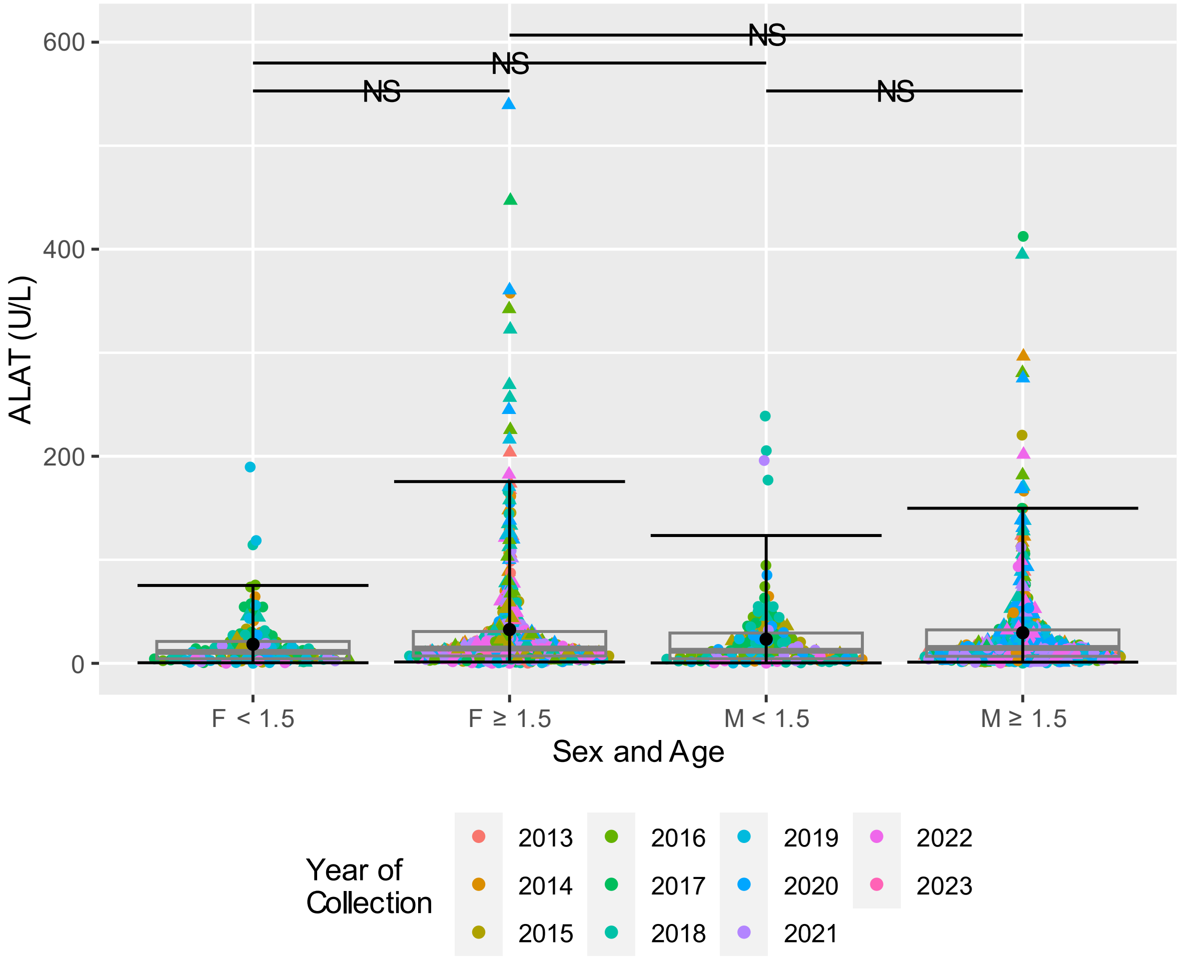

Figure S1 5. ASAT levels according to sex and age in common marmosets. Boxes indicate median and quartile ranges; black point indicates arithmetic mean and error bars indicate the normal range. Symbol colours indicate year of collection, round symbols indicate animals with outdoor access and triangles indicate indoor housed animals; NS = not significant, asterisks indicate statistical significance levels * < 0.001, ** < 0.0001, *** < 0.00001, **** < 0.000001

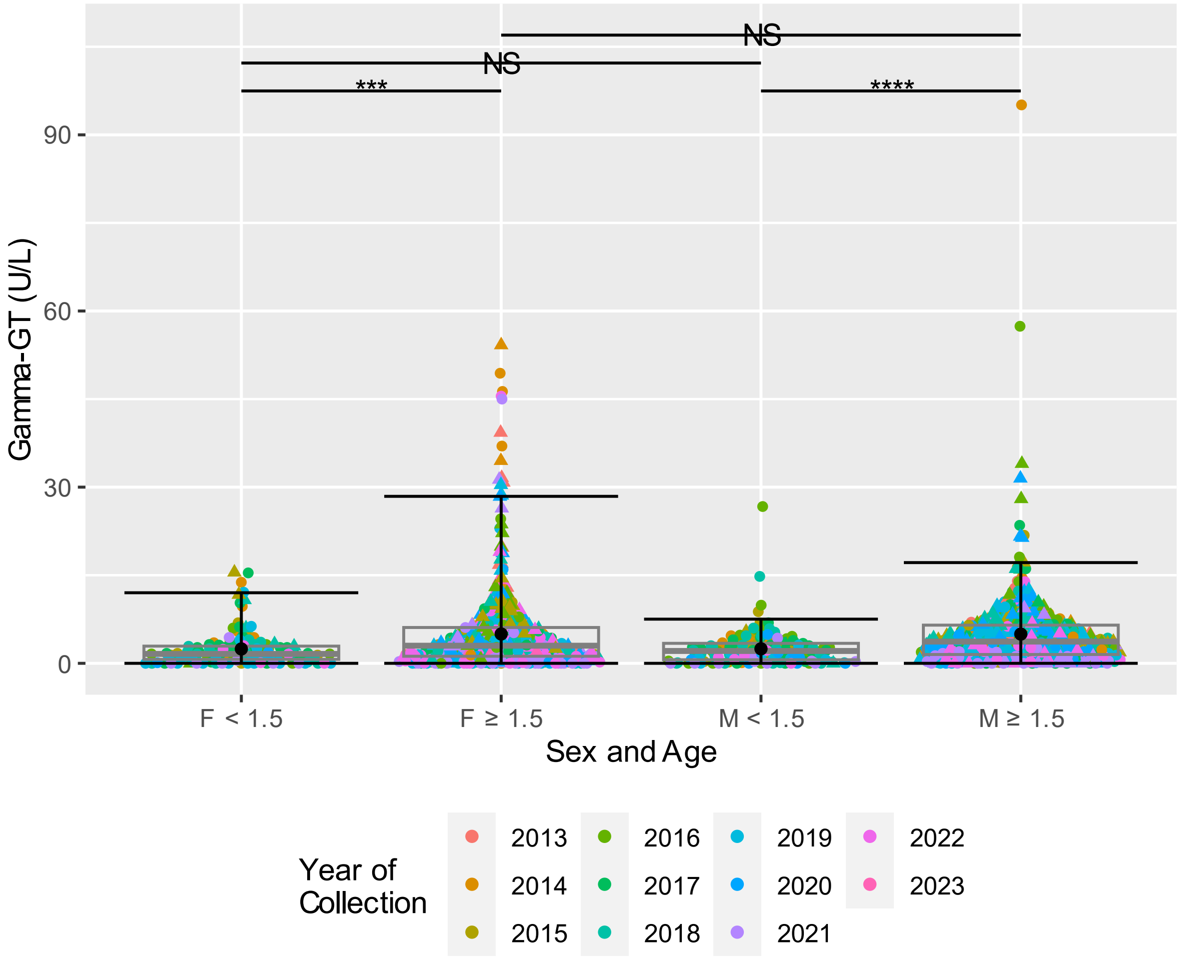

Figure S1 6. Gamma-GT levels according to sex and age in common marmosets. Boxes indicate median and quartile ranges; black point indicates arithmetic mean and error bars indicate the normal range. Symbol colours indicate year of collection, round symbols indicate animals with outdoor access and triangles indicate indoor housed animals; NS = not significant, asterisks indicate statistical significance levels * < 0.001, ** < 0.0001, *** < 0.00001, **** < 0.000001

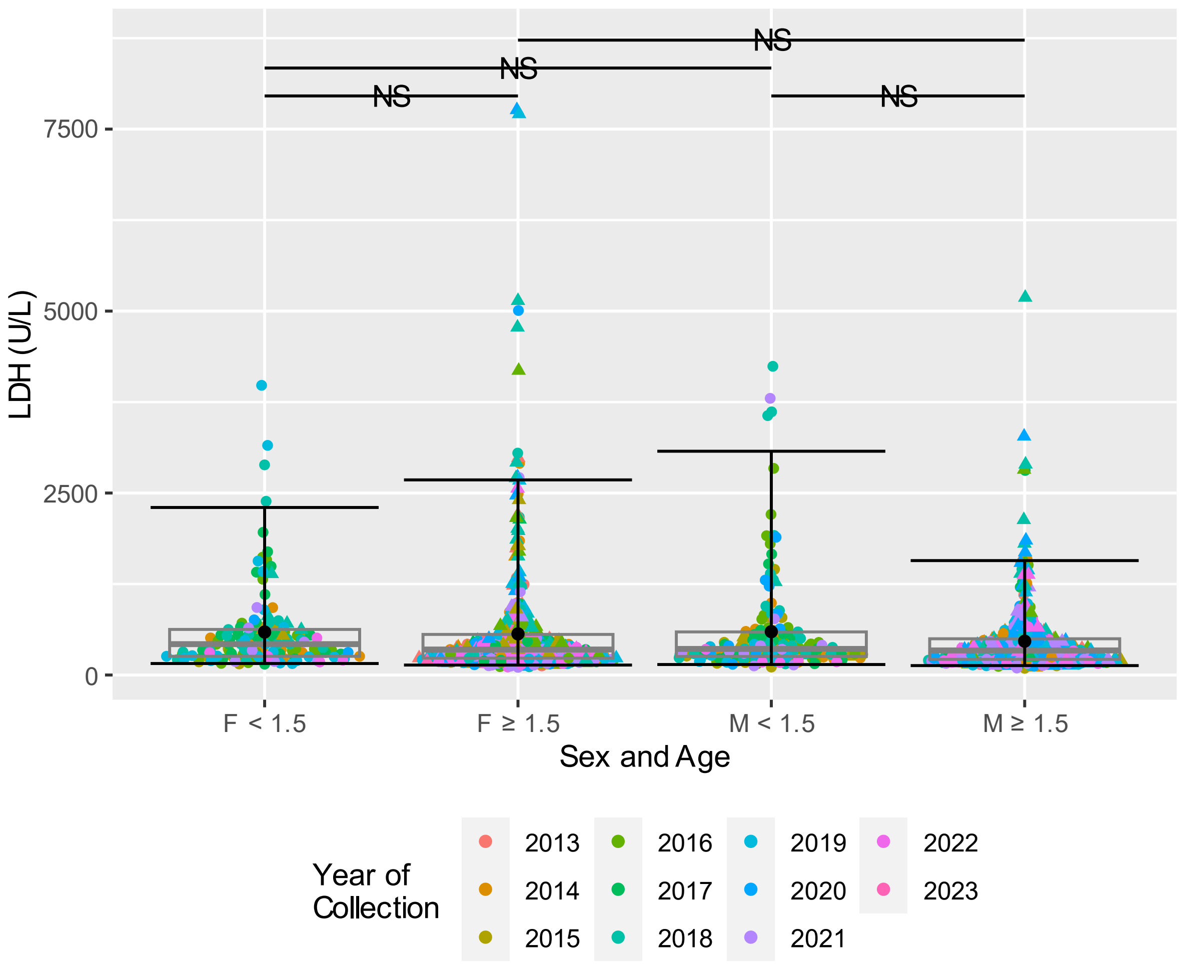

Figure S1 7. LDH levels according to sex and age in common marmosets. Boxes indicate median and quartile ranges; black point indicates arithmetic mean and error bars indicate the normal range. Symbol colours indicate year of collection, round symbols indicate animals with outdoor access and triangles indicate indoor housed animals; NS = not significant, asterisks indicate statistical significance levels * < 0.001, ** < 0.0001, *** < 0.00001, **** < 0.000001

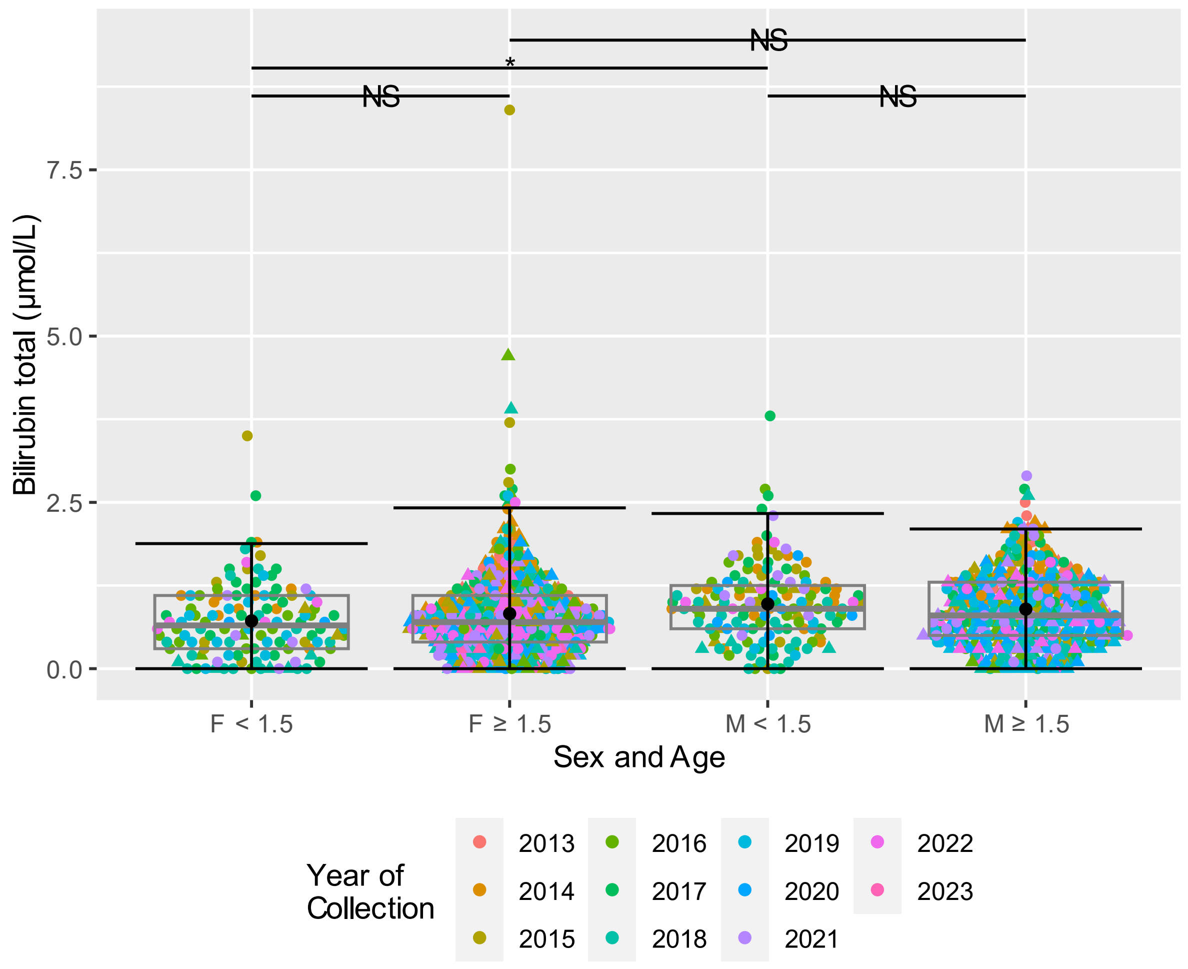

Figure S1 8. Total Bilirubin levels according to sex and age in common marmosets. Boxes indicate median and quartile ranges; black point indicates arithmetic mean and error bars indicate the normal range. Symbol colours indicate year of collection, round symbols indicate animals with outdoor access and triangles indicate indoor housed animals; NS = not significant, asterisks indicate statistical significance levels * < 0.001, ** < 0.0001, *** < 0.00001, **** < 0.000001

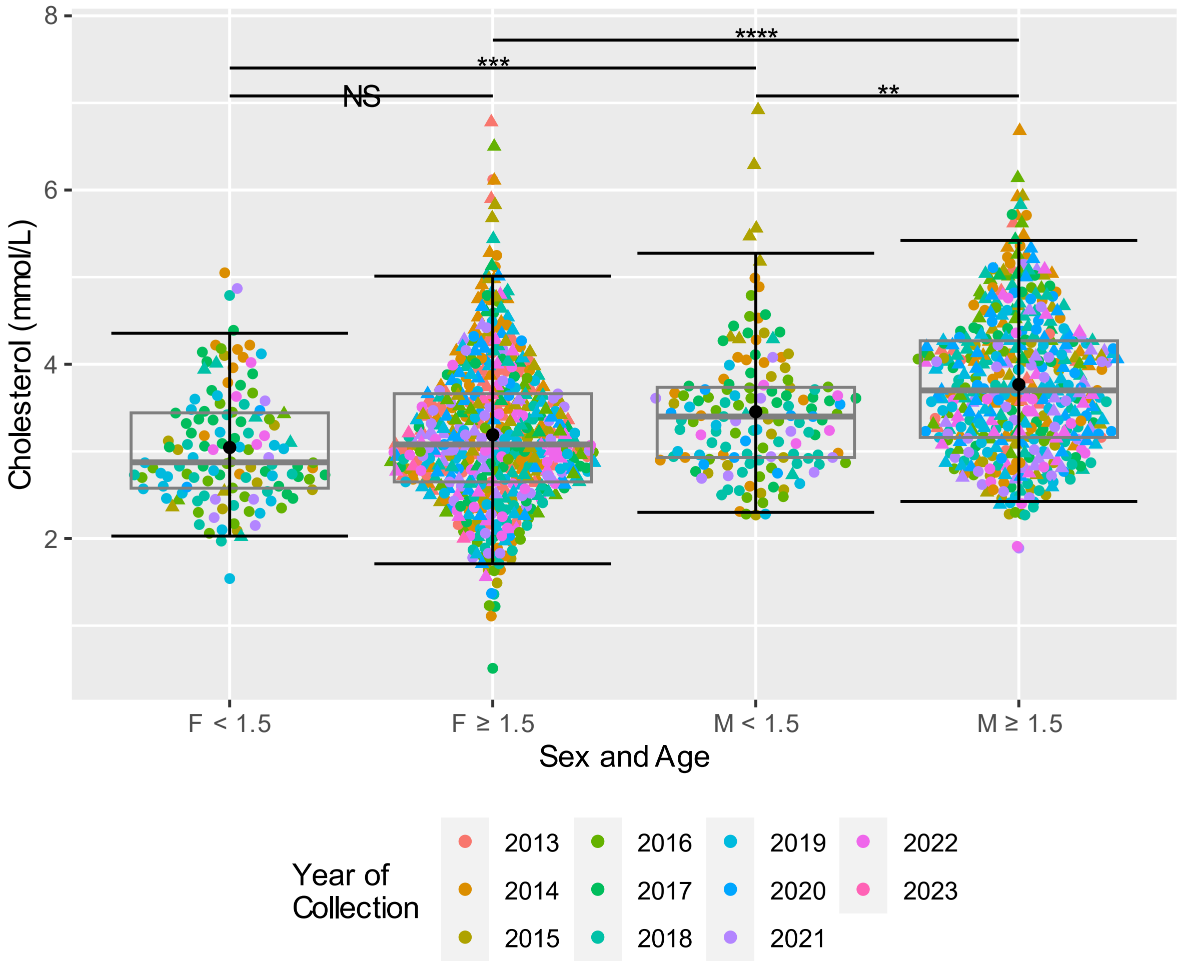

Figure S1 9. Cholesterol levels according to sex and age in common marmosets. Boxes indicate median and quartile ranges; black point indicates arithmetic mean and error bars indicate the normal range. Symbol colours indicate year of collection, round symbols indicate animals with outdoor access and triangles indicate indoor housed animals; NS = not significant, asterisks indicate statistical significance levels * < 0.001, ** < 0.0001, *** < 0.00001, **** < 0.000001

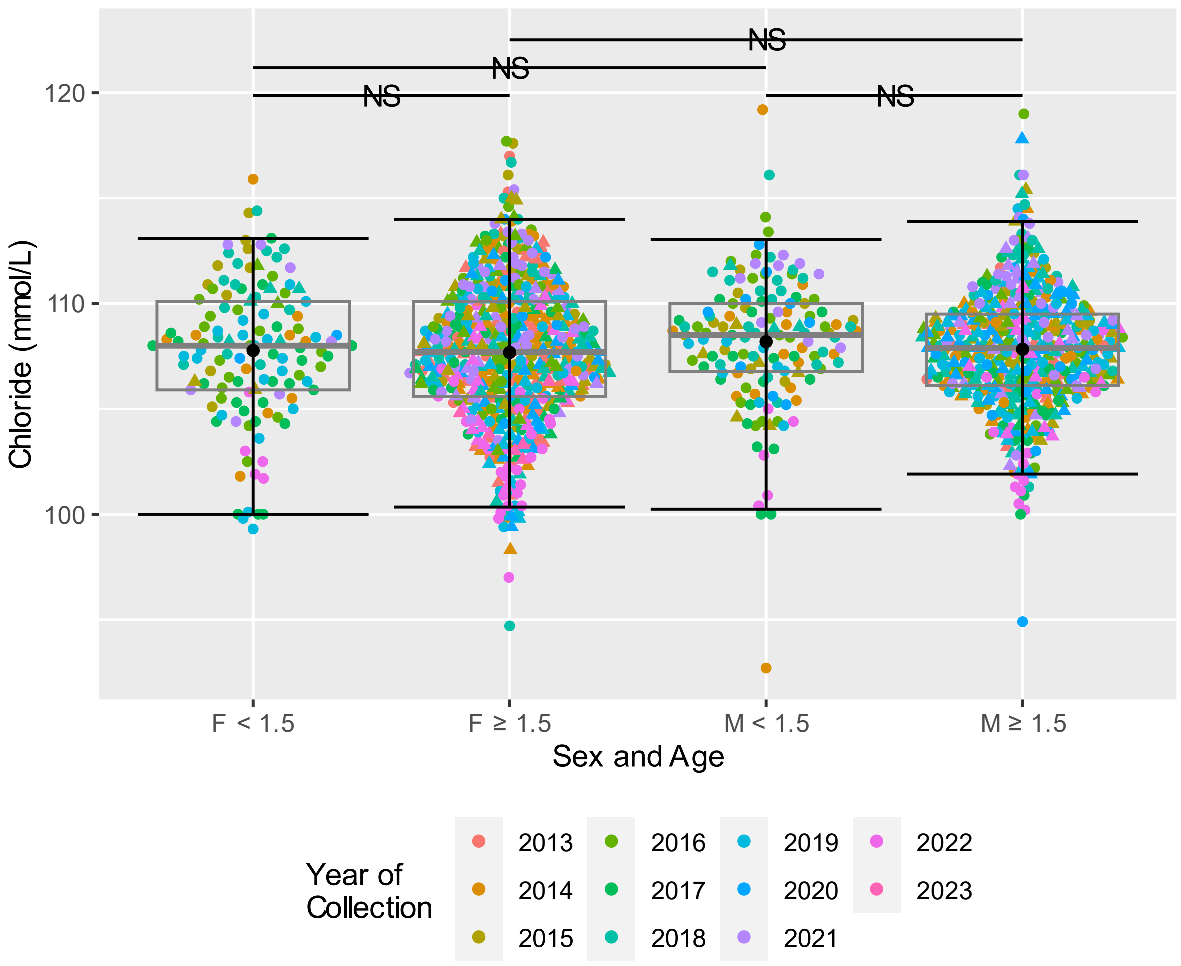

Figure S1 10. Chloride levels according to sex and age in common marmosets. Boxes indicate median and quartile ranges; black point indicates arithmetic mean and error bars indicate the normal range. Symbol colours indicate year of collection, round symbols indicate animals with outdoor access and triangles indicate indoor housed animals; NS = not significant, asterisks indicate statistical significance levels * < 0.001, ** < 0.0001, *** < 0.00001, **** < 0.000001

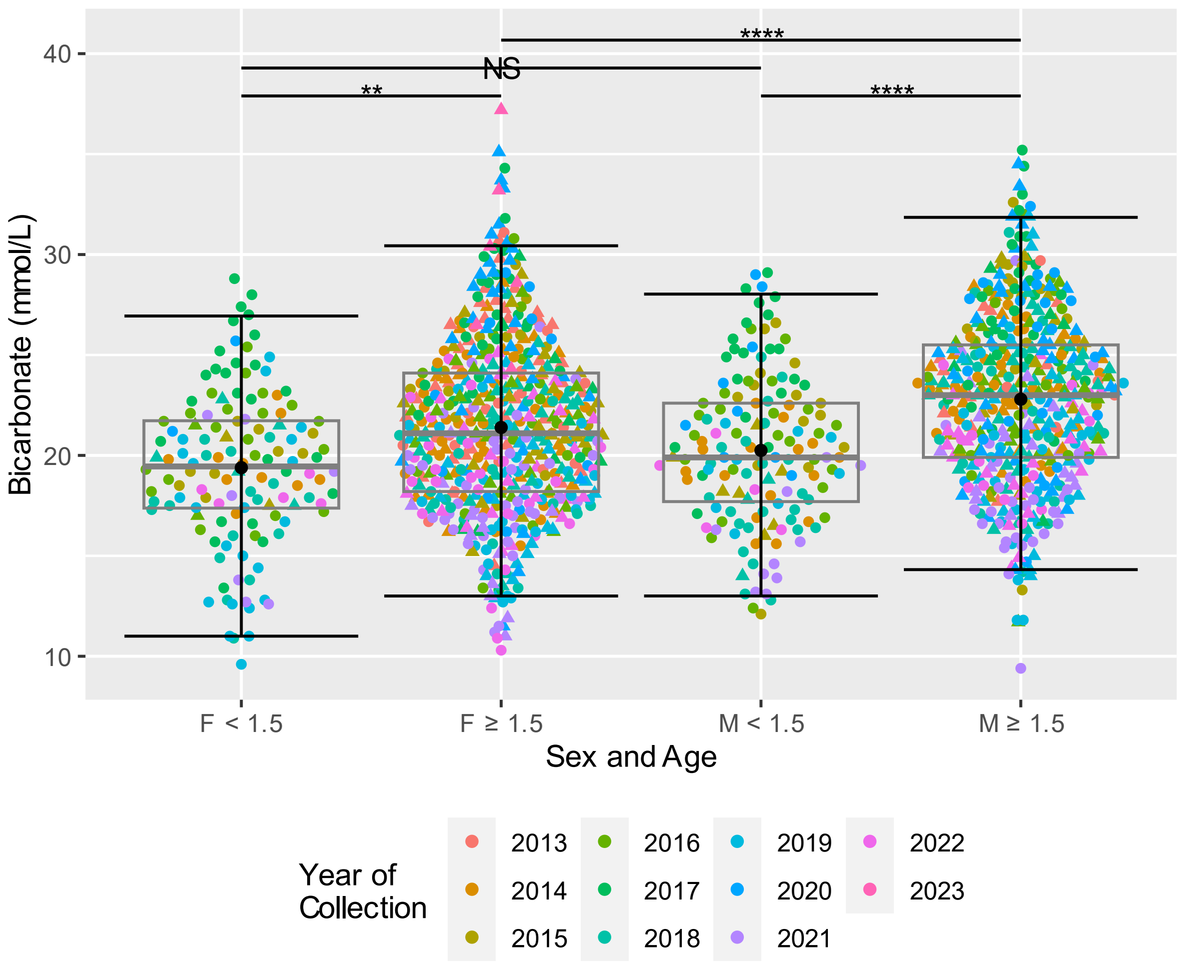

Figure S1 11. Bicarbonate levels according to sex and age in common marmosets. Boxes indicate median and quartile ranges; black point indicates arithmetic mean and error bars indicate the normal range. Symbol colours indicate year of collection, round symbols indicate animals with outdoor access and triangles indicate indoor housed animals; NS = not significant, asterisks indicate statistical significance levels * < 0.001, ** < 0.0001, *** < 0.00001, **** < 0.000001

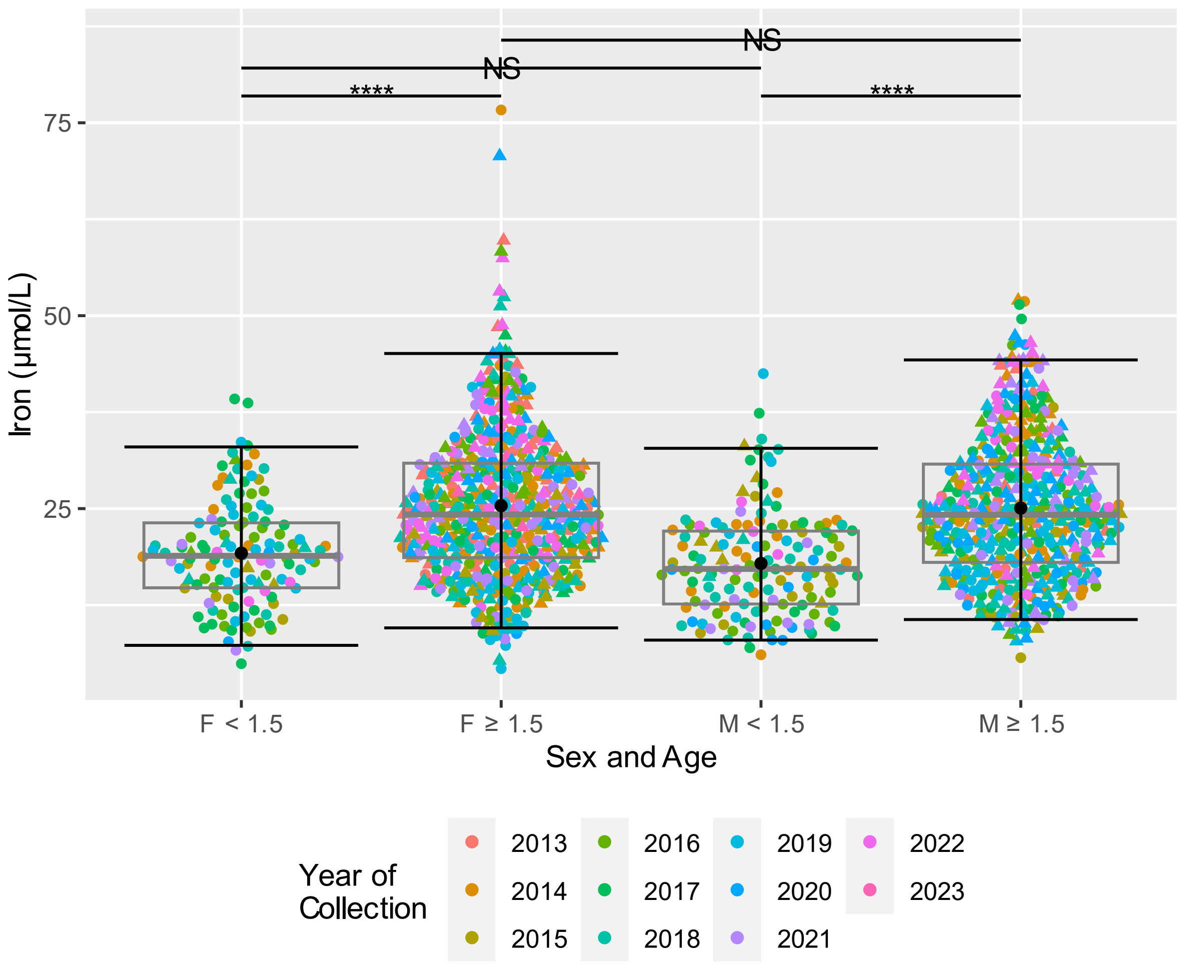

Figure S1 12. Iron levels according to sex and age in common marmosets. Boxes indicate median and quartile ranges; black point indicates arithmetic mean and error bars indicate the normal range. Symbol colours indicate year of collection, round symbols indicate animals with outdoor access and triangles indicate indoor housed animals; NS = not significant, asterisks indicate statistical significance levels * < 0.001, ** < 0.0001, *** < 0.00001, **** < 0.000001

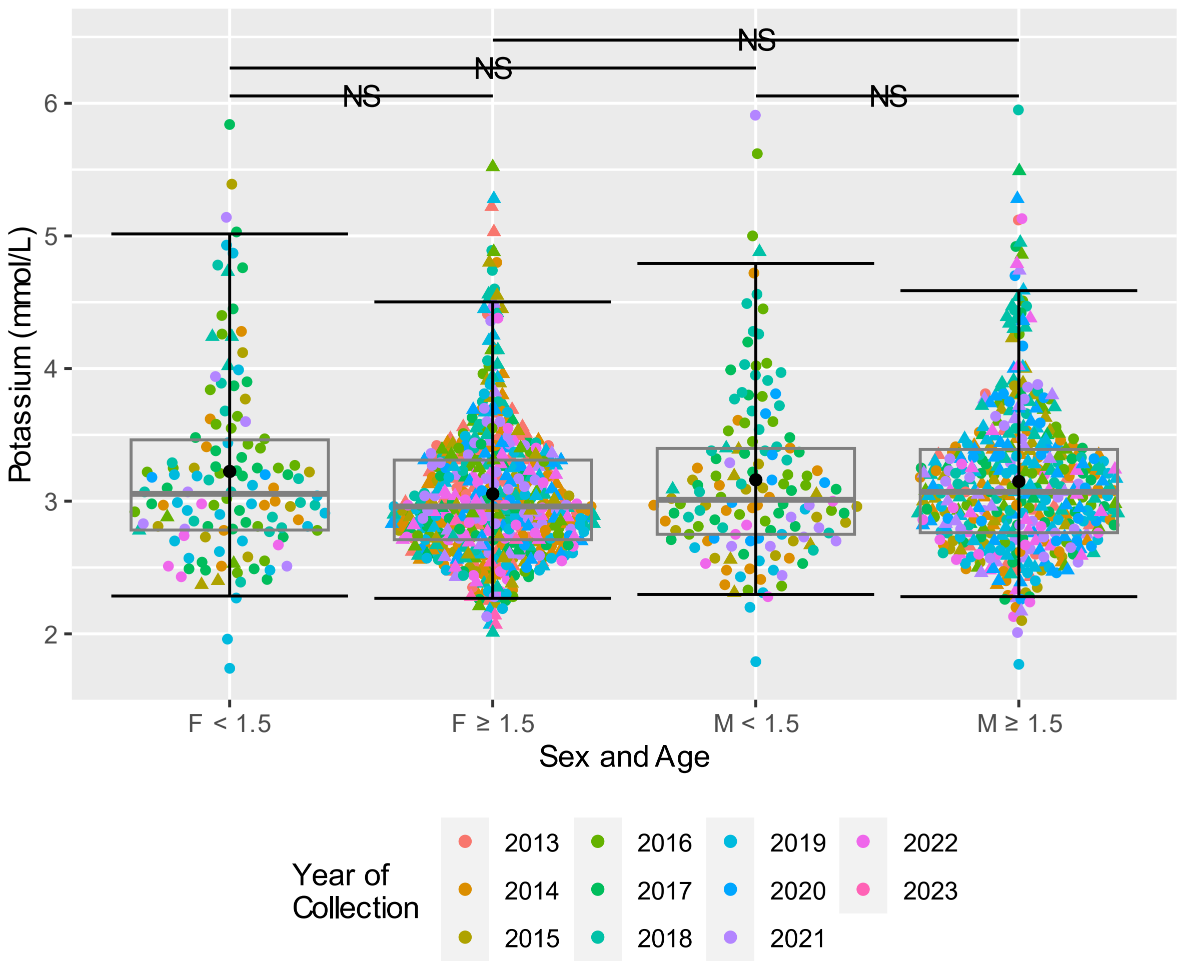

Figure S1 13. Potassium levels according to sex and age in common marmosets. Boxes indicate median and quartile ranges; black point indicates arithmetic mean and error bars indicate the normal range. Symbol colours indicate year of collection, round symbols indicate animals with outdoor access and triangles indicate indoor housed animals; NS = not significant, asterisks indicate statistical significance levels * < 0.001, ** < 0.0001, *** < 0.00001, **** < 0.000001

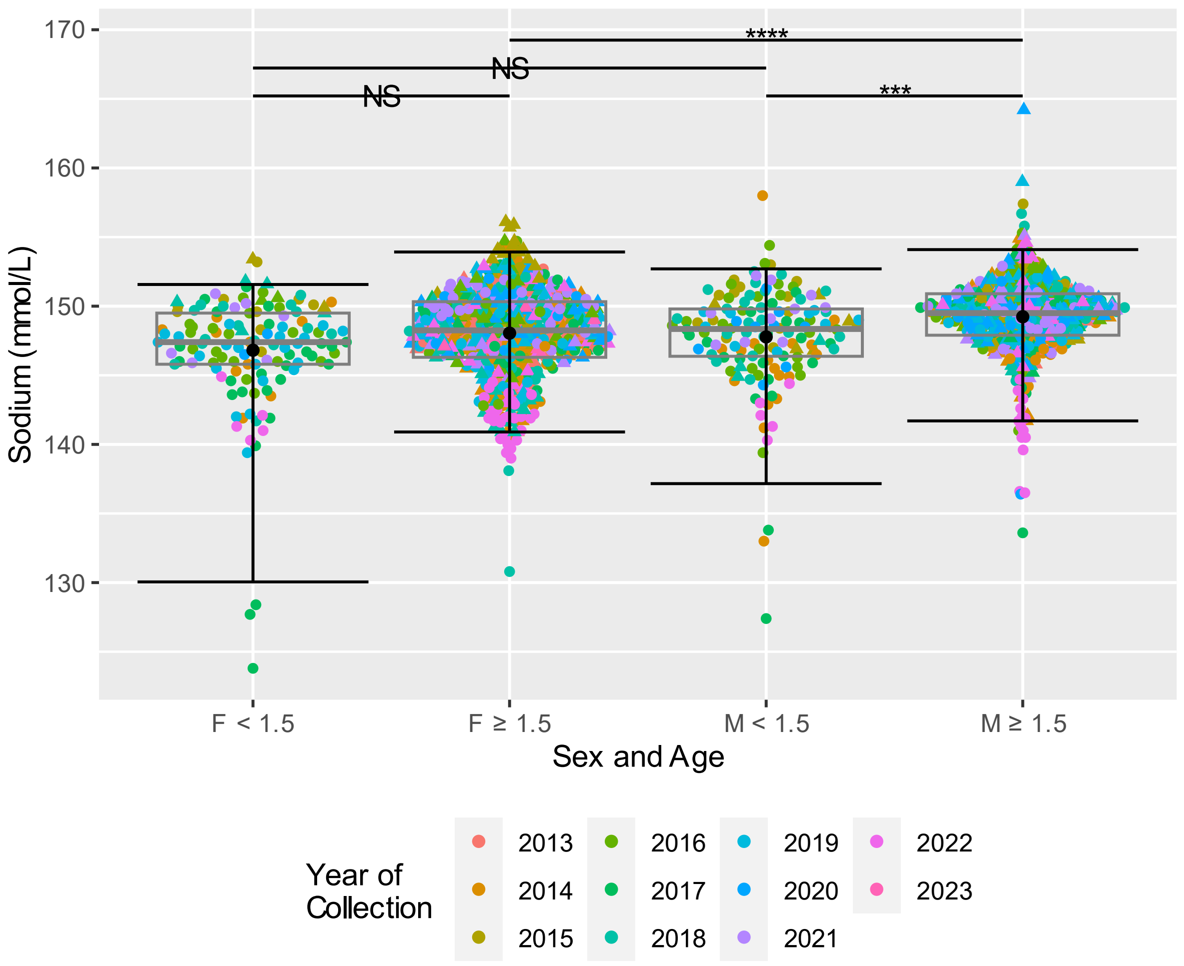

Figure S1 14. Sodium levels according to sex and age in common marmosets. Boxes indicate median and quartile ranges; black point indicates arithmetic mean and error bars indicate the normal range. Symbol colours indicate year of collection, round symbols indicate animals with outdoor access and triangles indicate indoor housed animals; NS = not significant, asterisks indicate statistical significance levels * < 0.001, ** < 0.0001, *** < 0.00001, **** < 0.000001

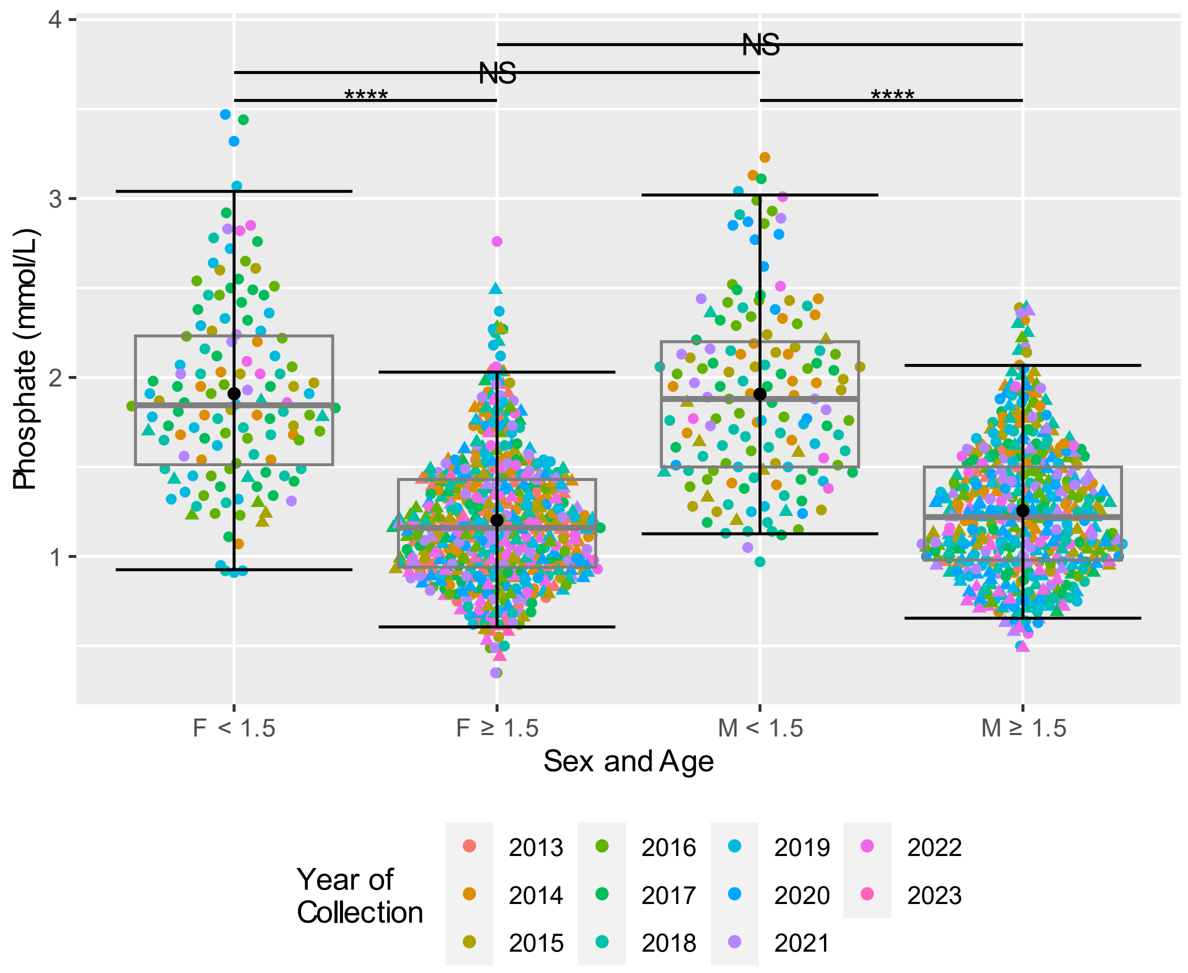

Figure S1 15. Phosphate levels according to sex and age in common marmosets. Boxes indicate median and quartile ranges; black point indicates arithmetic mean and error bars indicate the normal range. Symbol colours indicate year of collection, round symbols indicate animals with outdoor access and triangles indicate indoor housed animals; NS = not significant, asterisks indicate statistical significance levels * < 0.001, ** < 0.0001, *** < 0.00001, **** < 0.000001

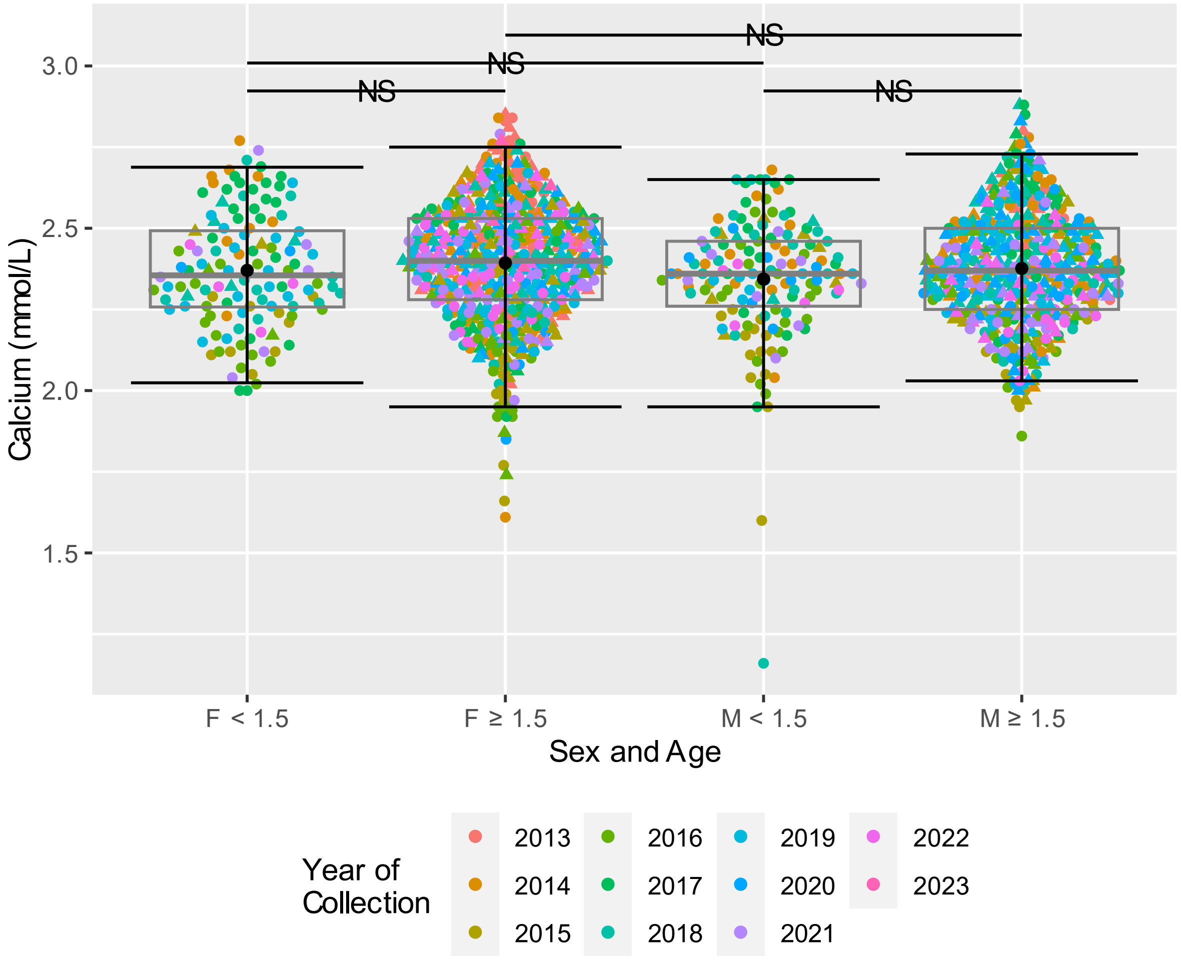

Figure S1 16. Calcium levels according to sex and age in common marmosets. Boxes indicate median and quartile ranges; black point indicates arithmetic mean and error bars indicate the normal range. Symbol colours indicate year of collection, round symbols indicate animals with outdoor access and triangles indicate indoor housed animals; NS = not significant, asterisks indicate statistical significance levels * < 0.001, ** < 0.0001, *** < 0.00001, **** < 0.000001

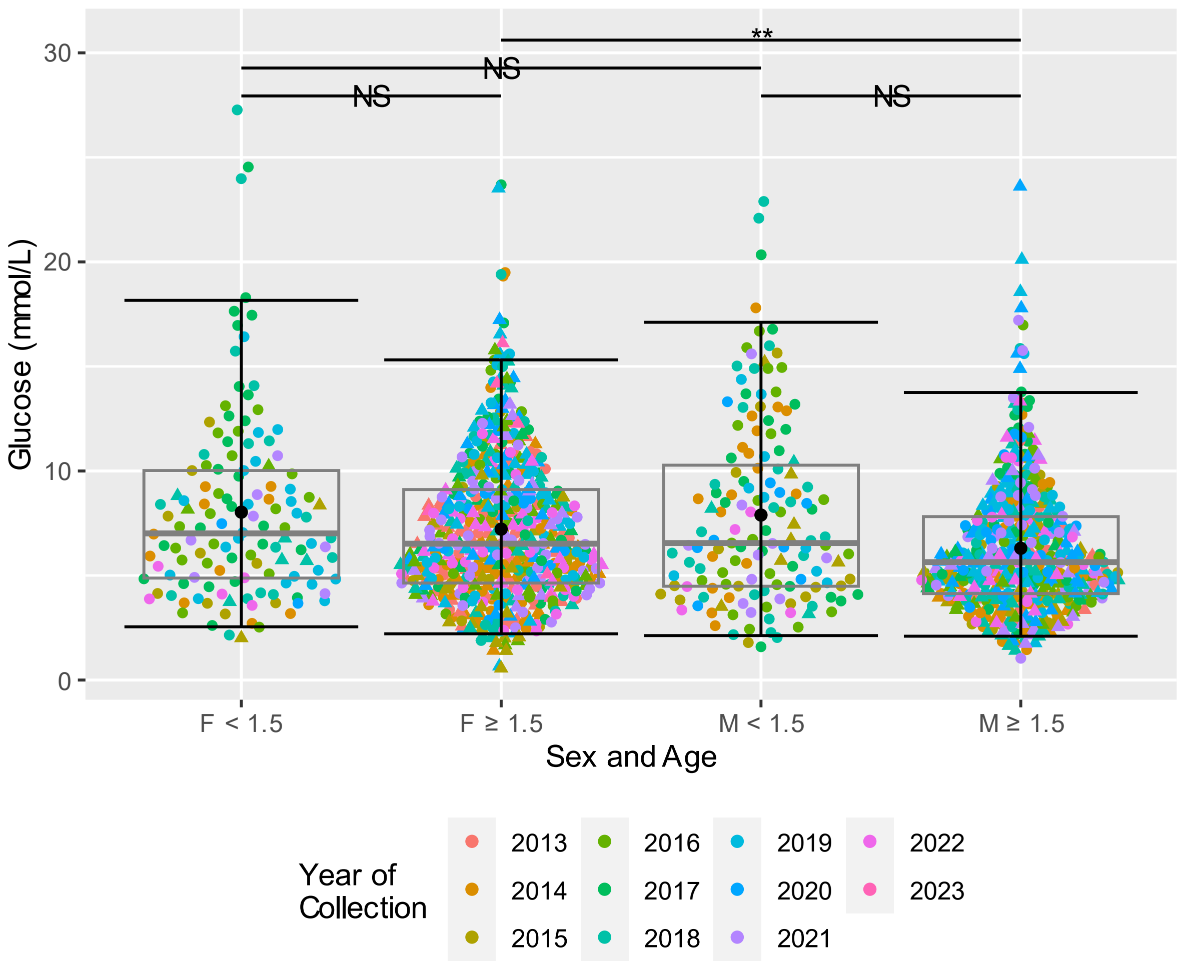

Figure S1 17. Glucose levels according to sex and age in common marmosets. Boxes indicate median and quartile ranges; black point indicates arithmetic mean and error bars indicate the normal range. Symbol colours indicate year of collection, round symbols indicate animals with outdoor access and triangles indicate indoor housed animals; NS = not significant, asterisks indicate statistical significance levels * < 0.001, ** < 0.0001, *** < 0.00001, **** < 0.000001

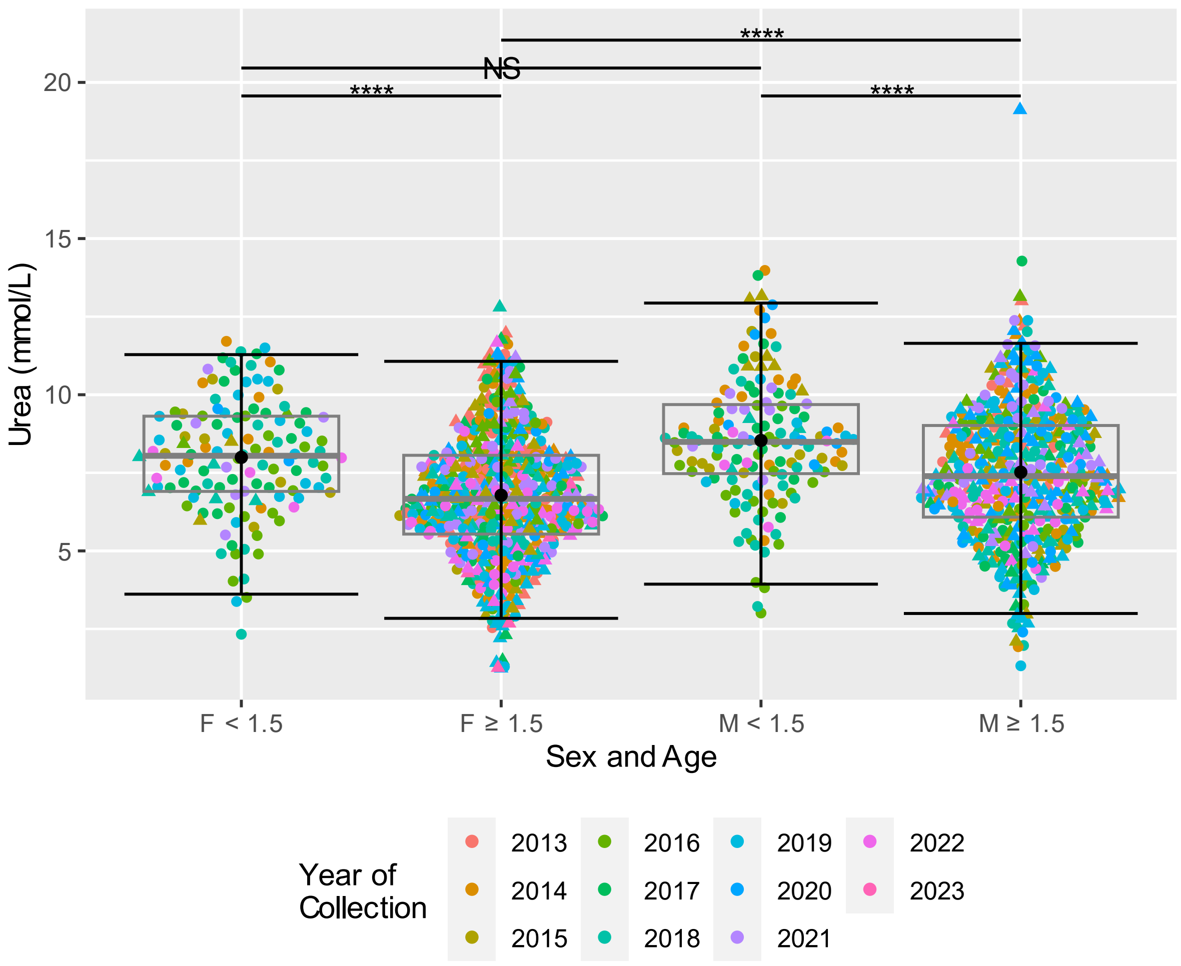

Figure S1 18. Urea levels according to sex and age in common marmosets. Boxes indicate median and quartile ranges; black point indicates arithmetic mean and error bars indicate the normal range. Symbol colours indicate year of collection, round symbols indicate animals with outdoor access and triangles indicate indoor housed animals; NS = not significant, asterisks indicate statistical significance levels * < 0.001, ** < 0.0001, *** < 0.00001, **** < 0.000001

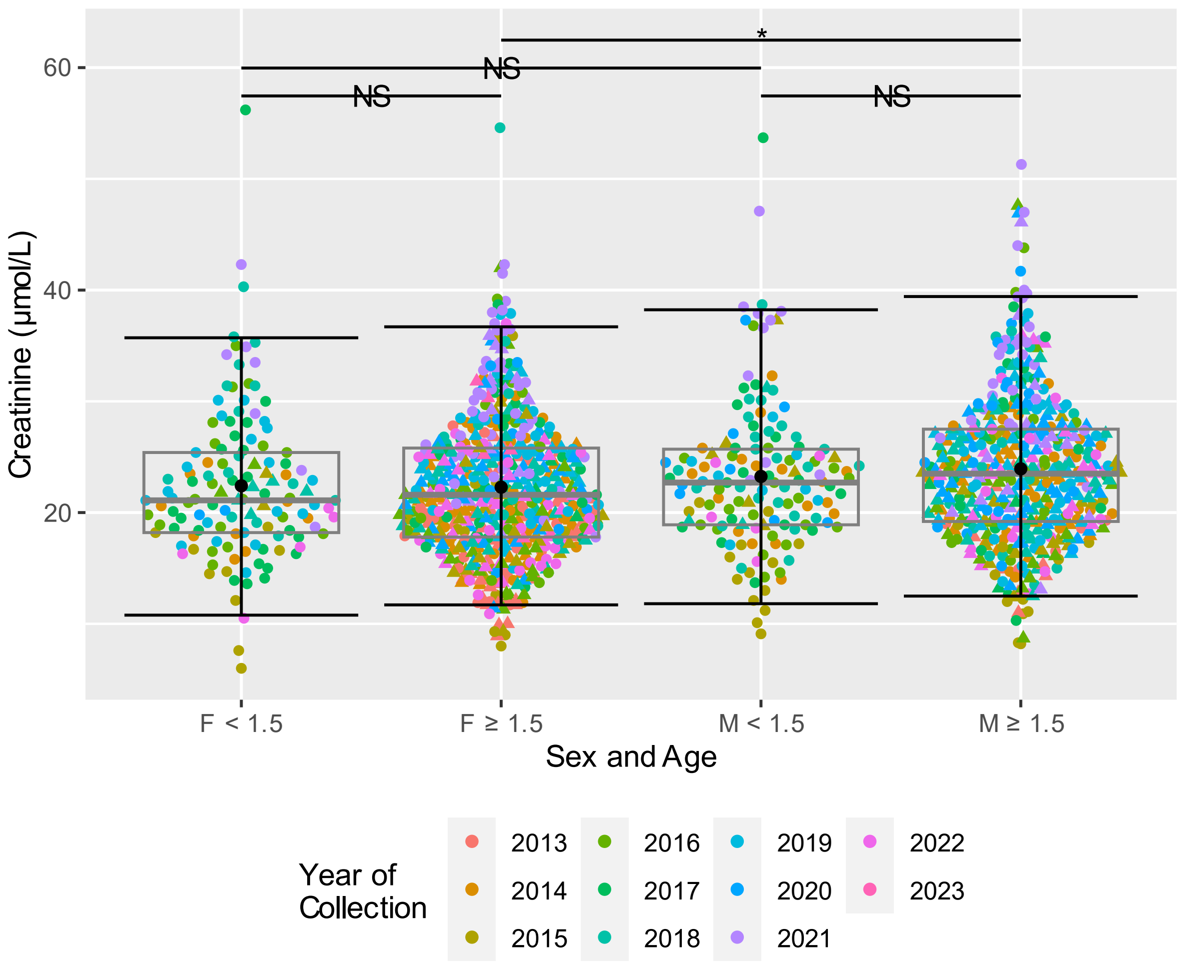

Figure S1 19. Creatinine levels according to sex and age in common marmosets. Boxes indicate median and quartile ranges; black point indicates arithmetic mean and error bars indicate the normal range. Symbol colours indicate year of collection, round symbols indicate animals with outdoor access and triangles indicate indoor housed animals; NS = not significant, asterisks indicate statistical significance levels * < 0.001, ** < 0.0001, *** < 0.00001, **** < 0.000001

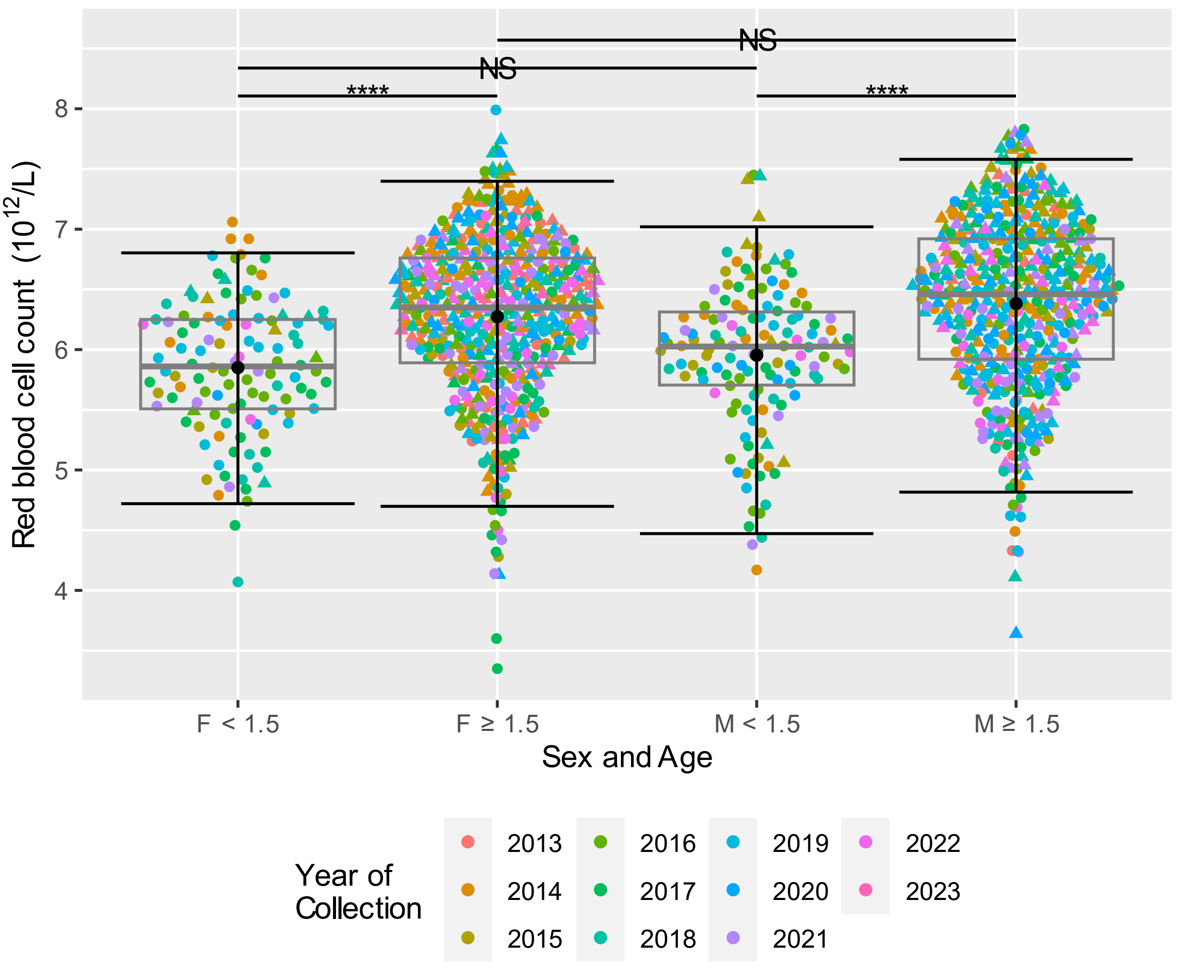

Figure S1 20. Red blood cell count according to sex and age in common marmosets. Boxes indicate median and quartile ranges; black point indicates arithmetic mean and error bars indicate the normal range. Symbol colours indicate year of collection, round symbols indicate animals with outdoor access and triangles indicate indoor housed animals; NS = not significant, asterisks indicate statistical significance levels * < 0.001, ** < 0.0001, *** < 0.00001, **** < 0.000001

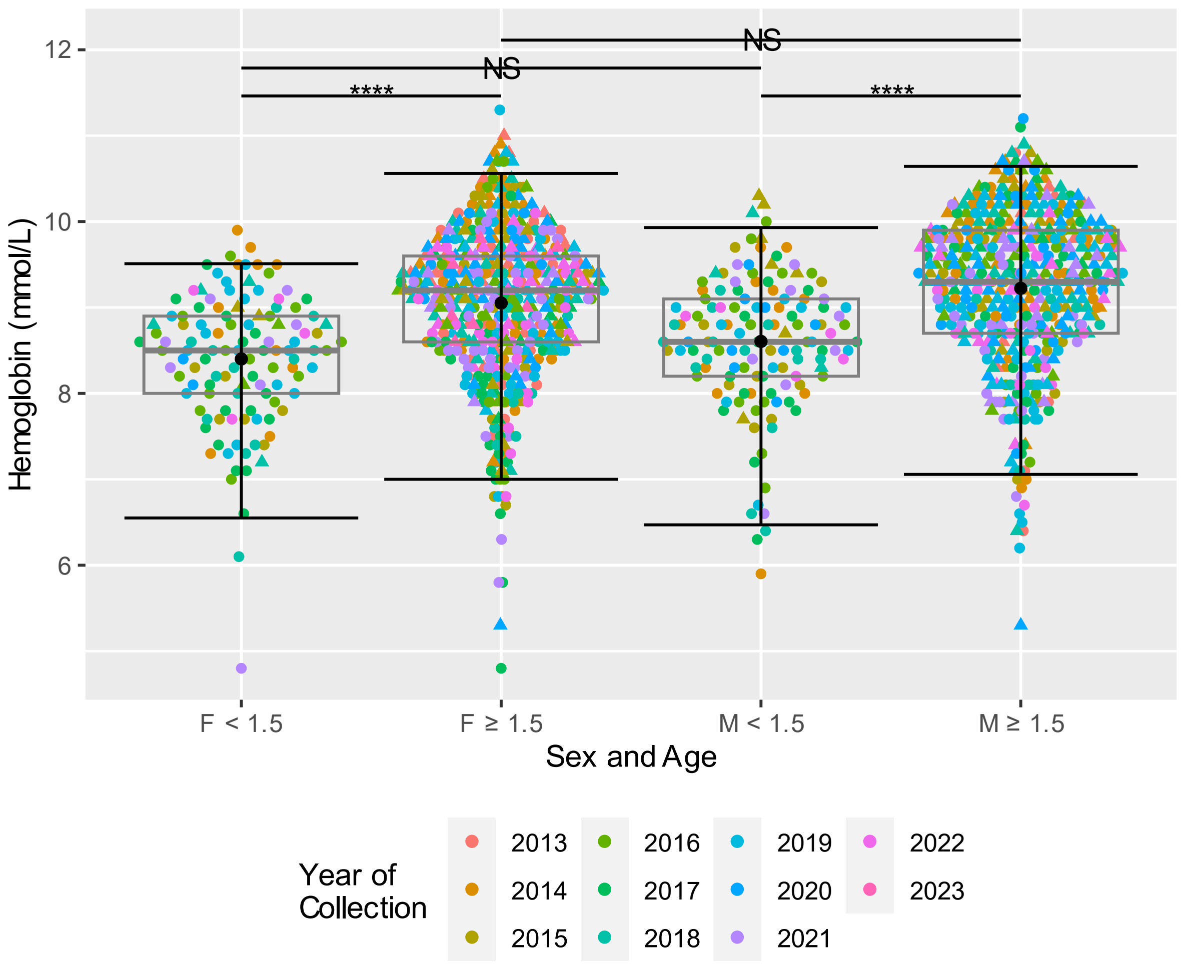

Figure S1 21. Haemoglobin levels according to sex and age in common marmosets. Boxes indicate median and quartile ranges; black point indicates arithmetic mean and error bars indicate the normal range. Symbol colours indicate year of collection, round symbols indicate animals with outdoor access and triangles indicate indoor housed animals; NS = not significant, asterisks indicate statistical significance levels * < 0.001, ** < 0.0001, *** < 0.00001, **** < 0.000001

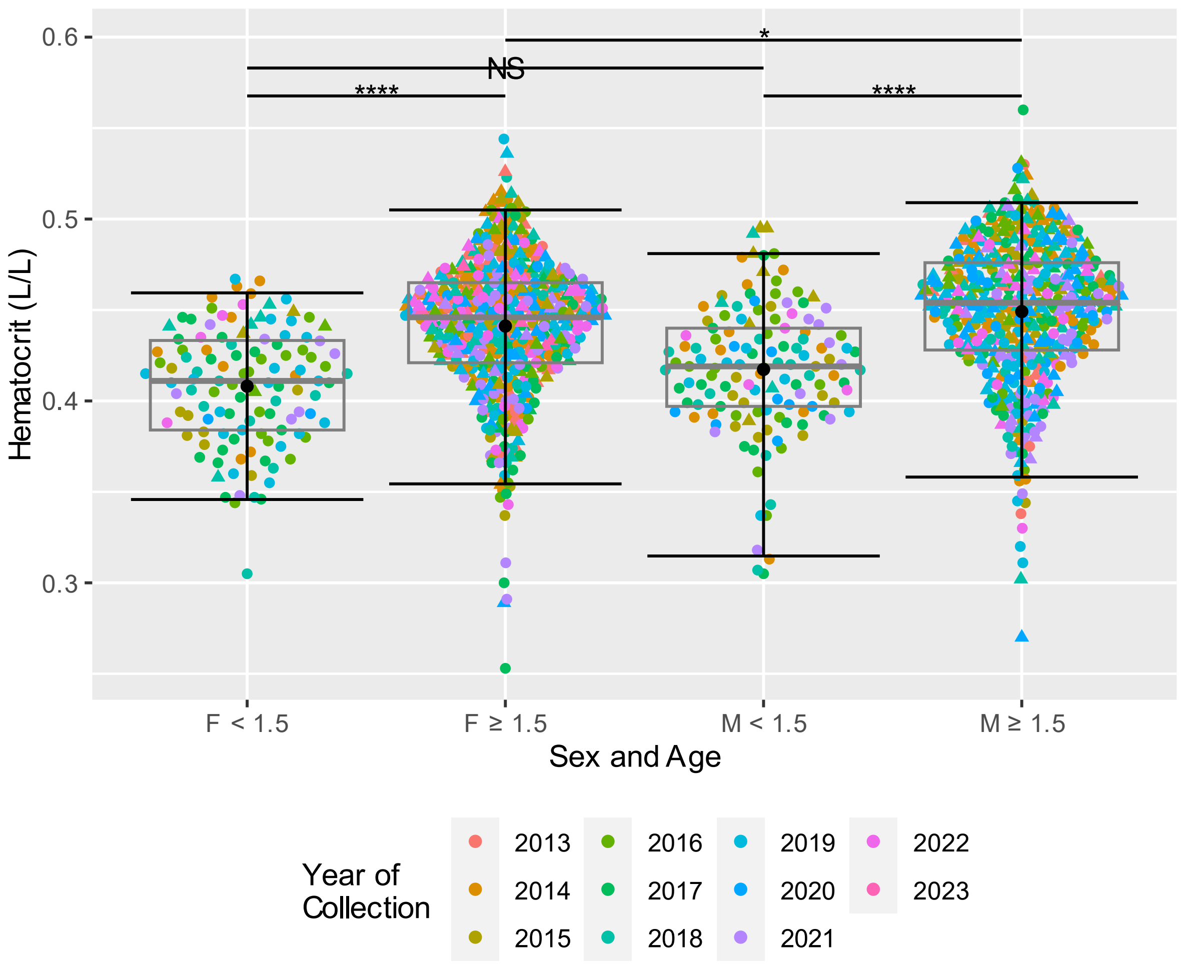

Figure S1 22. Haematocrit according to sex and age in common marmosets. Boxes indicate median and quartile ranges; black point indicates arithmetic mean and error bars indicate the normal range. Symbol colours indicate year of collection, round symbols indicate animals with outdoor access and triangles indicate indoor housed animals; NS = not significant, asterisks indicate statistical significance levels * < 0.001, ** < 0.0001, *** < 0.00001, **** < 0.000001

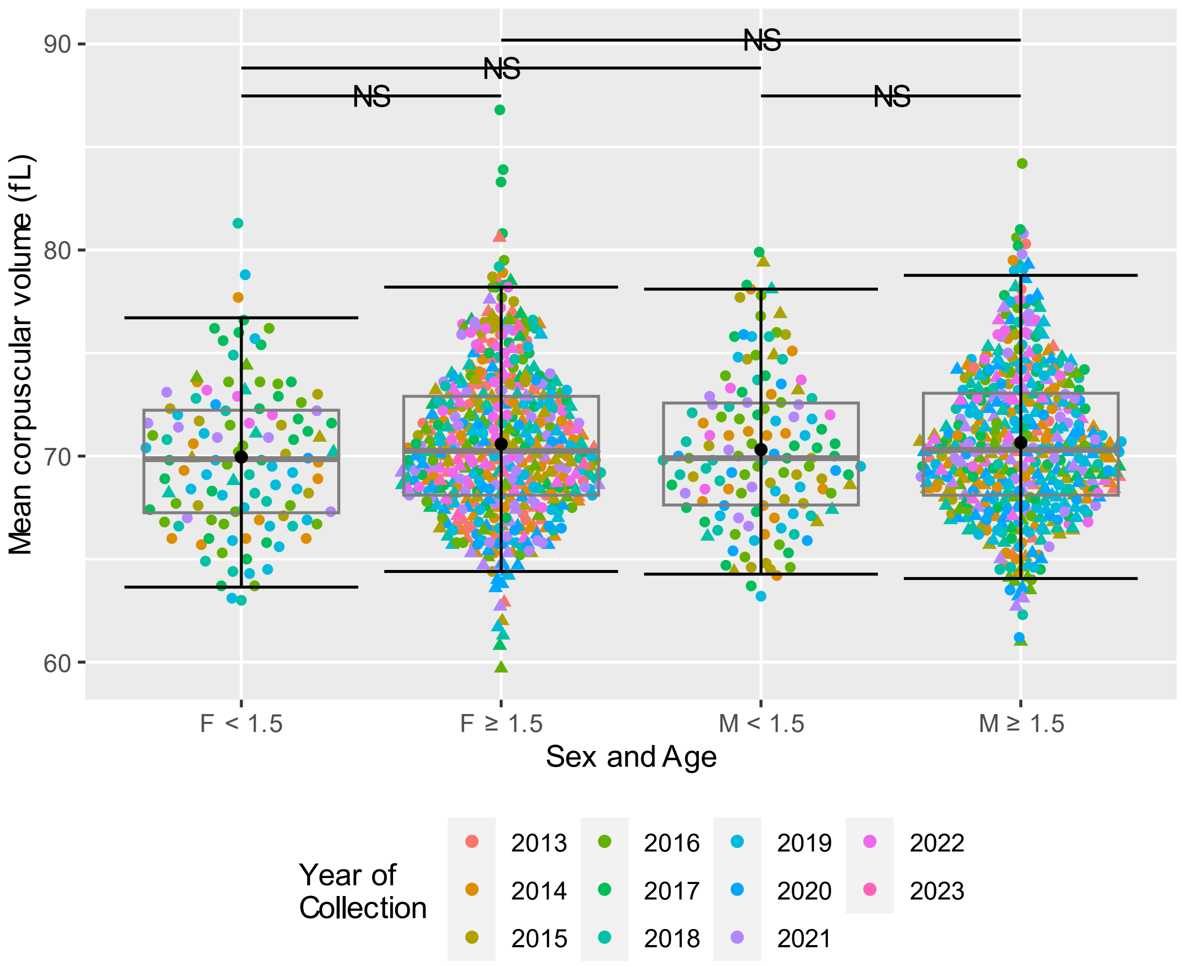

Figure S1 23. Mean Corpescular Volume according to sex and age in common marmosets. Boxes indicate median and quartile ranges; black point indicates arithmetic mean and error bars indicate the normal range. Symbol colours indicate year of collection, round symbols indicate animals with outdoor access and triangles indicate indoor housed animals; NS = not significant, asterisks indicate statistical significance levels * < 0.001, ** < 0.0001, *** < 0.00001, **** < 0.000001

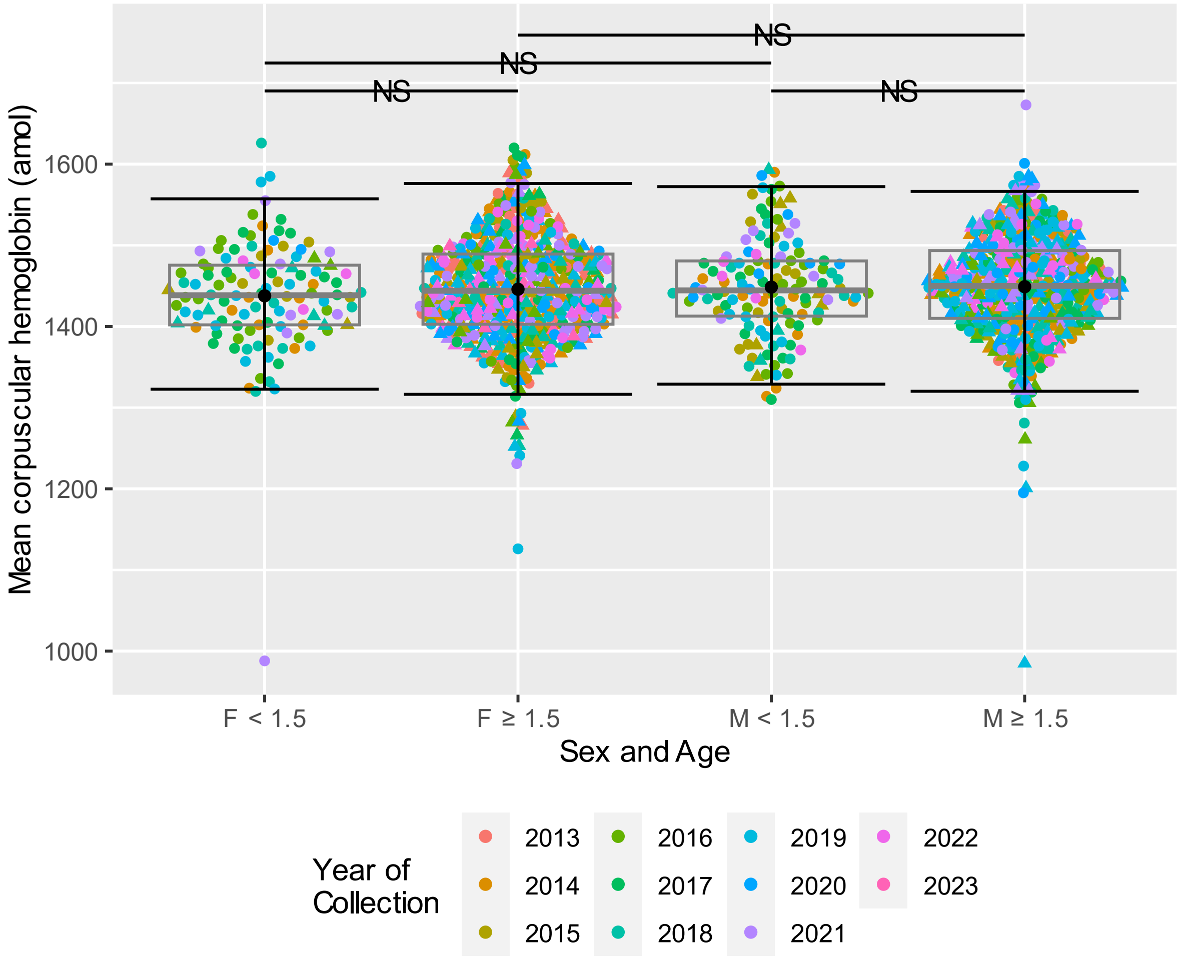

Figure S1 24. Mean Corpescular Haemoglobin according to sex and age in common marmosets. Boxes indicate median and quartile ranges; black point indicates arithmetic mean and error bars indicate the normal range. Symbol colours indicate year of collection, round symbols indicate animals with outdoor access and triangles indicate indoor housed animals; NS = not significant, asterisks indicate statistical significance levels * < 0.001, ** < 0.0001, *** < 0.00001, **** < 0.000001

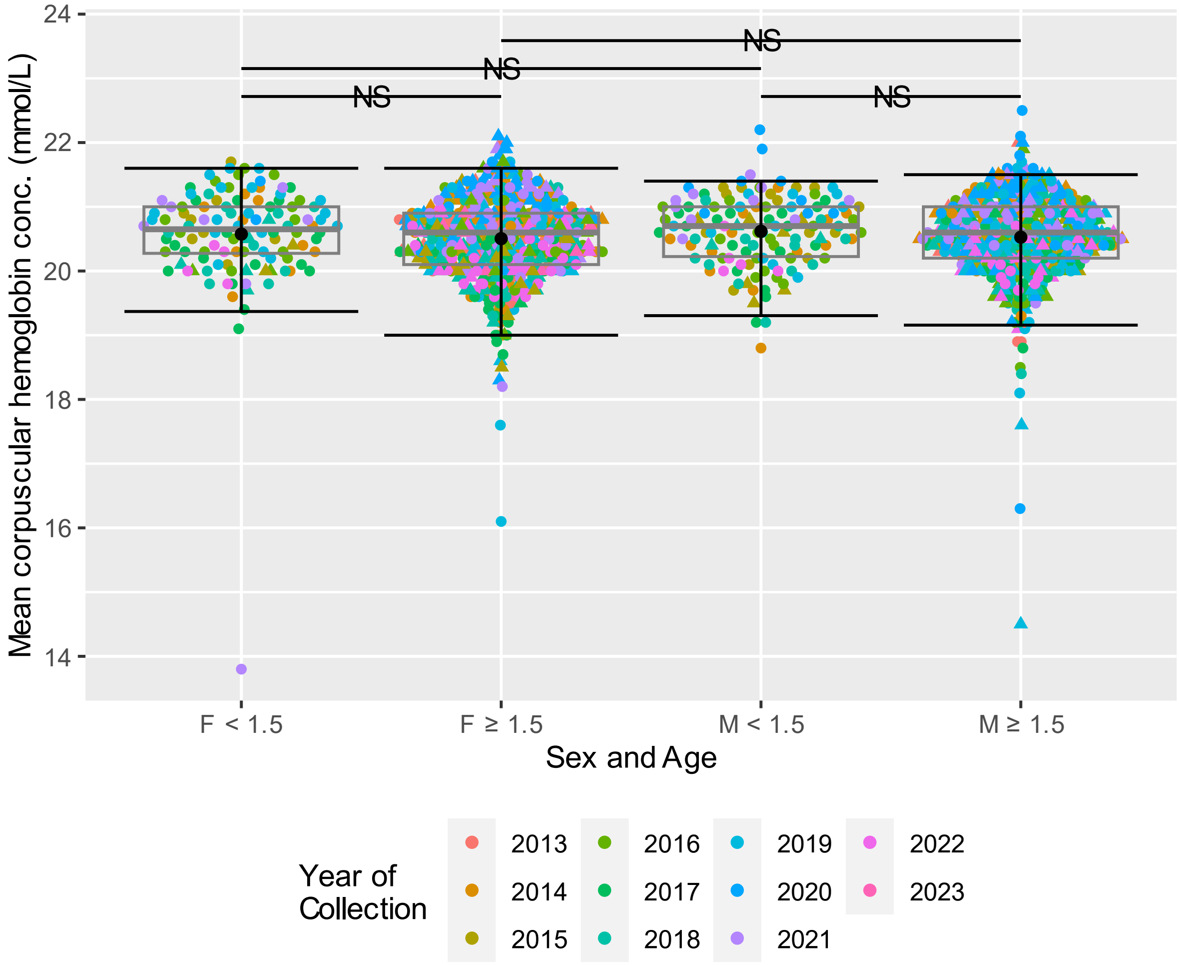

Figure S1 25. Mean Corpescular Haemoglobin Concentration according to sex and age in common marmosets. Boxes indicate median and quartile ranges; black point indicates arithmetic mean and error bars indicate the normal range. Symbol colours indicate year of collection, round symbols indicate animals with outdoor access and triangles indicate indoor housed animals; NS = not significant, asterisks indicate statistical significance levels * < 0.001, ** < 0.0001, *** < 0.00001, **** < 0.000001

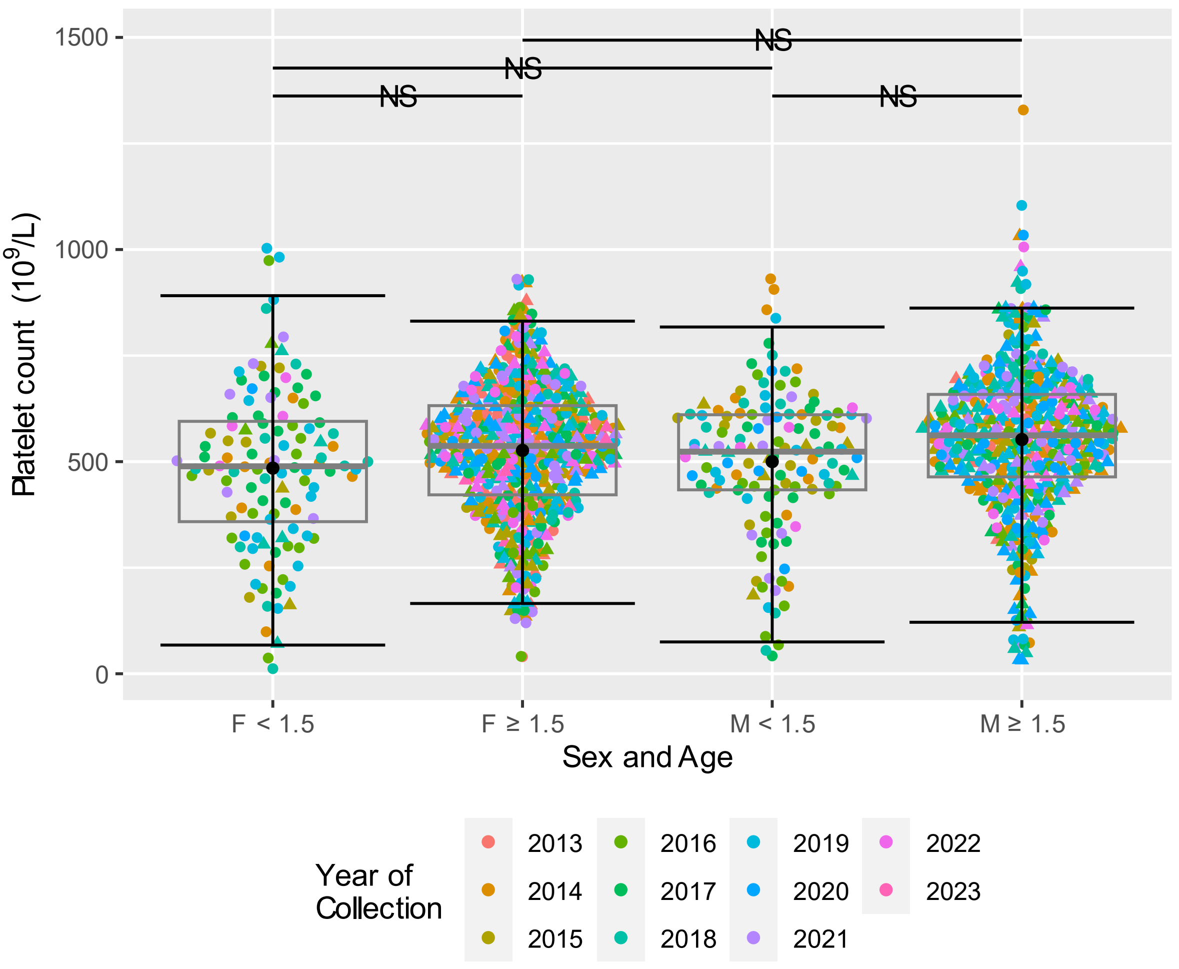

Figure S1 26. Platelet Count according to sex and age in common marmosets. Boxes indicate median and quartile ranges; black point indicates arithmetic mean and error bars indicate the normal range. Symbol colours indicate year of collection, round symbols indicate animals with outdoor access and triangles indicate indoor housed animals; NS = not significant, asterisks indicate statistical significance levels * < 0.001, ** < 0.0001, *** < 0.00001, **** < 0.000001

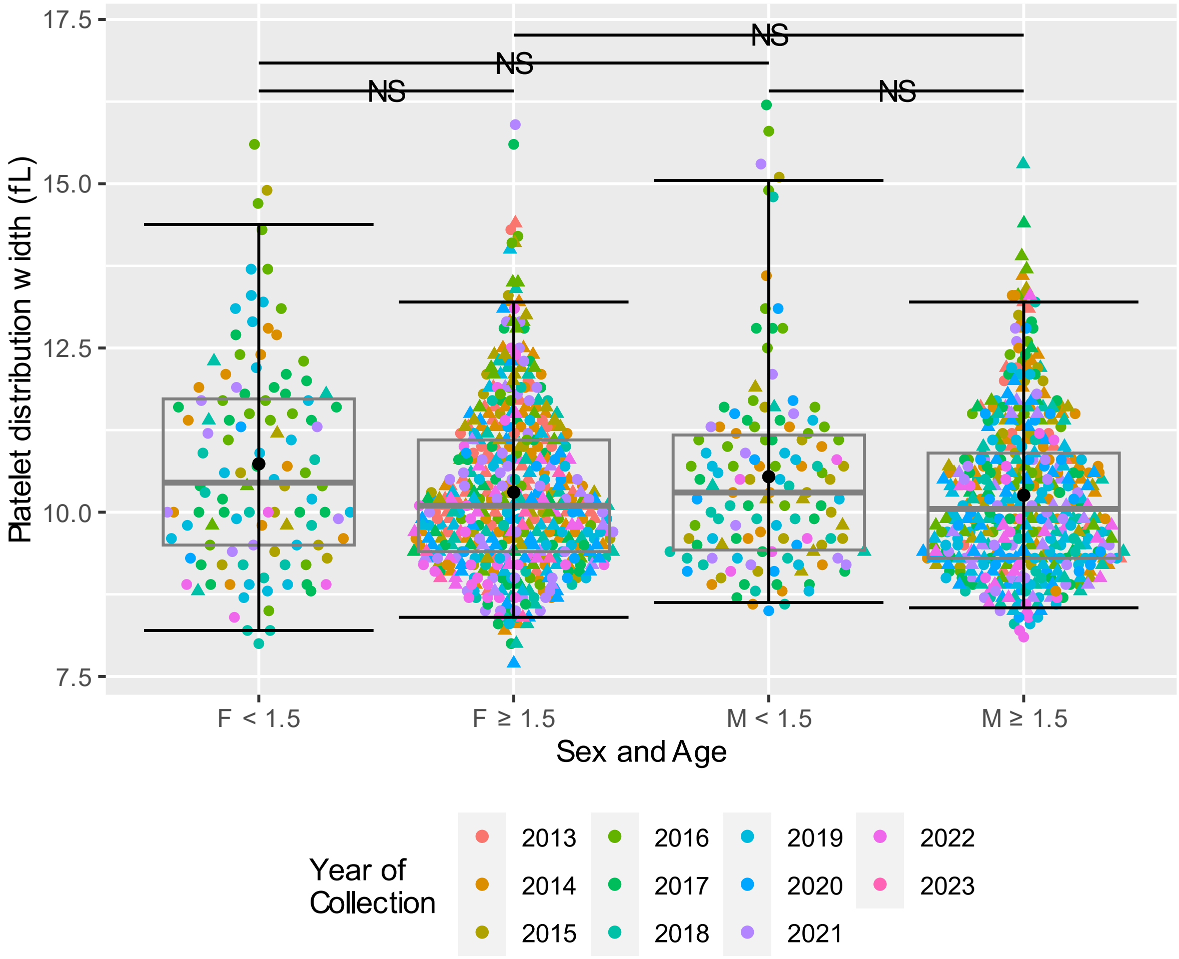

Figure S1 27. Platelet distribution width according to sex and age in common marmosets. Boxes indicate median and quartile ranges; black point indicates arithmetic mean and error bars indicate the normal range. Symbol colours indicate year of collection, round symbols indicate animals with outdoor access and triangles indicate indoor housed animals; NS = not significant, asterisks indicate statistical significance levels * < 0.001, ** < 0.0001, *** < 0.00001, **** < 0.000001

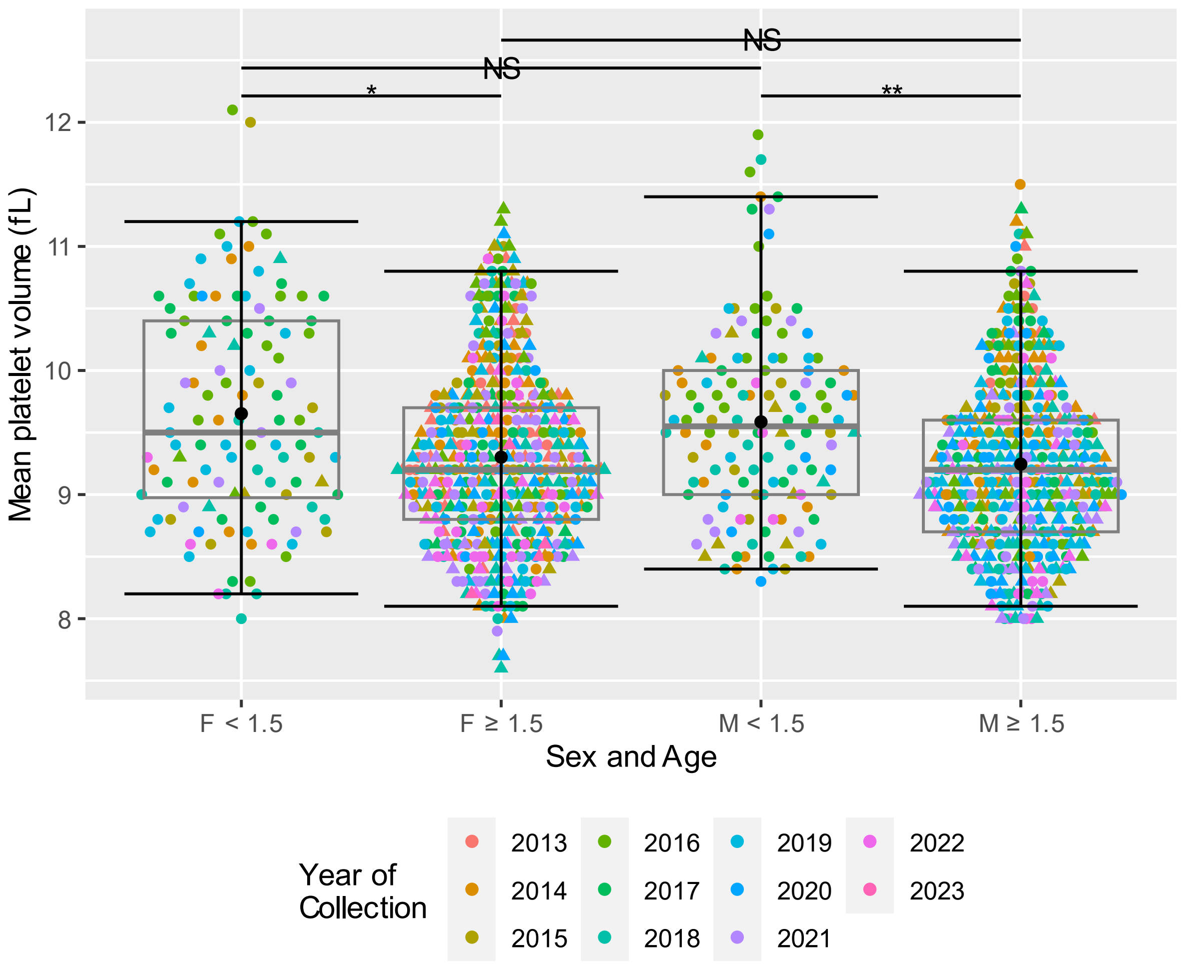

Figure S1 28. Mean Platelet Volume according to sex and age in common marmosets. Boxes indicate median and quartile ranges; black point indicates arithmetic mean and error bars indicate the normal range. Symbol colours indicate year of collection, round symbols indicate animals with outdoor access and triangles indicate indoor housed animals; NS = not significant, asterisks indicate statistical significance levels * < 0.001, ** < 0.0001, *** < 0.00001, **** < 0.000001

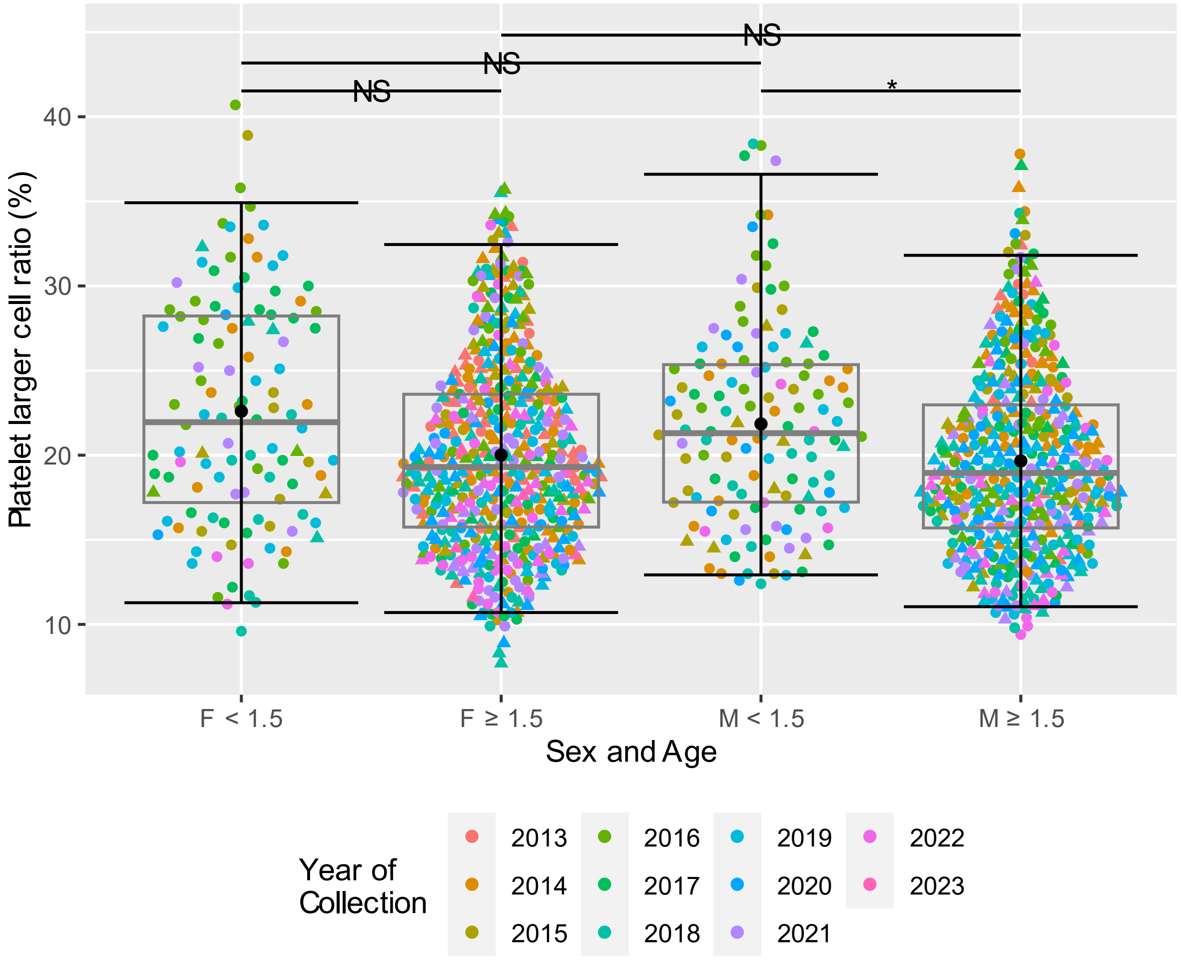

Figure S1 29. Platelet Larger Cell Ratio according to sex and age in common marmosets. Boxes indicate median and quartile ranges; black point indicates arithmetic mean and error bars indicate the normal range. Symbol colours indicate year of collection, round symbols indicate animals with outdoor access and triangles indicate indoor housed animals; NS = not significant, asterisks indicate statistical significance levels * < 0.001, ** < 0.0001, *** < 0.00001, **** < 0.000001

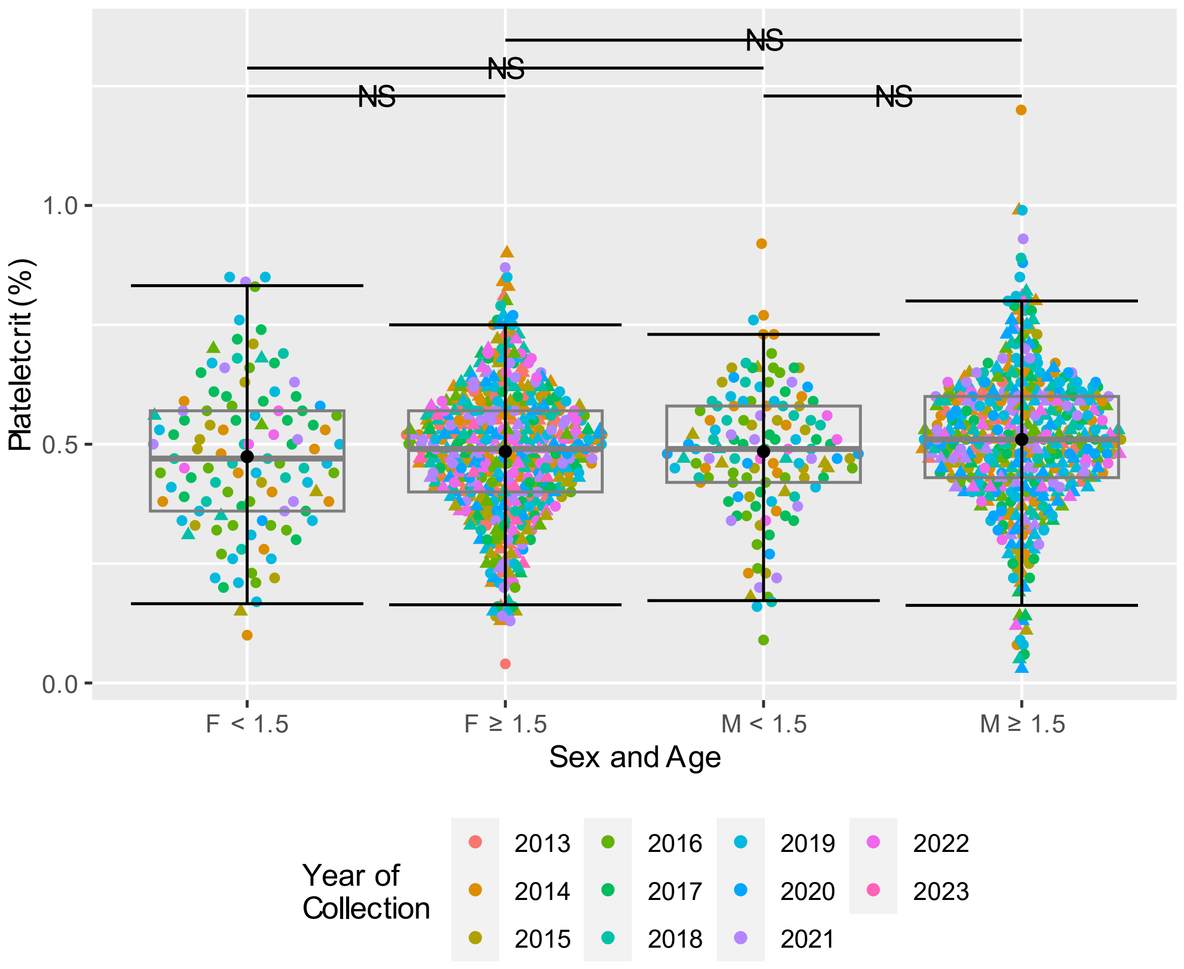

Figure S1 30. Plateletcrit according to sex and age in common marmosets. Boxes indicate median and quartile ranges; black point indicates arithmetic mean and error bars indicate the normal range. Symbol colours indicate year of collection, round symbols indicate animals with outdoor access and triangles indicate indoor housed animals; NS = not significant, asterisks indicate statistical significance levels * < 0.001, ** < 0.0001, *** < 0.00001, **** < 0.000001

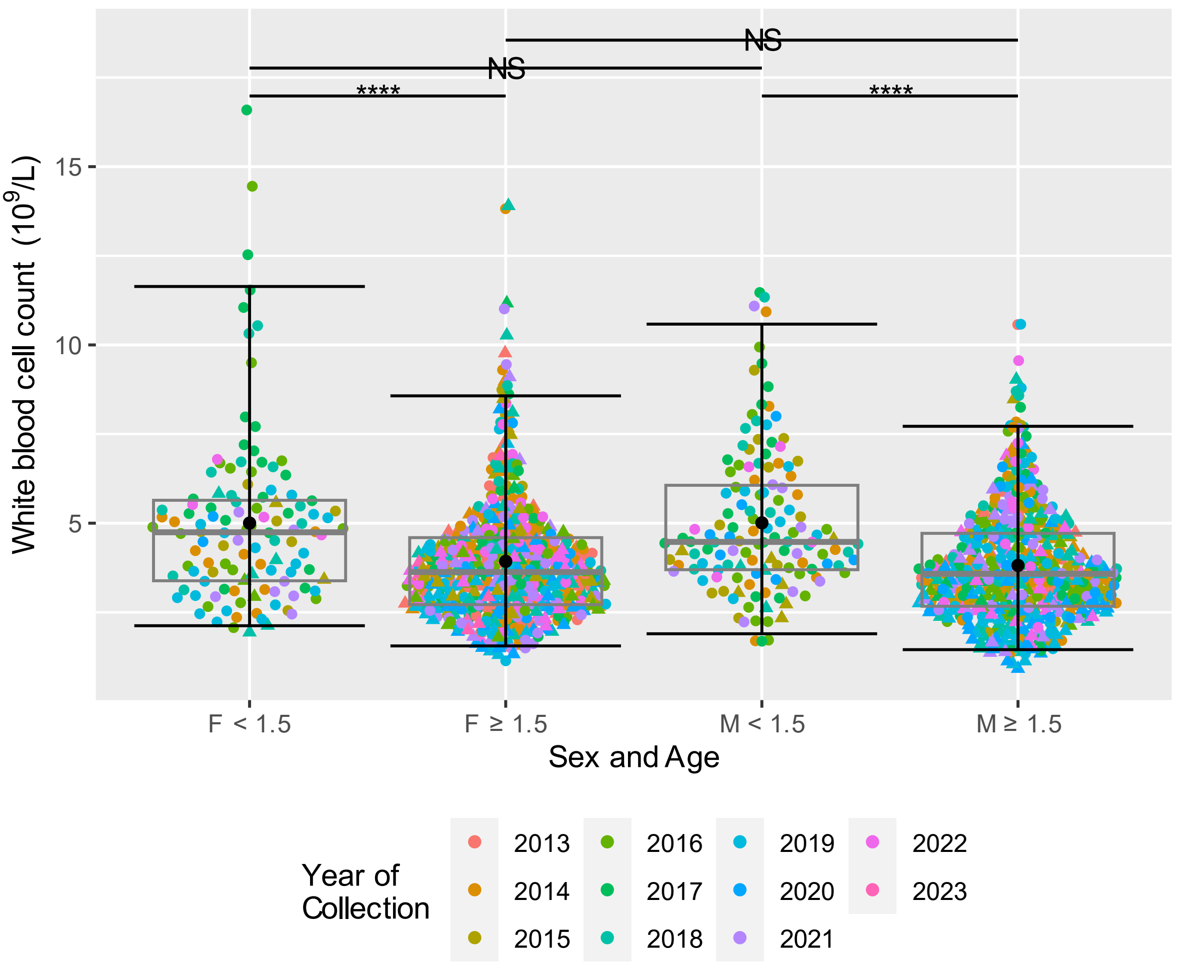

Figure S1 31. White Blood Cell count according to sex and age in common marmosets. Boxes indicate median and quartile ranges; black point indicates arithmetic mean and error bars indicate the normal range. Symbol colours indicate year of collection, round symbols indicate animals with outdoor access and triangles indicate indoor housed animals; NS = not significant, asterisks indicate statistical significance levels * < 0.001, ** < 0.0001, *** < 0.00001, **** < 0.000001

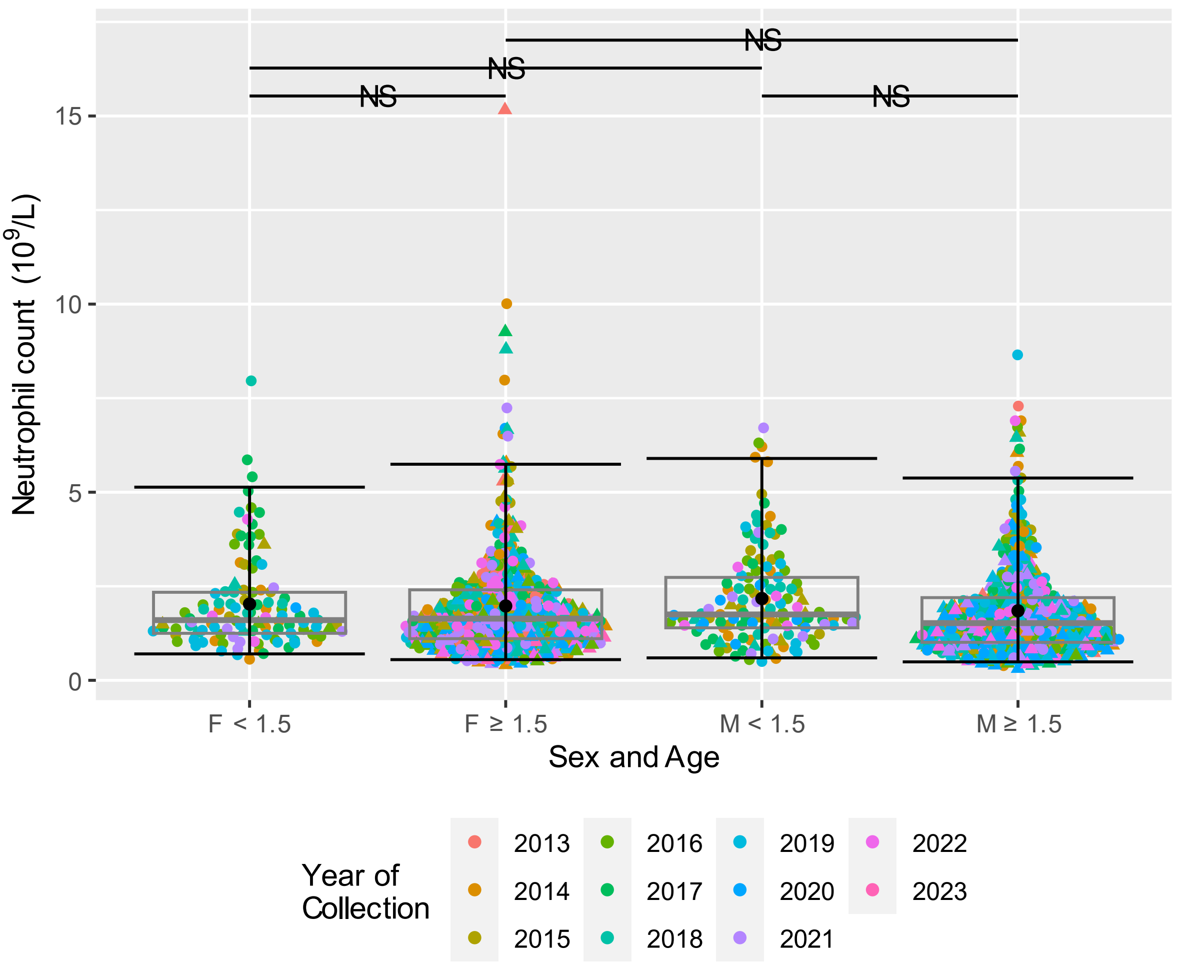

Figure S1 32. Neutrophil count according to sex and age in common marmosets. Boxes indicate median and quartile ranges; black point indicates arithmetic mean and error bars indicate the normal range. Symbol colours indicate year of collection, round symbols indicate animals with outdoor access and triangles indicate indoor housed animals; NS = not significant, asterisks indicate statistical significance levels * < 0.001, ** < 0.0001, *** < 0.00001, **** < 0.000001

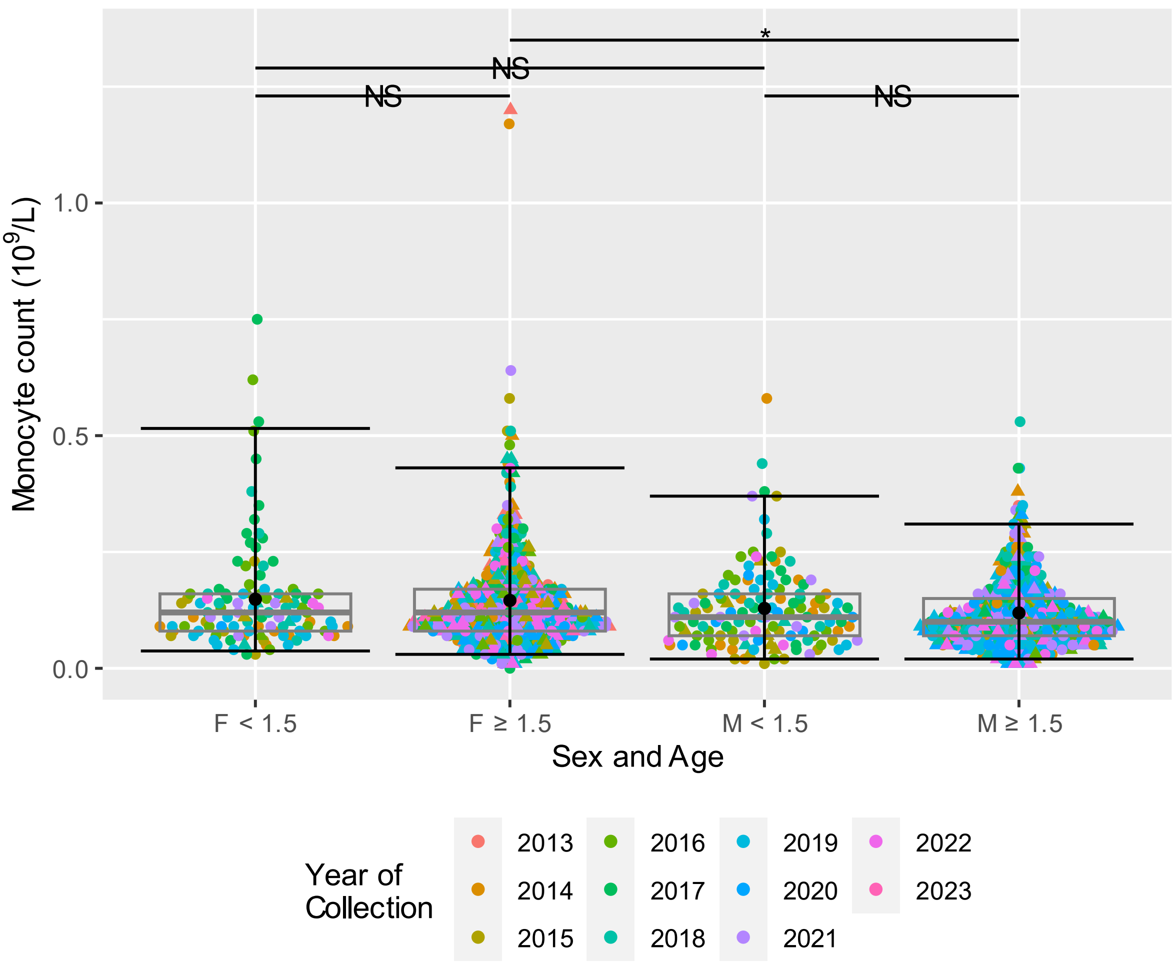

Figure S1 33. Monocyte count according to sex and age in common marmosets. Boxes indicate median and quartile ranges; black point indicates arithmetic mean and error bars indicate the normal range. Symbol colours indicate year of collection, round symbols indicate animals with outdoor access and triangles indicate indoor housed animals; NS = not significant, asterisks indicate statistical significance levels * < 0.001, ** < 0.0001, *** < 0.00001, **** < 0.000001

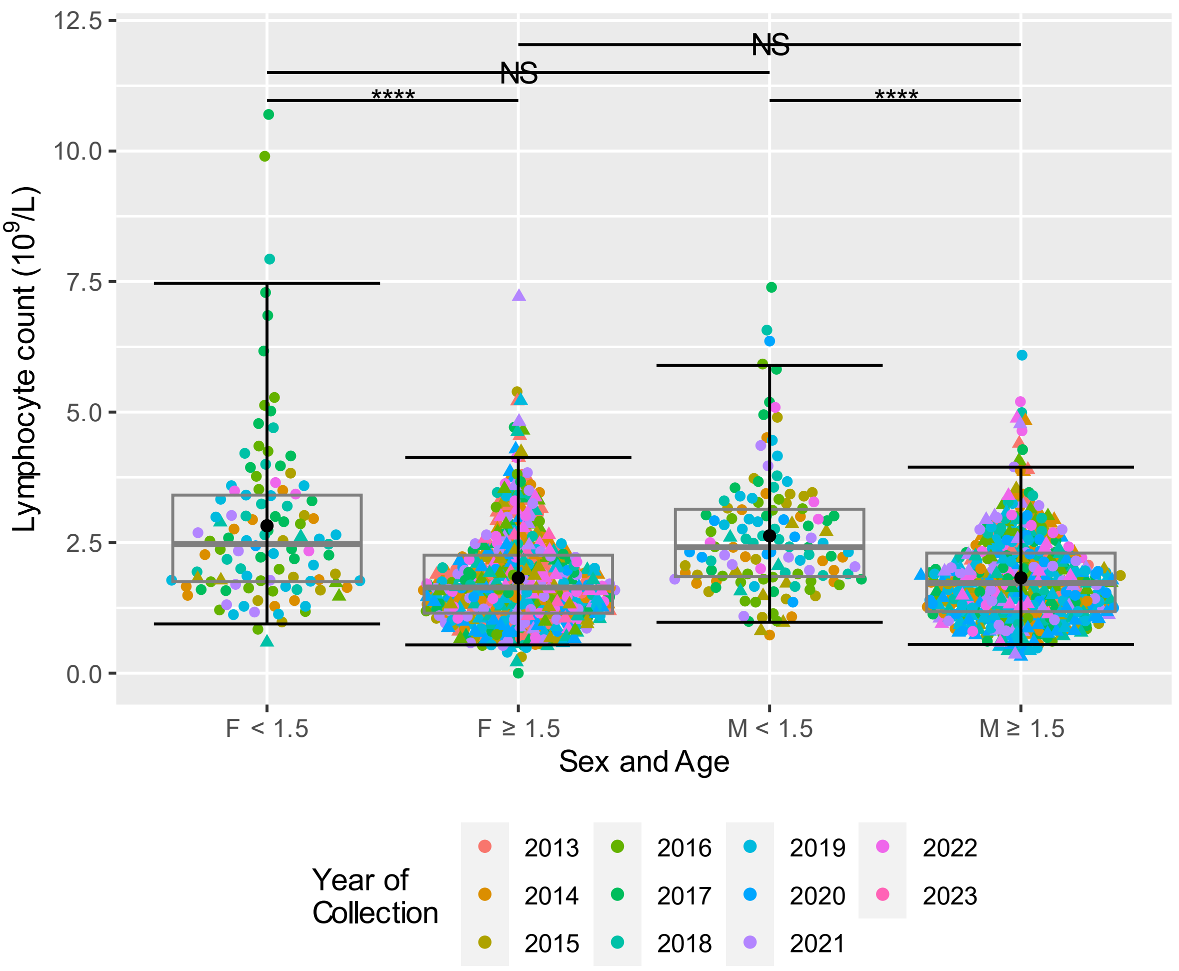

Figure S1 34. Lymphocyte count according to sex and age in common marmosets. Boxes indicate median and quartile ranges; black point indicates arithmetic mean and error bars indicate the normal range. Symbol colours indicate year of collection, round symbols indicate animals with outdoor access and triangles indicate indoor housed animals; NS = not significant, asterisks indicate statistical significance levels * < 0.001, ** < 0.0001, *** < 0.00001, **** < 0.000001

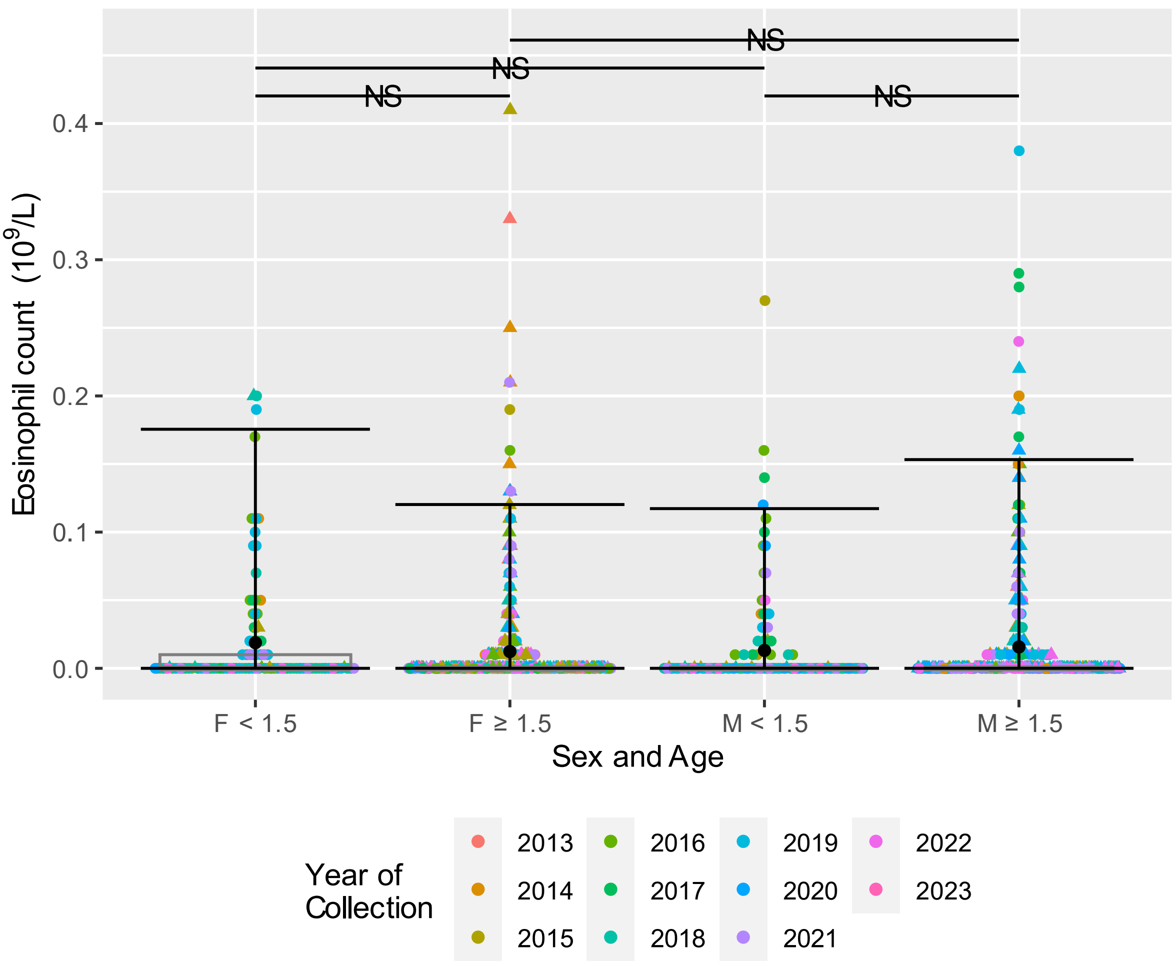

Figure S1 35. Eosinophil count according to sex and age in common marmosets. Boxes indicate median and quartile ranges; black point indicates arithmetic mean and error bars indicate the normal range. Symbol colours indicate year of collection, round symbols indicate animals with outdoor access and triangles indicate indoor housed animals; NS = not significant, asterisks indicate statistical significance levels * < 0.001, ** < 0.0001, *** < 0.00001, **** < 0.000001

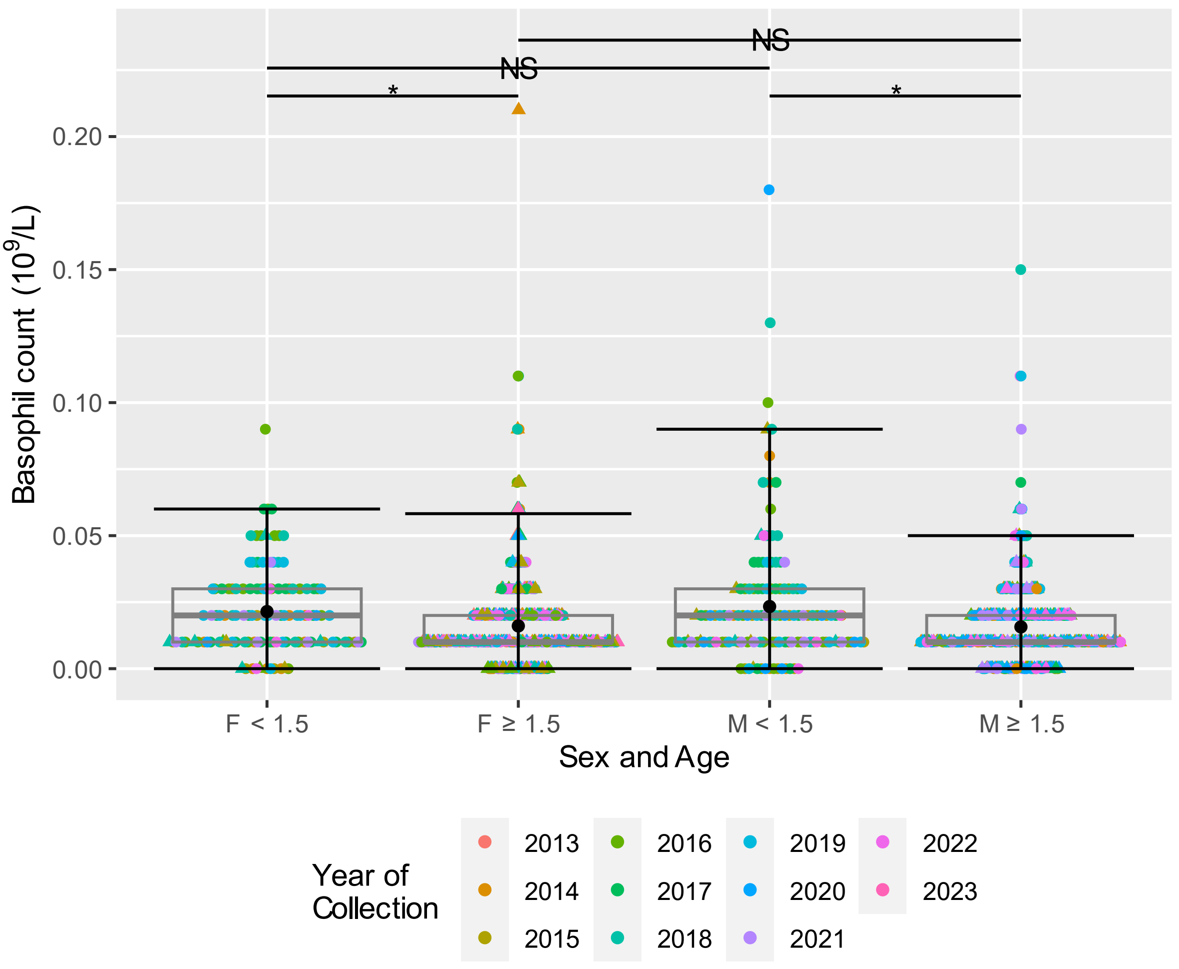

Figure S1 36. Basophil count according to sex and age in common marmosets. Boxes indicate median and quartile ranges; black point indicates arithmetic mean and error bars indicate the normal range. Symbol colours indicate year of collection, round symbols indicate animals with outdoor access and triangles indicate indoor housed animals; NS = not significant, asterisks indicate statistical significance levels * < 0.001, ** < 0.0001, *** < 0.00001, **** < 0.000001
