# Supplementary material for: Hematological and Serum Biochemical Reference Intervals for Alphaxalone Sedated Common Marmosets (Callithrix jacchus)
Source: Animals (Basel). 2024 Mar 3;14(5):790. doi: 10.3390/ani14050790 (PMC10930924; doi:10.3390/ani14050790)
Supplement: Supplementary file 1 [file animals-14-00790-s001.zip › Figure S2.pdf]

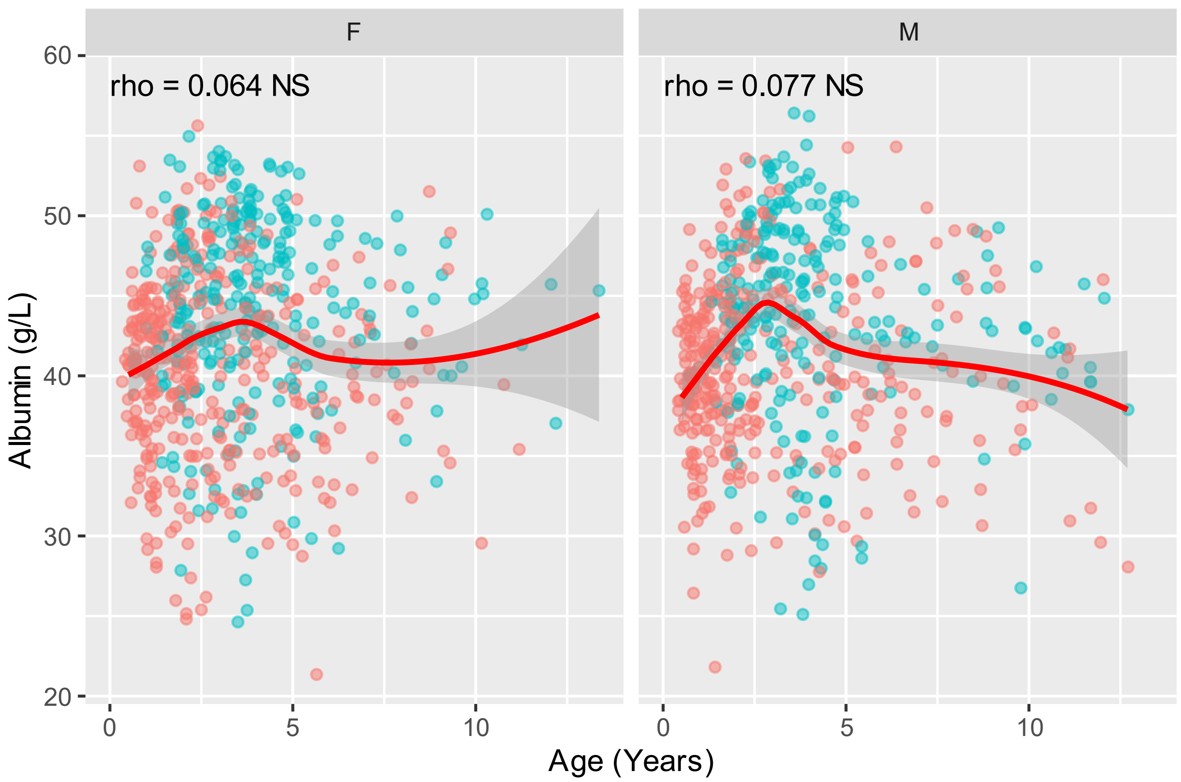


Figure S2 1. Albumin levels according to sex and age in common marmosets. Points represent individual animals (red = outdoor access, cyan = indoor only). The red line indicates a Loess moving average and the grey area indicates the 95% confidence interval. Rho is Spearman's rank correlation for parameter and Age; NS = not significant, asterisks indicate statistical significance levels * < 0.001, ** < 0.0001, *** < 0.00001, **** < 0.000001.

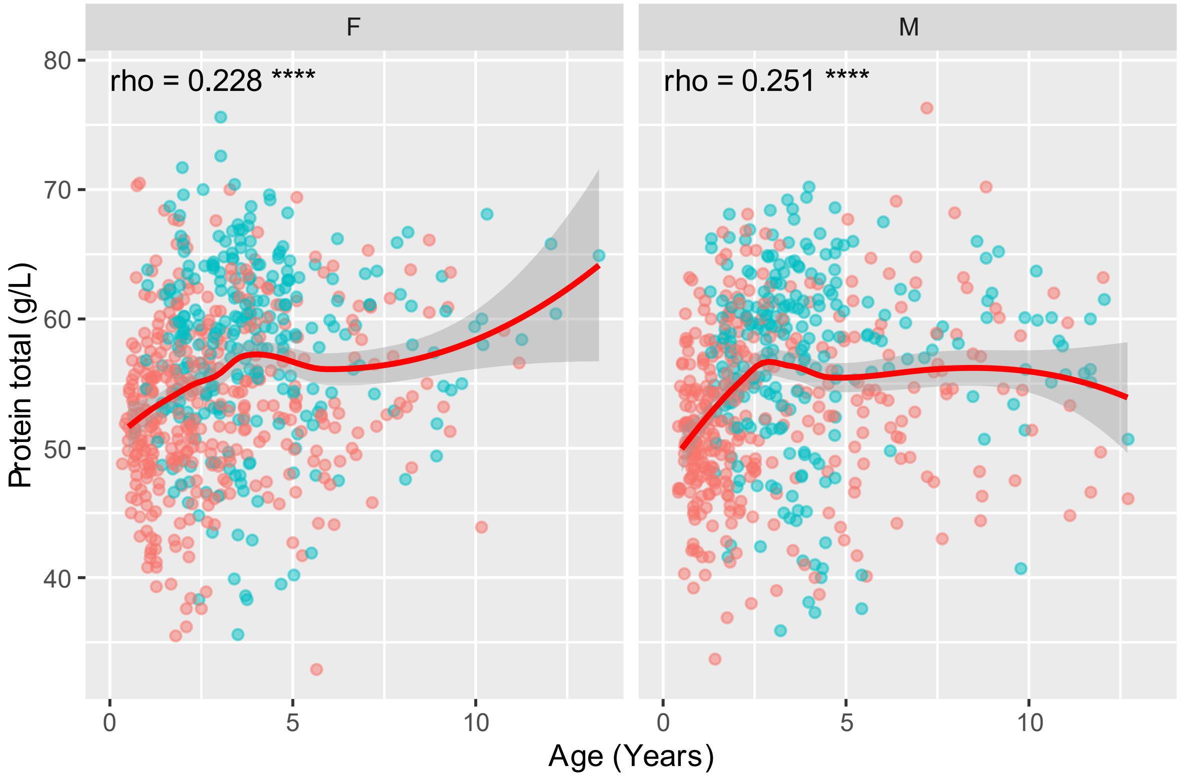


Figure S2 2. Total Protein levels according to sex and age in common marmosets. Points represent individual animals (red = outdoor access, cyan = indoor only). The red line indicates a Loess moving average and the grey area indicates the 95% confidence interval. Rho is Spearman's rank correlation for parameter and Age; NS = not significant, asterisks indicate statistical significance levels * < 0.001, ** < 0.0001, *** < 0.00001, **** < 0.000001.

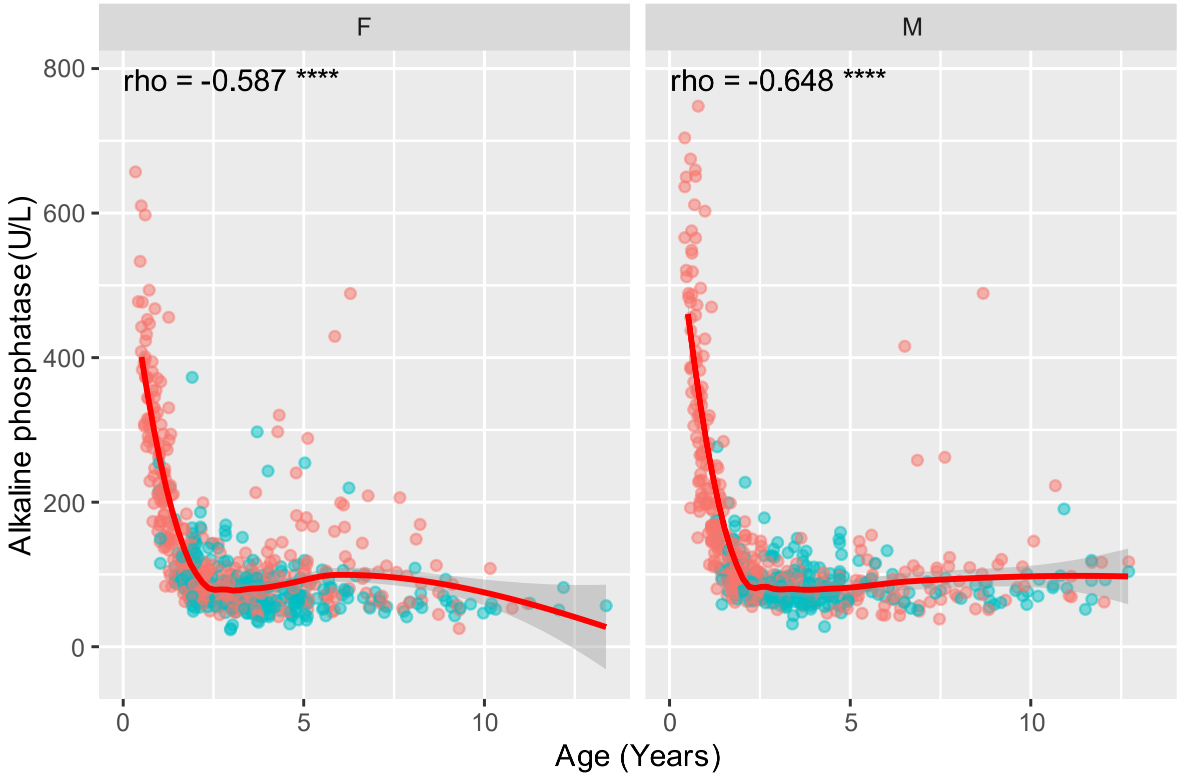


Figure S2 3. Alkaline Phosphatase levels according to sex and age in common marmosets. Points represent individual animals (red = outdoor access, cyan = indoor only). The red line indicates a Loess moving average and the grey area indicates the 95% confidence interval. Rho is Spearman's rank correlation for parameter and Age; NS = not significant, asterisks indicate statistical significance levels * < 0.001, ** < 0.0001, *** < 0.00001, **** < 0.000001.

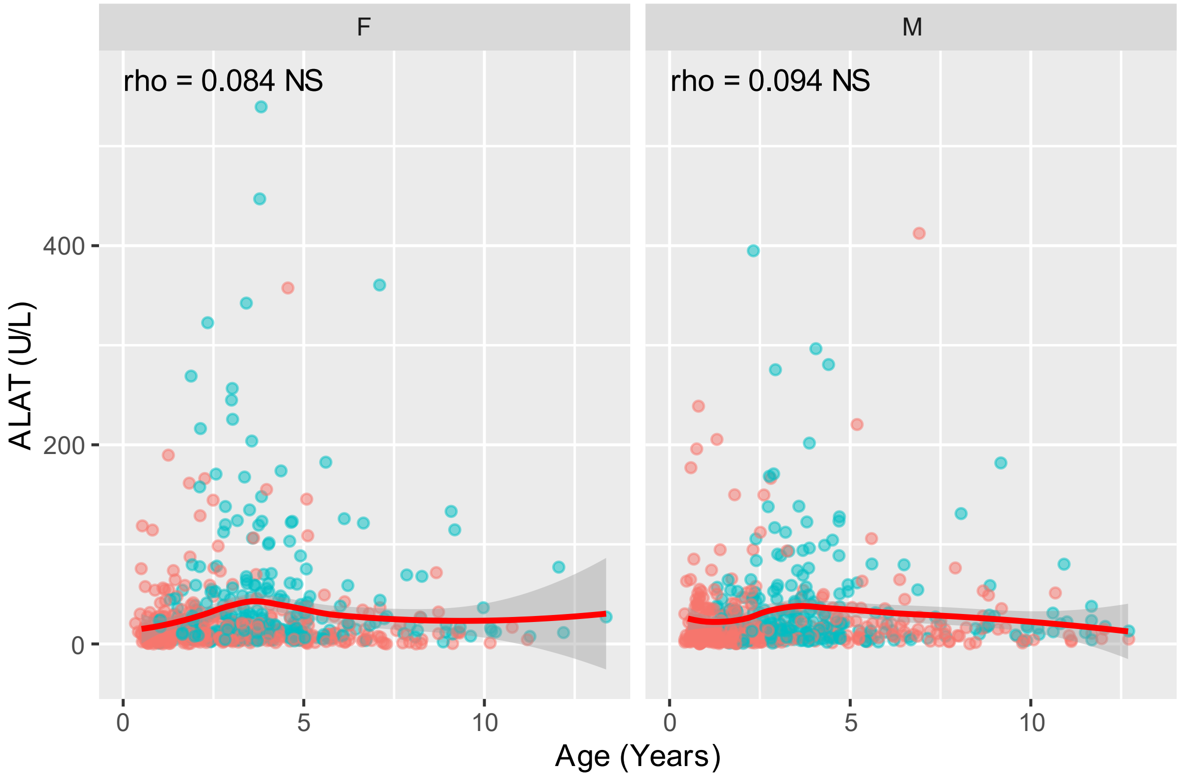


Figure S2 4. ALAT levels according to sex and age in common marmosets. Points represent individual animals (red = outdoor access, cyan = indoor only). The red line indicates a Loess moving average and the grey area indicates the 95% confidence interval. Rho is Spearman's rank correlation for parameter and Age; NS = not significant, asterisks indicate statistical significance levels * < 0.001, ** < 0.0001, *** < 0.00001, **** < 0.000001.

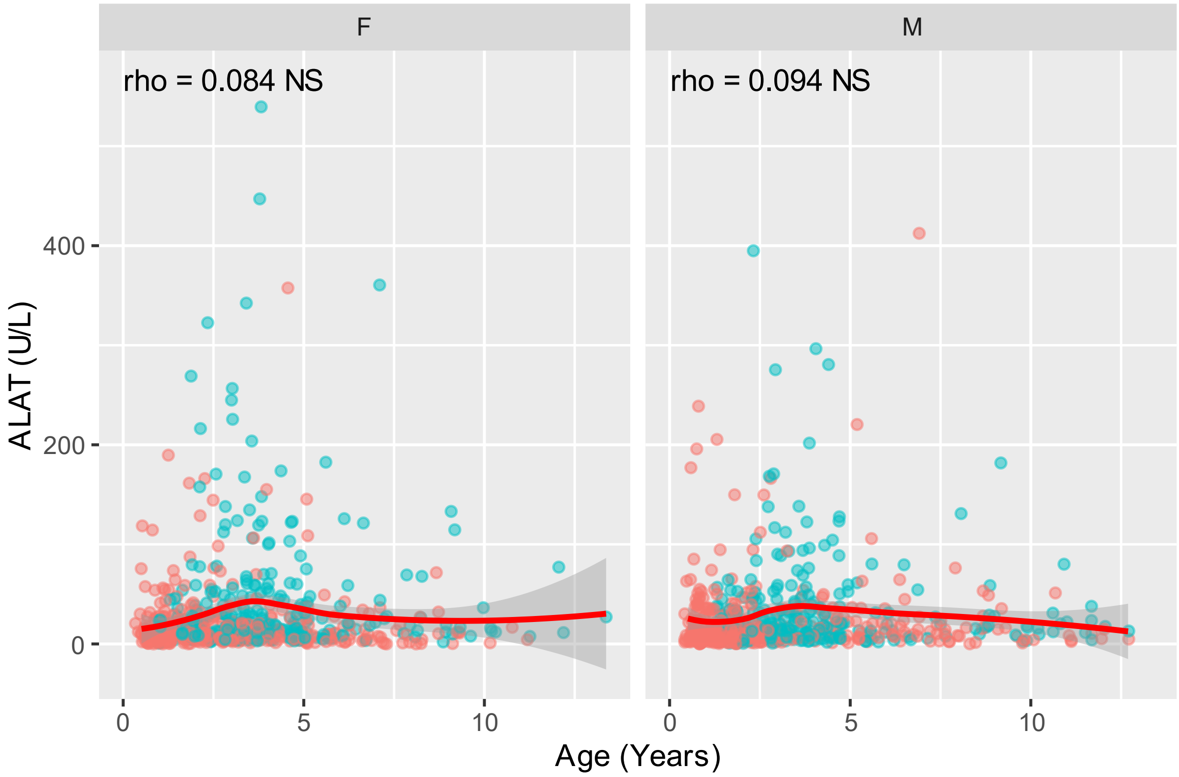


Figure S2 5. ASAT levels according to sex and age in common marmosets. Points represent individual animals (red = outdoor access, cyan = indoor only). The red line indicates a Loess moving average and the grey area indicates the 95% confidence interval. Rho is Spearman's rank correlation for parameter and Age; NS = not significant, asterisks indicate statistical significance levels * < 0.001, ** < 0.0001, *** < 0.00001, **** < 0.000001.

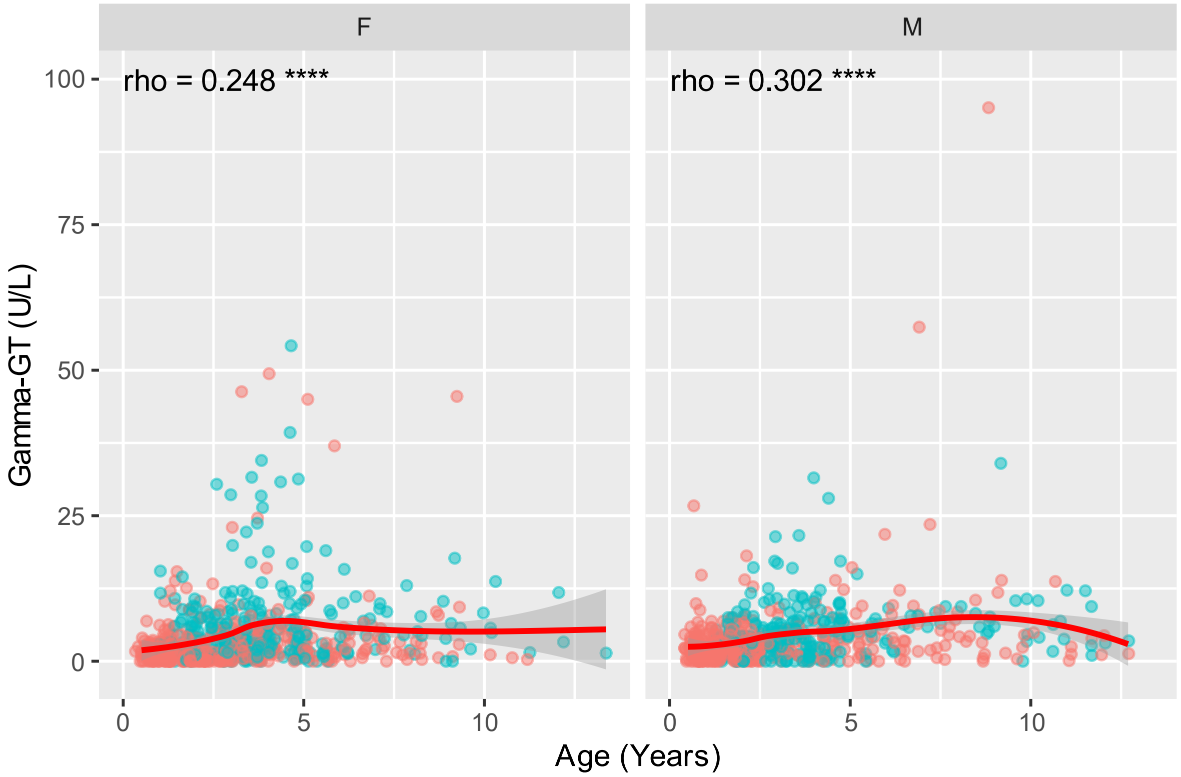


Figure S2 6. Gamma-GT levels according to sex and age in common marmosets. Points represent individual animals (red = outdoor access, cyan = indoor only). The red line indicates a Loess moving average and the grey area indicates the 95% confidence interval. Rho is Spearman's rank correlation for parameter and Age; NS = not significant, asterisks indicate statistical significance levels * < 0.001, ** < 0.0001, *** < 0.00001, **** < 0.000001.

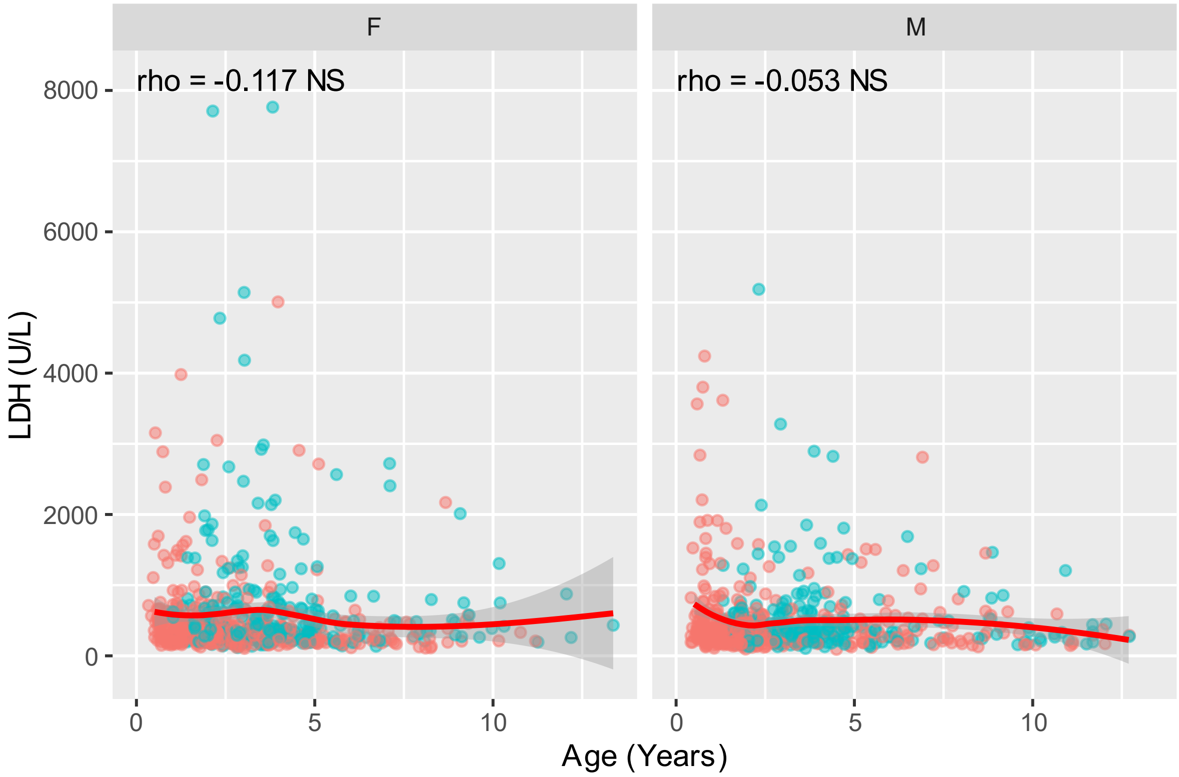


Figure S2 7. LDH levels according to sex and age in common marmosets. Points represent individual animals (red = outdoor access, cyan = indoor only). The red line indicates a Loess moving average and the grey area indicates the 95% confidence interval. Rho is Spearman's rank correlation for parameter and Age; NS = not significant, asterisks indicate statistical significance levels * < 0.001, ** < 0.0001, *** < 0.00001, **** < 0.000001.

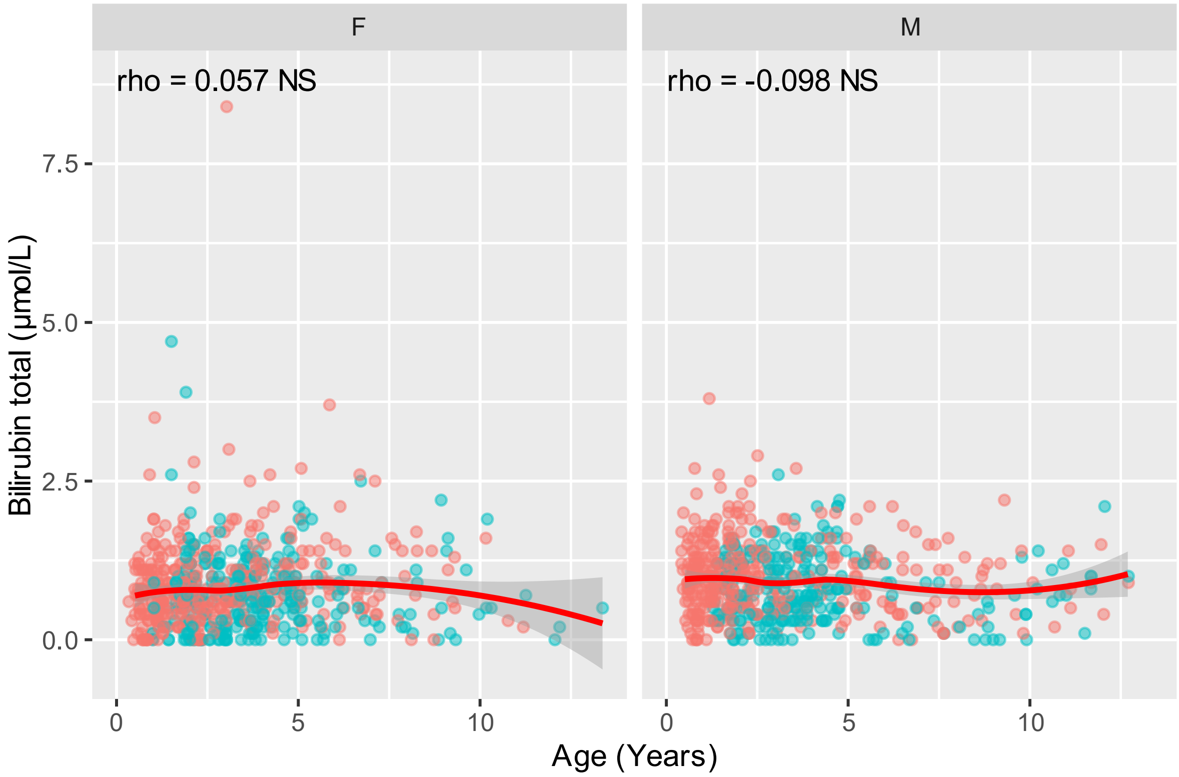


Figure S2 8. Total Bilirubin levels according to sex and age in common marmosets. Points represent individual animals (red = outdoor access, cyan = indoor only). The red line indicates a Loess moving average and the grey area indicates the 95% confidence interval. Rho is Spearman's rank correlation for parameter and Age; NS = not significant, asterisks indicate statistical significance levels * < 0.001, ** < 0.0001, *** < 0.00001, **** < 0.000001.

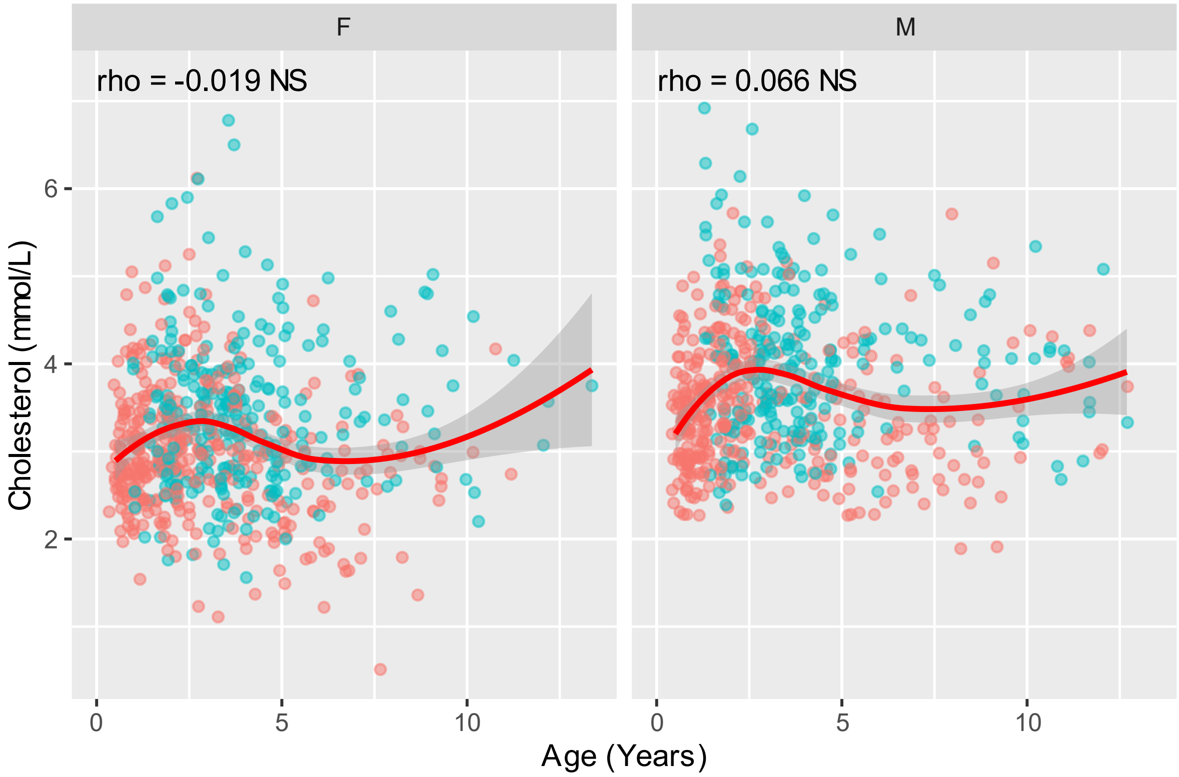


Figure S2 9. Cholesterol levels according to sex and age in common marmosets. Points represent individual animals (red = outdoor access, cyan = indoor only). The red line indicates a Loess moving average and the grey area indicates the 95% confidence interval. Rho is Spearman's rank correlation for parameter and Age; NS = not significant, asterisks indicate statistical significance levels * < 0.001, ** < 0.0001, *** < 0.00001, **** < 0.000001.

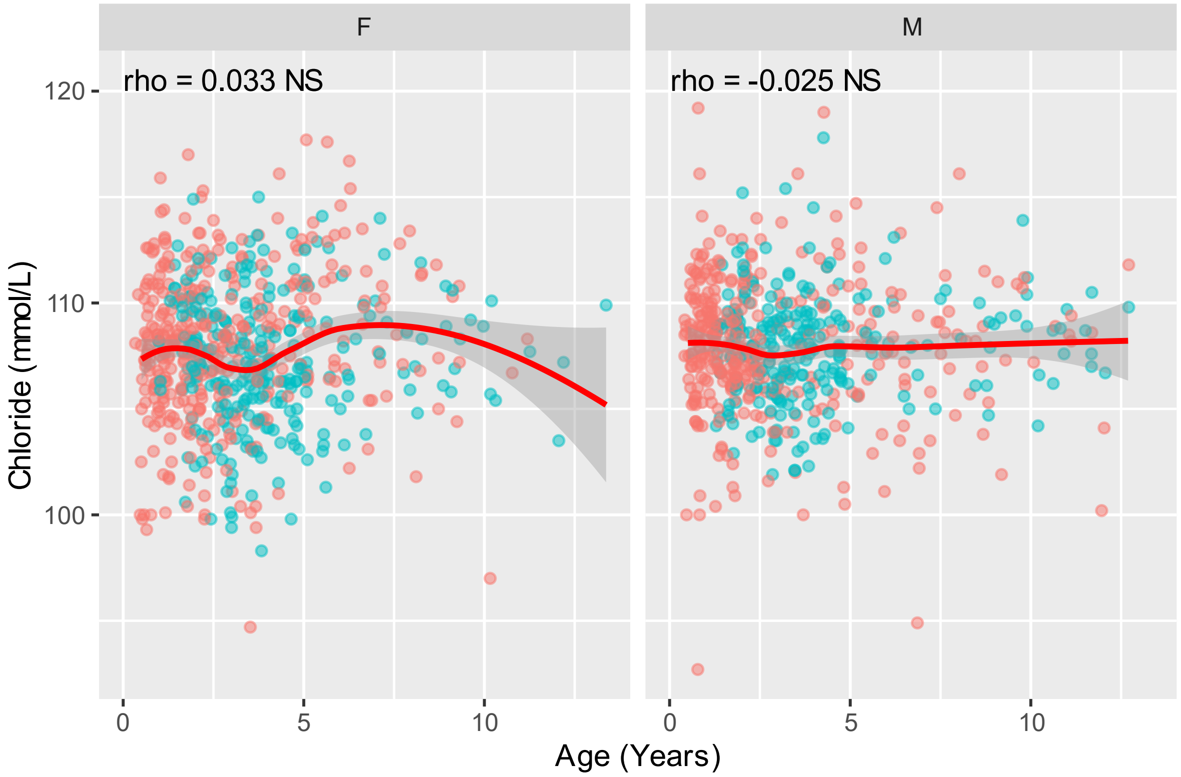


Figure S2 10. Chloride levels according to sex and age in common marmosets. Points represent individual animals (red = outdoor access, cyan = indoor only). The red line indicates a Loess moving average and the grey area indicates the 95% confidence interval. Rho is Spearman's rank correlation for parameter and Age; NS = not significant, asterisks indicate statistical significance levels * < 0.001, ** < 0.0001, *** < 0.00001, **** < 0.000001.

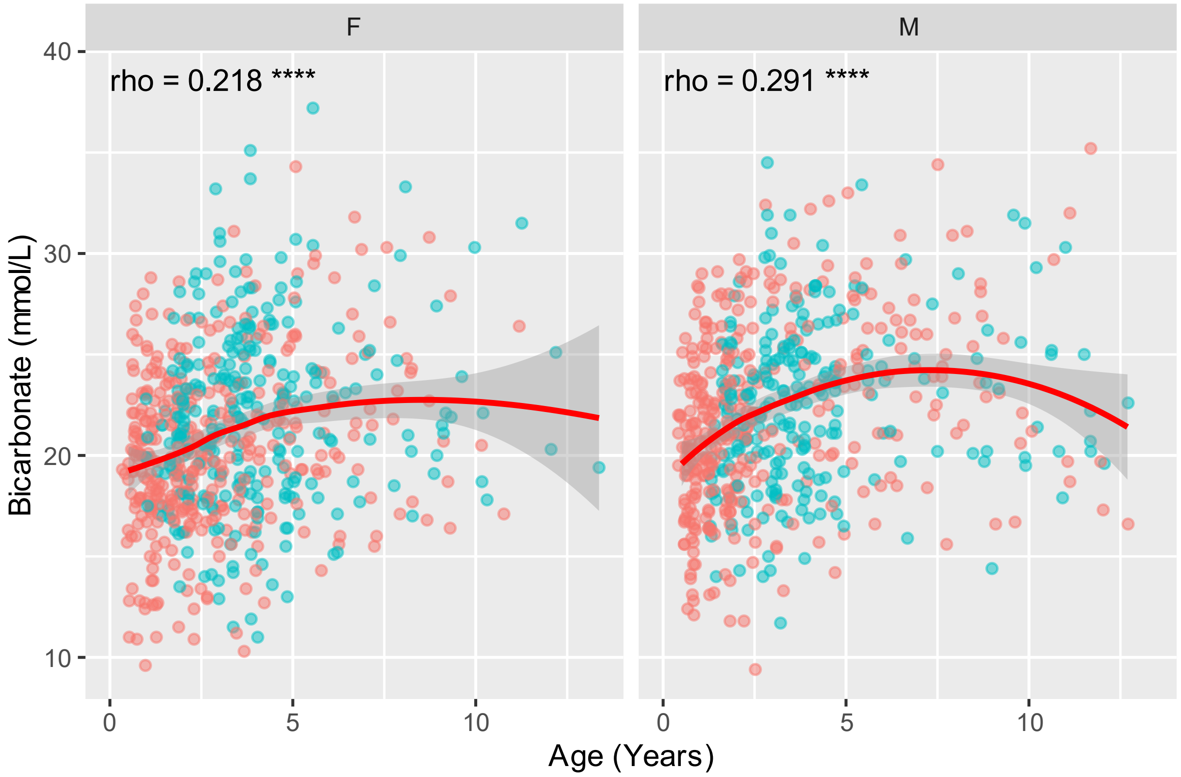


Figure S2 11. Bicarbonate levels according to sex and age in common marmosets. Points represent individual animals (red = outdoor access, cyan = indoor only). The red line indicates a Loess moving average and the grey area indicates the 95% confidence interval. Rho is Spearman’s rank correlation for parameter and Age; NS = not significant, asterisks indicate statistical significance levels * < 0.001, ** < 0.0001, *** < 0.00001, **** < 0.000001.

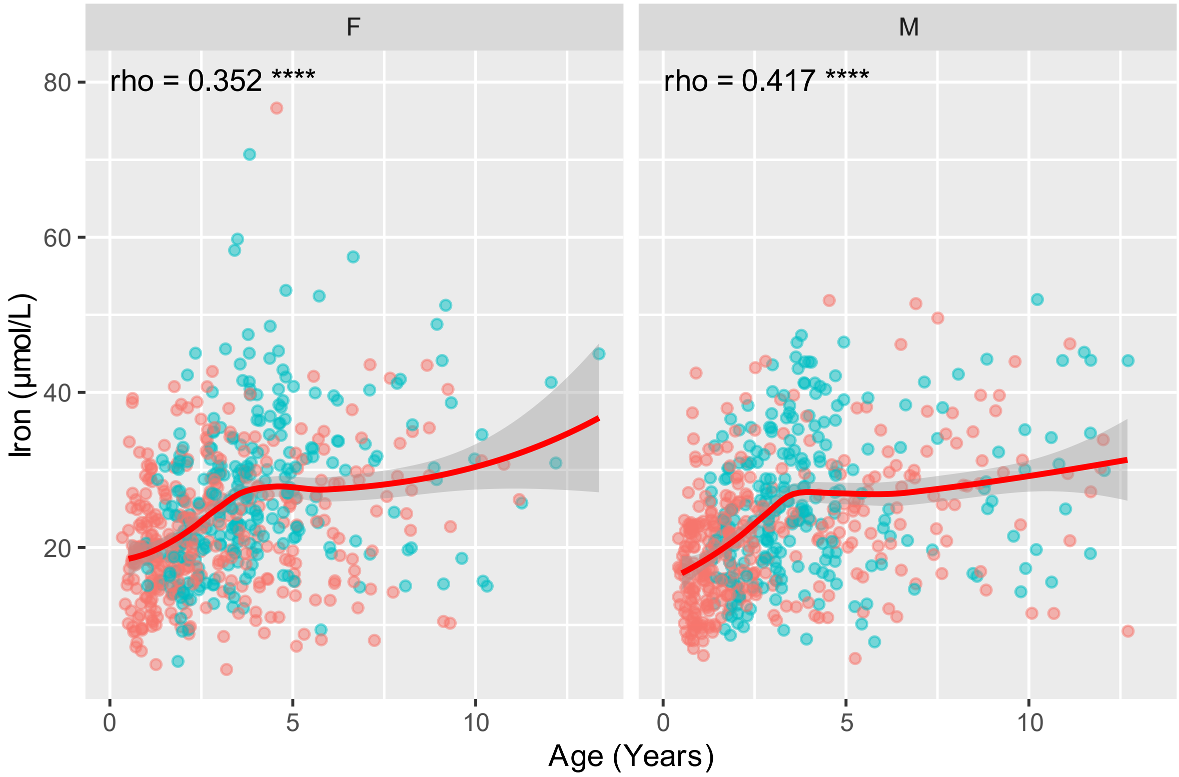


Figure S2 12. Iron levels according to sex and age in common marmosets. Points represent individual animals (red = outdoor access, cyan = indoor only). The red line indicates a Loess moving average and the grey area indicates the 95% confidence interval. Rho is Spearman's rank correlation for parameter and Age; NS = not significant, asterisks indicate statistical significance levels * < 0.001, ** < 0.0001, *** < 0.00001, **** < 0.000001.

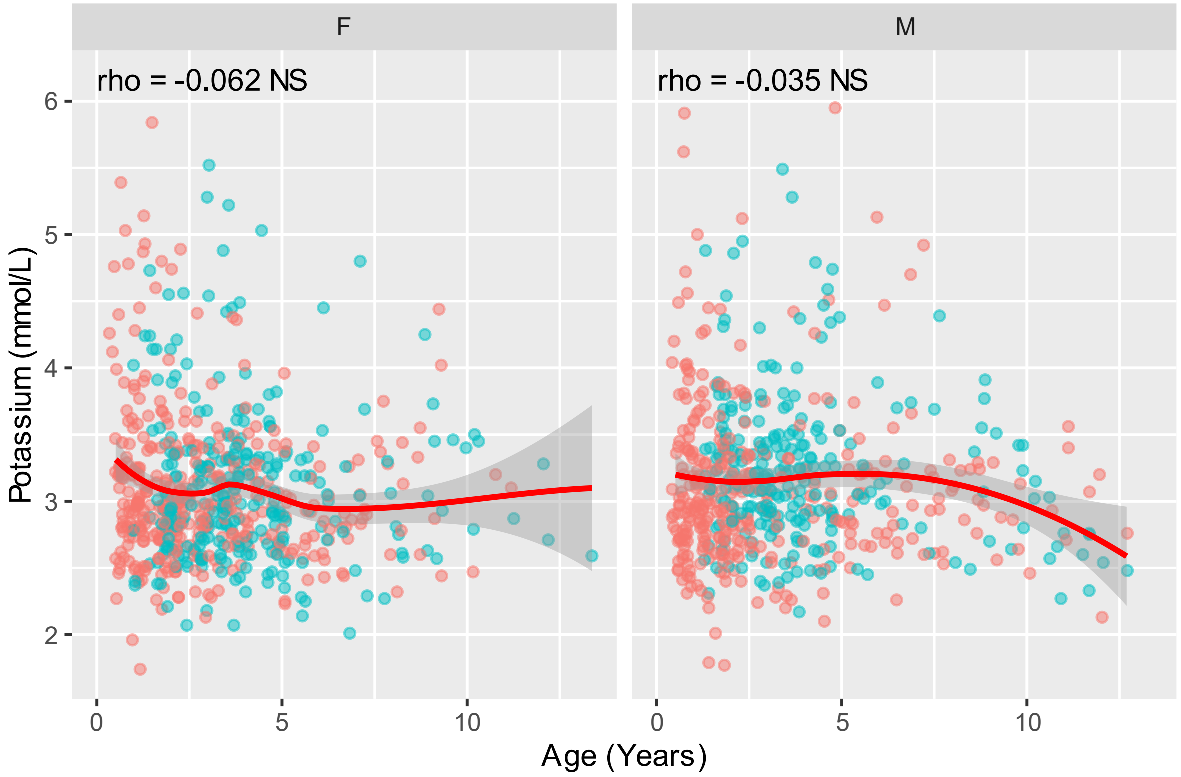


Figure S2 13. Potassium levels according to sex and age in common marmosets. Points represent individual animals (red = outdoor access, cyan = indoor only). The red line indicates a Loess moving average and the grey area indicates the 95% confidence interval. Rho is Spearman's rank correlation for parameter and Age; NS = not significant, asterisks indicate statistical significance levels * < 0.001, ** < 0.0001, *** < 0.00001, **** < 0.000001.

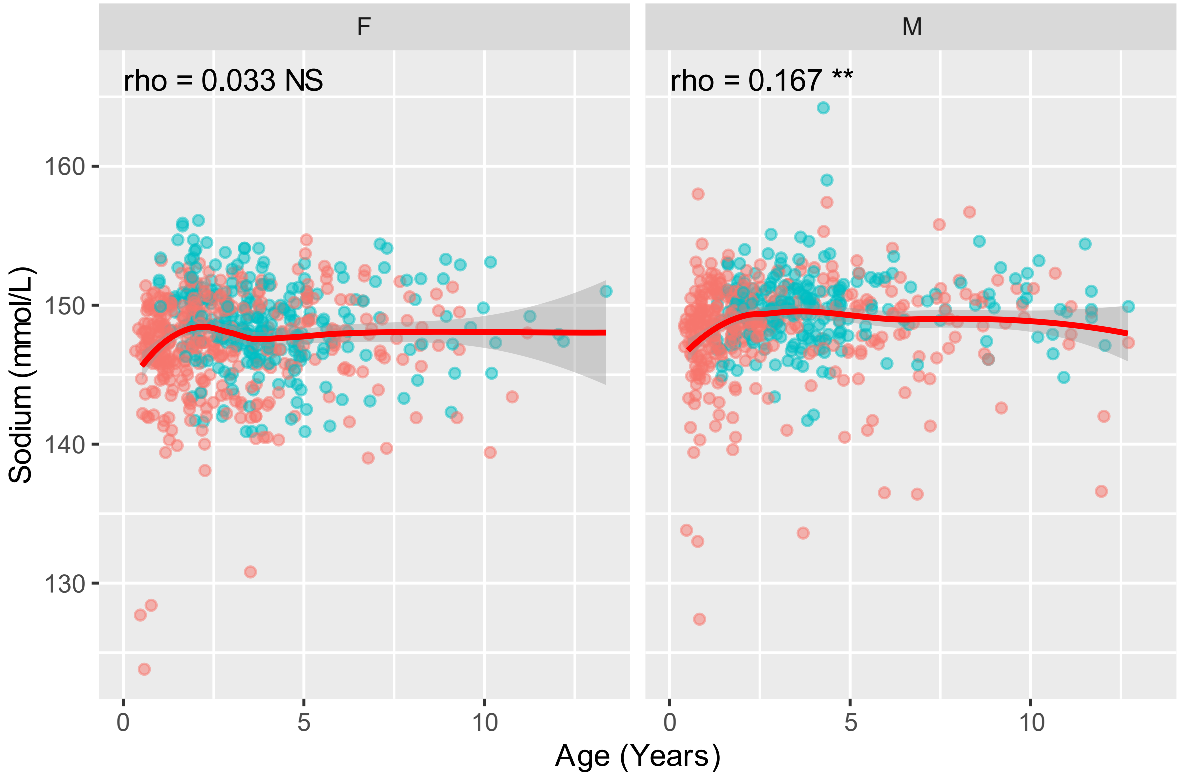


Figure S2 14. Sodium levels according to sex and age in common marmosets. Points represent individual animals (red = outdoor access, cyan = indoor only). The red line indicates a Loess moving average and the grey area indicates the 95% confidence interval. Rho is Spearman's rank correlation for parameter and Age; NS = not significant, asterisks indicate statistical significance levels * < 0.001, ** < 0.0001, *** < 0.00001, **** < 0.000001.

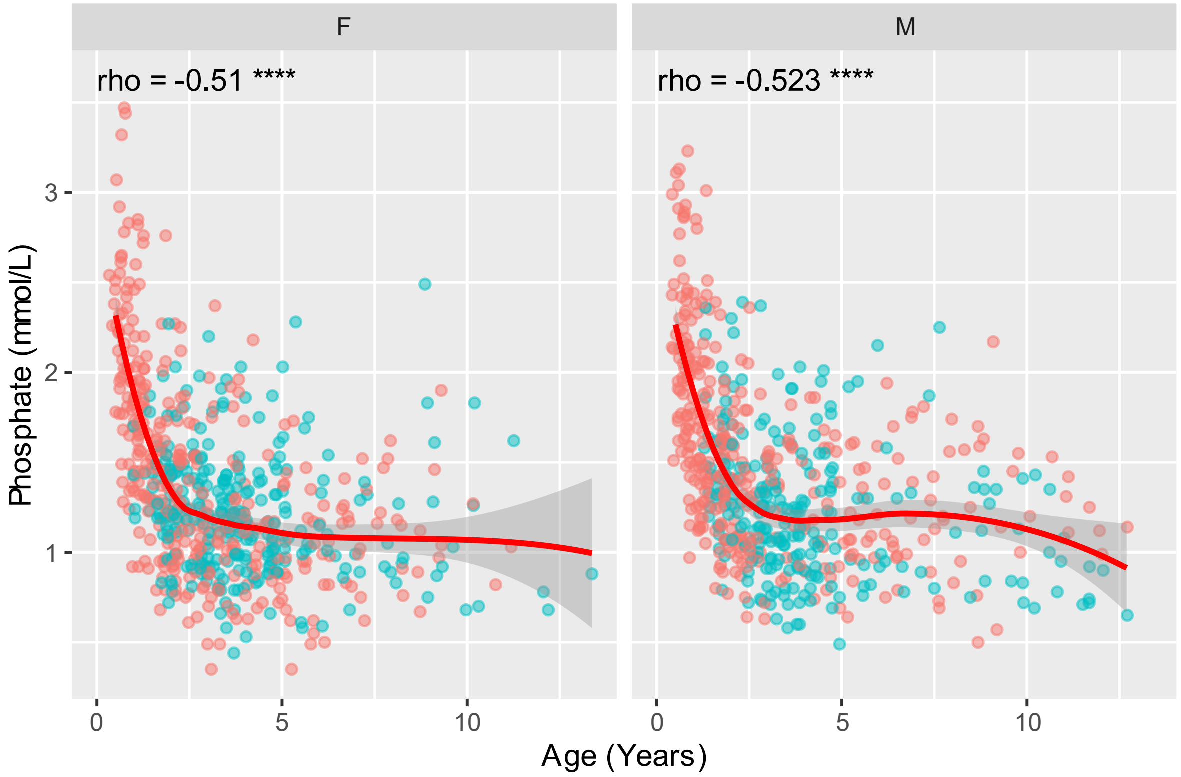


Figure S2 15. Phosphate levels according to sex and age in common marmosets. Points represent individual animals (red = outdoor access, cyan = indoor only). The red line indicates a Loess moving average and the grey area indicates the 95% confidence interval. Rho is Spearman's rank correlation for parameter and Age; NS = not significant, asterisks indicate statistical significance levels * < 0.001, ** < 0.0001, *** < 0.00001, **** < 0.000001.

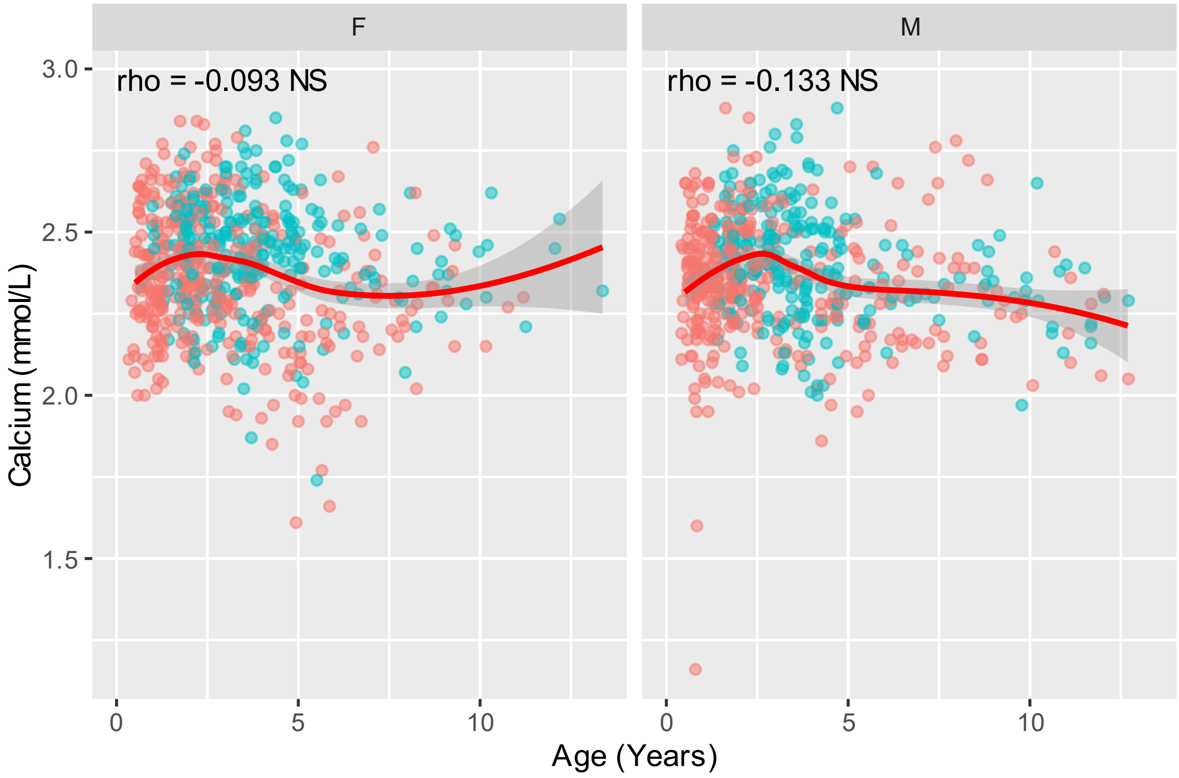


Figure S2 16. Calcium levels according to sex and age in common marmosets. Points represent individual animals (red = outdoor access, cyan = indoor only). The red line indicates a Loess moving average and the grey area indicates the 95% confidence interval. Rho is Spearman's rank correlation for parameter and Age; NS = not significant, asterisks indicate statistical significance levels * < 0.001, ** < 0.0001, *** < 0.00001, **** < 0.000001.

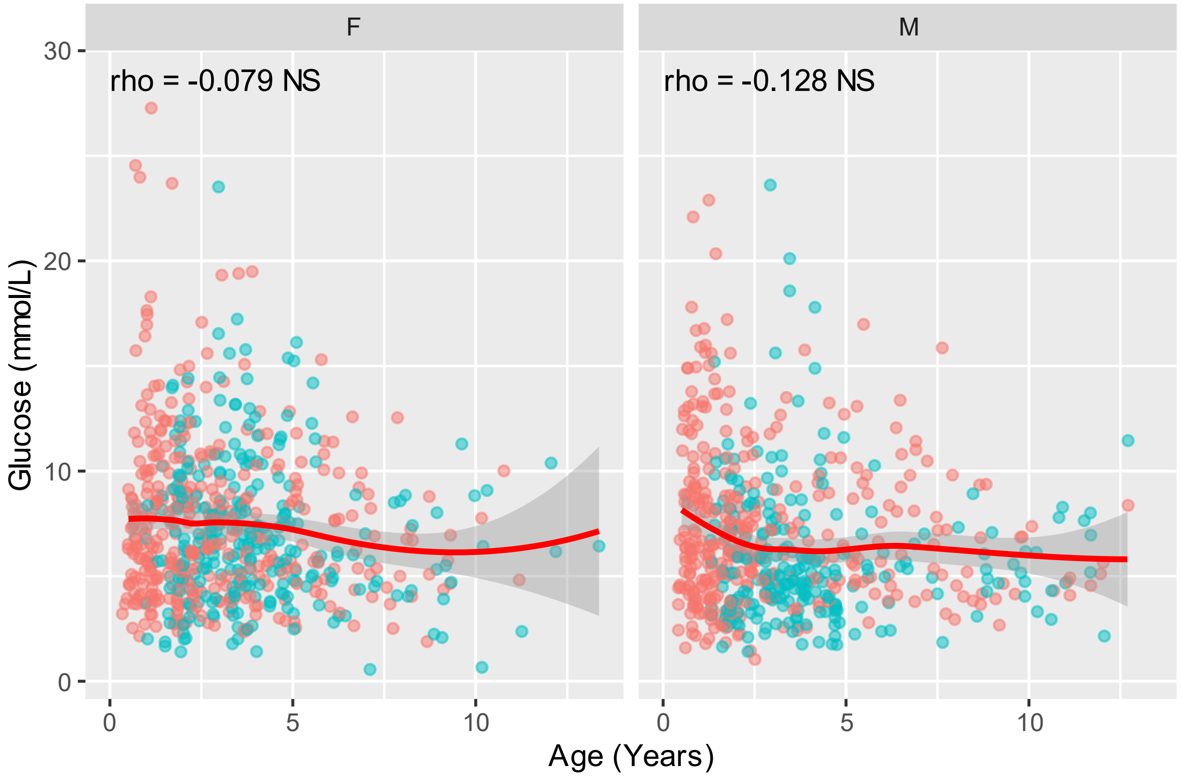


Figure S2 17. Glucose levels according to sex and age in common marmosets. Points represent individual animals (red = outdoor access, cyan = indoor only). The red line indicates a Loess moving average and the grey area indicates the 95% confidence interval. Rho is Spearman’s rank correlation for parameter and Age; NS = not significant, asterisks indicate statistical significance levels * < 0.001, ** < 0.0001, *** < 0.00001, **** < 0.000001.

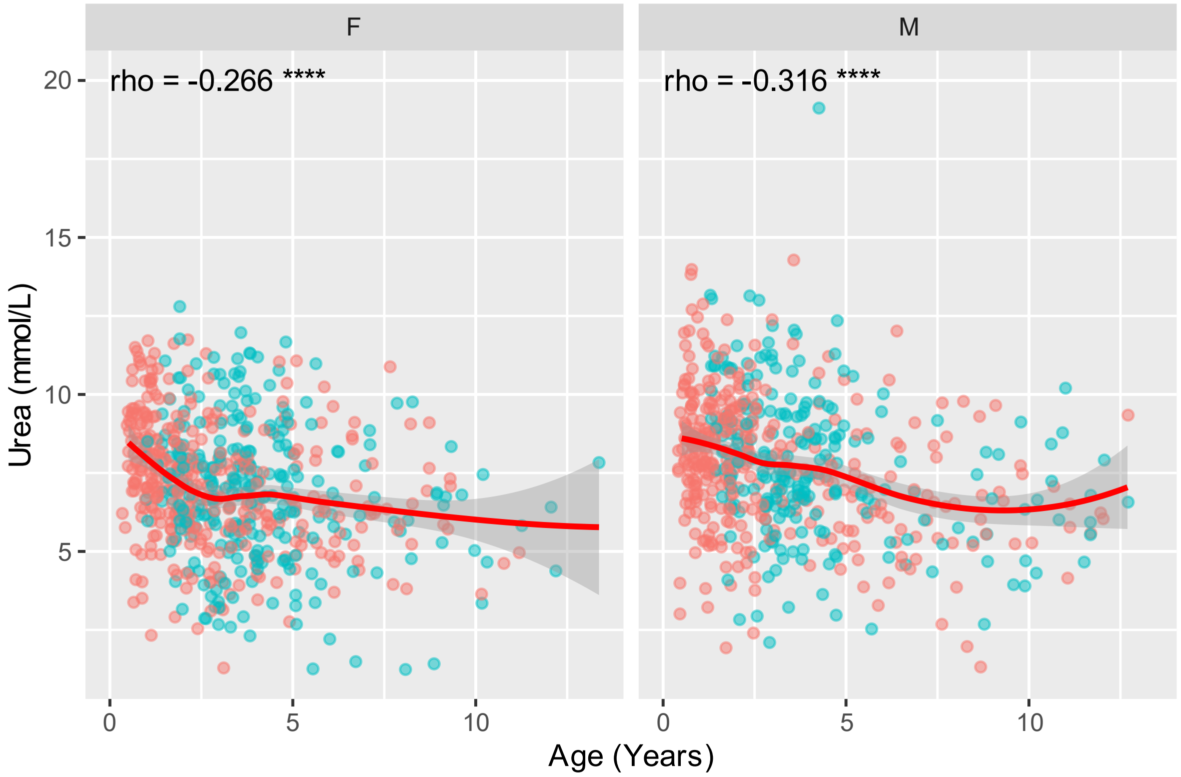


Figure S2 18. Urea levels according to sex and age in common marmosets. Points represent individual animals (red = outdoor access, cyan = indoor only). The red line indicates a Loess moving average and the grey area indicates the 95% confidence interval. Rho is Spearman's rank correlation for parameter and Age; NS = not significant, asterisks indicate statistical significance levels * < 0.001, ** < 0.0001, *** < 0.00001, **** < 0.000001.

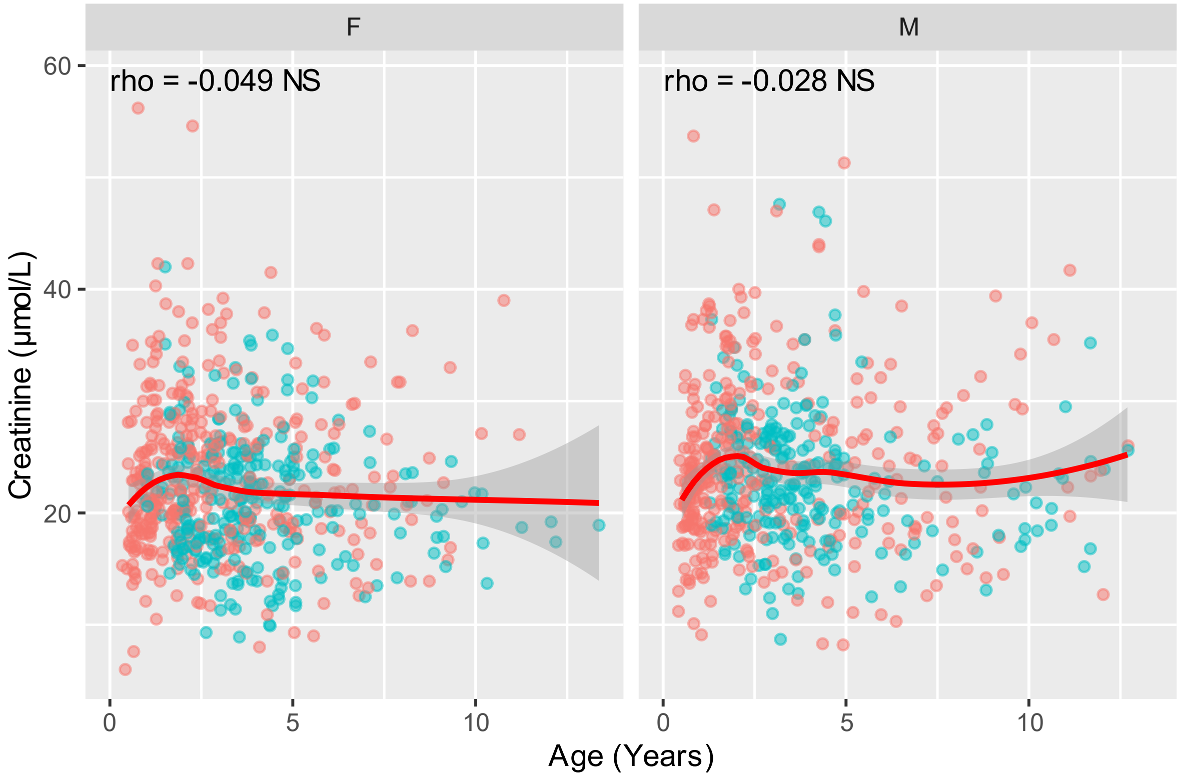


Figure S2 19. Creatinine levels according to sex and age in common marmosets. Points represent individual animals (red = outdoor access, cyan = indoor only). The red line indicates a Loess moving average and the grey area indicates the 95% confidence interval. Rho is Spearman's rank correlation for parameter and Age; NS = not significant, asterisks indicate statistical significance levels * < 0.001, ** < 0.0001, *** < 0.00001, **** < 0.000001.

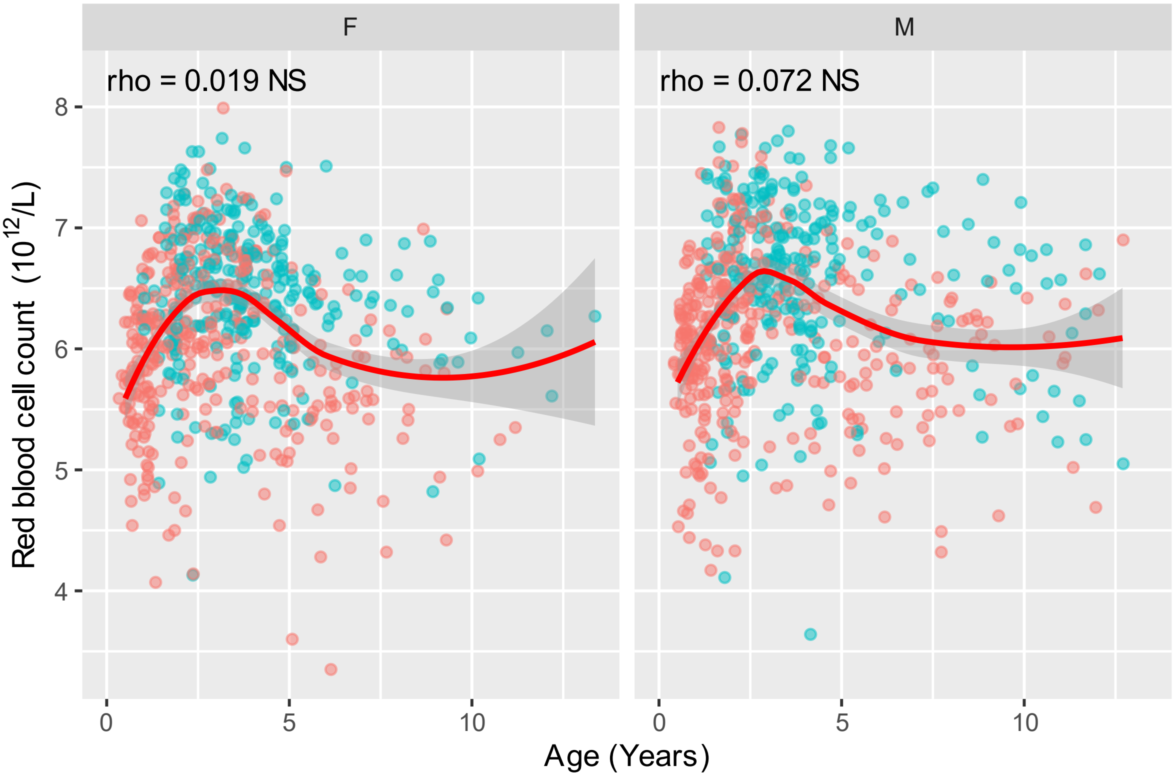


Figure S2 20. Red blood cell count according to sex and age in common marmosets. Points represent individual animals (red = outdoor access, cyan = indoor only). The red line indicates a Loess moving average and the grey area indicates the 95% confidence interval. Rho is Spearman's rank correlation for parameter and Age; NS = not significant, asterisks indicate statistical significance levels * < 0.001, ** < 0.0001, *** < 0.00001, **** < 0.000001.

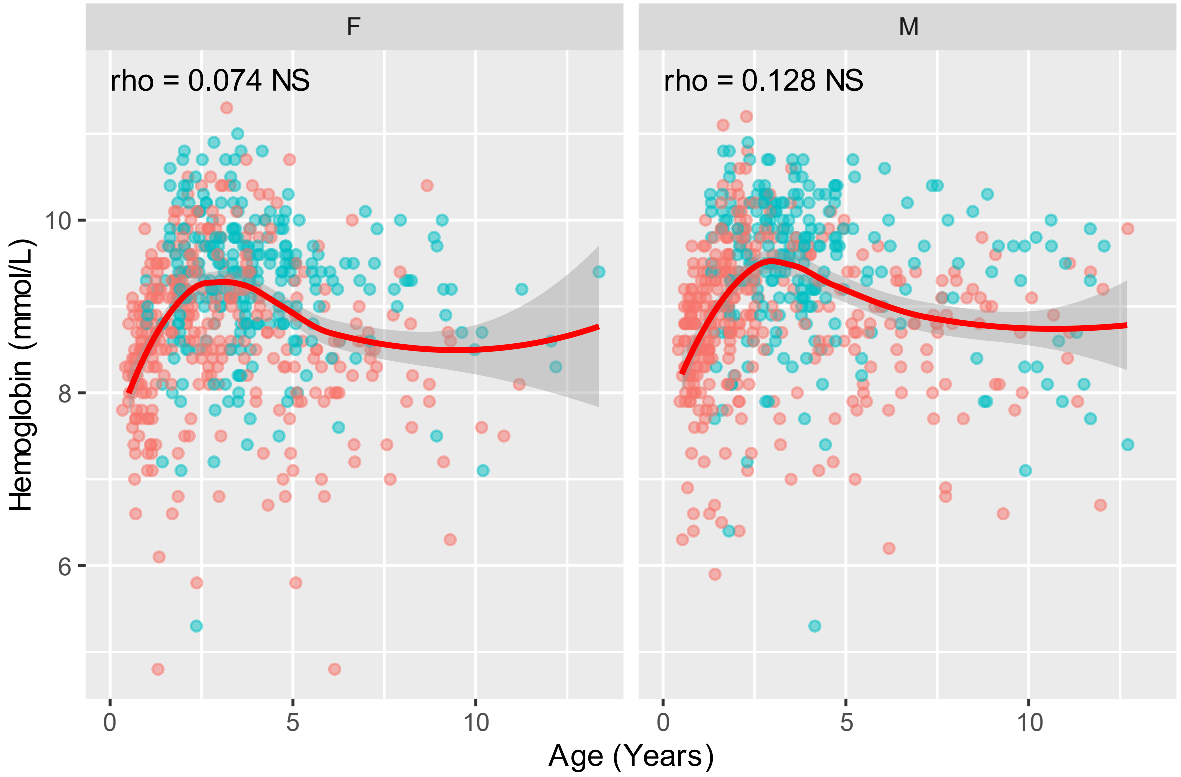


Figure S2 21. Haemoglobin levels according to sex and age in common marmosets. Points represent individual animals (red = outdoor access, cyan = indoor only). The red line indicates a Loess moving average and the grey area indicates the 95% confidence interval. Rho is Spearman's rank correlation for parameter and Age; NS = not significant, asterisks indicate statistical significance levels * < 0.001, ** < 0.0001, *** < 0.00001, **** < 0.000001.

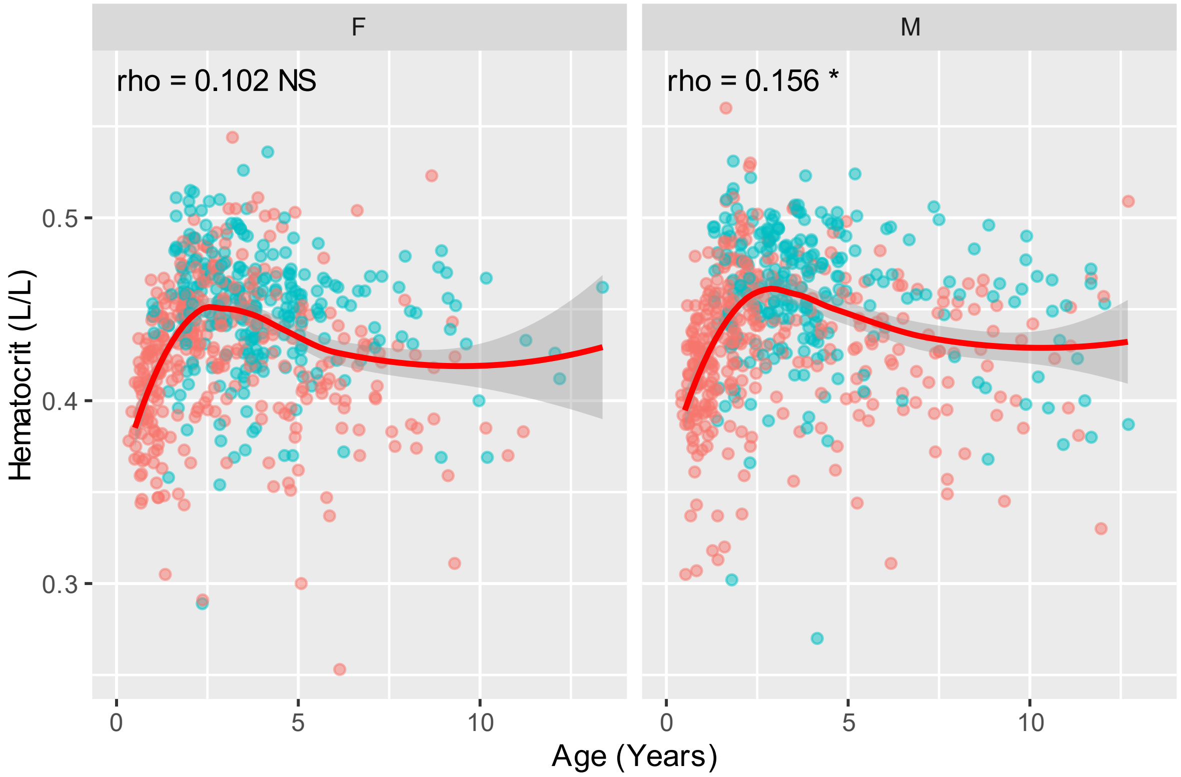


Figure S2 22. Haematocrit according to sex and age in common marmosets. Points represent individual animals (red = outdoor access, cyan = indoor only). The red line indicates a Loess moving average and the grey area indicates the 95% confidence interval. Rho is Spearman's rank correlation for parameter and Age; NS = not significant, asterisks indicate statistical significance levels * < 0.001, ** < 0.0001, *** < 0.00001, **** < 0.000001.

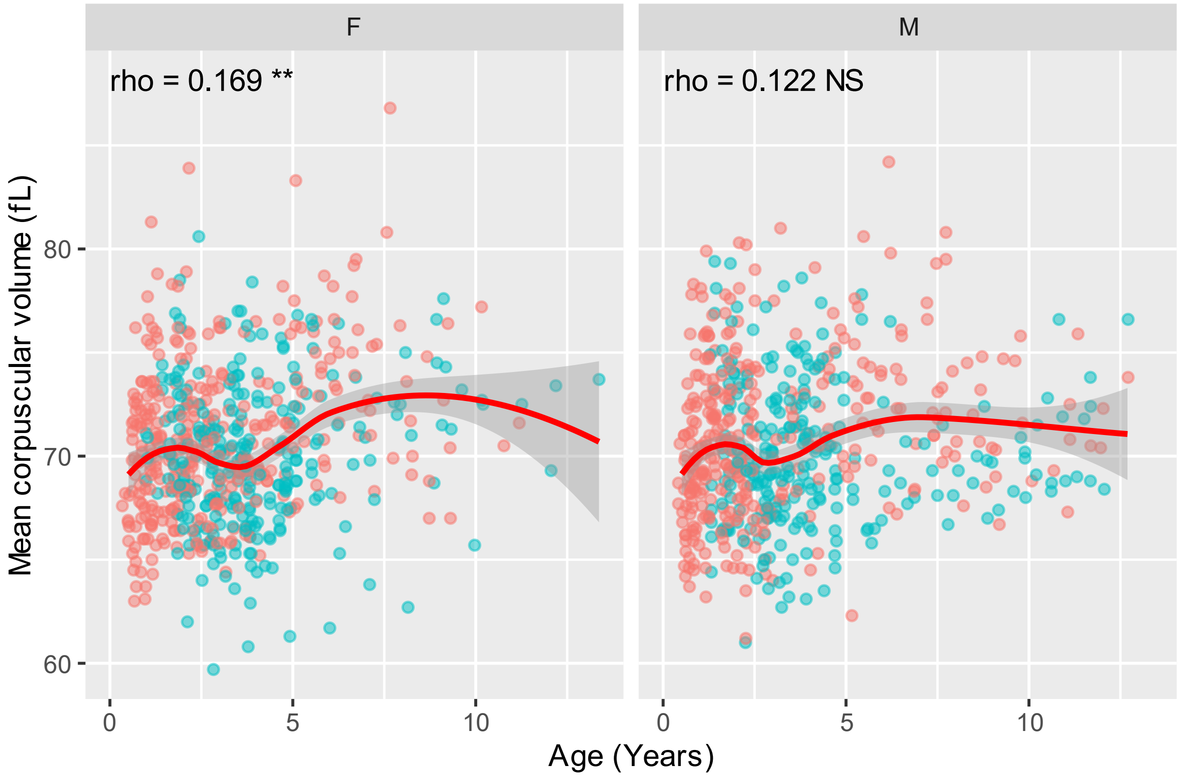


Figure S2 23. Mean Corpescular Volume according to sex and age in common marmosets. Points represent individual animals (red = outdoor access, cyan = indoor only). The red line indicates a Loess moving average and the grey area indicates the 95% confidence interval. Rho is Spearman's rank correlation for parameter and Age; NS = not significant, asterisks indicate statistical significance levels * < 0.001, ** < 0.0001, *** < 0.00001, **** < 0.000001.

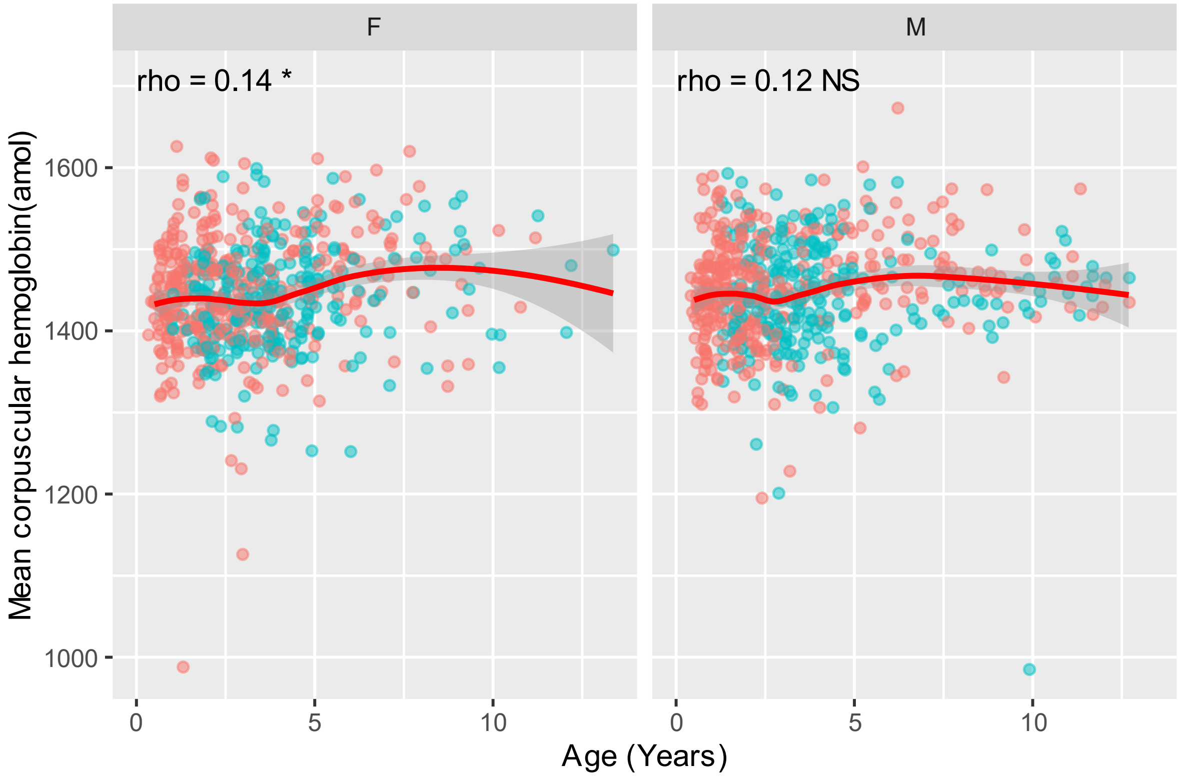


Figure S2 24. Mean Corpescular Haemoglobin according to sex and age in common marmosets. Points represent individual animals (red = outdoor access, cyan = indoor only). The red line indicates a Loess moving average and the grey area indicates the 95% confidence interval. Rho is Spearman's rank correlation for parameter and Age; NS = not significant, asterisks indicate statistical significance levels * < 0.001, ** < 0.0001, *** < 0.00001, **** < 0.000001.

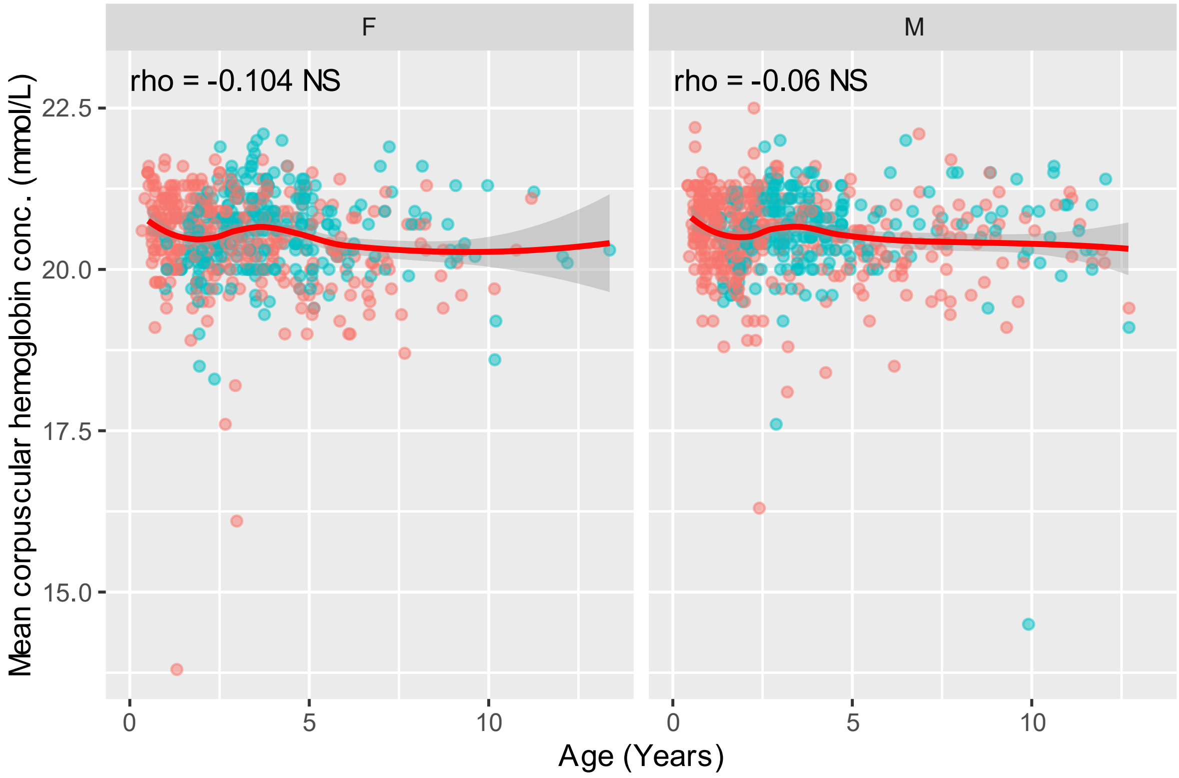


Figure S2 25. Mean Corpescular Haemoglobin Concentration according to sex and age in common marmosets. Points represent individual animals (red = outdoor access, cyan = indoor only). The red line indicates a Loess moving average and the grey area indicates the 95% confidence interval. Rho is Spearman's rank correlation for parameter and Age; NS = not significant, asterisks indicate statistical significance levels * < 0.001, ** < 0.0001, *** < 0.00001, **** < 0.000001.

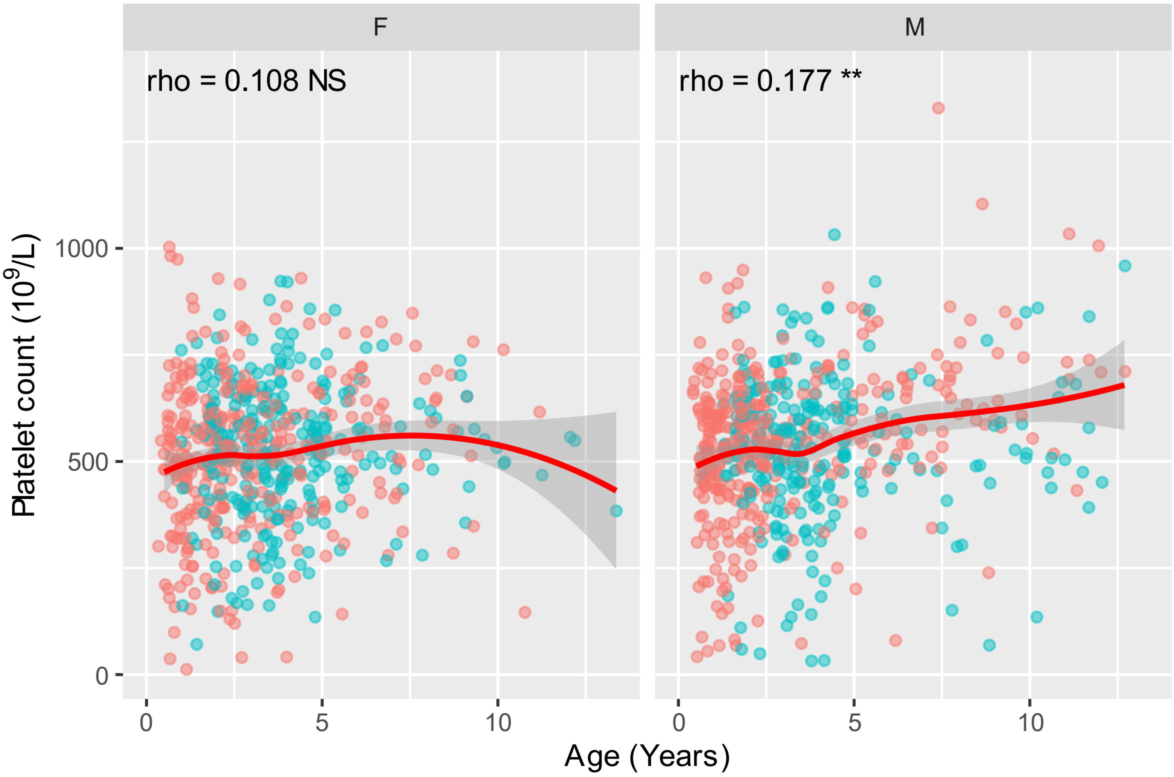


Figure S2 26. Platelet Count according to sex and age in common marmosets. Points represent individual animals (red = outdoor access, cyan = indoor only). The red line indicates a Loess moving average and the grey area indicates the 95% confidence interval. Rho is Spearman's rank correlation for parameter and Age; NS = not significant, asterisks indicate statistical significance levels * < 0.001, ** < 0.0001, *** < 0.00001, **** < 0.000001.

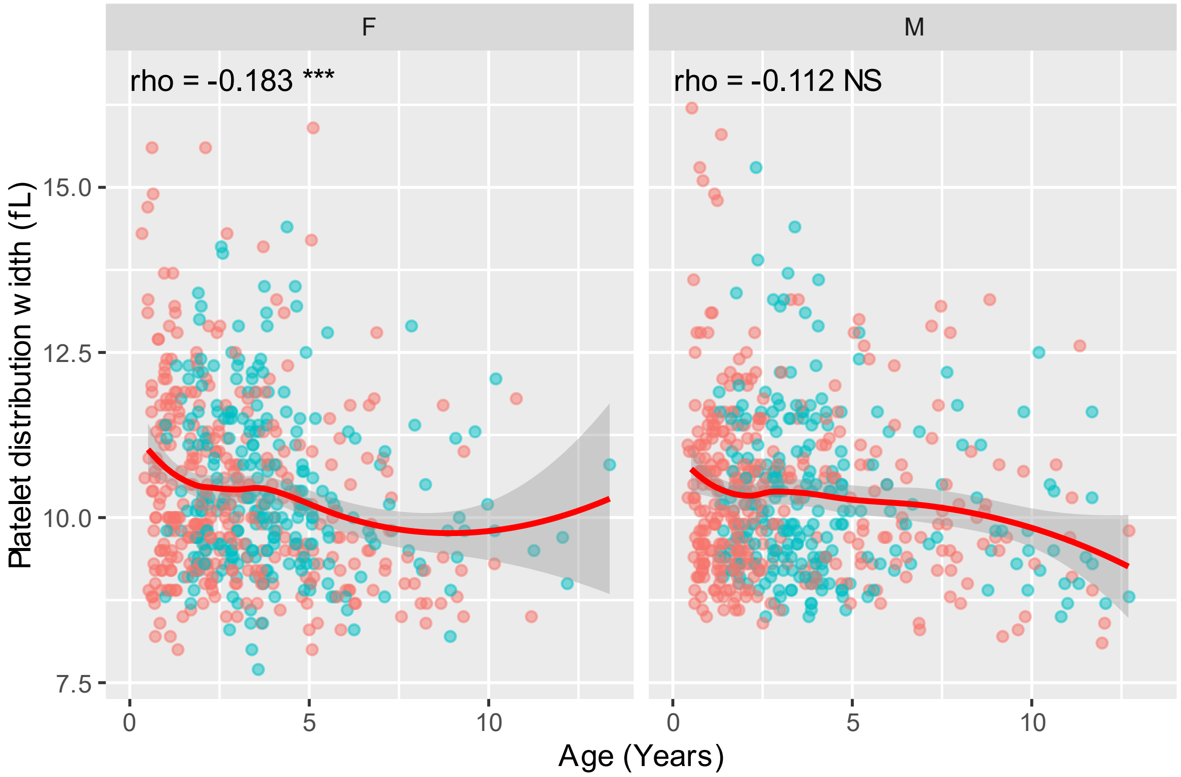


Figure S2 27. Platelet distribution width according to sex and age in common marmosets. Points represent individual animals (red = outdoor access, cyan = indoor only). The red line indicates a Loess moving average and the grey area indicates the 95% confidence interval. Rho is Spearman's rank correlation for parameter and Age; NS = not significant, asterisks indicate statistical significance levels * < 0.001, ** < 0.0001, *** < 0.00001, **** < 0.000001.

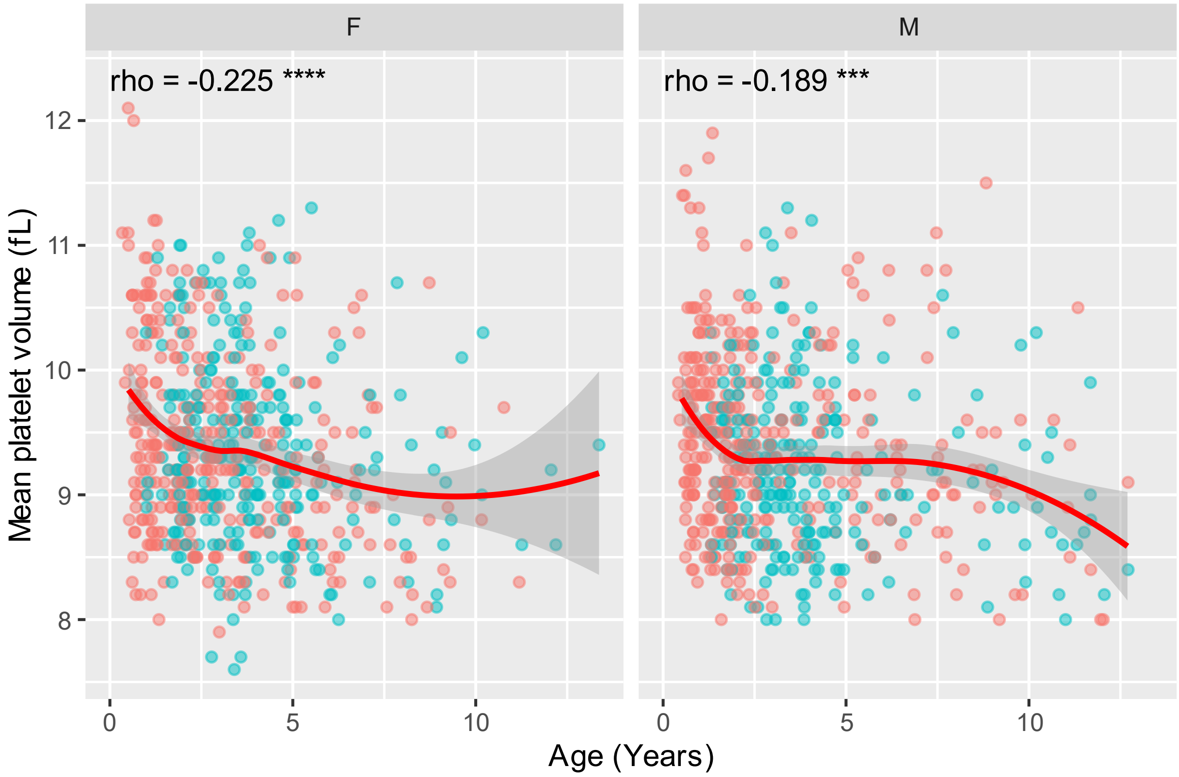


Figure S2 28. Mean Platelet Volume according to sex and age in common marmosets. Points represent individual animals (red = outdoor access, cyan = indoor only). The red line indicates a Loess moving average and the grey area indicates the 95% confidence interval. Rho is Spearman's rank correlation for parameter and Age; NS = not significant, asterisks indicate statistical significance levels * < 0.001, ** < 0.0001, *** < 0.00001, **** < 0.000001.

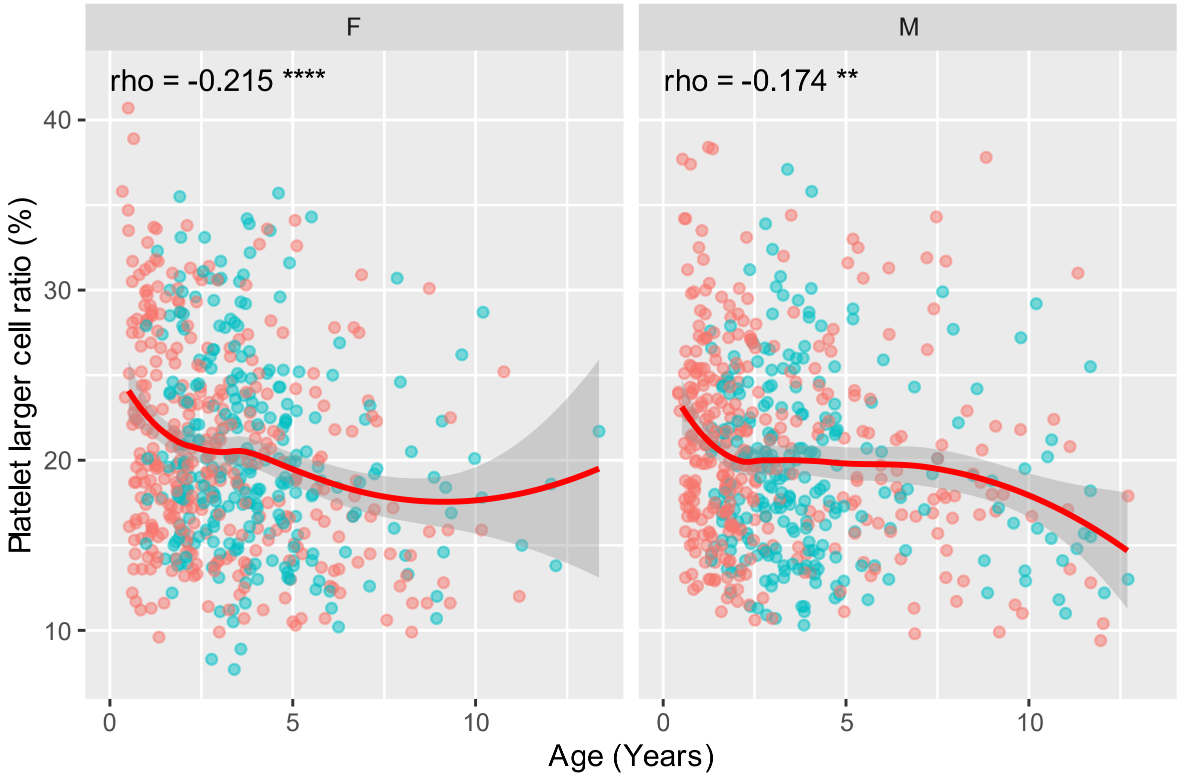


Figure S2 29. Platelet Larger Cell Ratio according to sex and age in common marmosets. Points represent individual animals (red = outdoor access, cyan = indoor only). The red line indicates a Loess moving average and the grey area indicates the 95% confidence interval. Rho is Spearman's rank correlation for parameter and Age; NS = not significant, asterisks indicate statistical significance levels * < 0.001, ** < 0.0001, *** < 0.00001, **** < 0.000001.

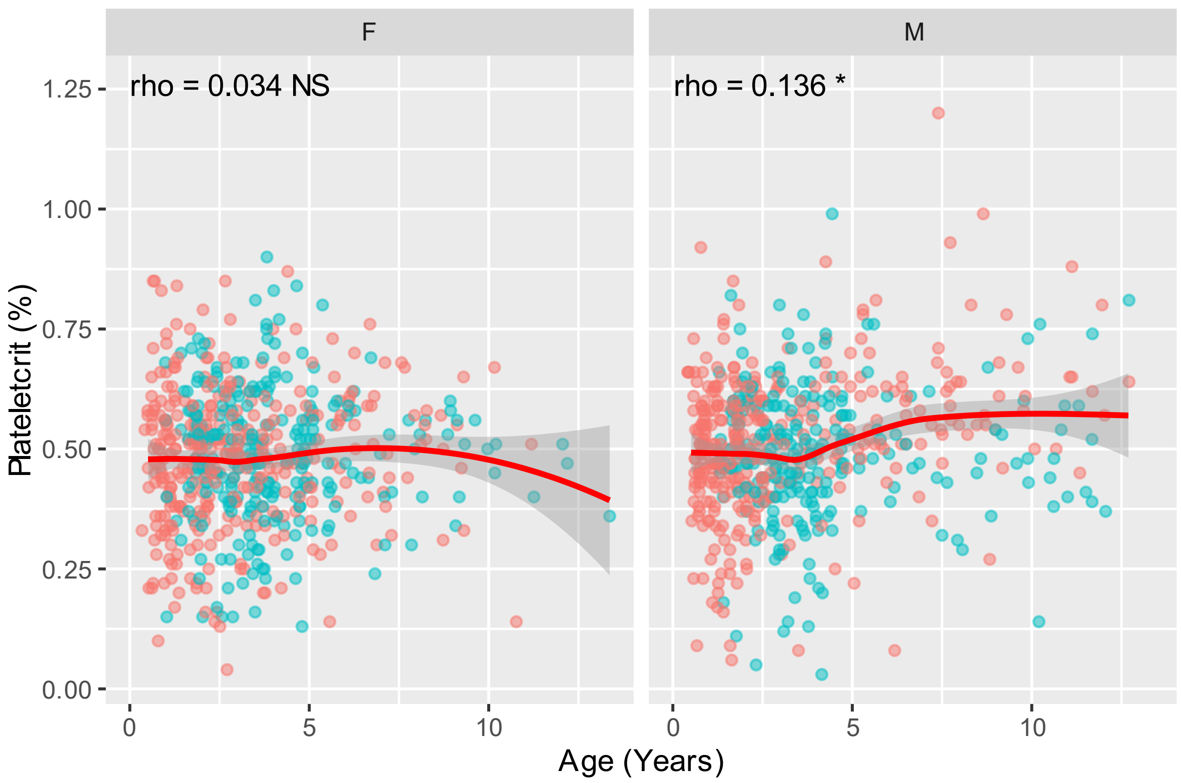


Figure S2 30. Plateletcrit according to sex and age in common marmosets. Points represent individual animals (red = outdoor access, cyan = indoor only). The red line indicates a Loess moving average and the grey area indicates the 95% confidence interval. Rho is Spearman's rank correlation for parameter and Age; NS = not significant, asterisks indicate statistical significance levels * < 0.001, ** < 0.0001, *** < 0.00001, **** < 0.000001.

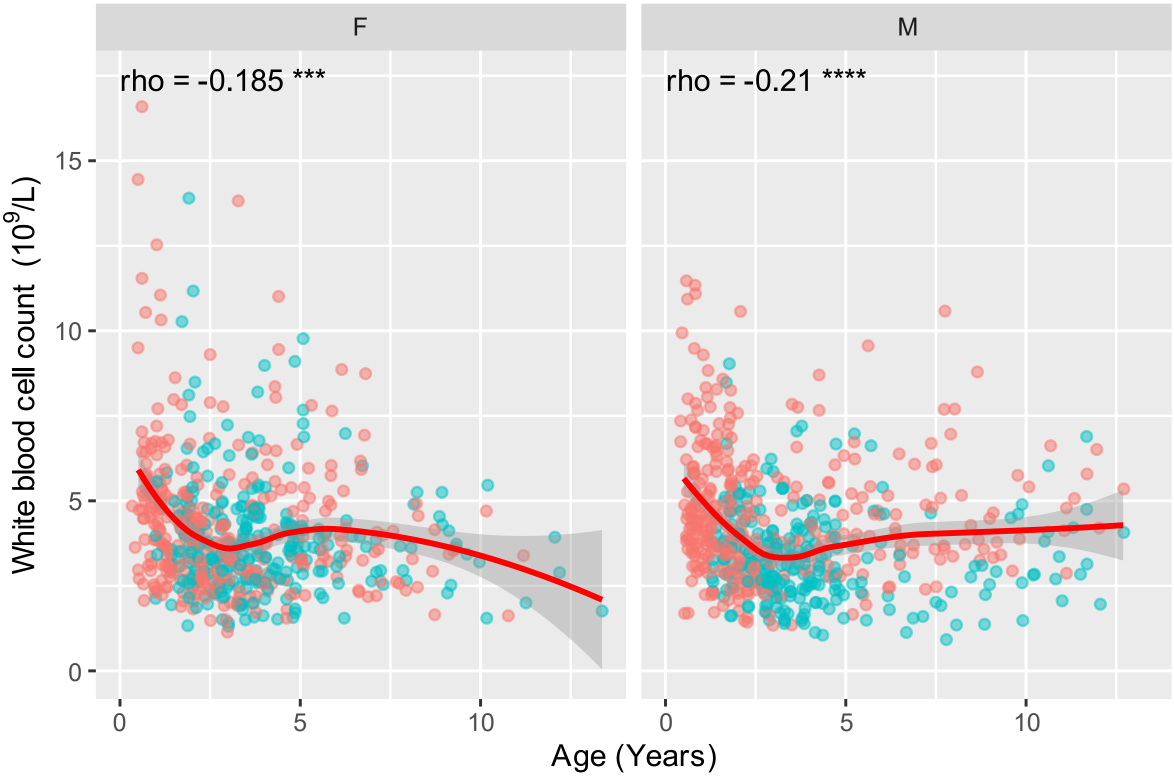


Figure S2 31. White Blood Cell count according to sex and age in common marmosets. Points represent individual animals (red = outdoor access, cyan = indoor only). The red line indicates a Loess moving average and the grey area indicates the 95% confidence interval. Rho is Spearman's rank correlation for parameter and Age; NS = not significant, asterisks indicate statistical significance levels * < 0.001, ** < 0.0001, *** < 0.00001, **** < 0.000001.

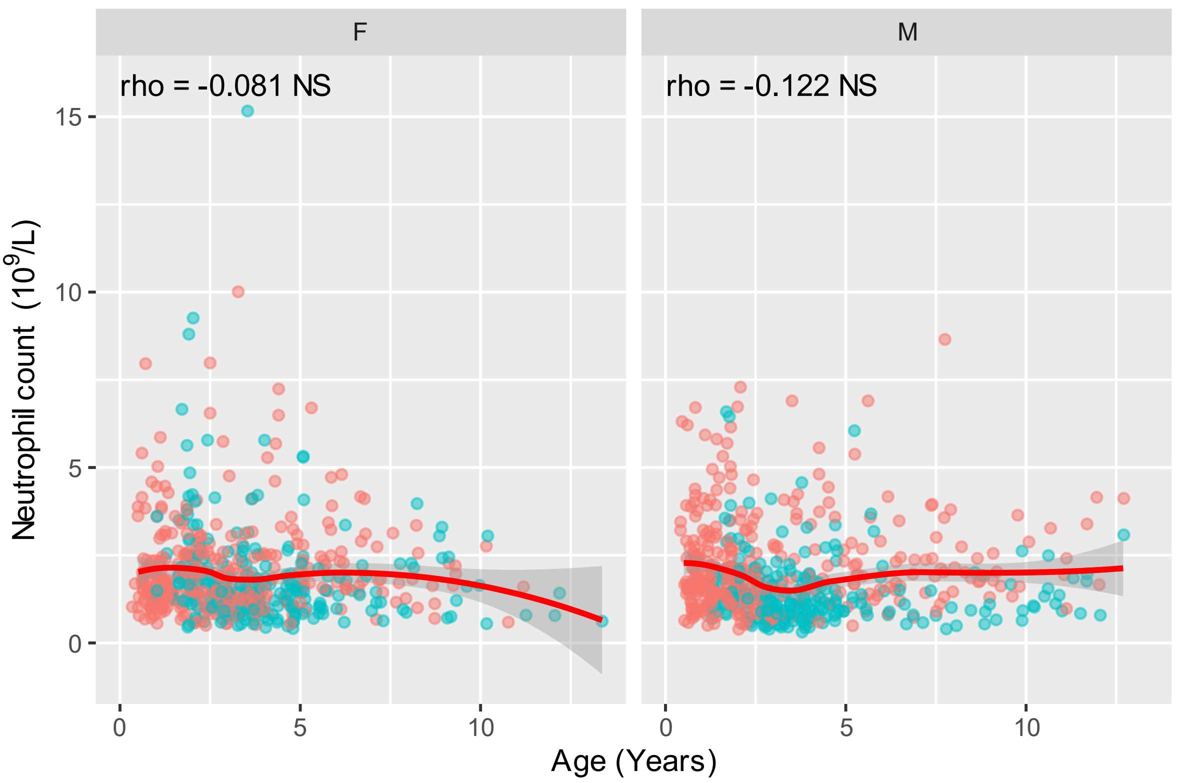


Figure S2 32. Neutrophil count according to sex and age in common marmosets. Points represent individual animals (red = outdoor access, cyan = indoor only). The red line indicates a Loess moving average and the grey area indicates the 95% confidence interval. Rho is Spearman's rank correlation for parameter and Age; NS = not significant, asterisks indicate statistical significance levels * < 0.001, ** < 0.0001, *** < 0.00001, **** < 0.000001.

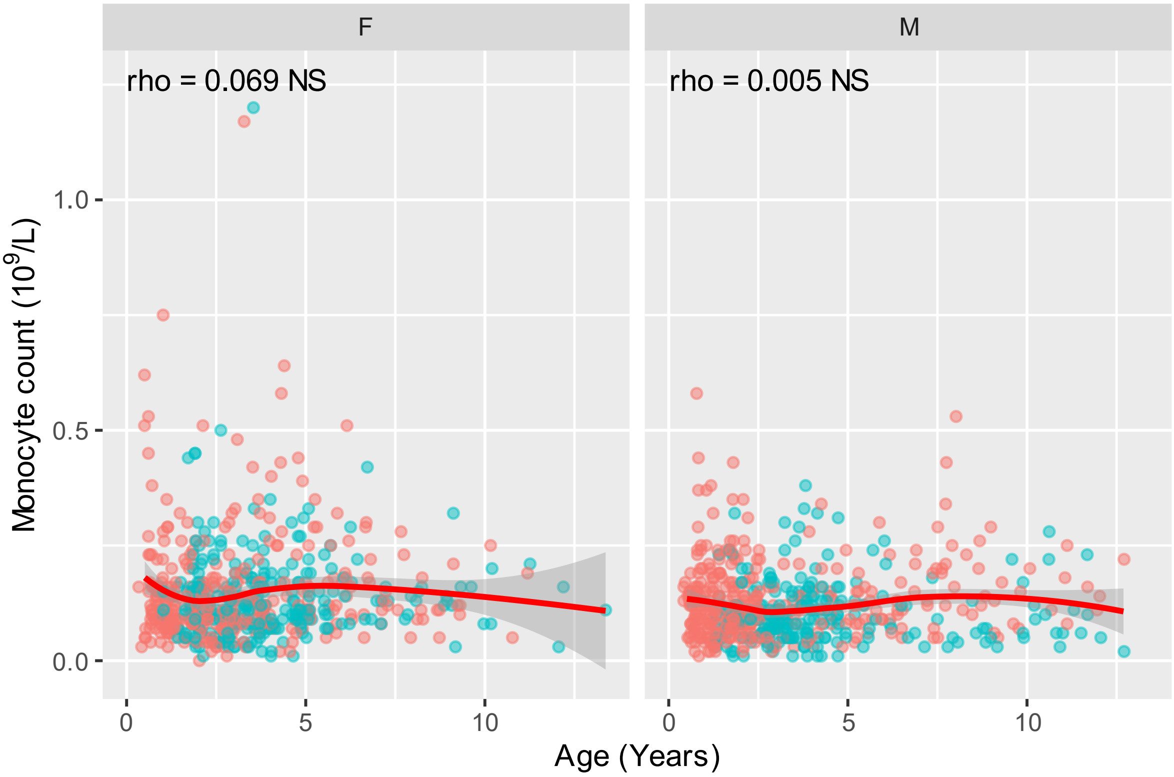


Figure S2 33. Monocyte count according to sex and age in common marmosets. Points represent individual animals (red = outdoor access, cyan = indoor only). The red line indicates a Loess moving average and the grey area indicates the 95% confidence interval. Rho is Spearman's rank correlation for parameter and Age; NS = not significant, asterisks indicate statistical significance levels * < 0.001, ** < 0.0001, *** < 0.00001, **** < 0.000001.

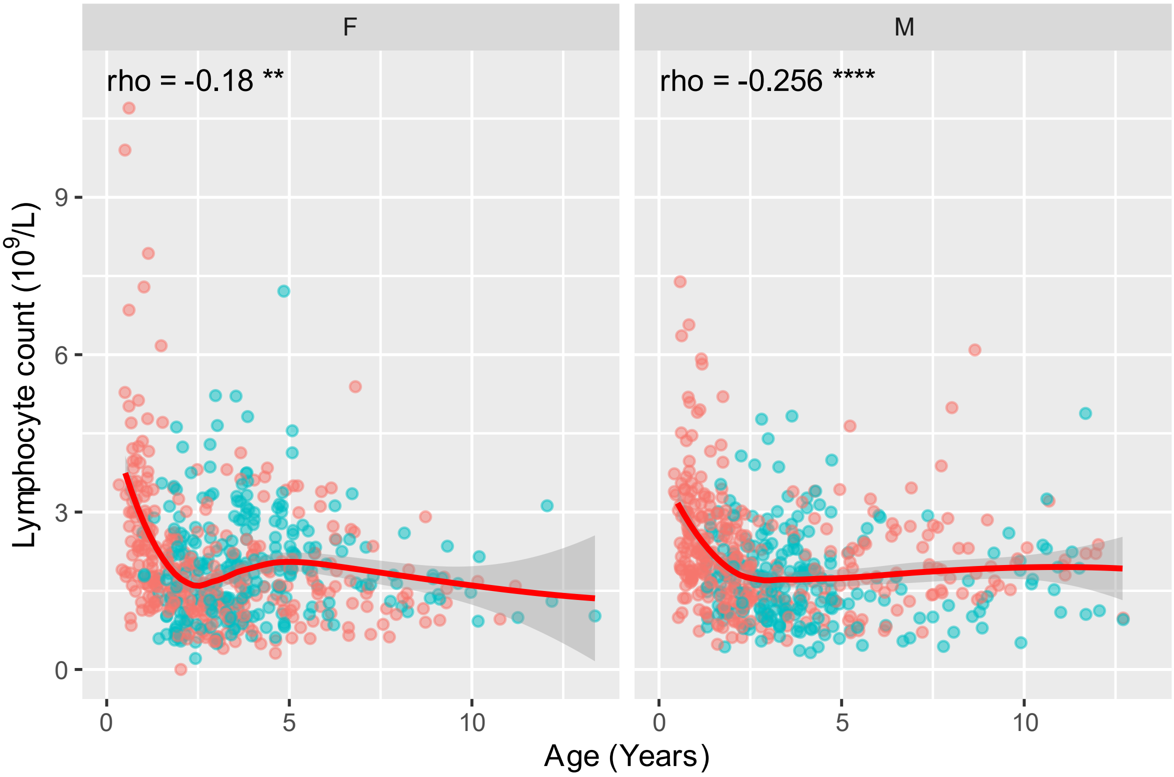


Figure S2 34. Lymphocyte count according to sex and age in common marmosets. Points represent individual animals (red = outdoor access, cyan = indoor only). The red line indicates a Loess moving average and the grey area indicates the 95% confidence interval. Rho is Spearman's rank correlation for parameter and Age; NS = not significant, asterisks indicate statistical significance levels * < 0.001, ** < 0.0001, *** < 0.00001, **** < 0.000001.

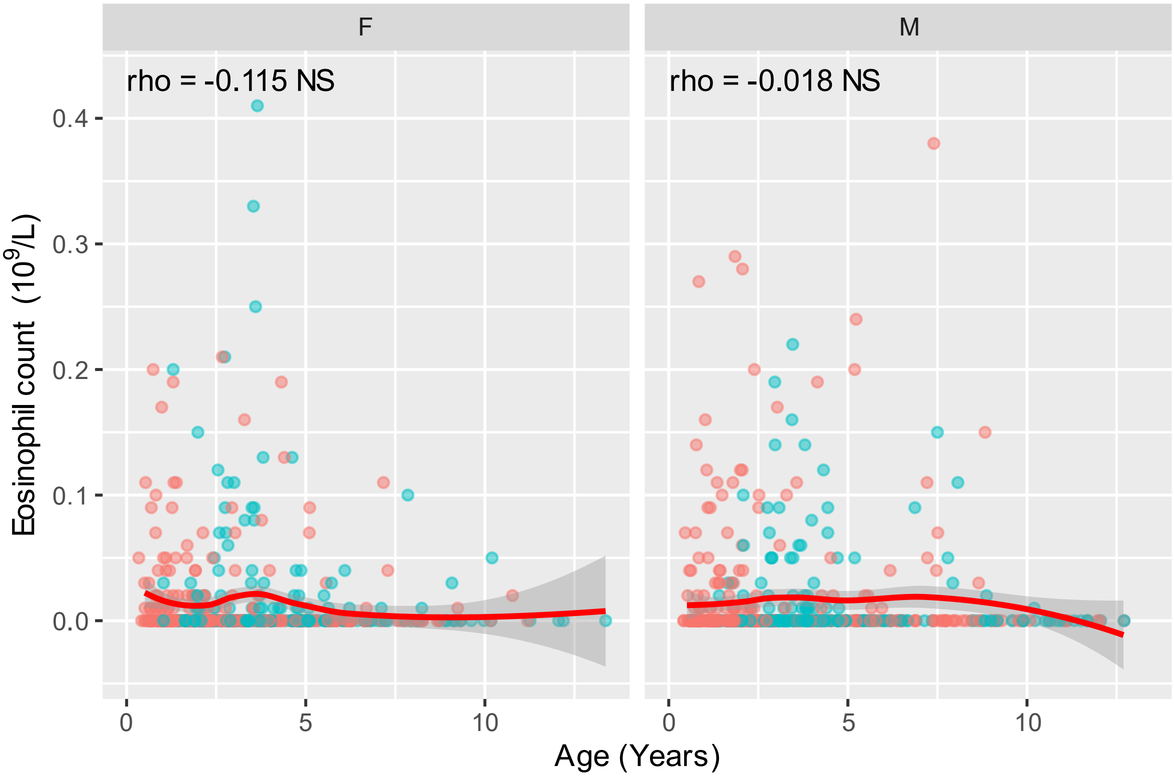


Figure S2 35. Eosinophil count according to sex and age in common marmosets. Points represent individual animals (red = outdoor access, cyan = indoor only). The red line indicates a Loess moving average and the grey area indicates the 95% confidence interval. Rho is Spearman's rank correlation for parameter and Age; NS = not significant, asterisks indicate statistical significance levels * < 0.001, ** < 0.0001, *** < 0.00001, **** < 0.000001.

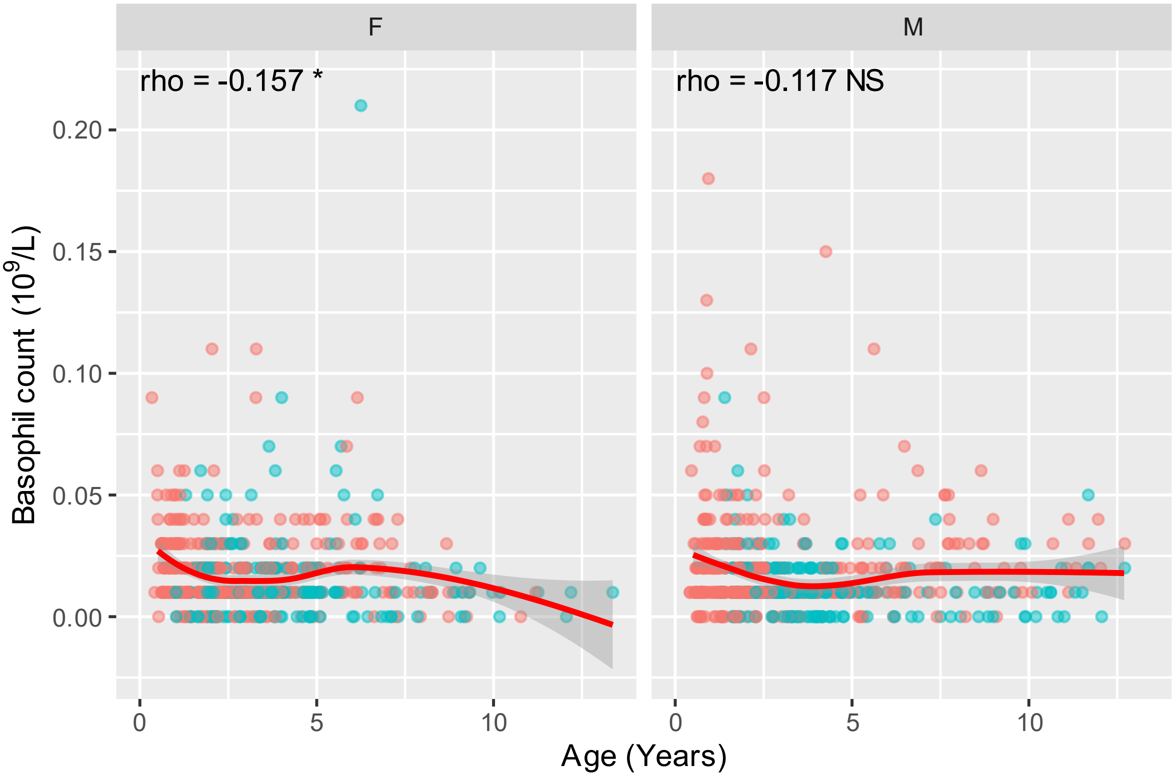


Figure S2 36. Basophil count according to sex and age in common marmosets. Points represent individual animals (red = outdoor access, cyan = indoor only). The red line indicates a Loess moving average and the grey area indicates the 95% confidence interval. Rho is Spearman’s rank correlation for parameter and Age; NS = not significant, asterisks indicate statistical significance levels * < 0.001, ** < 0.0001, *** < 0.00001, **** < 0.000001.
