# Supplementary material for: Hematological and Serum Biochemical Reference Intervals for Alphaxalone Sedated Common Marmosets (Callithrix jacchus)
Source: Animals (Basel). 2024 Mar 3;14(5):790. doi: 10.3390/ani14050790 (PMC10930924; doi:10.3390/ani14050790)
Supplement: Supplementary file 1 [file animals-14-00790-s001.zip › Figure S3.pdf]

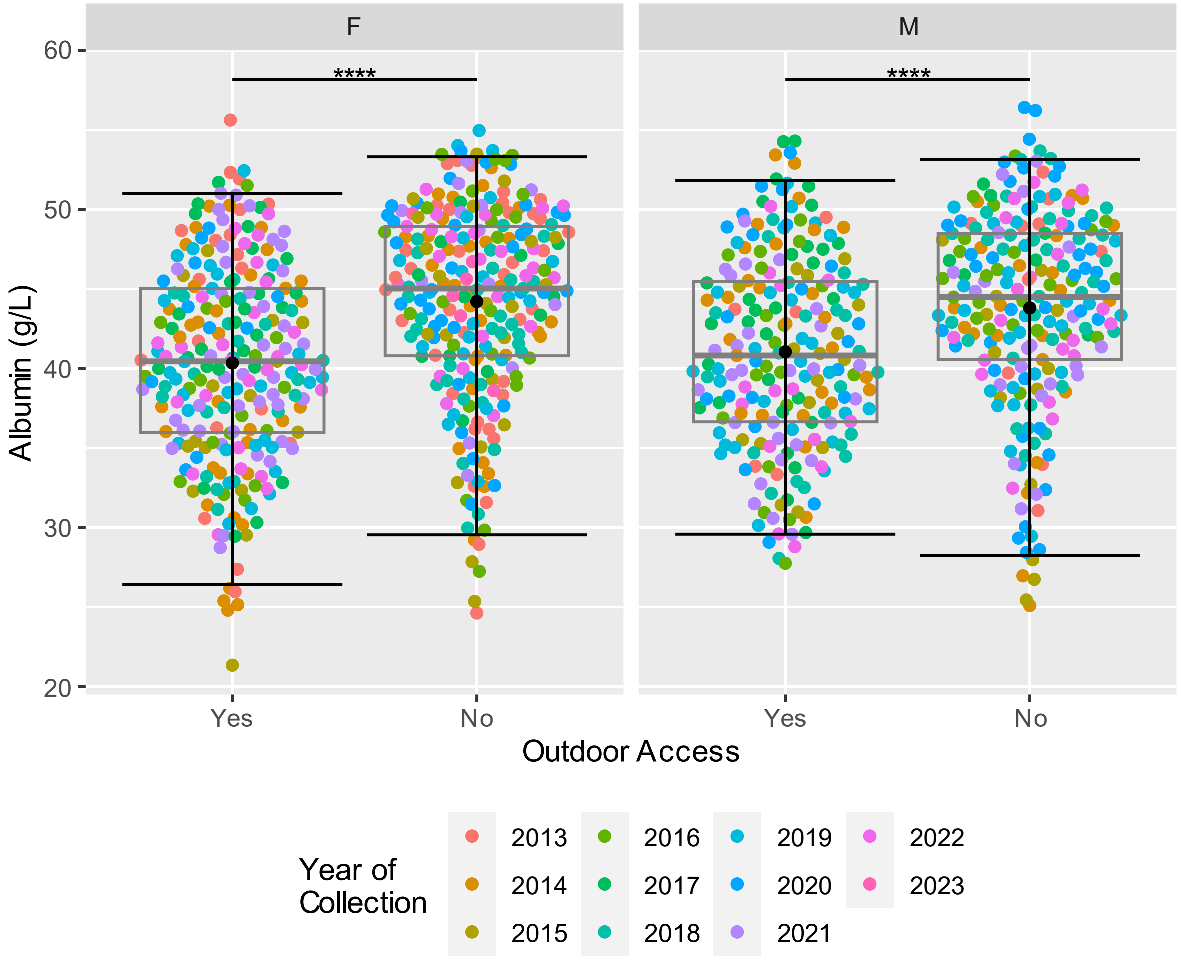

Figure S3 1. Albumin levels according to outdoor access in adult common marmosets. Boxes indicate median and quartile ranges; black point indicates arithmetic mean and error bars indicate the normal range. Symbol colours indicate year of collection; NS = not significant, NS = not significant, asterisks indicate statistical significance levels * < 0.001, ** < 0.0001, *** < 0.00001, **** < 0.000001

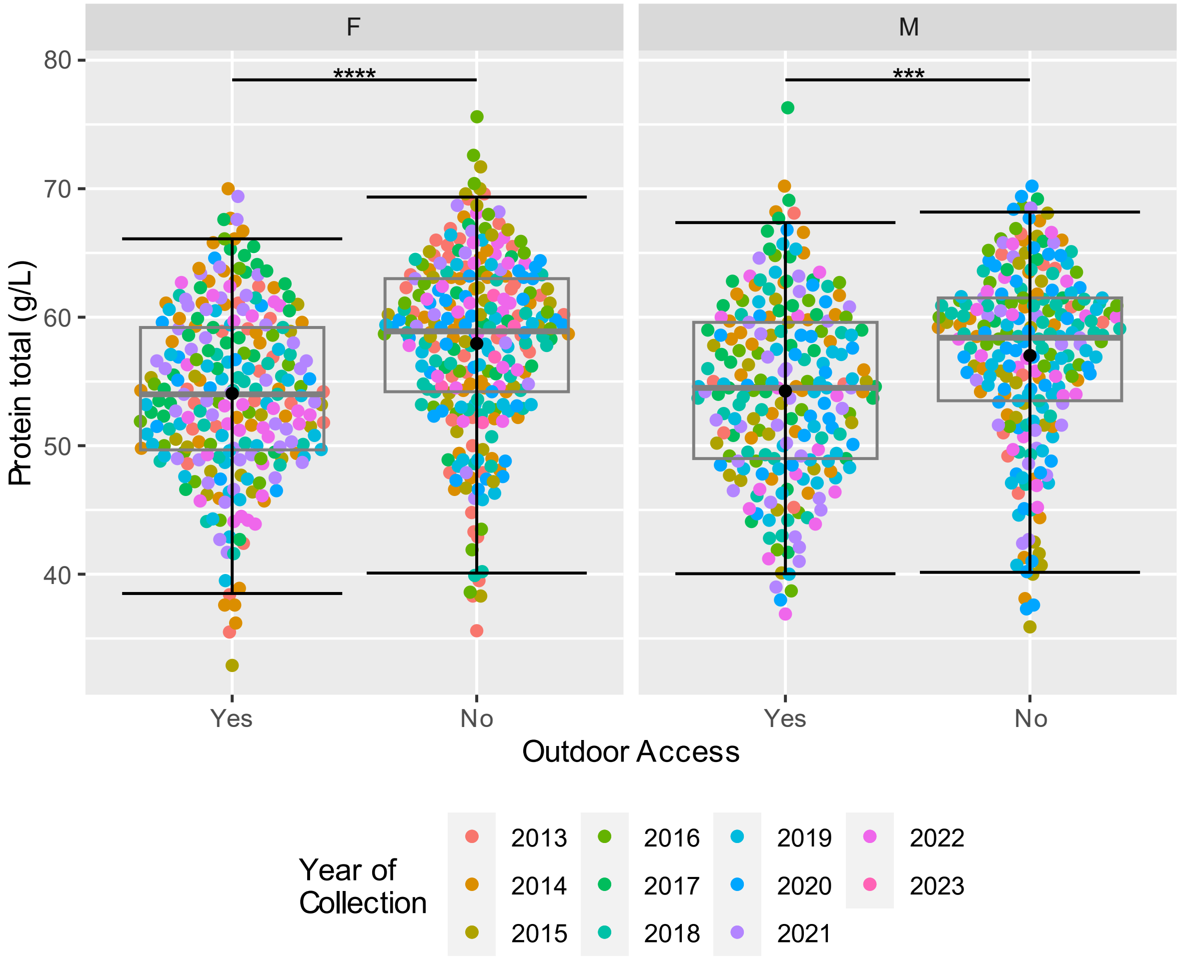

Figure S3 2. Total Protein levels according to outdoor access in adult common marmosets. Boxes indicate median and quartile ranges; black point indicates arithmetic mean and error bars indicate the normal range. Symbol colours indicate year of collection; NS = not significant, asterisks indicate statistical significance levels * < 0.001, ** < 0.0001, *** < 0.00001, **** < 0.000001

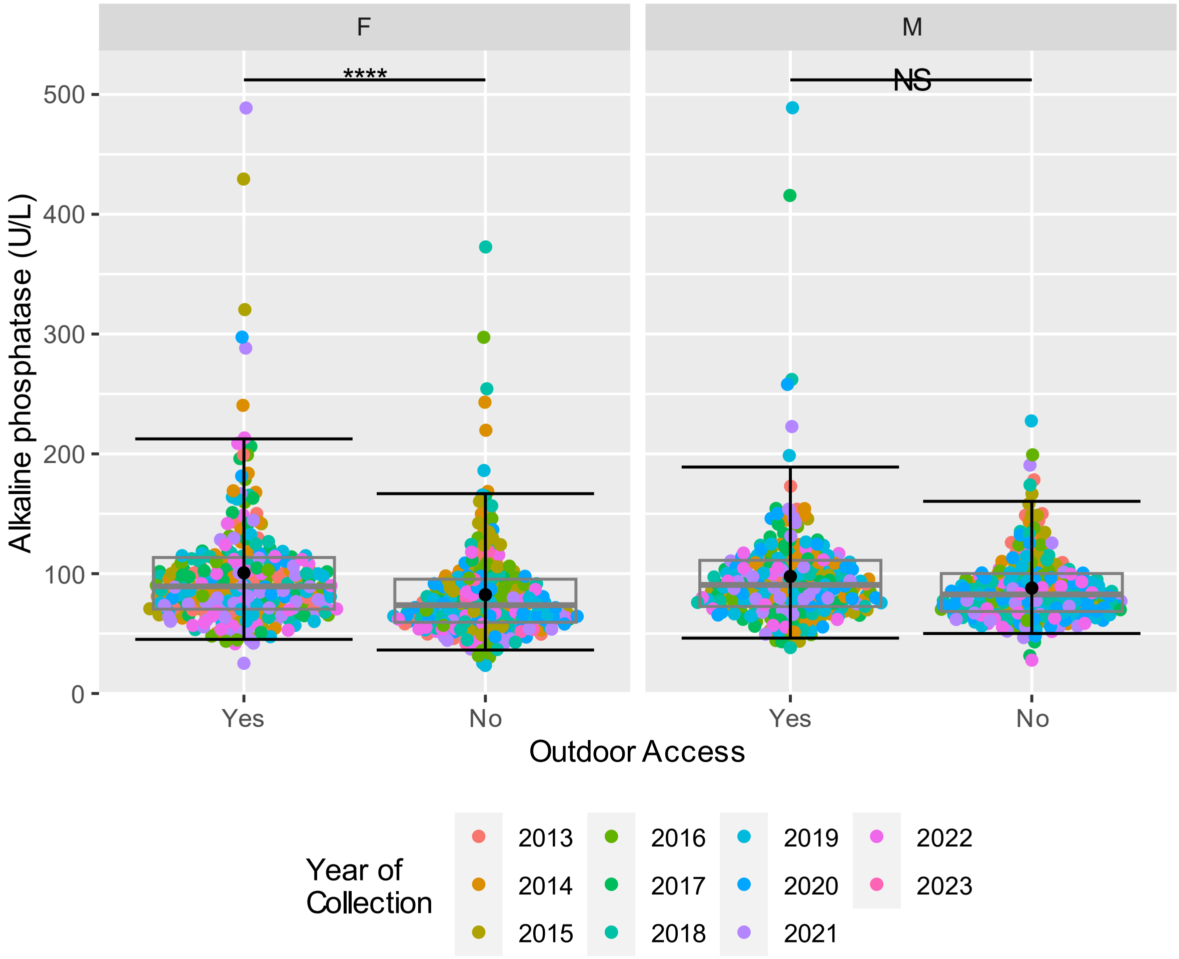

Figure S3 3. Alkaline Phosphatase levels according to outdoor access in adult common marmosets. Boxes indicate median and quartile ranges; black point indicates arithmetic mean and error bars indicate the normal range. Symbol colours indicate year of collection; NS = not significant, asterisks indicate statistical significance levels * < 0.001, ** < 0.0001, *** < 0.00001, **** < 0.000001

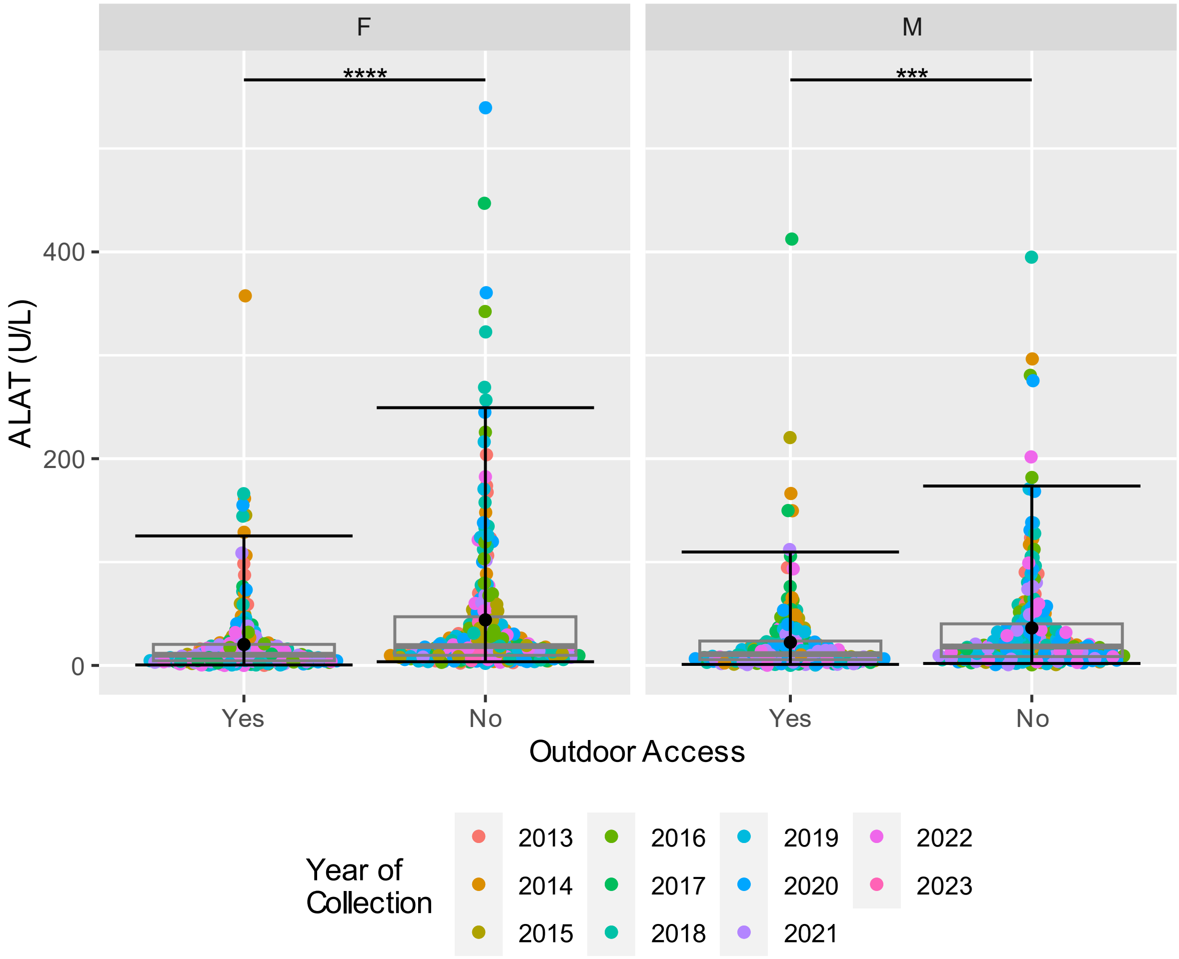

Figure S3 4. ALAT levels according to outdoor access in adult common marmosets. Boxes indicate median and quartile ranges; black point indicates arithmetic mean and error bars indicate the normal range. Symbol colours indicate year of collection; NS = not significant, asterisks indicate statistical significance levels * < 0.001, ** < 0.0001, *** < 0.00001, **** < 0.000001

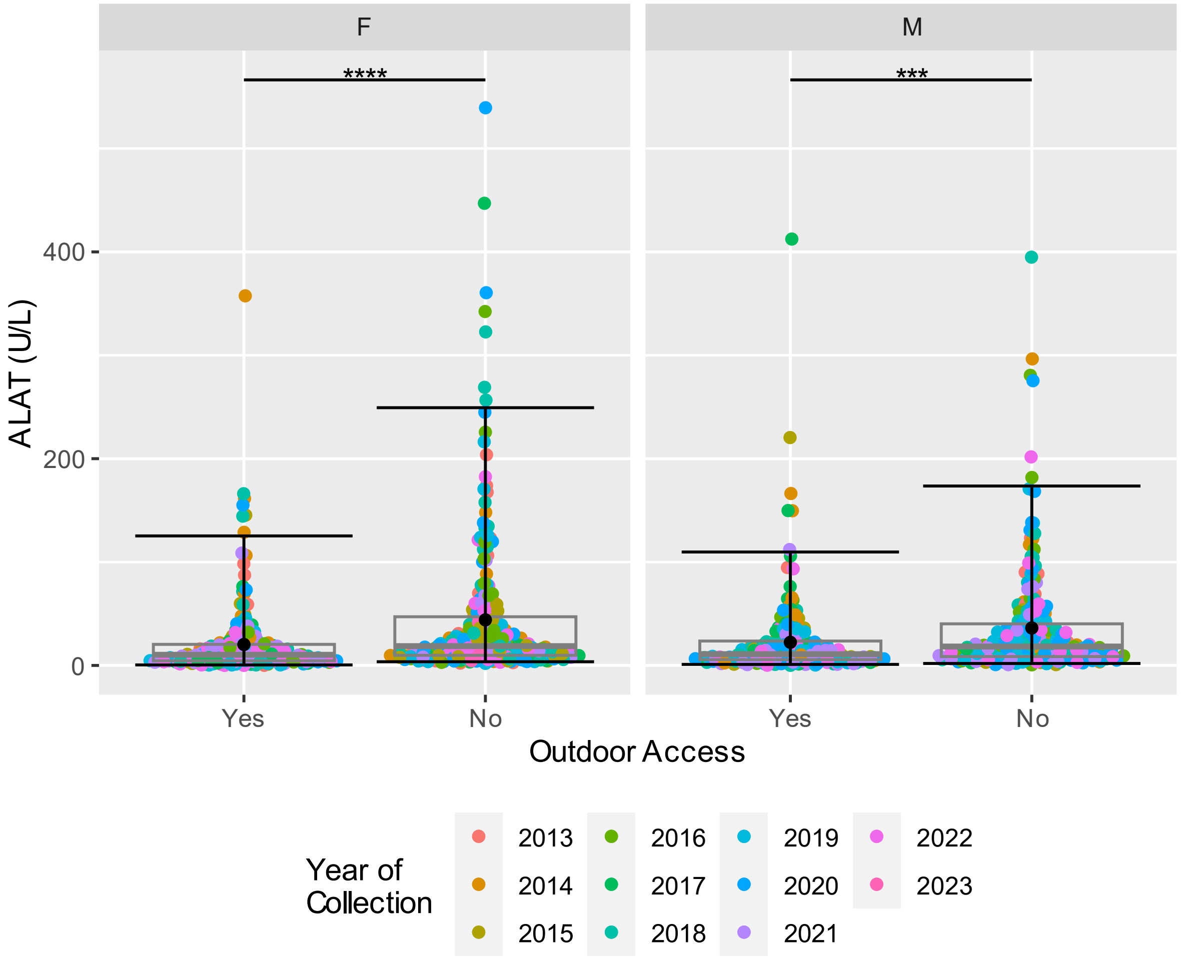

Figure S3 5. ASAT levels according to outdoor access in adult common marmosets. Boxes indicate median and quartile ranges; black point indicates arithmetic mean and error bars indicate the normal range. Symbol colours indicate year of collection; NS = not significant, asterisks indicate statistical significance levels * < 0.001, ** < 0.0001, *** < 0.00001, **** < 0.000001

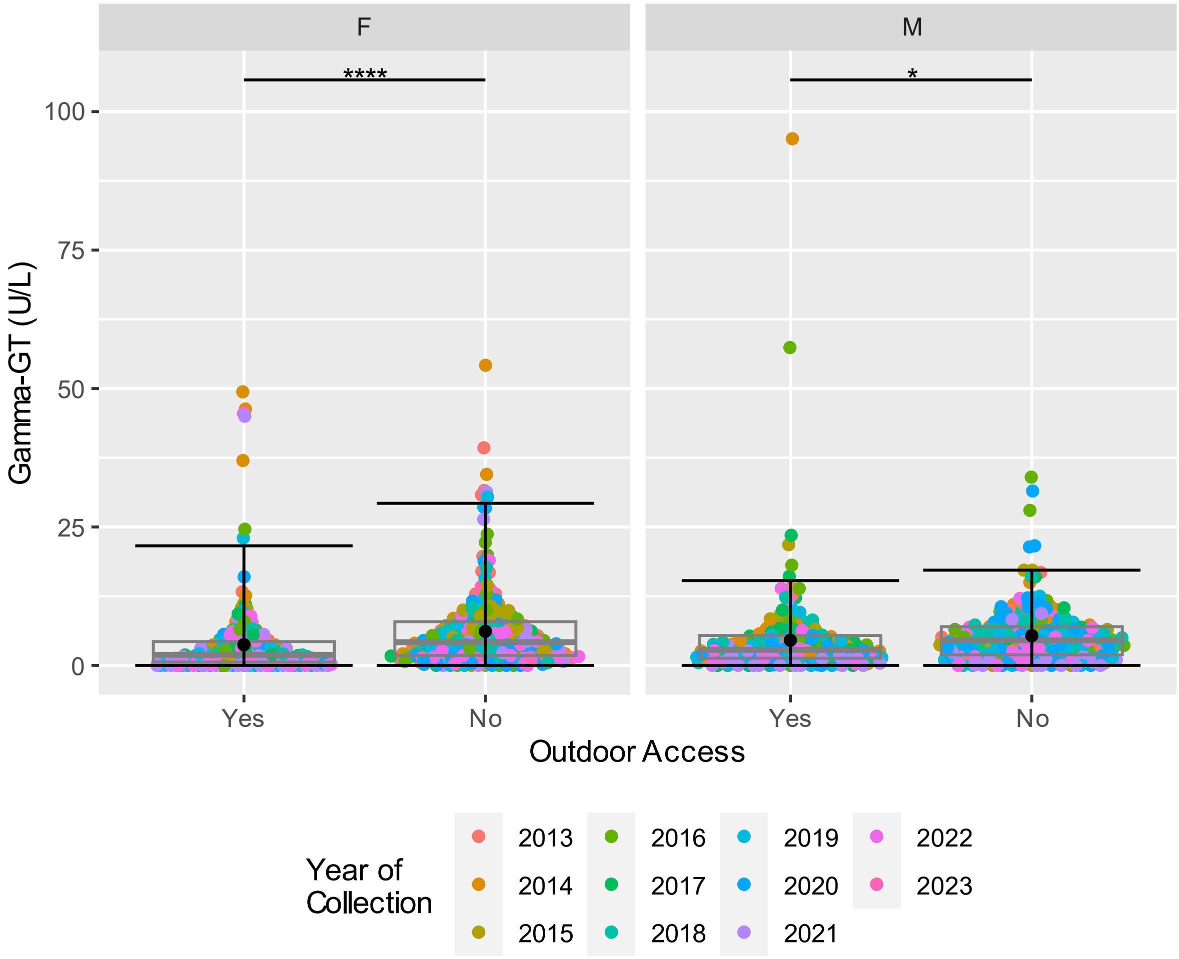

Figure S3 6. Gamma-GT levels according to outdoor access in adult common marmosets. Boxes indicate median and quartile ranges; black point indicates arithmetic mean and error bars indicate the normal range. Symbol colours indicate year of collection; NS = not significant, asterisks indicate statistical significance levels * < 0.001, ** < 0.0001, *** < 0.00001, **** < 0.000001

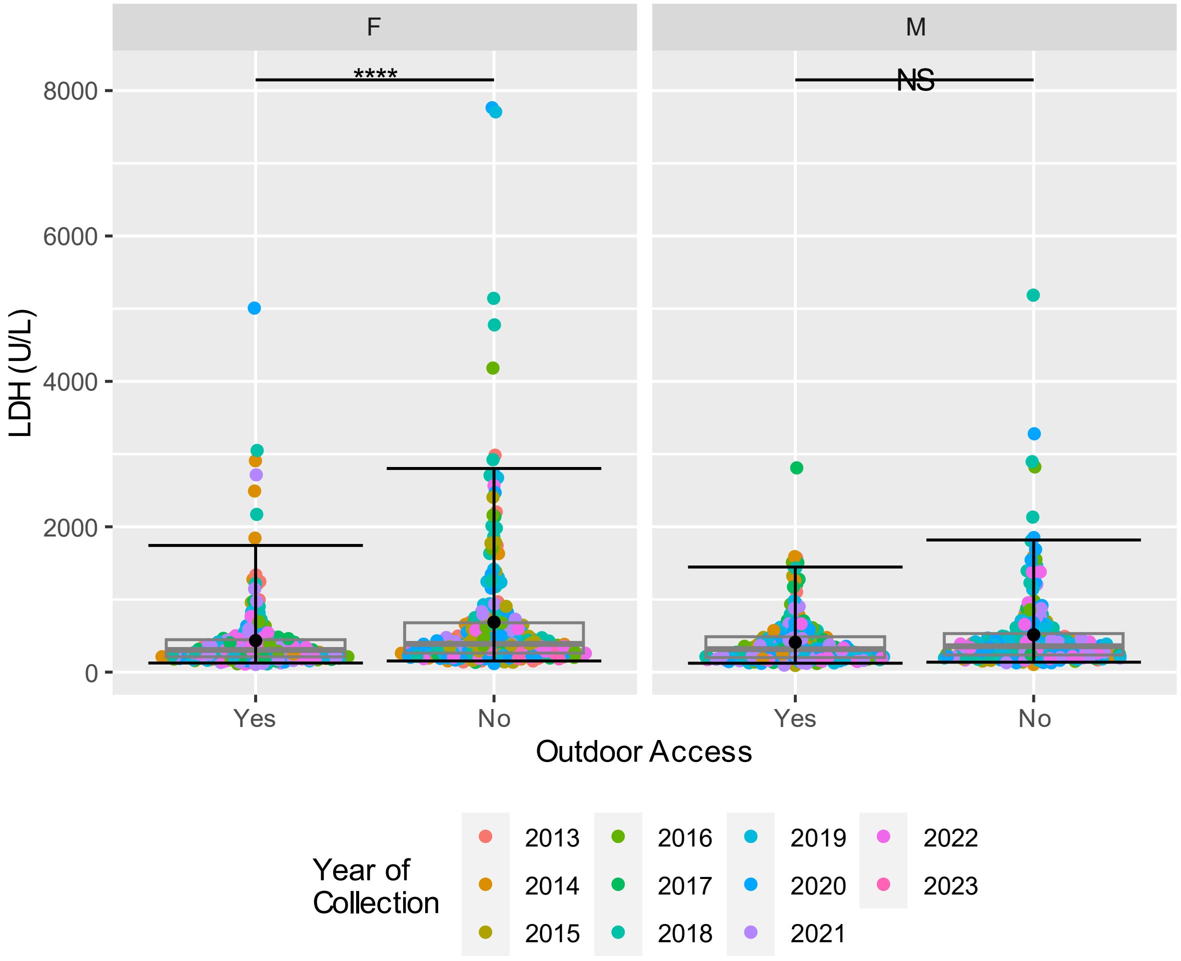

Figure S3 7. LDH levels according to outdoor access in adult common marmosets. Boxes indicate median and quartile ranges; black point indicates arithmetic mean and error bars indicate the normal range. Symbol colours indicate year of collection; NS = not significant, asterisks indicate statistical significance levels * < 0.001, ** < 0.0001, *** < 0.00001, **** < 0.000001

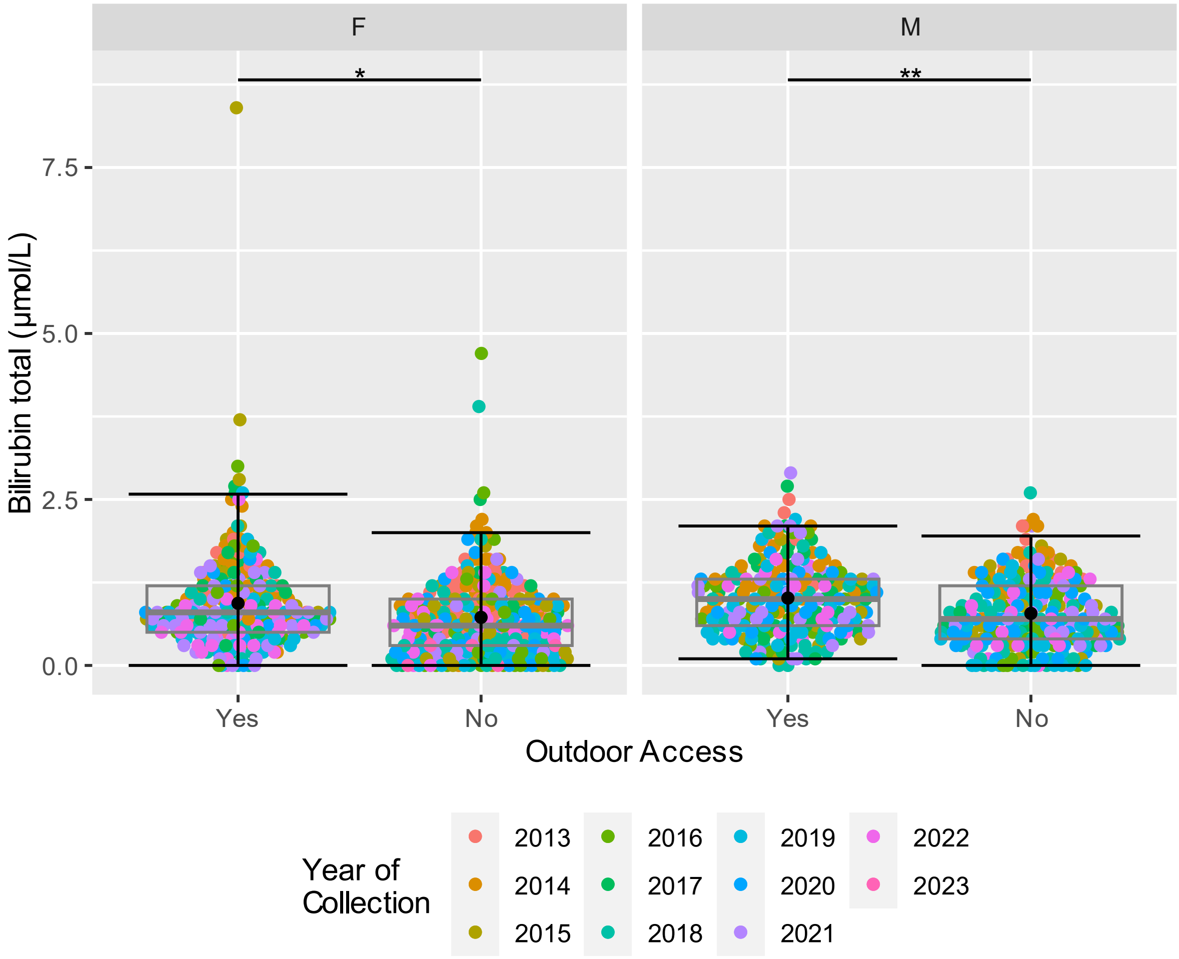

Figure S3 8. Total Bilirubin levels according to outdoor access in adult common marmosets. Boxes indicate median and quartile ranges; black point indicates arithmetic mean and error bars indicate the normal range. Symbol colours indicate year of collection; NS = not significant, asterisks indicate statistical significance levels * < 0.001, ** < 0.0001, *** < 0.00001, **** < 0.000001

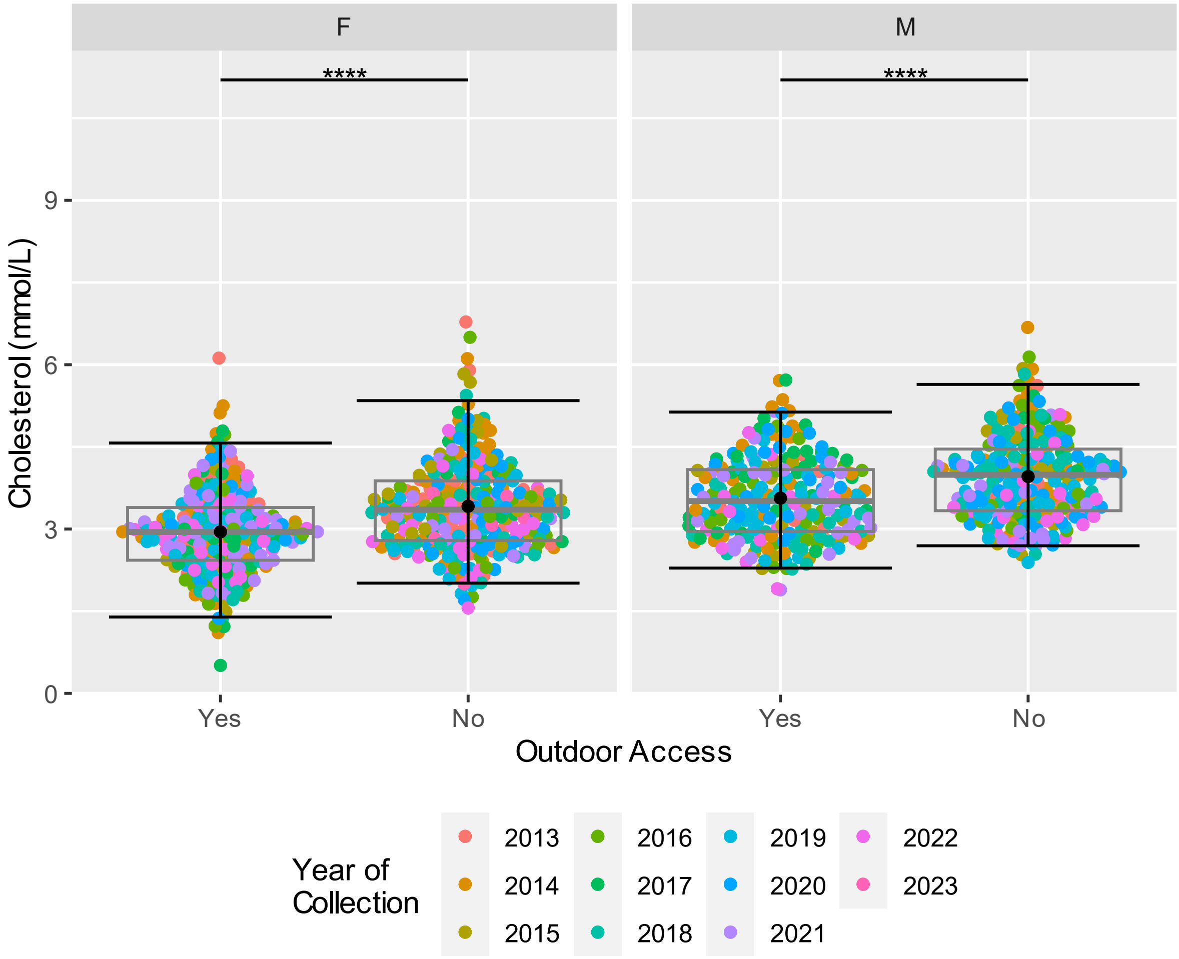

Figure S3 9. Cholesterol levels according to outdoor access in adult common marmosets. Boxes indicate median and quartile ranges; black point indicates arithmetic mean and error bars indicate the normal range. Symbol colours indicate year of collection; NS = not significant, asterisks indicate statistical significance levels * < 0.001, ** < 0.0001, *** < 0.00001, **** < 0.000001

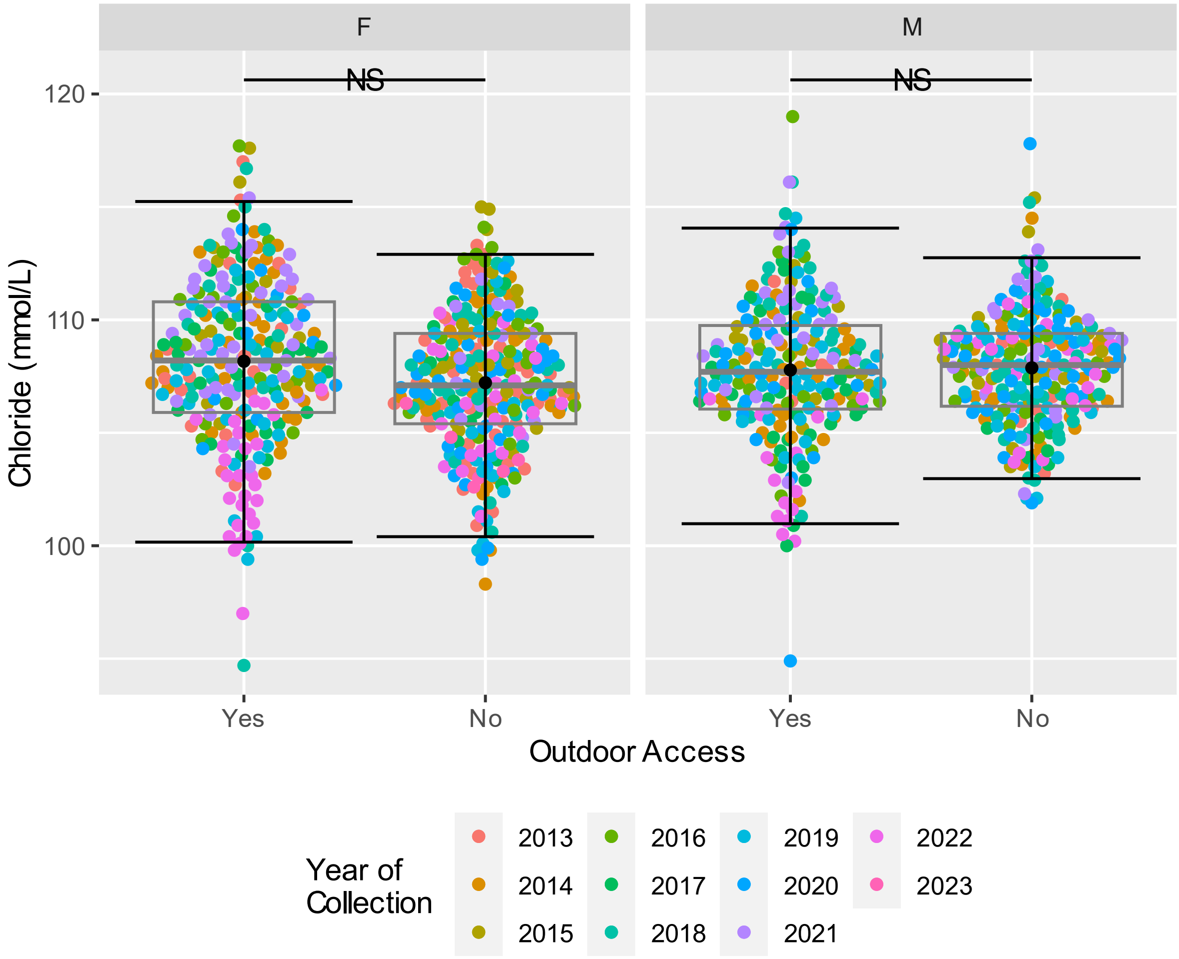

Figure S3 10. Chloride levels according to outdoor access in adult common marmosets. Boxes indicate median and quartile ranges; black point indicates arithmetic mean and error bars indicate the normal range. Symbol colours indicate year of collection; NS = not significant, asterisks indicate statistical significance levels * < 0.001, ** < 0.0001, *** < 0.00001, **** < 0.000001

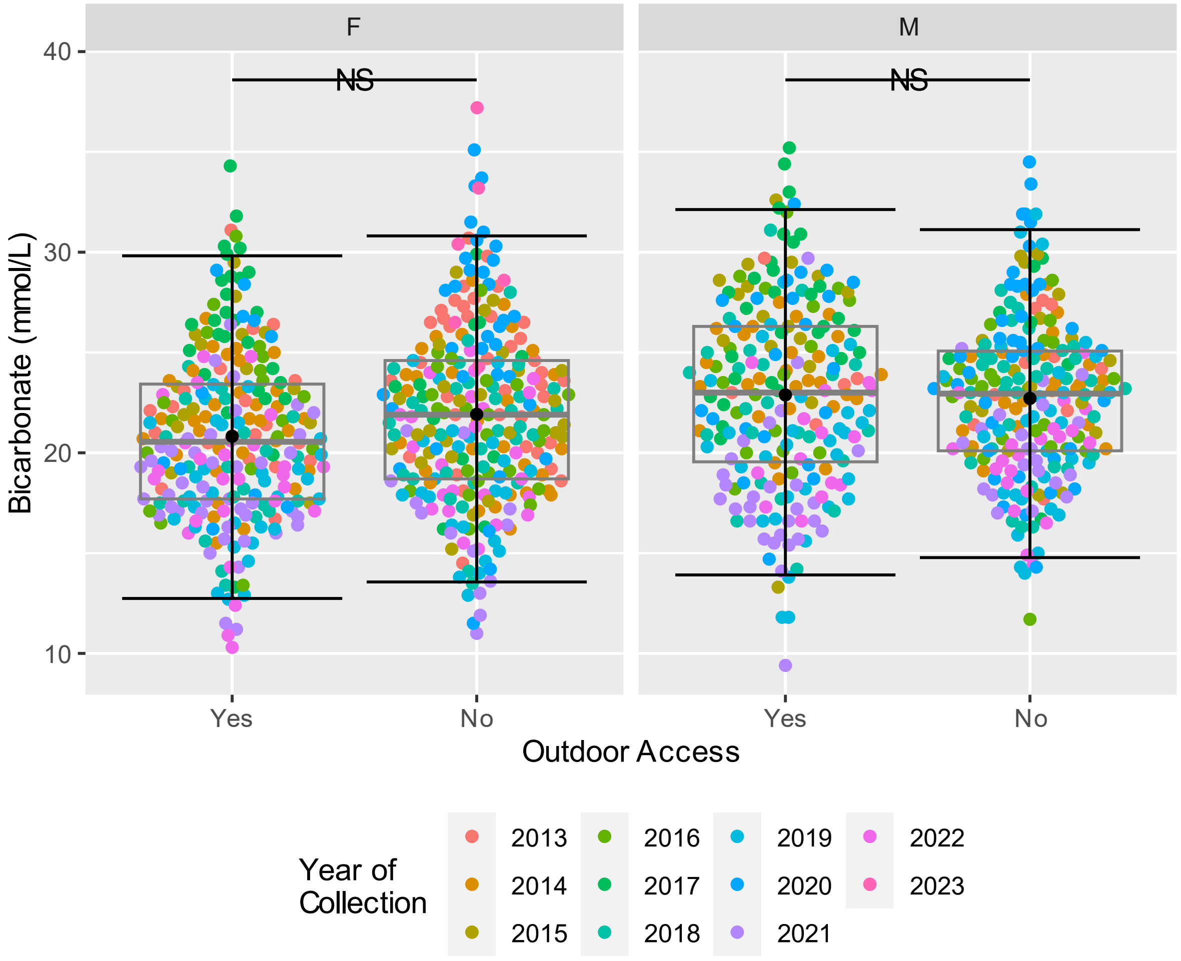

Figure S3 11. Bicarbonate levels according to outdoor access in adult common marmosets. Boxes indicate median and quartile ranges; black point indicates arithmetic mean and error bars indicate the normal range. Symbol colours indicate year of collection; NS = not significant, asterisks indicate statistical significance levels * < 0.001, ** < 0.0001, *** < 0.00001, **** < 0.000001

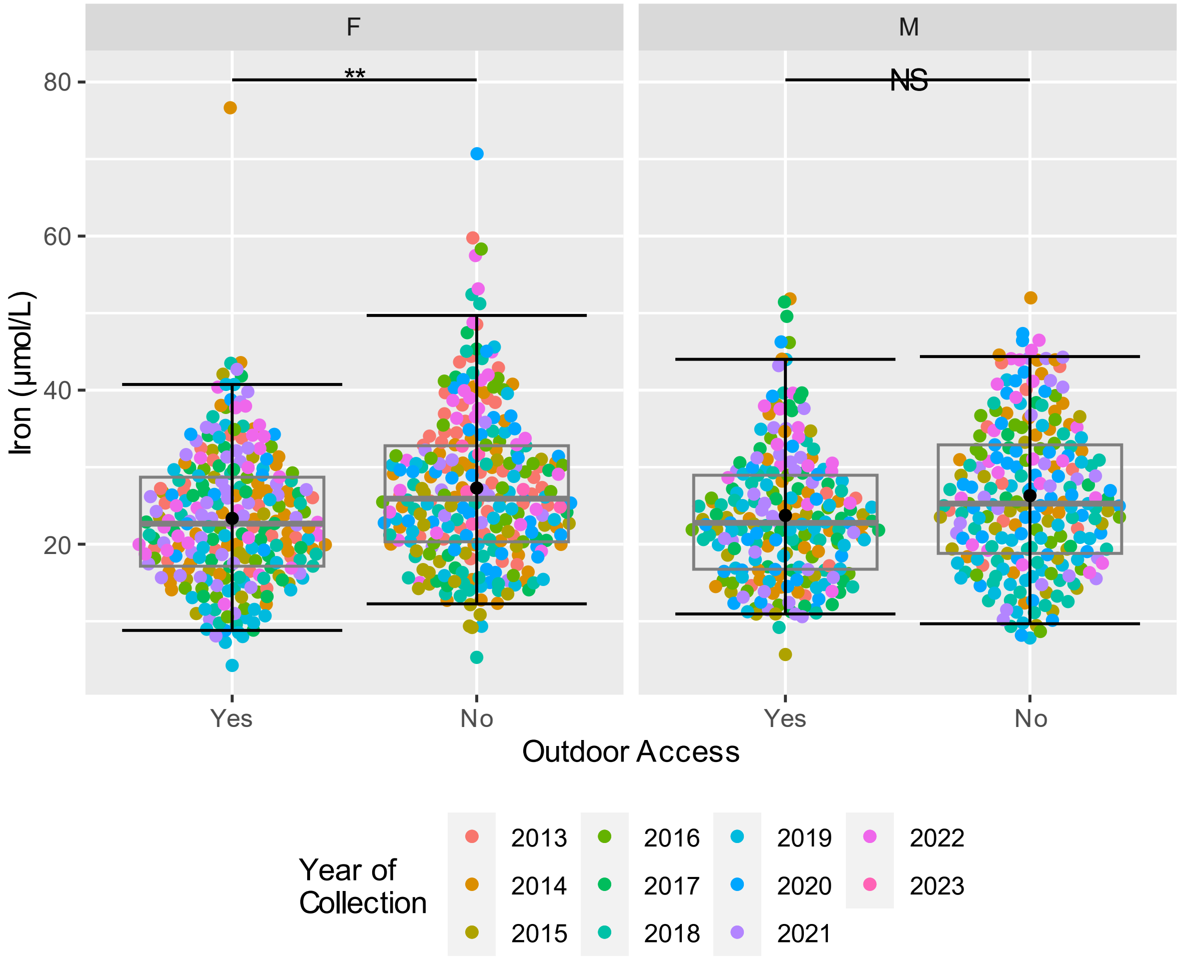

Figure S3 12. Iron levels according to outdoor access in adult common marmosets. Boxes indicate median and quartile ranges; black point indicates arithmetic mean and error bars indicate the normal range. Symbol colours indicate year of collection; NS = not significant, asterisks indicate statistical significance levels * < 0.001, ** < 0.0001, *** < 0.00001, **** < 0.000001

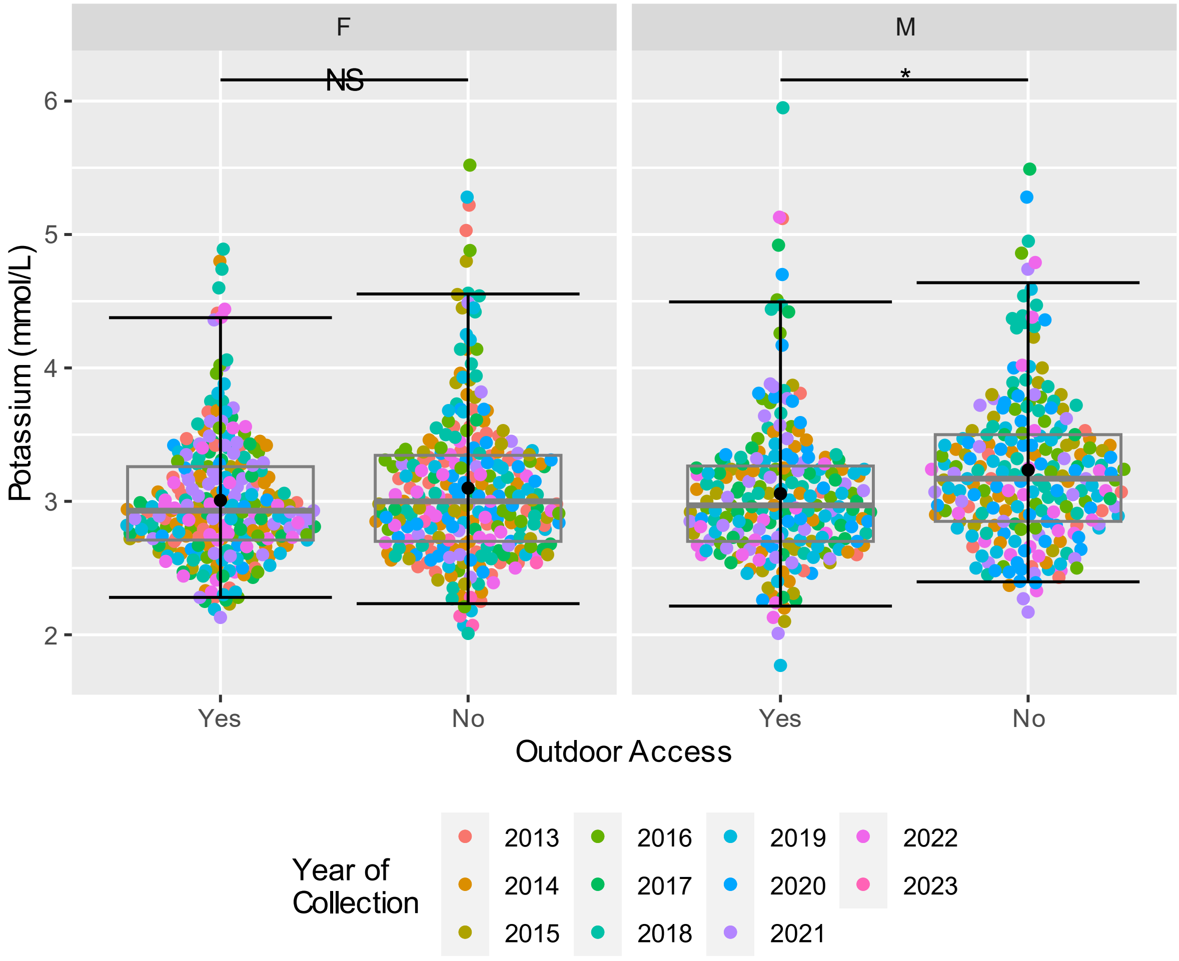

Figure S3 13. Potassium levels according to outdoor access in adult common marmosets. Boxes indicate median and quartile ranges; black point indicates arithmetic mean and error bars indicate the normal range. Symbol colours indicate year of collection; NS = not significant, asterisks indicate statistical significance levels * < 0.001, ** < 0.0001, *** < 0.00001, **** < 0.000001

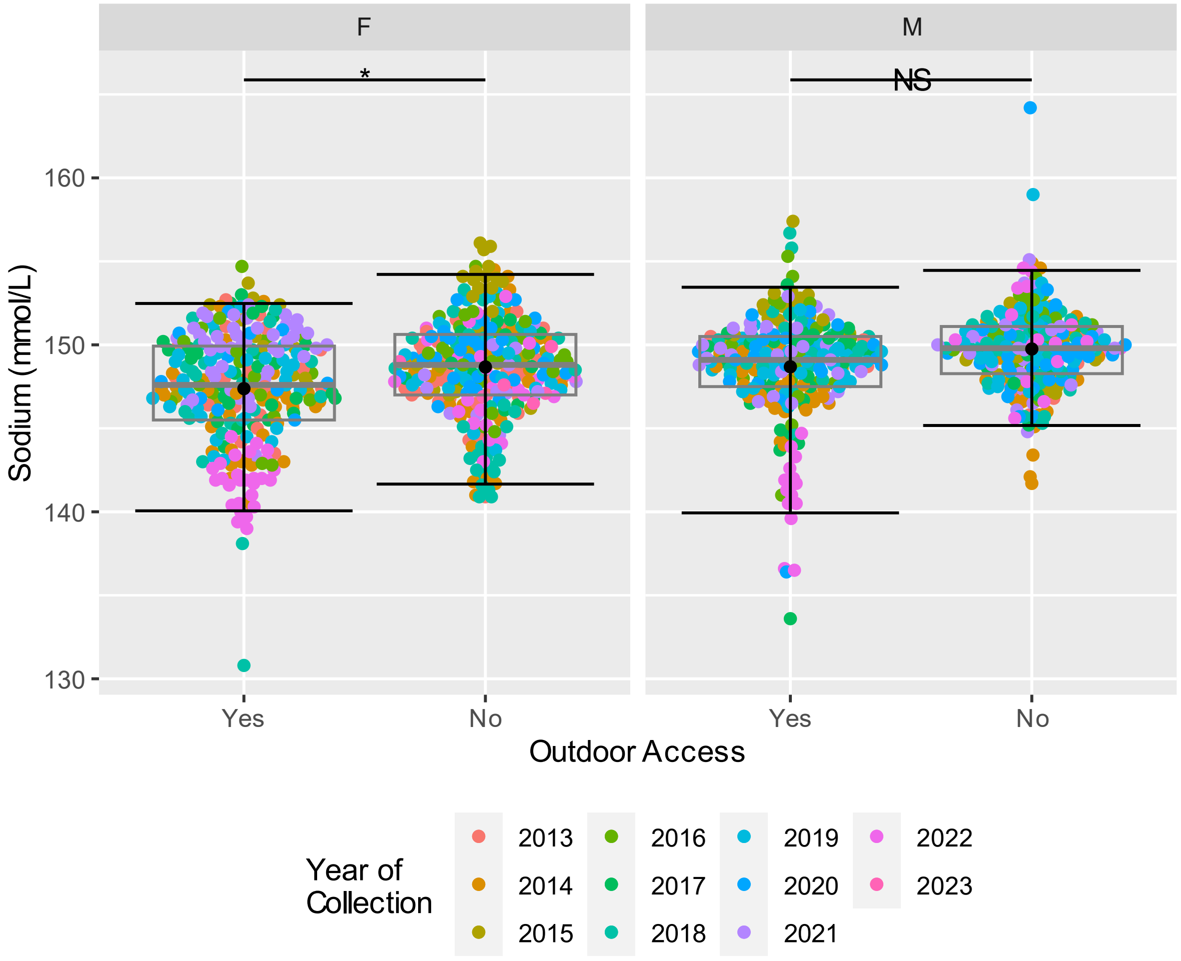

Figure S3 14. Sodium levels according to outdoor access in adult common marmosets. Boxes indicate median and quartile ranges; black point indicates arithmetic mean and error bars indicate the normal range. Symbol colours indicate year of collection; NS = not significant, asterisks indicate statistical significance levels * < 0.001, ** < 0.0001, *** < 0.00001, **** < 0.000001

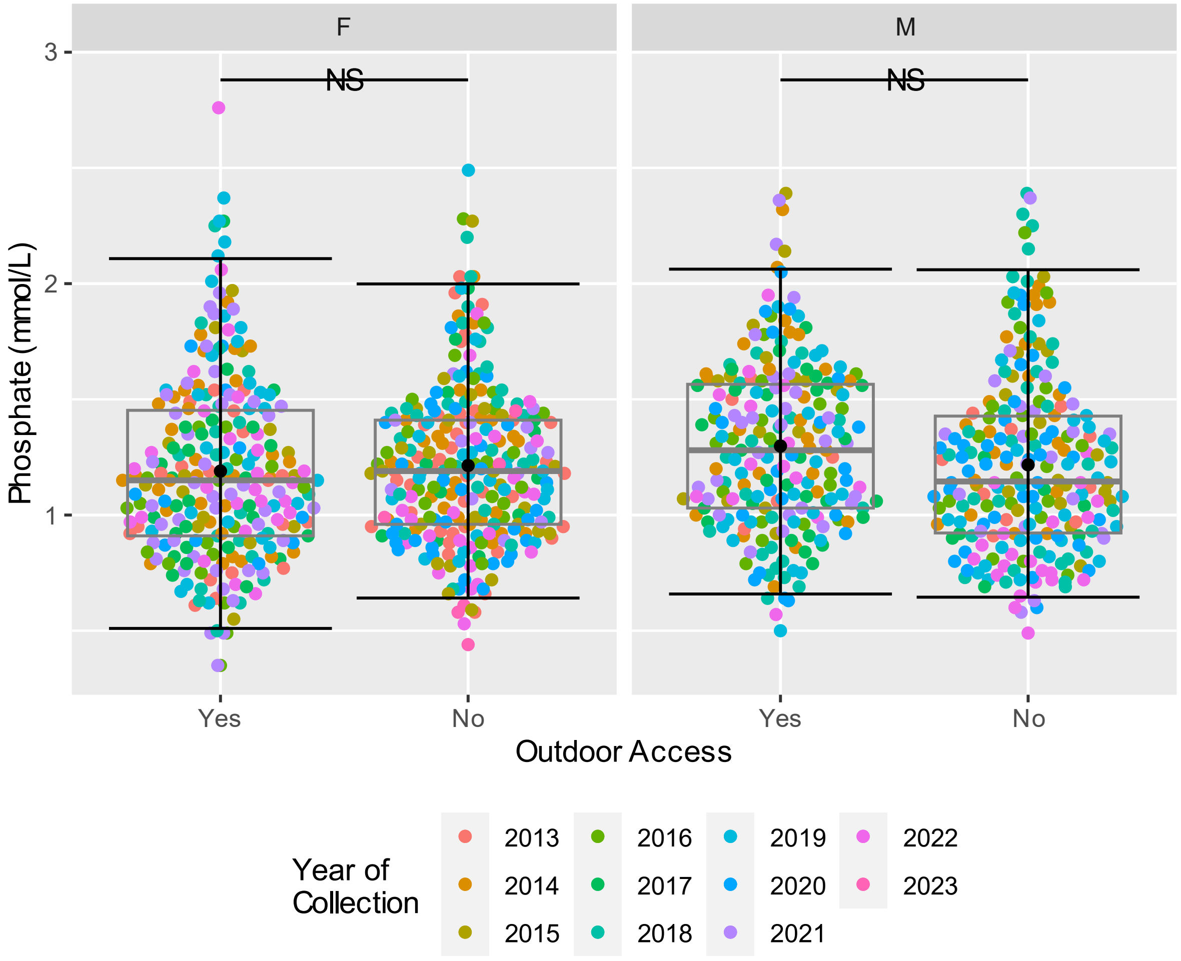

Figure S3 15. Phosphate levels according to outdoor access in adult common marmosets. Boxes indicate median and quartile ranges; black point indicates arithmetic mean and error bars indicate the normal range. Symbol colours indicate year of collection; NS = not significant, asterisks indicate statistical significance levels * < 0.001, ** < 0.0001, *** < 0.00001, **** < 0.000001

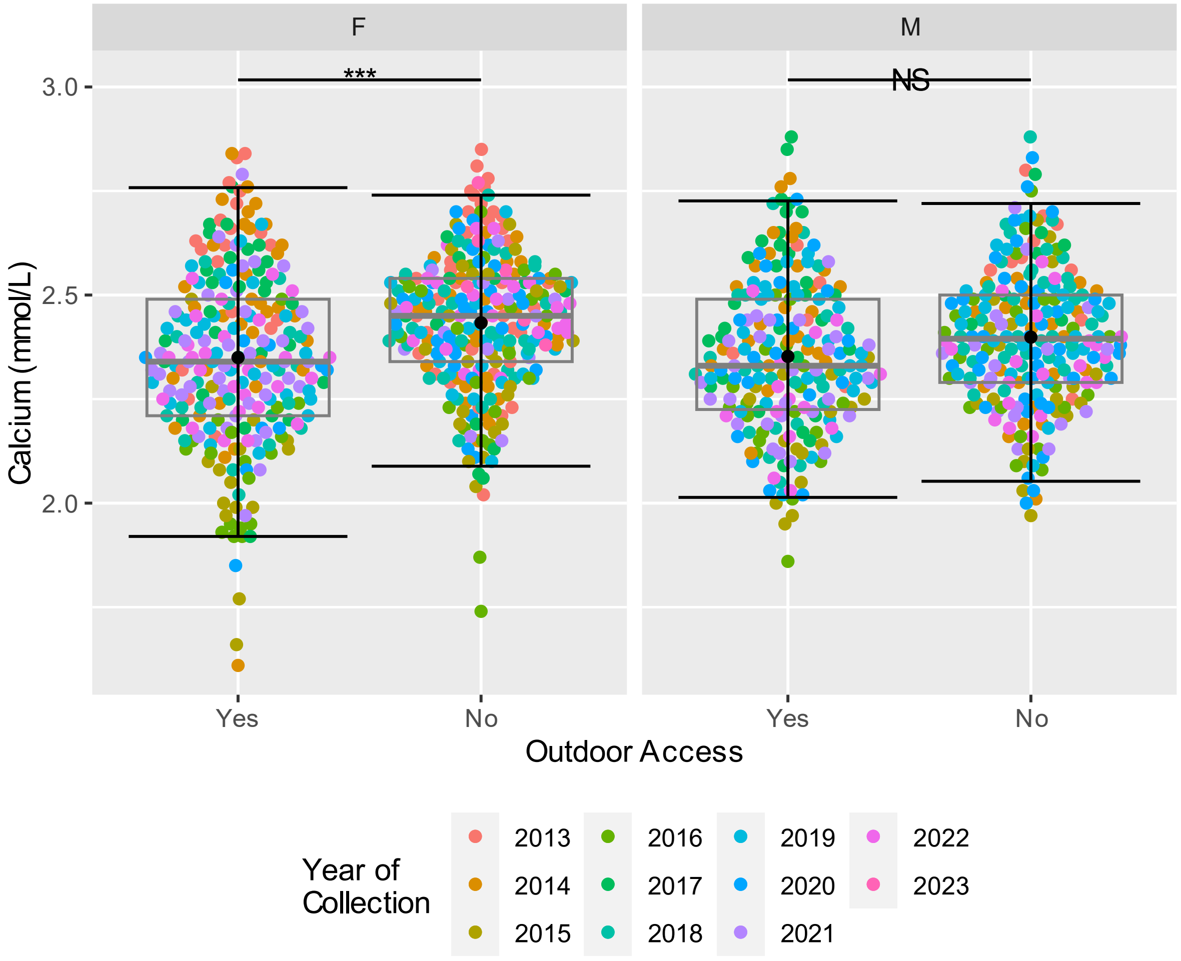

Figure S3 16. Calcium levels according to outdoor access in adult common marmosets. Boxes indicate median and quartile ranges; black point indicates arithmetic mean and error bars indicate the normal range. Symbol colours indicate year of collection; NS = not significant, asterisks indicate statistical significance levels * < 0.001, ** < 0.0001, *** < 0.00001, **** < 0.000001

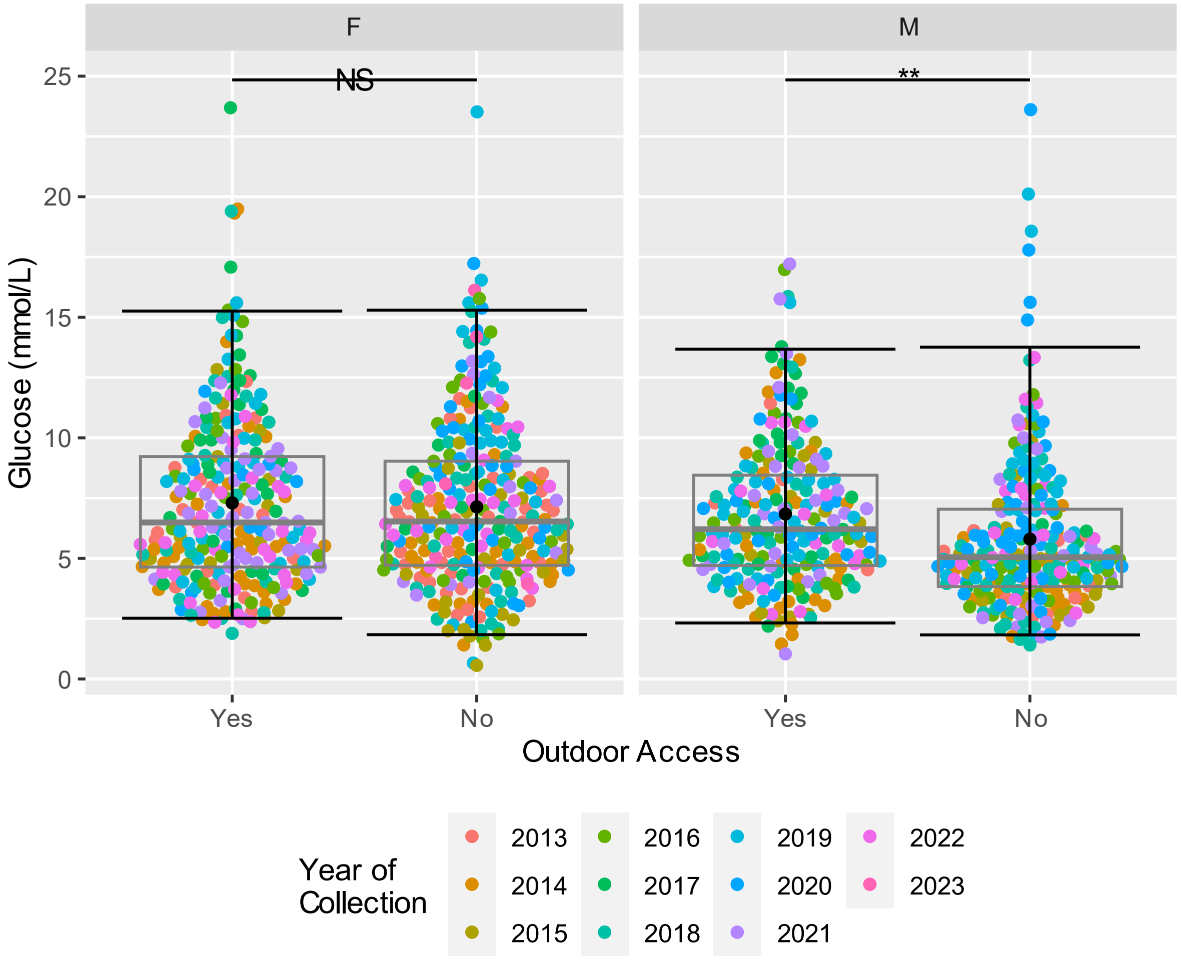

Figure S3 17. Glucose levels according to outdoor access in adult common marmosets. Boxes indicate median and quartile ranges; black point indicates arithmetic mean and error bars indicate the normal range. Symbol colours indicate year of collection; NS = not significant, asterisks indicate statistical significance levels * < 0.001, ** < 0.0001, *** < 0.00001, **** < 0.000001

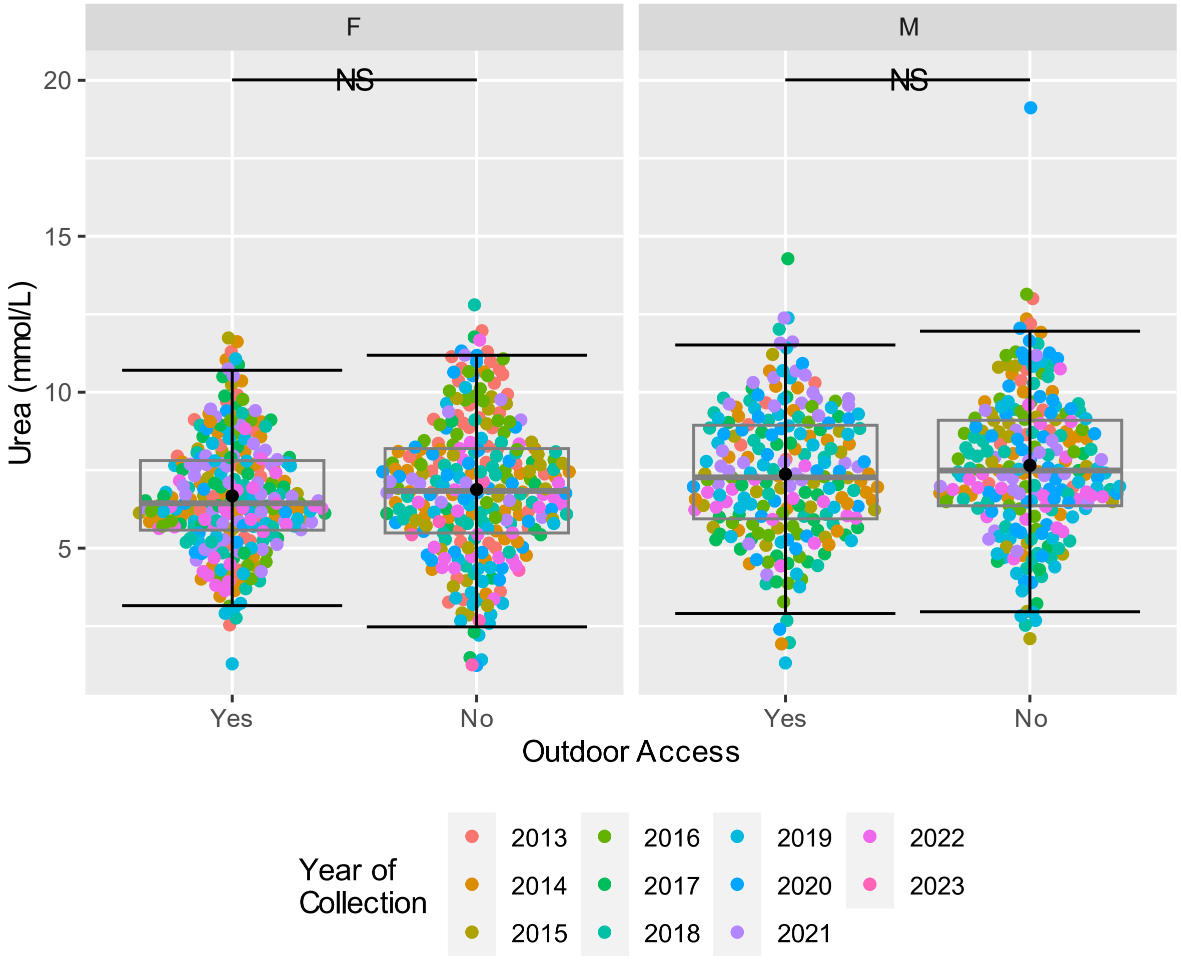

Figure S3 18. Urea levels according to outdoor access in adult common marmosets. Boxes indicate median and quartile ranges; black point indicates arithmetic mean and error bars indicate the normal range. Symbol colours indicate year of collection; NS = not significant, asterisks indicate statistical significance levels * < 0.001, ** < 0.0001, *** < 0.00001, **** < 0.000001

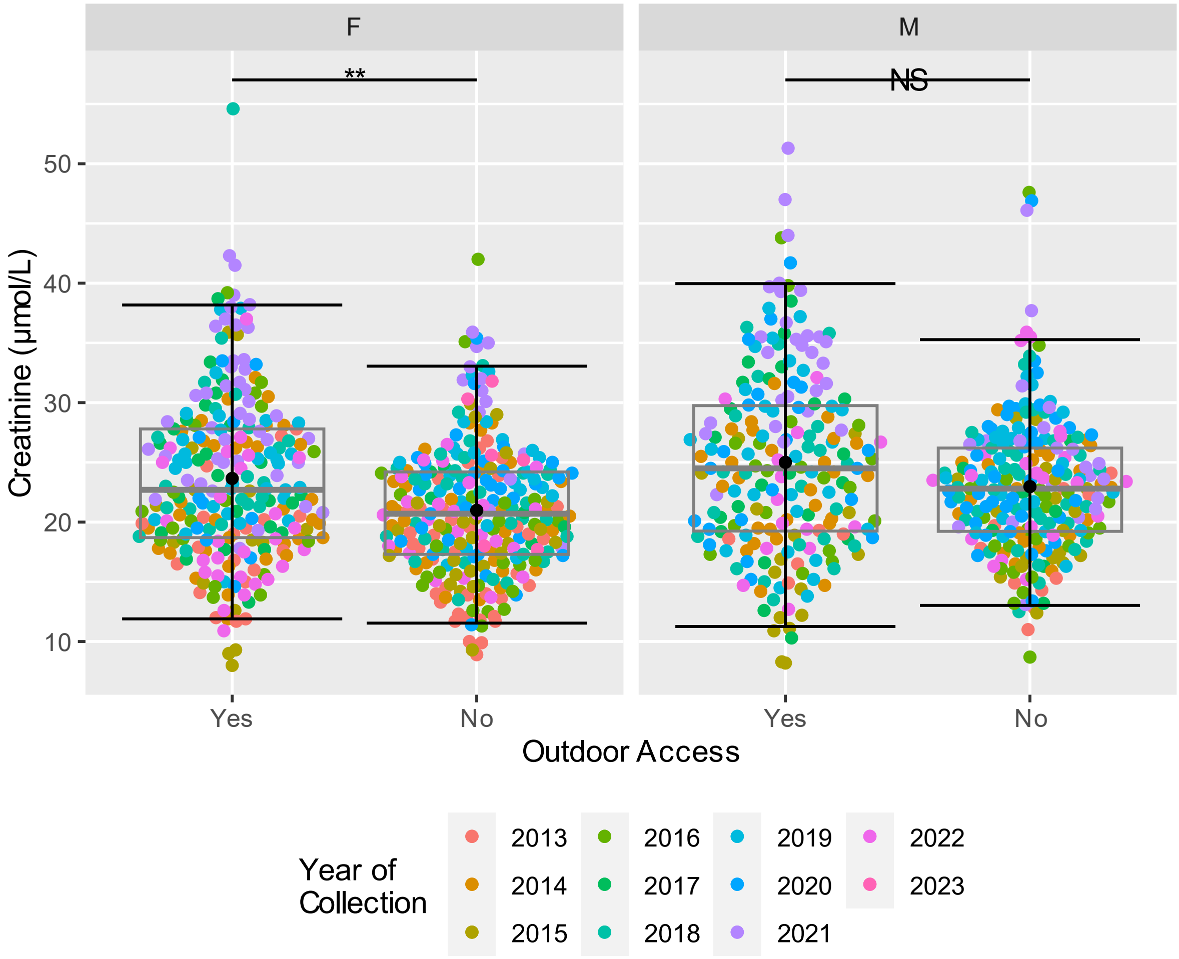

Figure S3 19. Creatinine levels according to outdoor access in adult common marmosets. Boxes indicate median and quartile ranges; black point indicates arithmetic mean and error bars indicate the normal range. Symbol colours indicate year of collection; NS = not significant, asterisks indicate statistical significance levels * < 0.001, ** < 0.0001, *** < 0.00001, **** < 0.000001

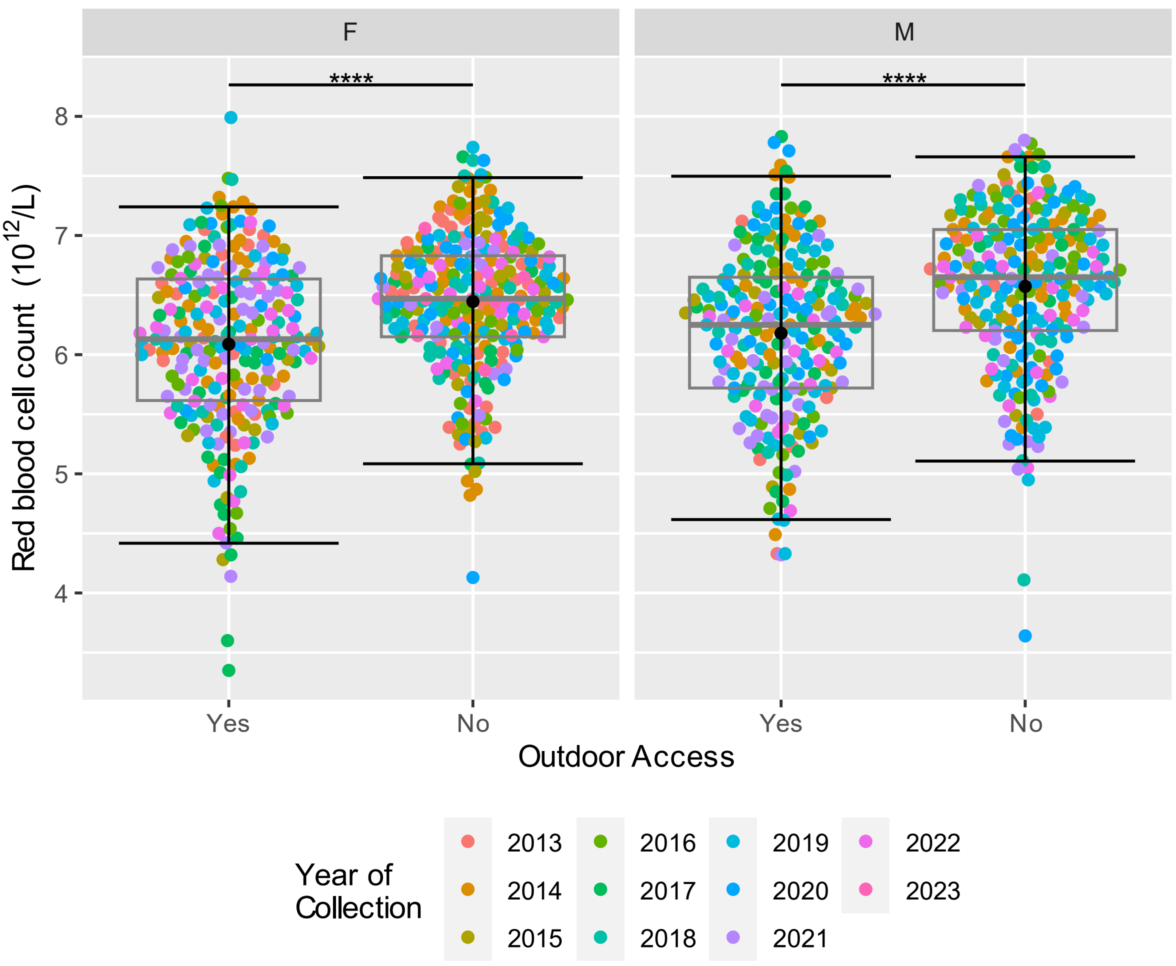

Figure S3 20. Red blood cell count according to outdoor access in adult common marmosets. Boxes indicate median and quartile ranges; black point indicates arithmetic mean and error bars indicate the normal range. Symbol colours indicate year of collection; NS = not significant, asterisks indicate statistical significance levels * < 0.001, ** < 0.0001, *** < 0.00001, **** < 0.000001

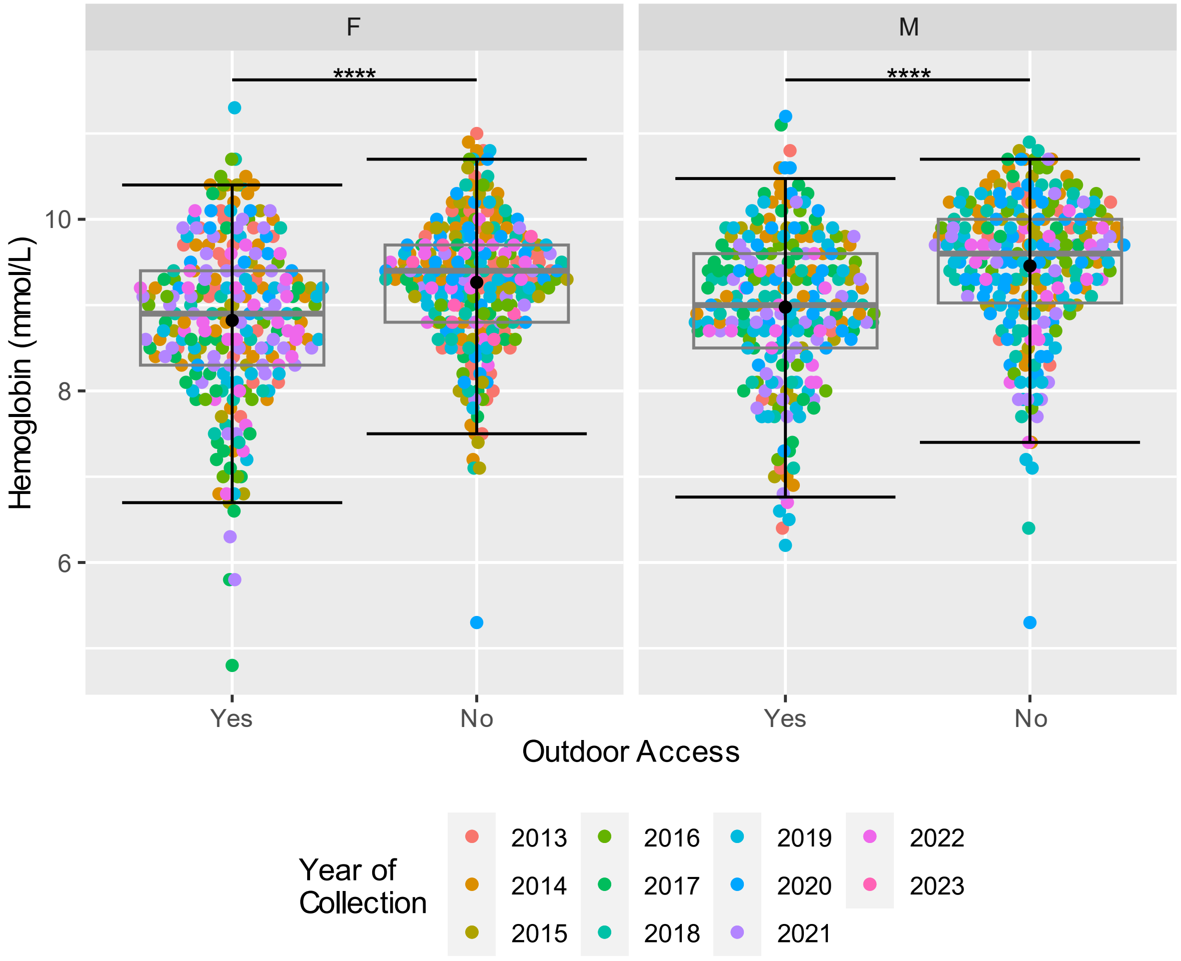

Figure S3 21. Haemoglobin levels according to outdoor access in adult common marmosets. Boxes indicate median and quartile ranges; black point indicates arithmetic mean and error bars indicate the normal range. Symbol colours indicate year of collection; NS = not significant, asterisks indicate statistical significance levels * < 0.001, ** < 0.0001, *** < 0.00001, **** < 0.000001

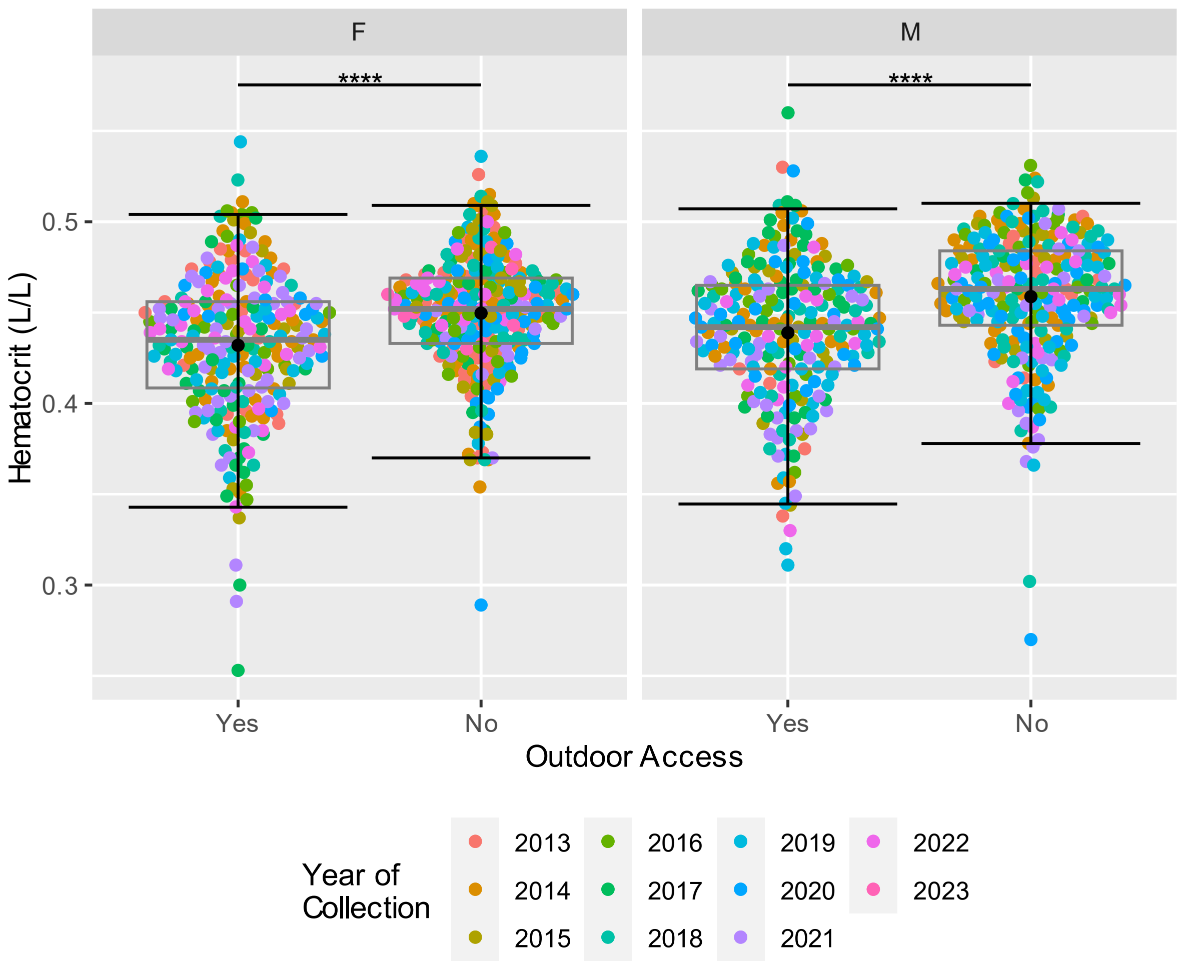

Figure S3 22. Haematocrit according to outdoor access in adult common marmosets. Boxes indicate median and quartile ranges; black point indicates arithmetic mean and error bars indicate the normal range. Symbol colours indicate year of collection; NS = not significant, asterisks indicate statistical significance levels * < 0.001, ** < 0.0001, *** < 0.00001, **** < 0.000001

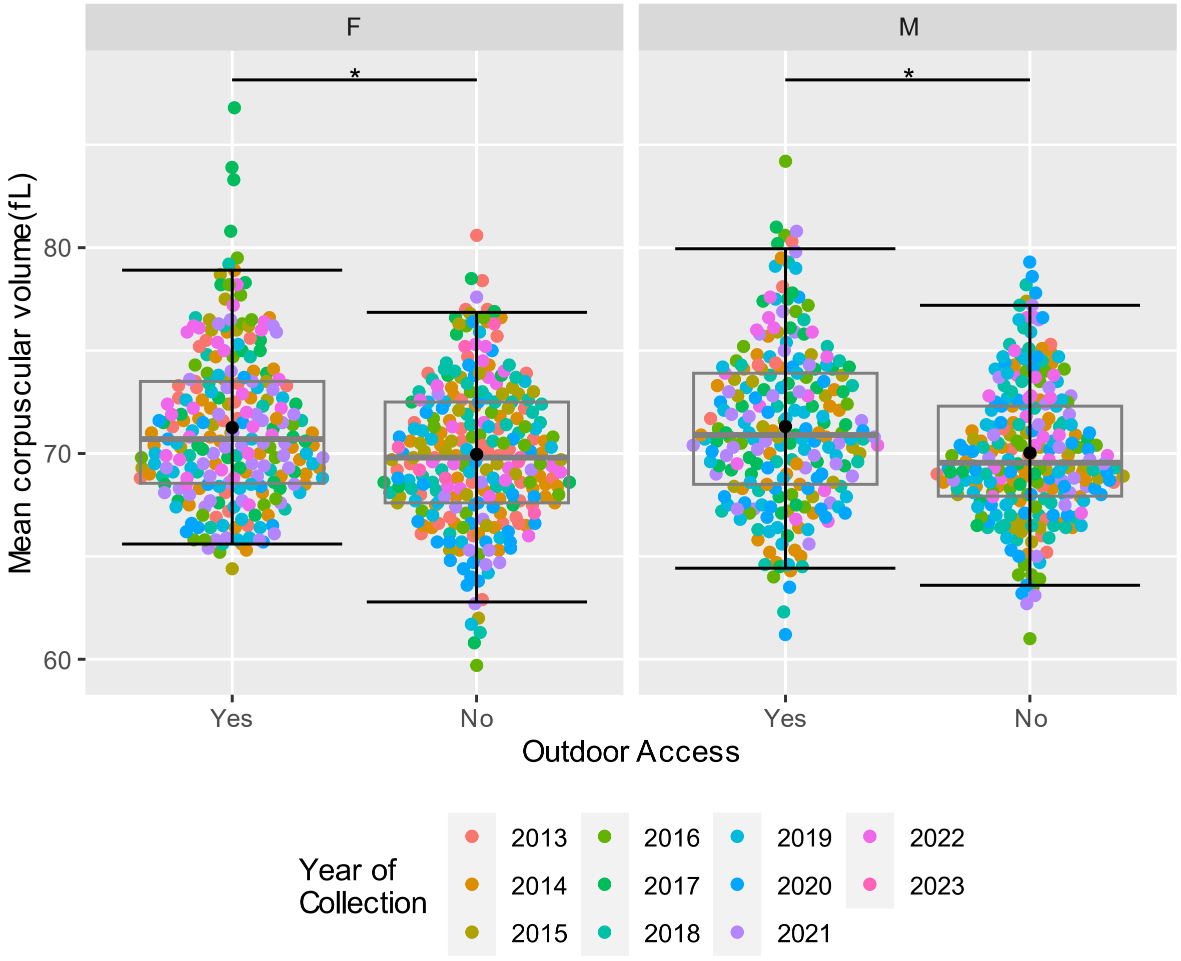

Figure S3 23. Mean Corpescular Volume according to outdoor access in adult common marmosets. Boxes indicate median and quartile ranges; black point indicates arithmetic mean and error bars indicate the normal range. Symbol colours indicate year of collection; NS = not significant, asterisks indicate statistical significance levels * < 0.001, ** < 0.0001, *** < 0.00001, **** < 0.000001

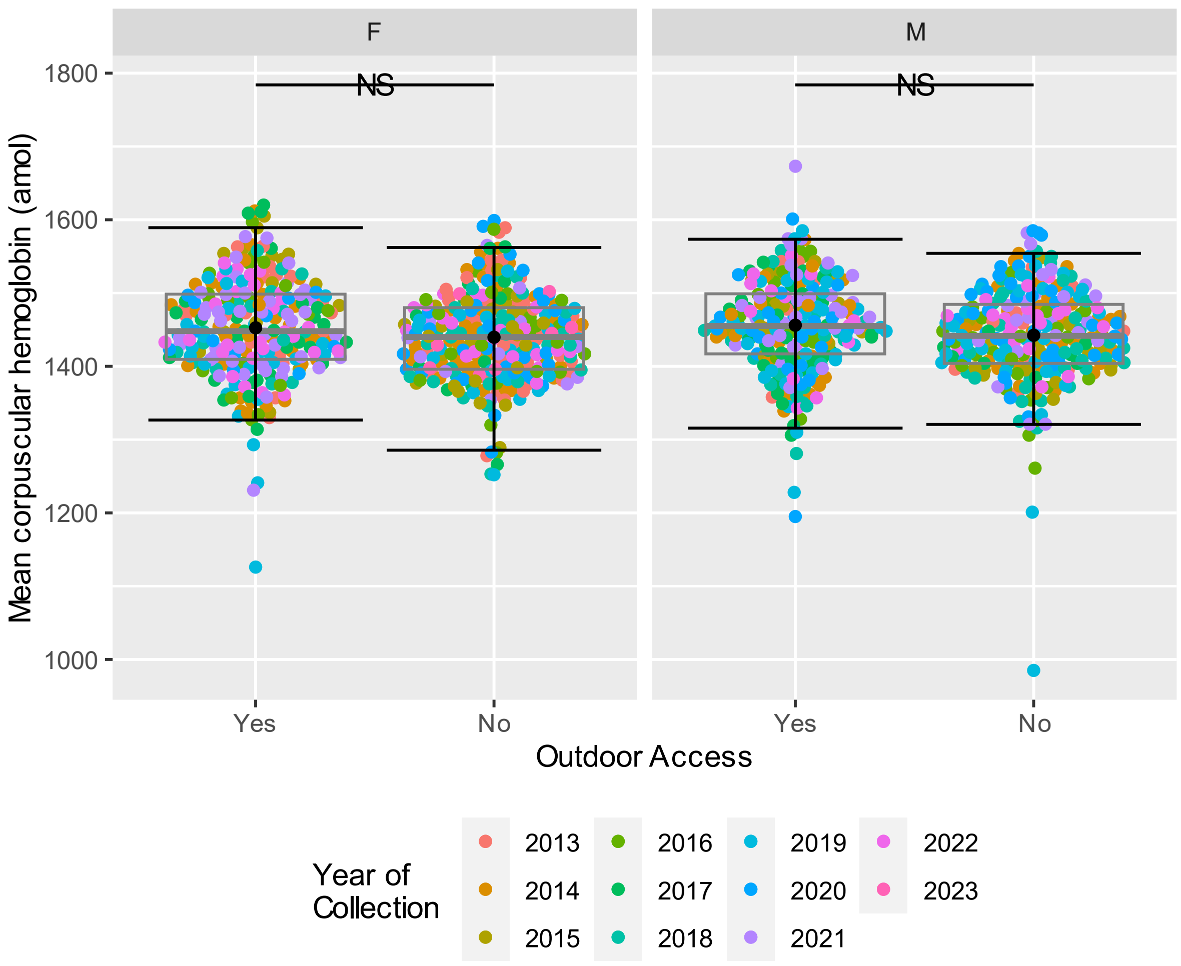

Figure S3 24. Mean Corpescular Haemoglobin according to outdoor access in adult common marmosets. Boxes indicate median and quartile ranges; black point indicates arithmetic mean and error bars indicate the normal range. Symbol colours indicate year of collection; NS = not significant, asterisks indicate statistical significance levels * < 0.001, ** < 0.0001, *** < 0.00001, **** < 0.000001

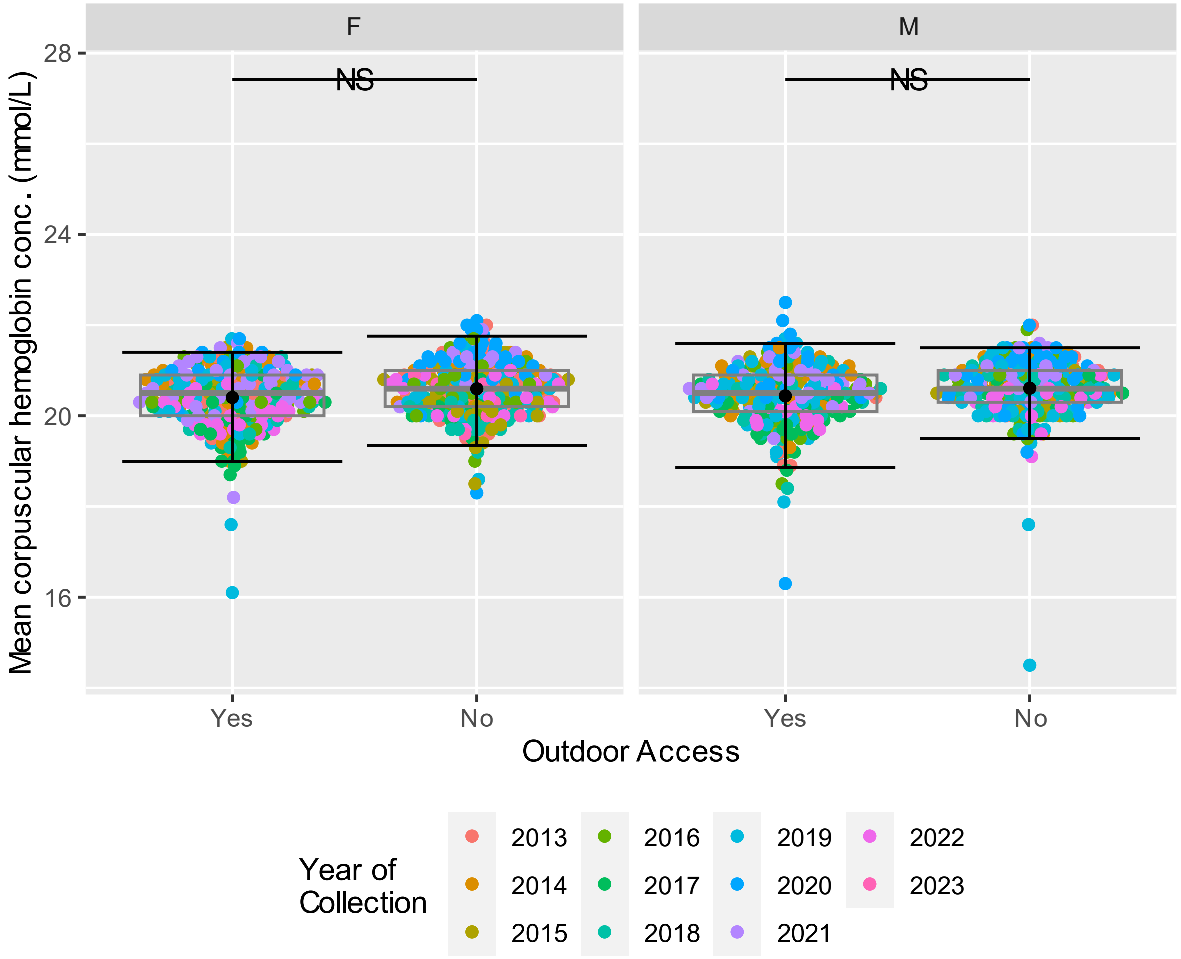

Figure S3 25. Mean Corpescular Haemoglobin Concentration according to outdoor access in adult common marmosets. Boxes indicate median and quartile ranges; black point indicates arithmetic mean and error bars indicate the normal range. Symbol colours indicate year of collection; NS = not significant, asterisks indicate statistical significance levels * < 0.001, ** < 0.0001, *** < 0.00001, **** < 0.000001

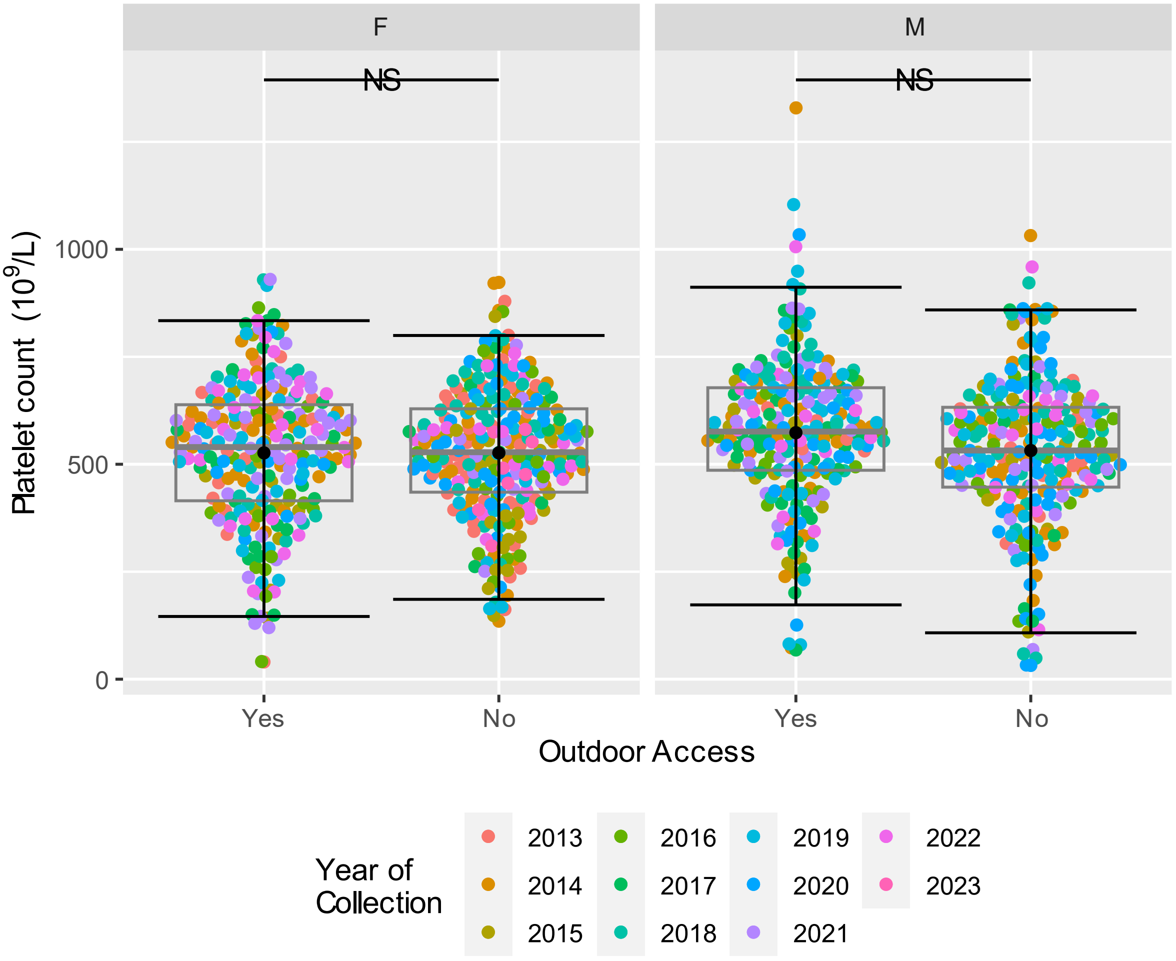

Figure S3 26. Platelet Count according to outdoor access in adult common marmosets. Boxes indicate median and quartile ranges; black point indicates arithmetic mean and error bars indicate the normal range. Symbol colours indicate year of collection; NS = not significant, asterisks indicate statistical significance levels * < 0.001, ** < 0.0001, *** < 0.00001, **** < 0.000001

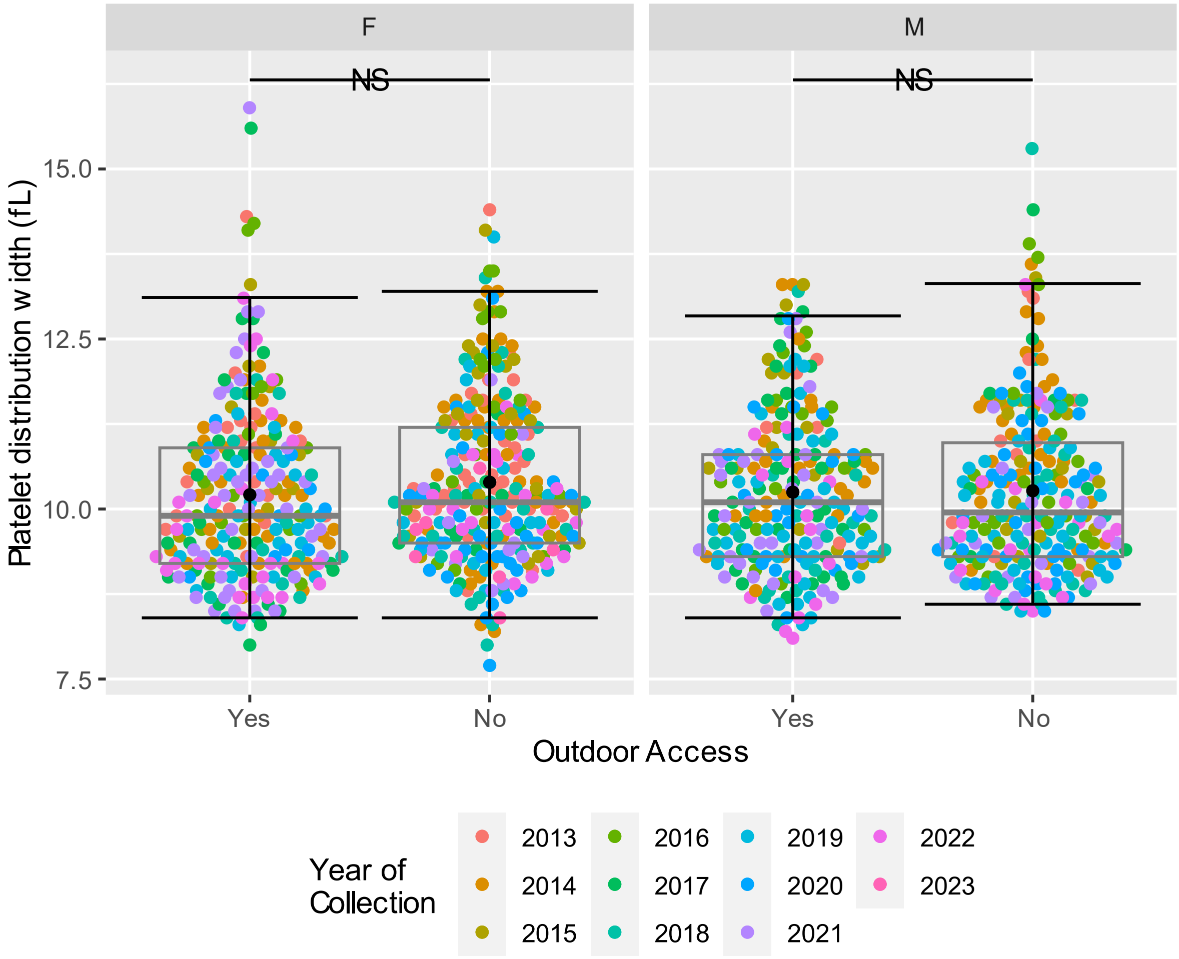

Figure S3 27. Platelet distribution width according to outdoor access in adult common marmosets. Boxes indicate median and quartile ranges; black point indicates arithmetic mean and error bars indicate the normal range. Symbol colours indicate year of collection; NS = not significant, asterisks indicate statistical significance levels * < 0.001, ** < 0.0001, *** < 0.00001, **** < 0.000001

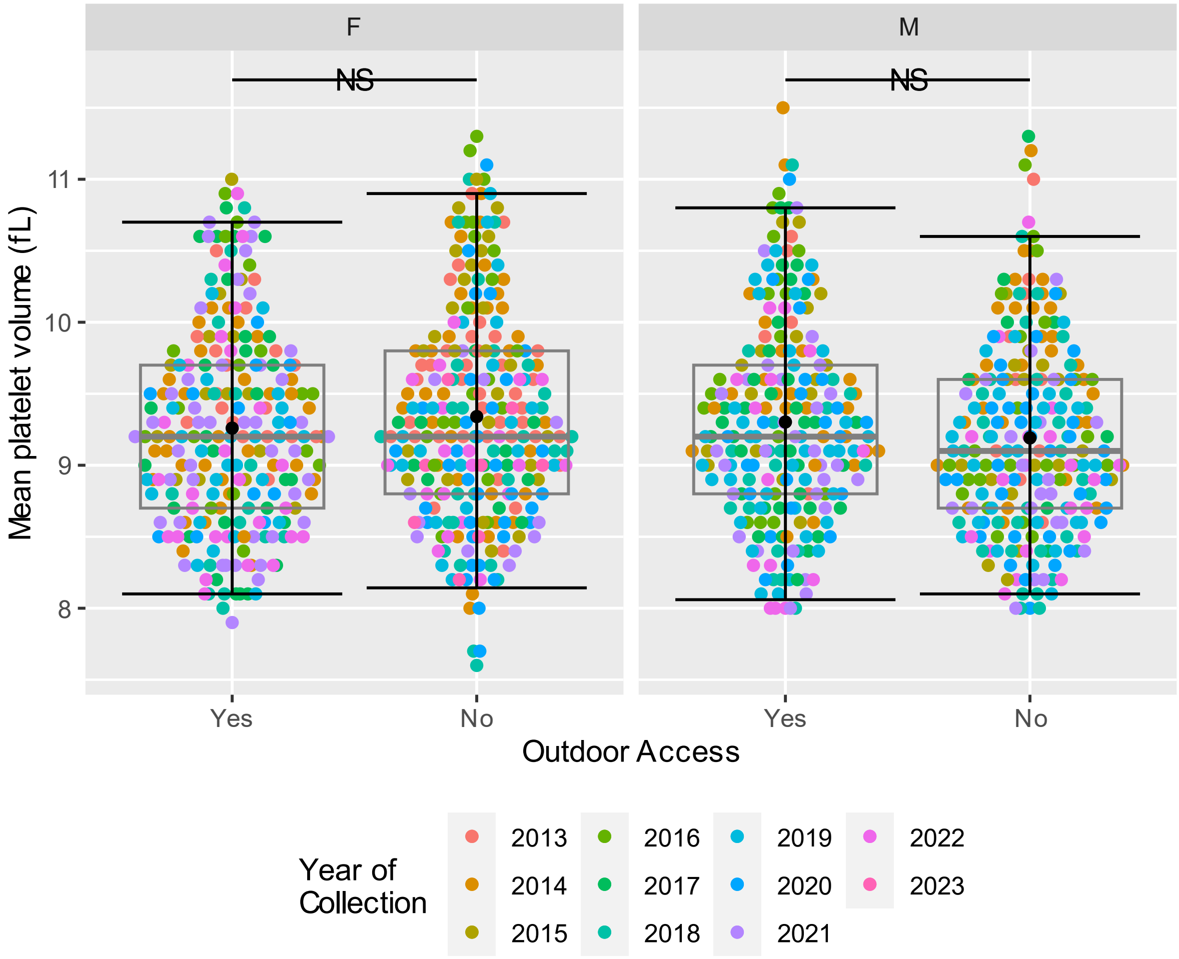

Figure S3 28. Mean Platelet Volume according to outdoor access in adult common marmosets. Boxes indicate median and quartile ranges; black point indicates arithmetic mean and error bars indicate the normal range. Symbol colours indicate year of collection; NS = not significant, asterisks indicate statistical significance levels * < 0.001, ** < 0.0001, *** < 0.00001, **** < 0.000001

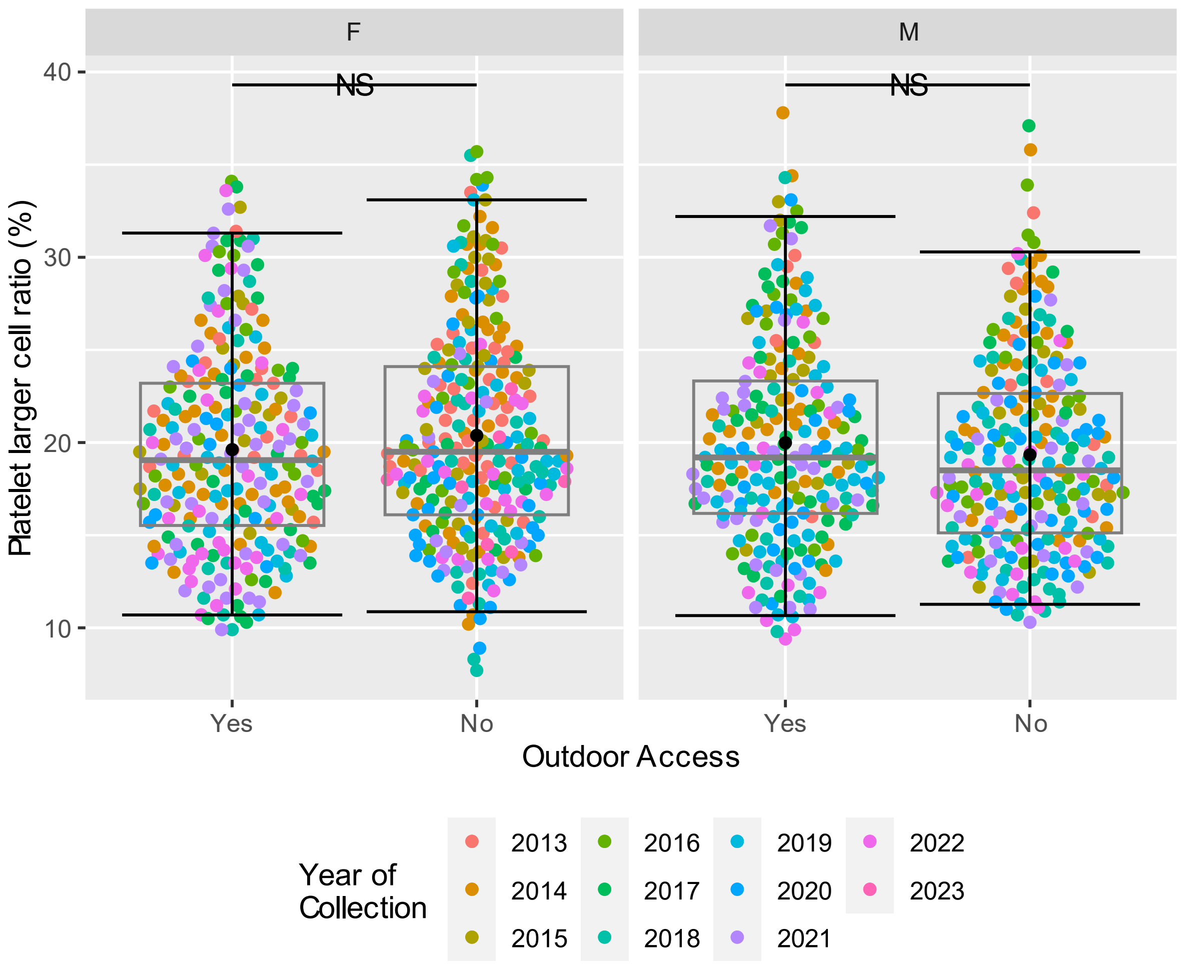

Figure S3 29. Platelet Larger Cell Ratio according to outdoor access in adult common marmosets. Boxes indicate median and quartile ranges; black point indicates arithmetic mean and error bars indicate the normal range. Symbol colours indicate year of collection; NS = not significant, asterisks indicate statistical significance levels * < 0.001, ** < 0.0001, *** < 0.00001, **** < 0.000001

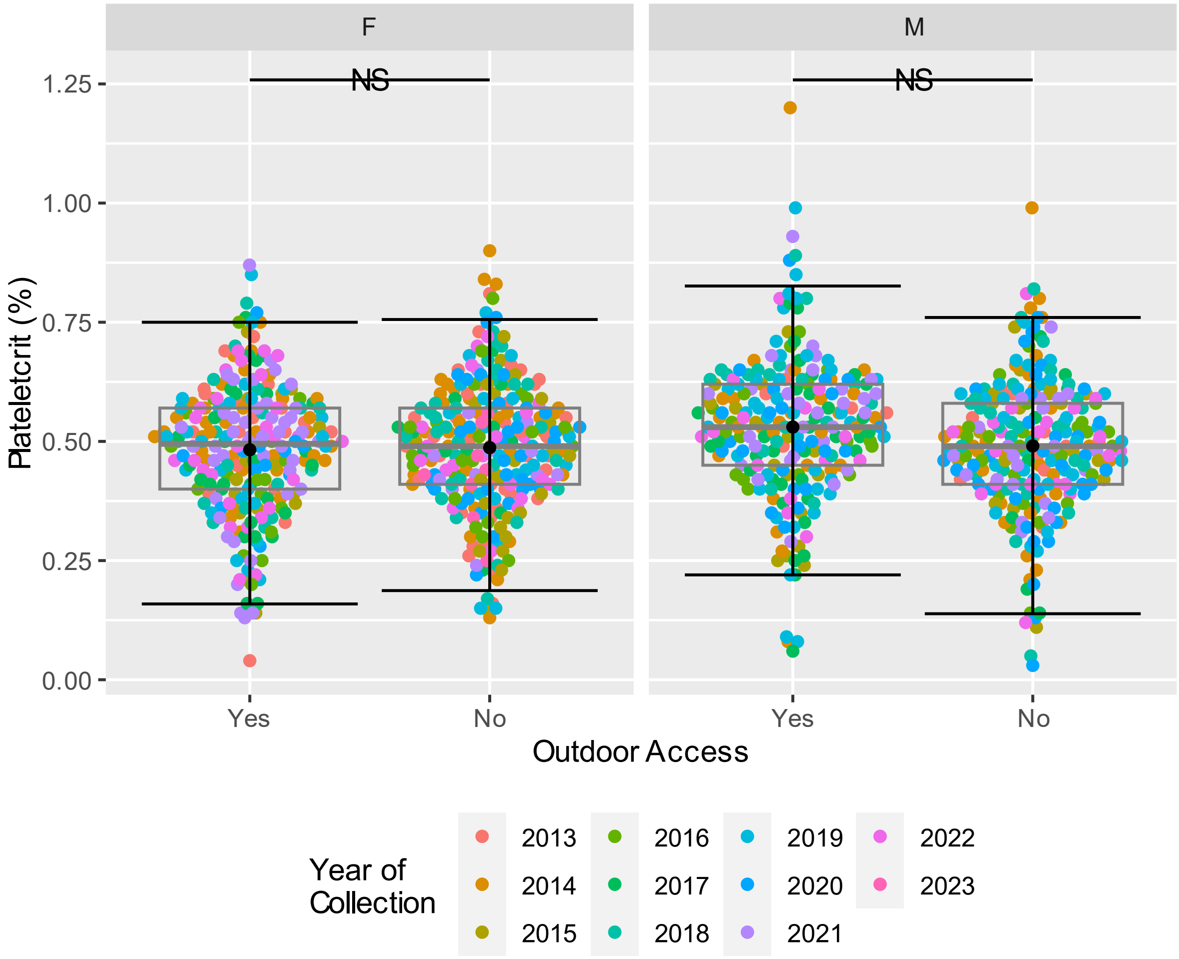

Figure S3 30. Plateletcrit according to outdoor access in adult common marmosets. Boxes indicate median and quartile ranges; black point indicates arithmetic mean and error bars indicate the normal range. Symbol colours indicate year of collection; NS = not significant, asterisks indicate statistical significance levels * < 0.001, ** < 0.0001, *** < 0.00001, **** < 0.000001

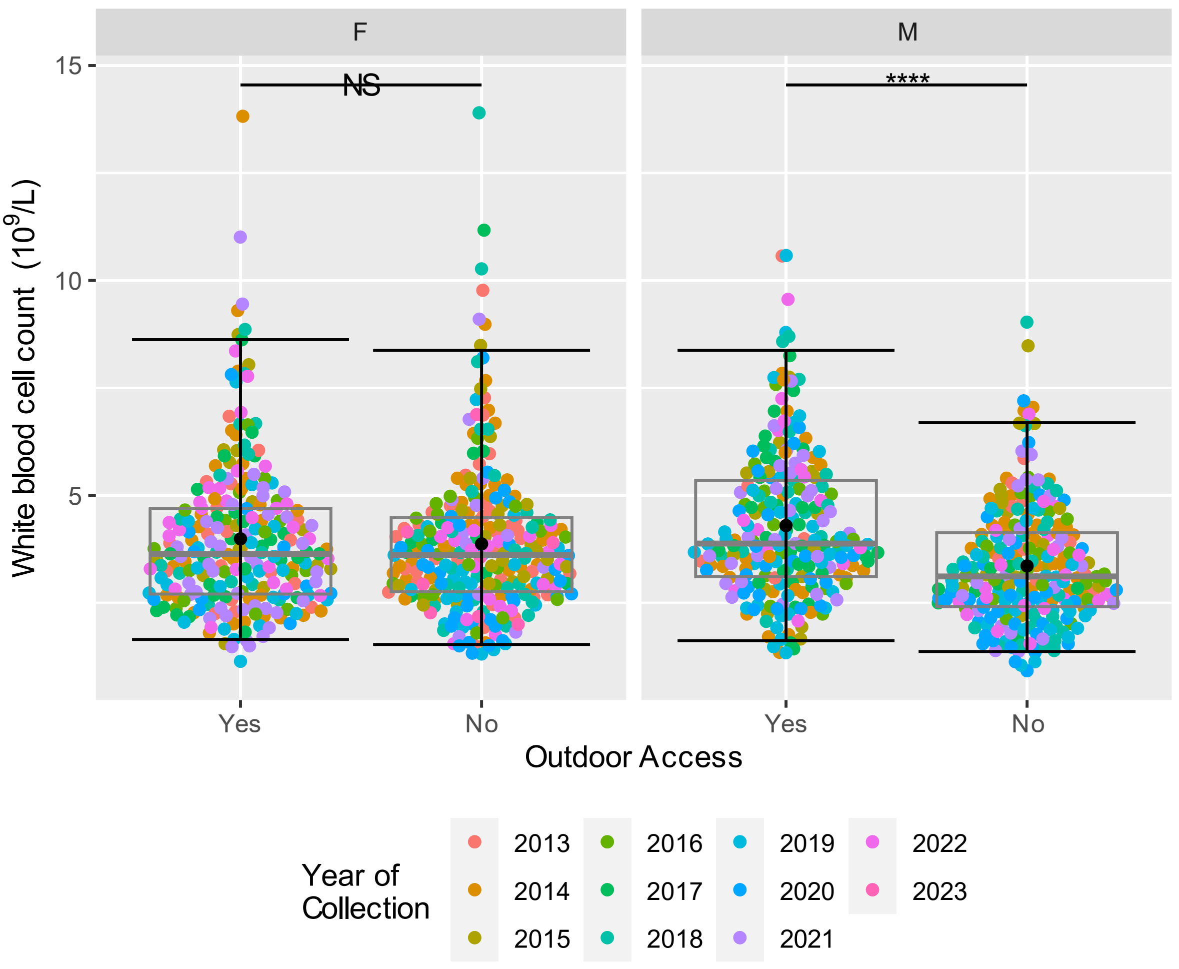

Figure S3 31. White Blood Cell count according to outdoor access in adult common marmosets. Boxes indicate median and quartile ranges; black point indicates arithmetic mean and error bars indicate the normal range. Symbol colours indicate year of collection; NS = not significant, asterisks indicate statistical significance levels * < 0.001, ** < 0.0001, *** < 0.00001, **** < 0.000001

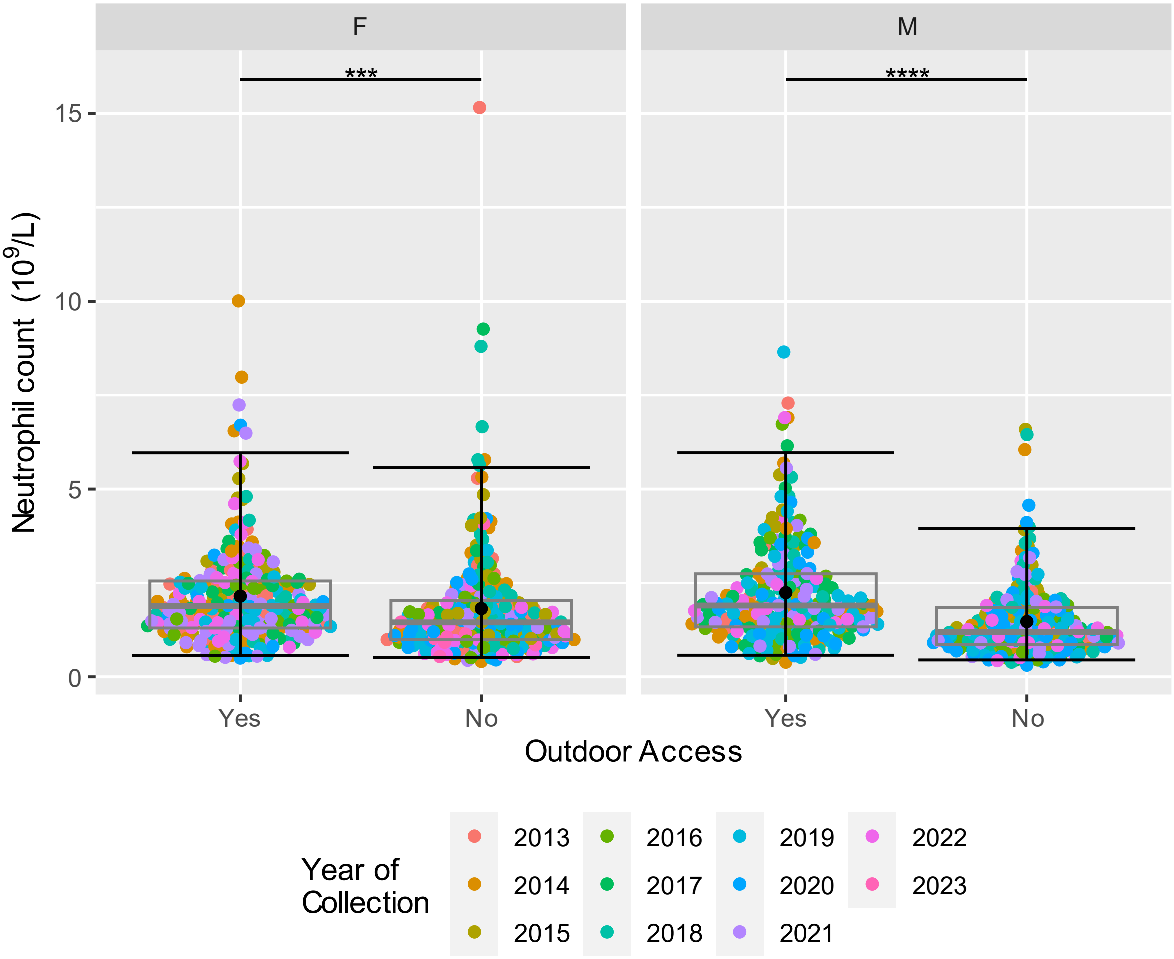

Figure S3 32. Neutrophil count according to outdoor access in adult common marmosets. Boxes indicate median and quartile ranges; black point indicates arithmetic mean and error bars indicate the normal range. Symbol colours indicate year of collection; NS = not significant, asterisks indicate statistical significance levels * < 0.001, ** < 0.0001, *** < 0.00001, **** < 0.000001

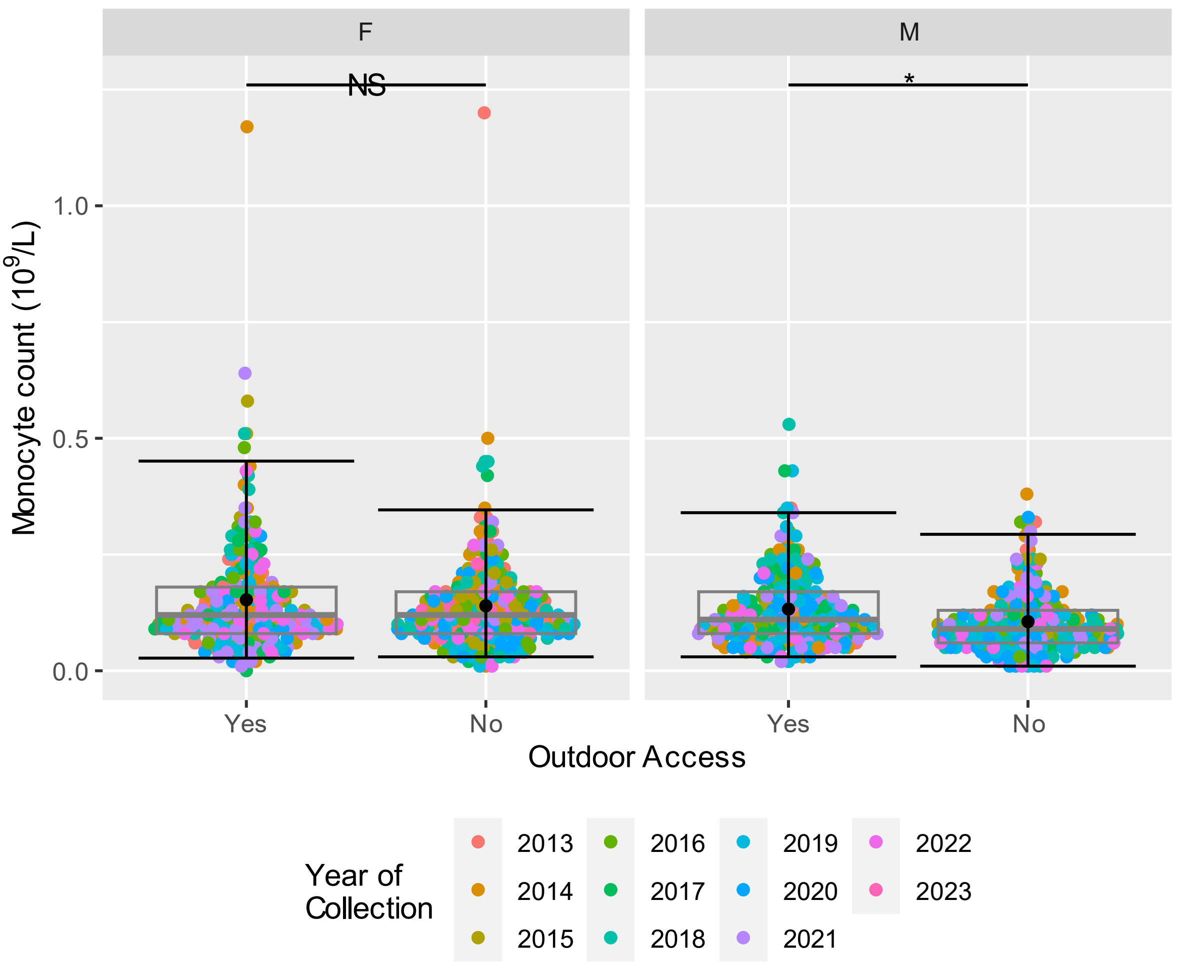

Figure S3 33. Monocyte count according to outdoor access in adult common marmosets. Boxes indicate median and quartile ranges; black point indicates arithmetic mean and error bars indicate the normal range. Symbol colours indicate year of collection; NS = not significant, asterisks indicate statistical significance levels * < 0.001, ** < 0.0001, *** < 0.00001, **** < 0.000001

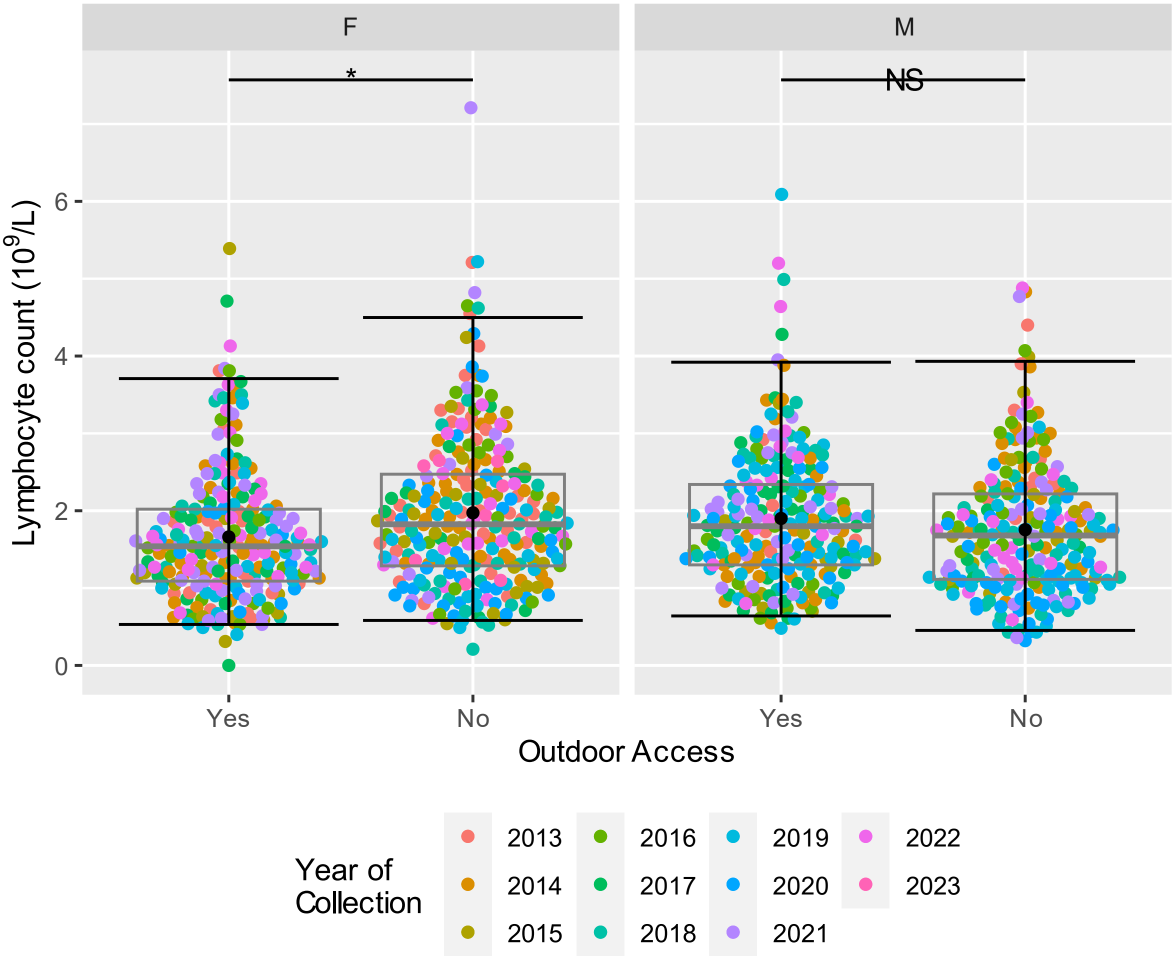

Figure S3 34. Lymphocyte count according to outdoor access in adult common marmosets. Boxes indicate median and quartile ranges; black point indicates arithmetic mean and error bars indicate the normal range. Symbol colours indicate year of collection; NS = not significant, asterisks indicate statistical significance levels * < 0.001, ** < 0.0001, *** < 0.00001, **** < 0.000001

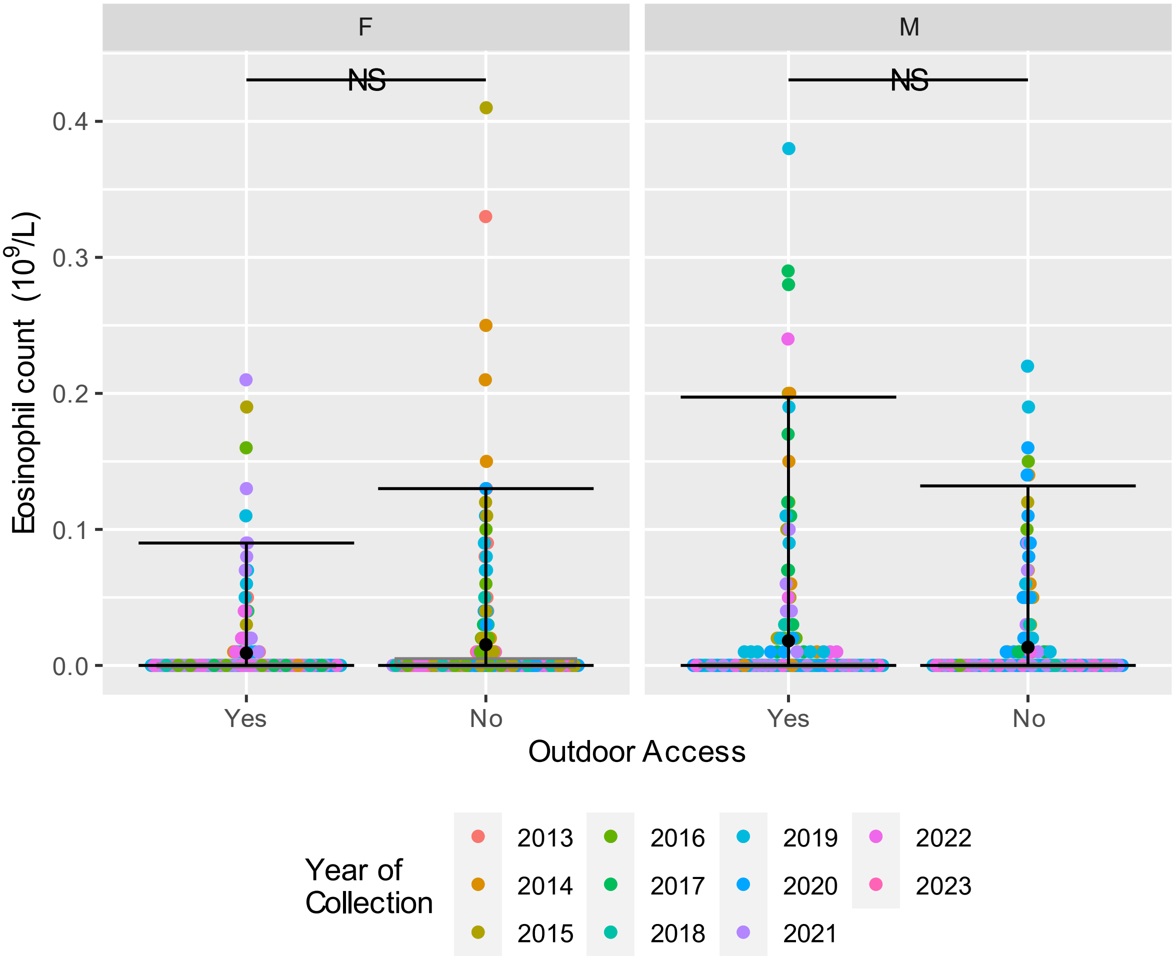

Figure S3 35. Eosinophil count according to outdoor access in adult common marmosets. Boxes indicate median and quartile ranges; black point indicates arithmetic mean and error bars indicate the normal range. Symbol colours indicate year of collection; NS = not significant, asterisks indicate statistical significance levels * < 0.001, ** < 0.0001, *** < 0.00001, **** < 0.000001

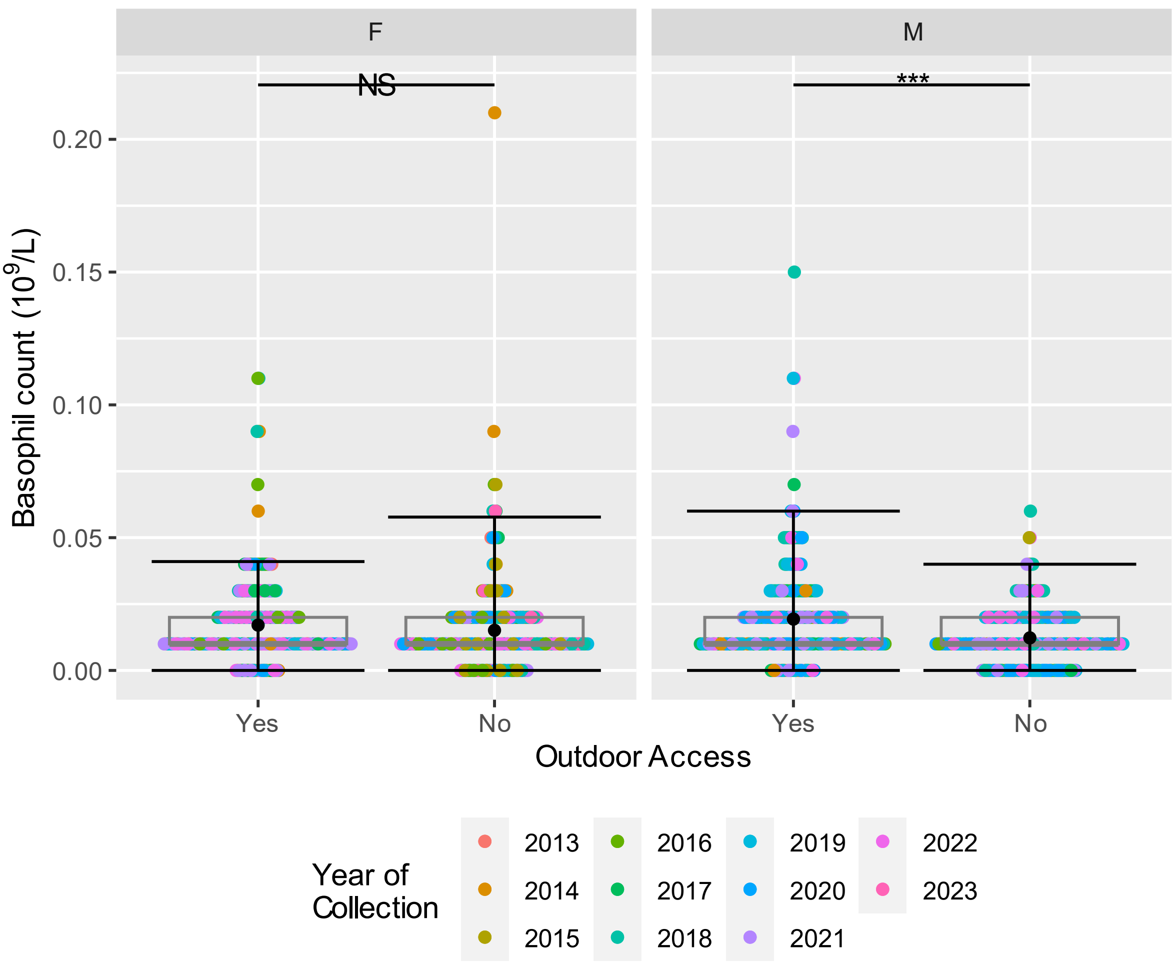

Figure S3 36. Basophil count according to outdoor access in adult common marmosets. Boxes indicate median and quartile ranges; black point indicates arithmetic mean and error bars indicate the normal range. Symbol colours indicate year of collection; NS = not significant, asterisks indicate statistical significance levels * < 0.001, ** < 0.0001, *** < 0.00001, **** < 0.000001
