# Supplementary material for: Hematological and Serum Biochemical Reference Intervals for Alphaxalone Sedated Common Marmosets (Callithrix jacchus)
Source: Animals (Basel). 2024 Mar 3;14(5):790. doi: 10.3390/ani14050790 (PMC10930924; doi:10.3390/ani14050790)
Supplement: Supplementary file 1 [file animals-14-00790-s001.zip › Figure S4.pdf]

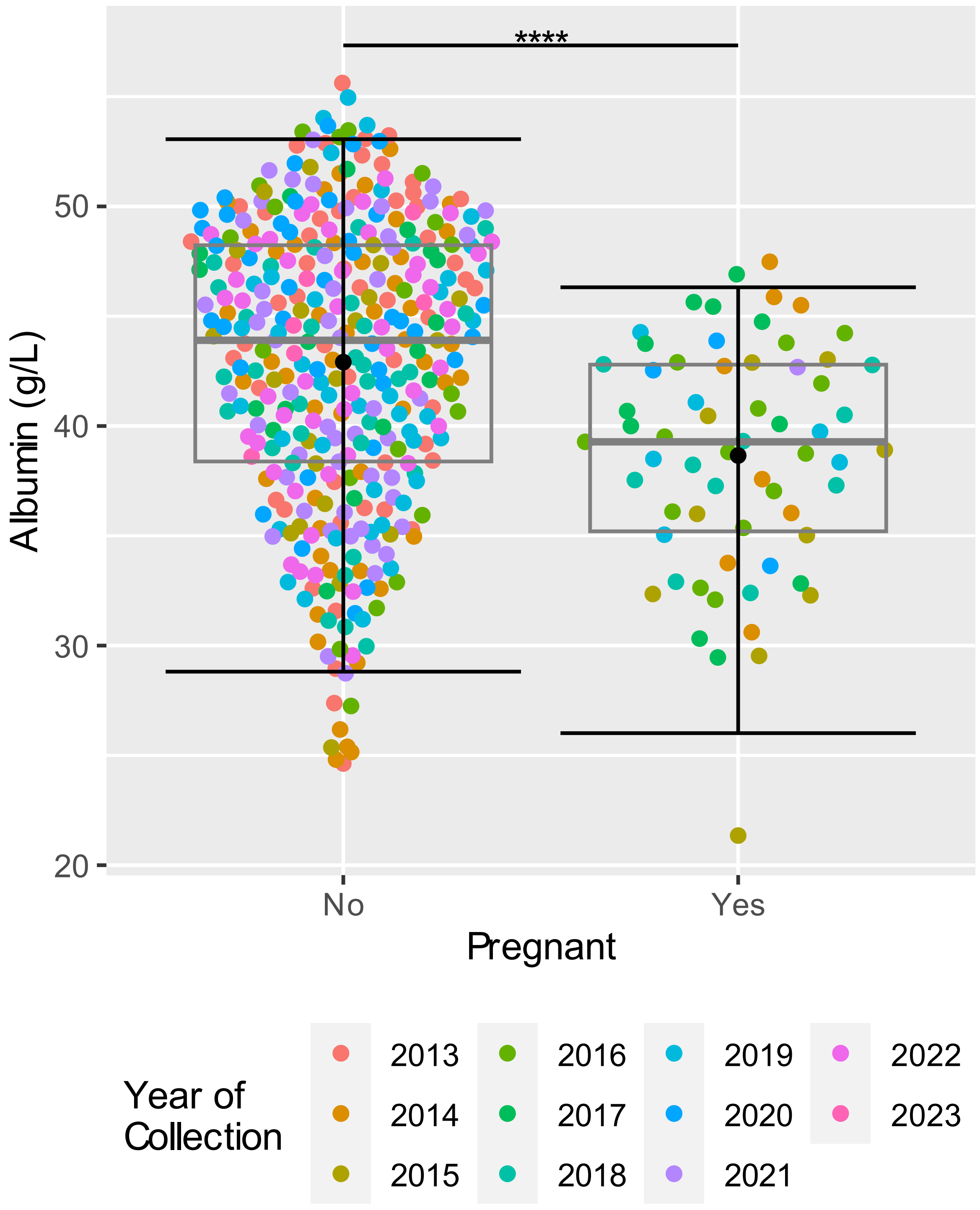

Figure S4 1. Albumin levels according to pregnancy status in adult female common marmosets. Boxes indicate median and quartile ranges; black point indicates arithmetic mean and error bars indicate the normal range. Symbol colours indicate year of collection; NS = not significant, NS = not significant, asterisks indicate statistical significance levels * < 0.001, ** < 0.0001, *** < 0.00001, **** < 0.000001

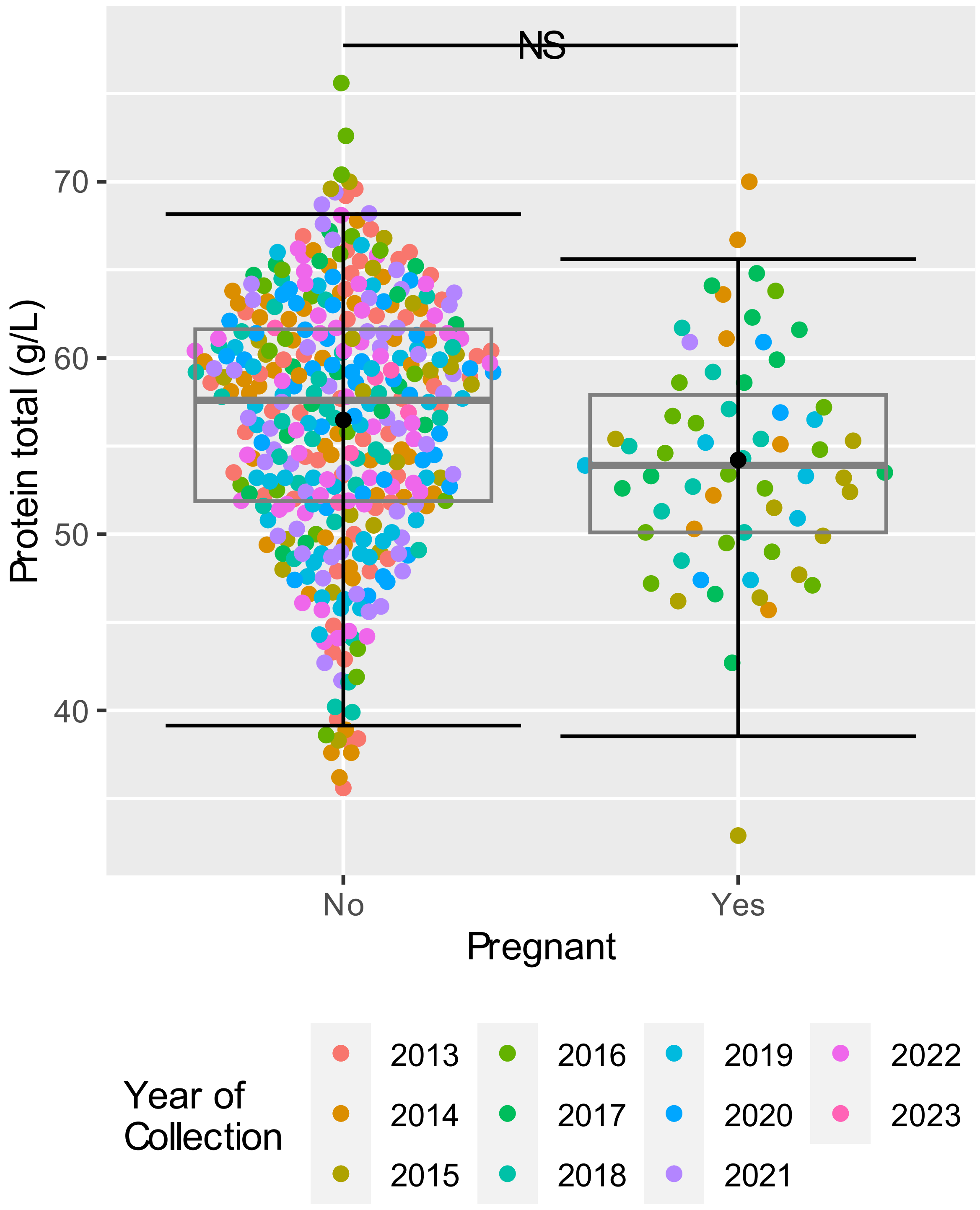

Figure S4 2. Total Protein levels according to pregnancy status in adult female common marmosets. Boxes indicate median and quartile ranges; black point indicates arithmetic mean and error bars indicate the normal range. Symbol colours indicate year of collection; NS = not significant, NS = not significant, asterisks indicate statistical significance levels * < 0.001, ** < 0.0001, *** < 0.00001, **** < 0.000001

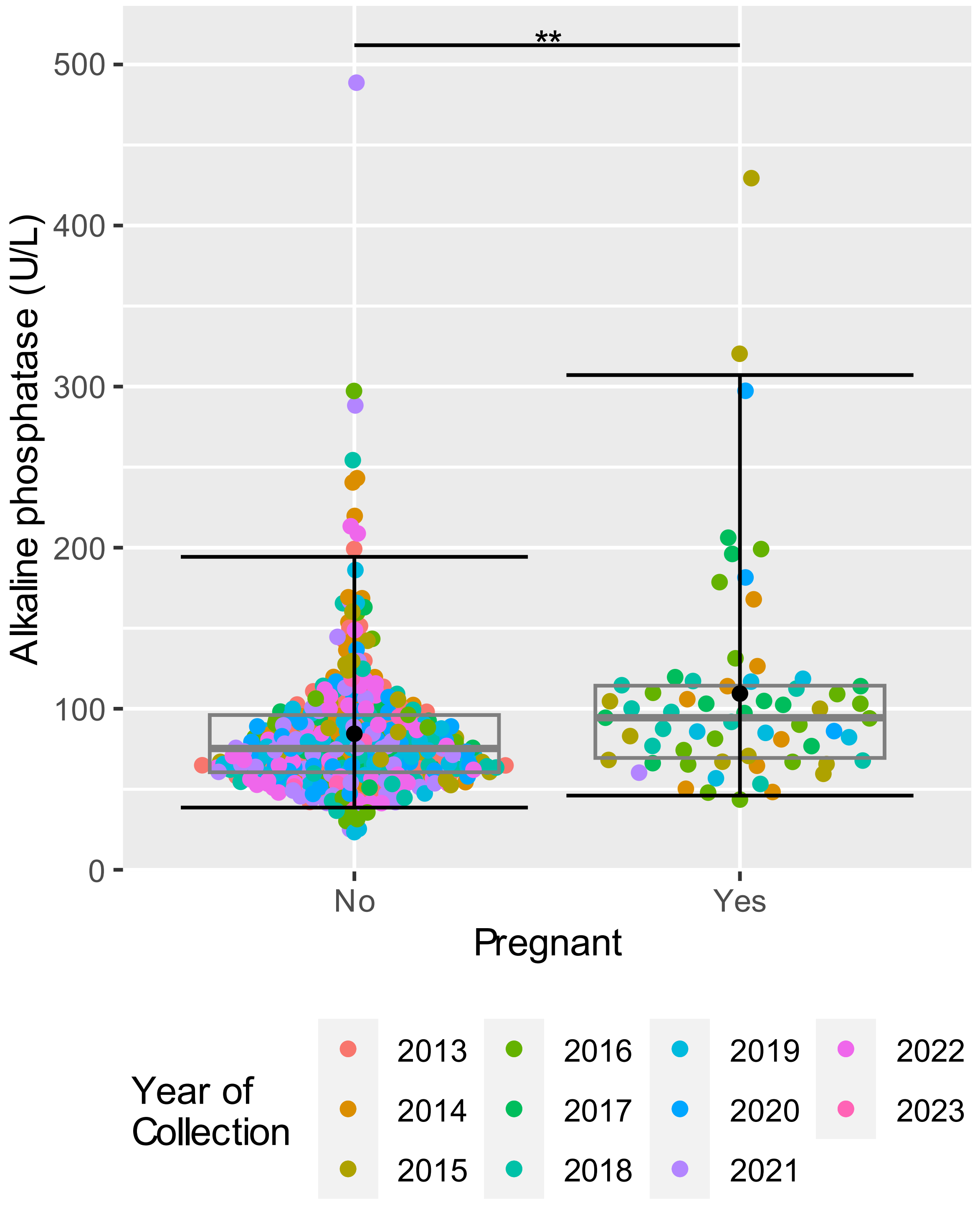

Figure S4 3. Alkaline Phosphatase levels according to pregnancy status in adult female common marmosets. Boxes indicate median and quartile ranges; black point indicates arithmetic mean and error bars indicate the normal range. Symbol colours indicate year of collection; NS = not significant, NS = not significant, asterisks indicate statistical significance levels * < 0.001, ** < 0.0001, *** < 0.00001, **** < 0.000001

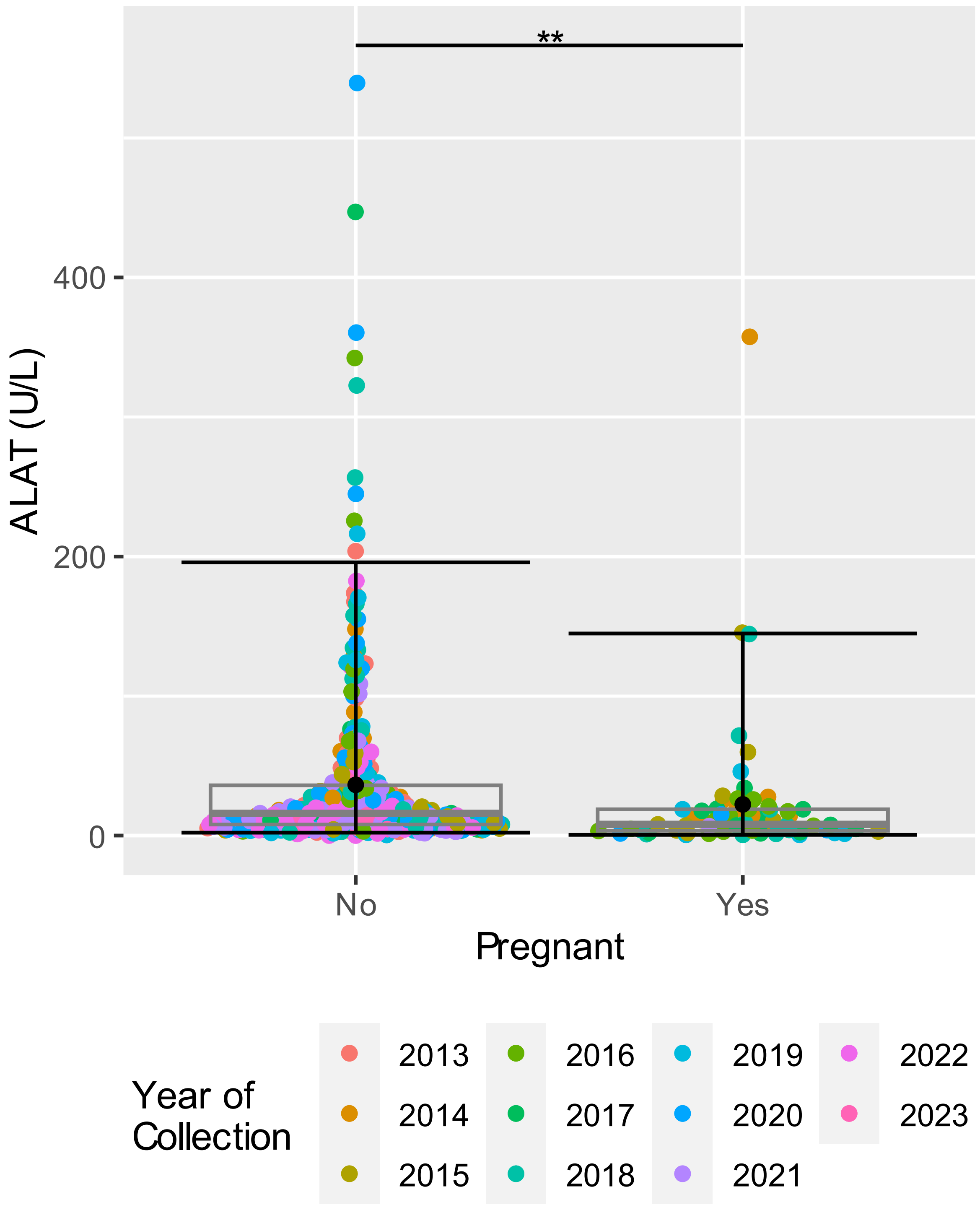

Figure S4 4. ALAT levels according to pregnancy status in adult female common marmosets. Boxes indicate median and quartile ranges; black point indicates arithmetic mean and error bars indicate the normal range. Symbol colours indicate year of collection; NS = not significant, NS = not significant, asterisks indicate statistical significance levels * < 0.001, ** < 0.0001, *** < 0.00001, **** < 0.000001

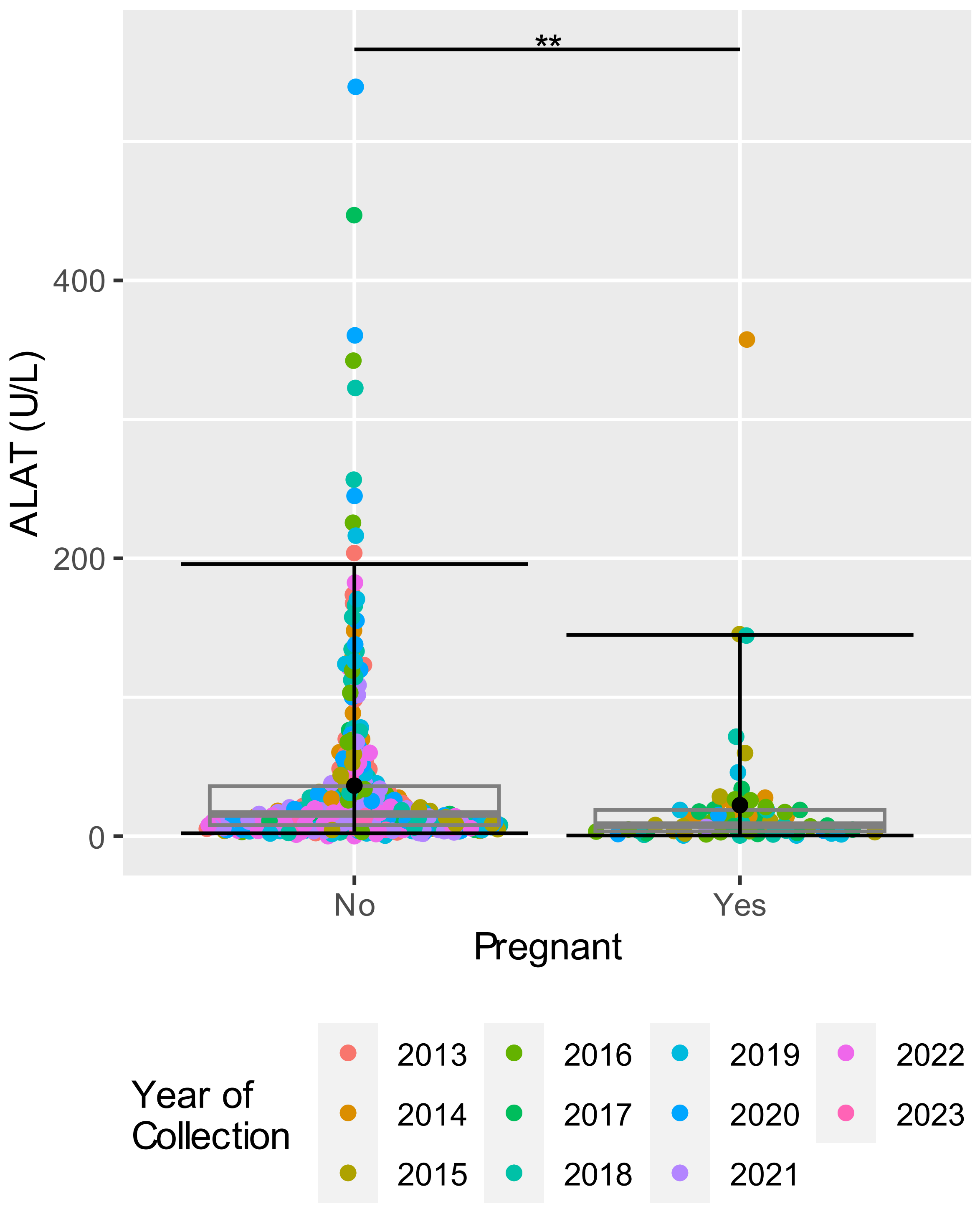

Figure S4 5. ASAT levels according to pregnancy status in adult female common marmosets. Boxes indicate median and quartile ranges; black point indicates arithmetic mean and error bars indicate the normal range. Symbol colours indicate year of collection; NS = not significant, NS = not significant, asterisks indicate statistical significance levels * < 0.001, ** < 0.0001, *** < 0.00001, **** < 0.000001

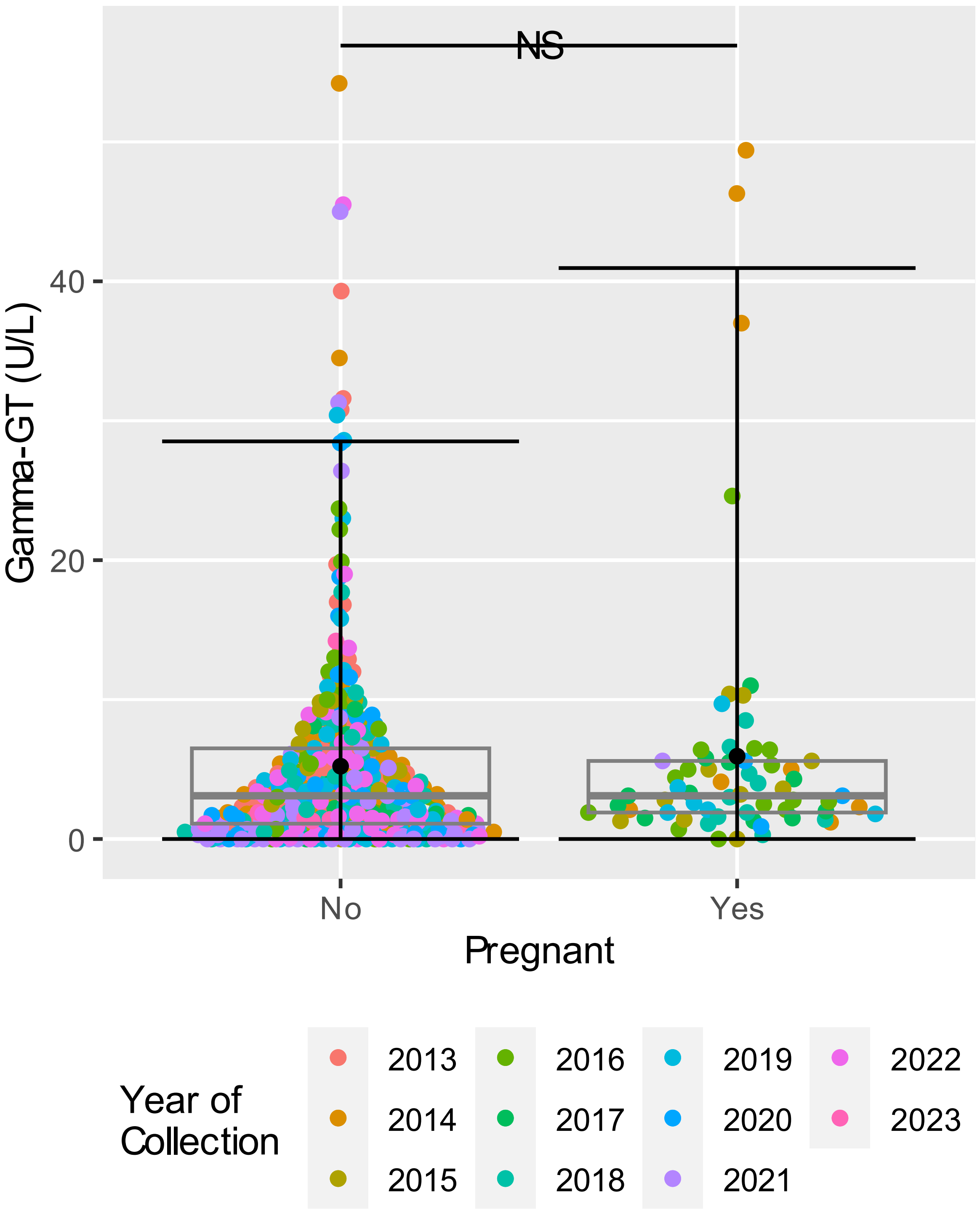

Figure S4 6. Gamma-GT levels according to pregnancy status in adult female common marmosets. Boxes indicate median and quartile ranges; black point indicates arithmetic mean and error bars indicate the normal range. Symbol colours indicate year of collection; NS = not significant, NS = not significant, asterisks indicate statistical significance levels * < 0.001, ** < 0.0001, *** < 0.00001, **** < 0.000001

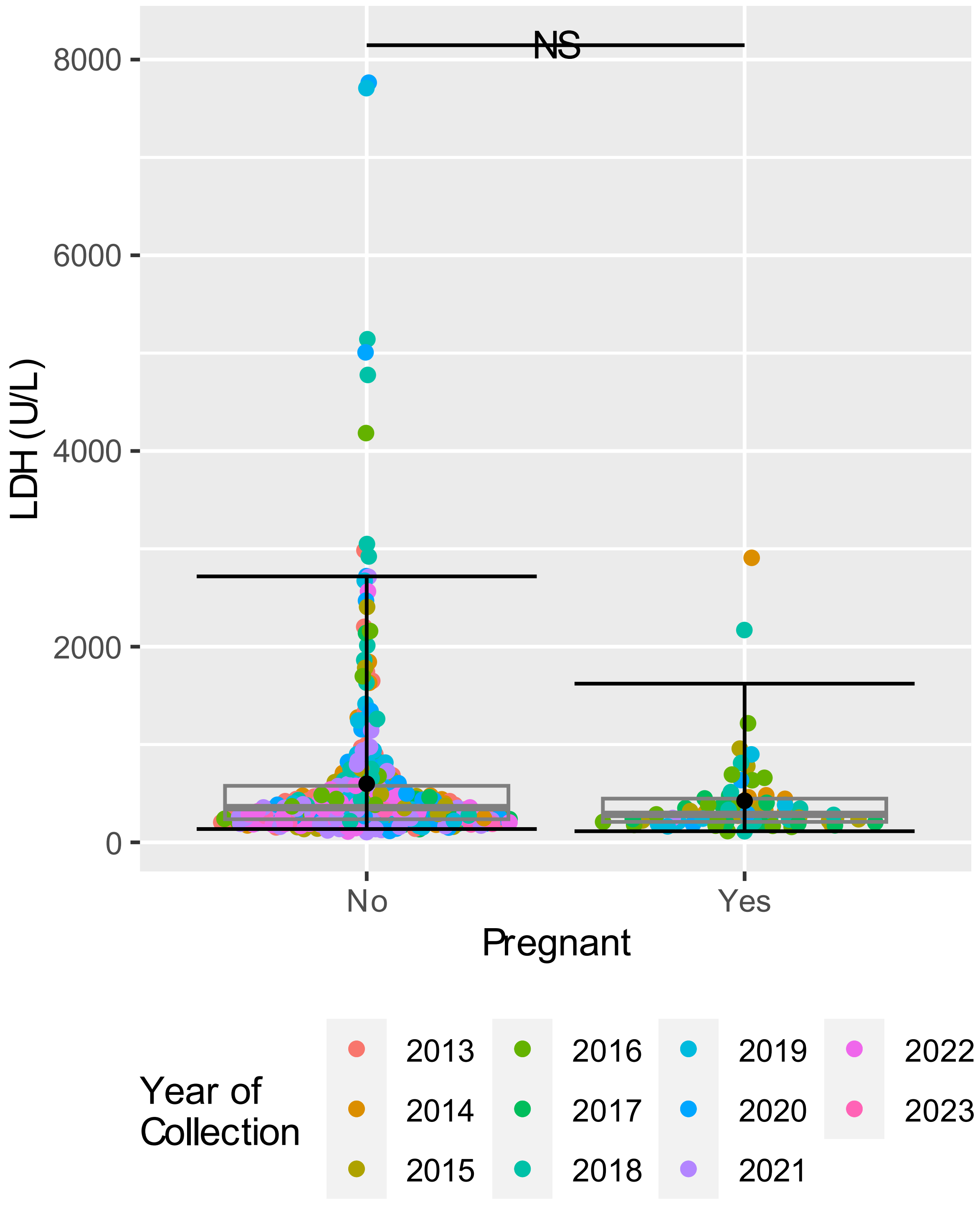

Figure S4 7. LDH levels according to pregnancy status in adult female common marmosets. Boxes indicate median and quartile ranges; black point indicates arithmetic mean and error bars indicate the normal range. Symbol colours indicate year of collection; NS = not significant, asterisks indicate statistical significance levels * < 0.001, ** < 0.0001, *** < 0.00001, **** < 0.000001

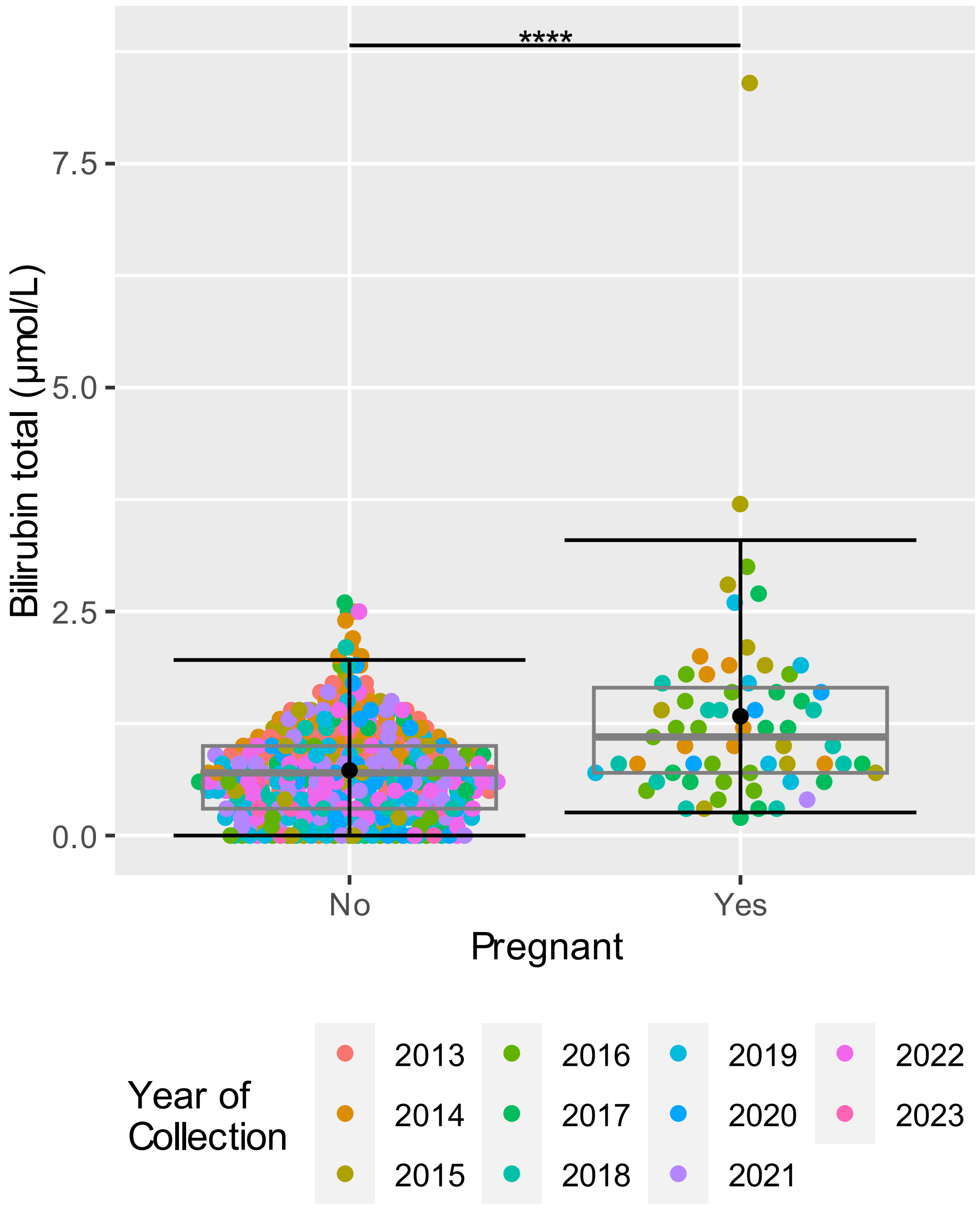

Figure S4 8. Total Bilirubin levels according to pregnancy status in adult female common marmosets. Boxes indicate median and quartile ranges; black point indicates arithmetic mean and error bars indicate the normal range. Symbol colours indicate year of collection; NS = not significant, asterisks indicate statistical significance levels * < 0.001, ** < 0.0001, *** < 0.00001, **** < 0.000001

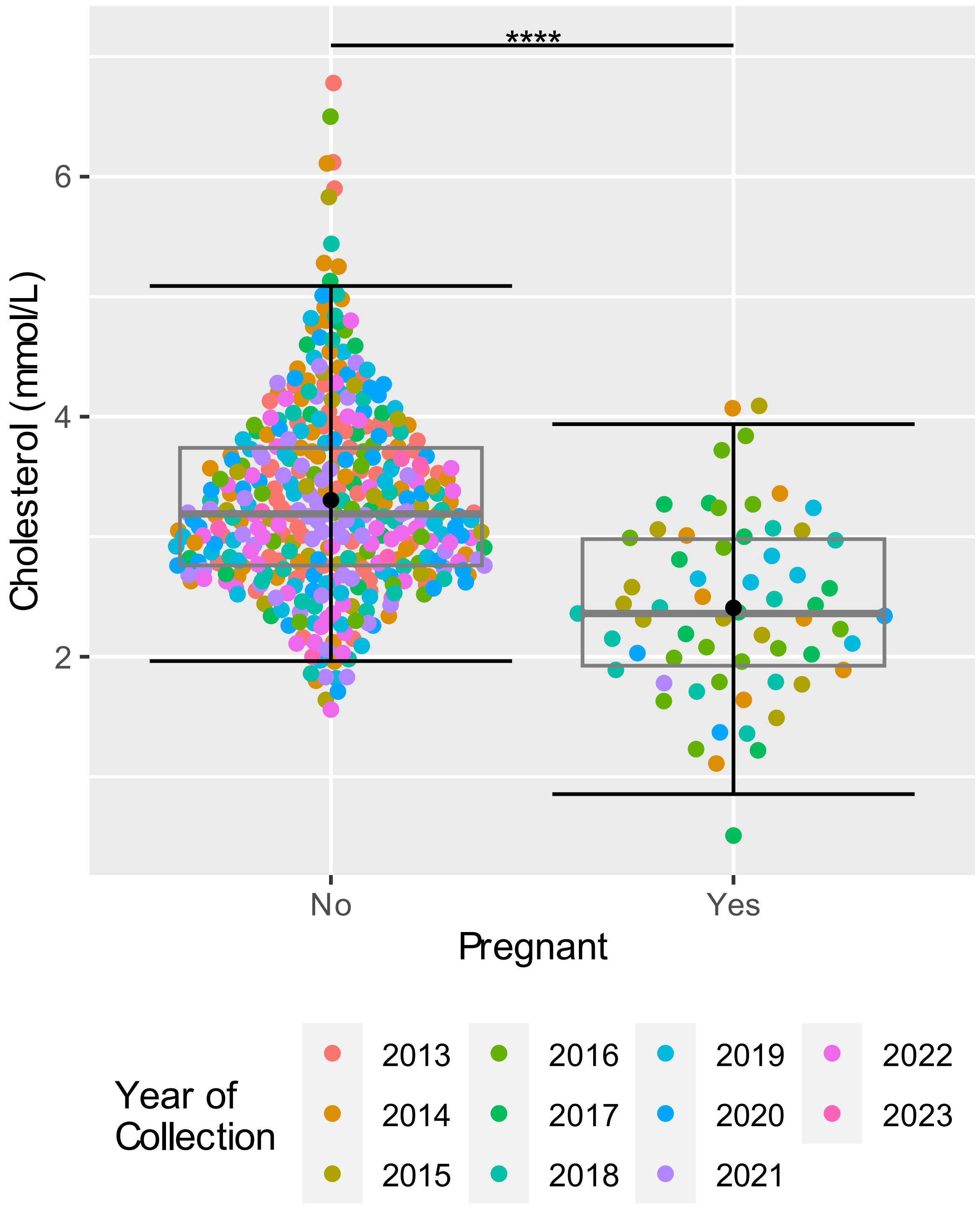

Figure S4 9. Cholesterol levels according to pregnancy status in adult female common marmosets. Boxes indicate median and quartile ranges; black point indicates arithmetic mean and error bars indicate the normal range. Symbol colours indicate year of collection; NS = not significant, asterisks indicate statistical significance levels * < 0.001, ** < 0.0001, *** < 0.00001, **** < 0.000001

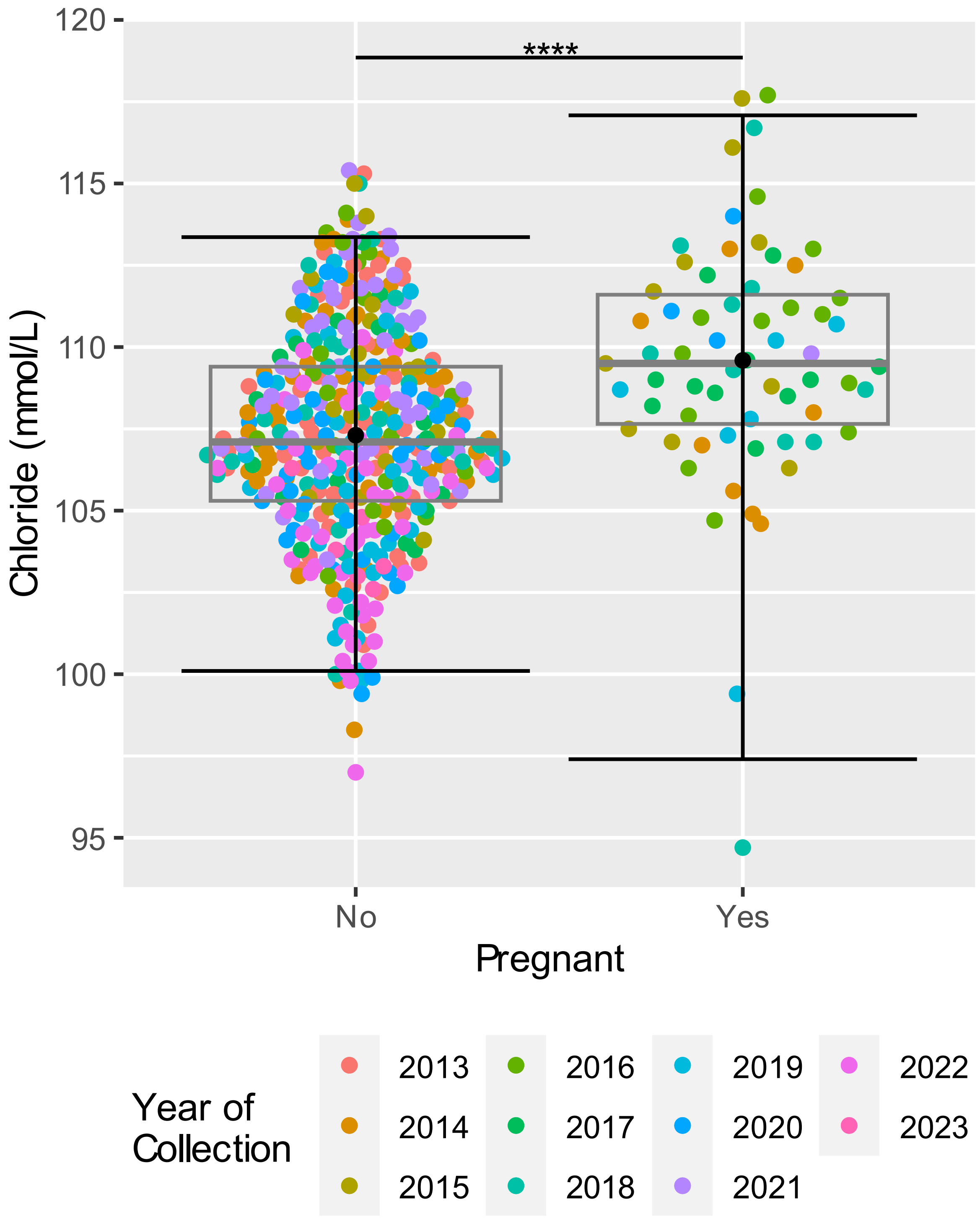

Figure S4 10. Chloride levels according to pregnancy status in adult female common marmosets. Boxes indicate median and quartile ranges; black point indicates arithmetic mean and error bars indicate the normal range. Symbol colours indicate year of collection; NS = not significant, asterisks indicate statistical significance levels * < 0.001, ** < 0.0001, *** < 0.00001, **** < 0.000001

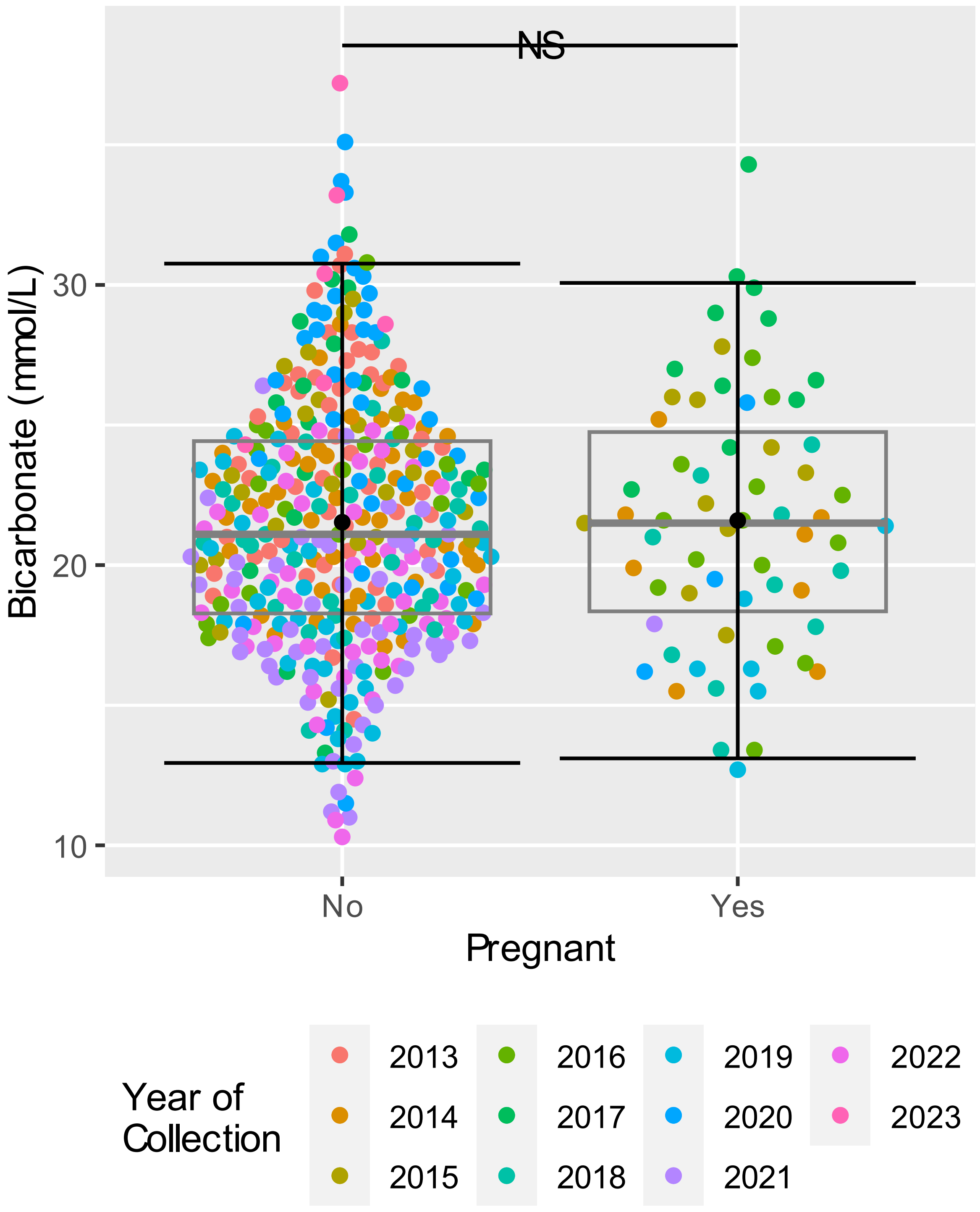

Figure S4 11. Bicarbonate levels according to pregnancy status in adult female common marmosets. Boxes indicate median and quartile ranges; black point indicates arithmetic mean and error bars indicate the normal range. Symbol colours indicate year of collection; NS = not significant, asterisks indicate statistical significance levels * < 0.001, ** < 0.0001, *** < 0.00001, **** < 0.000001

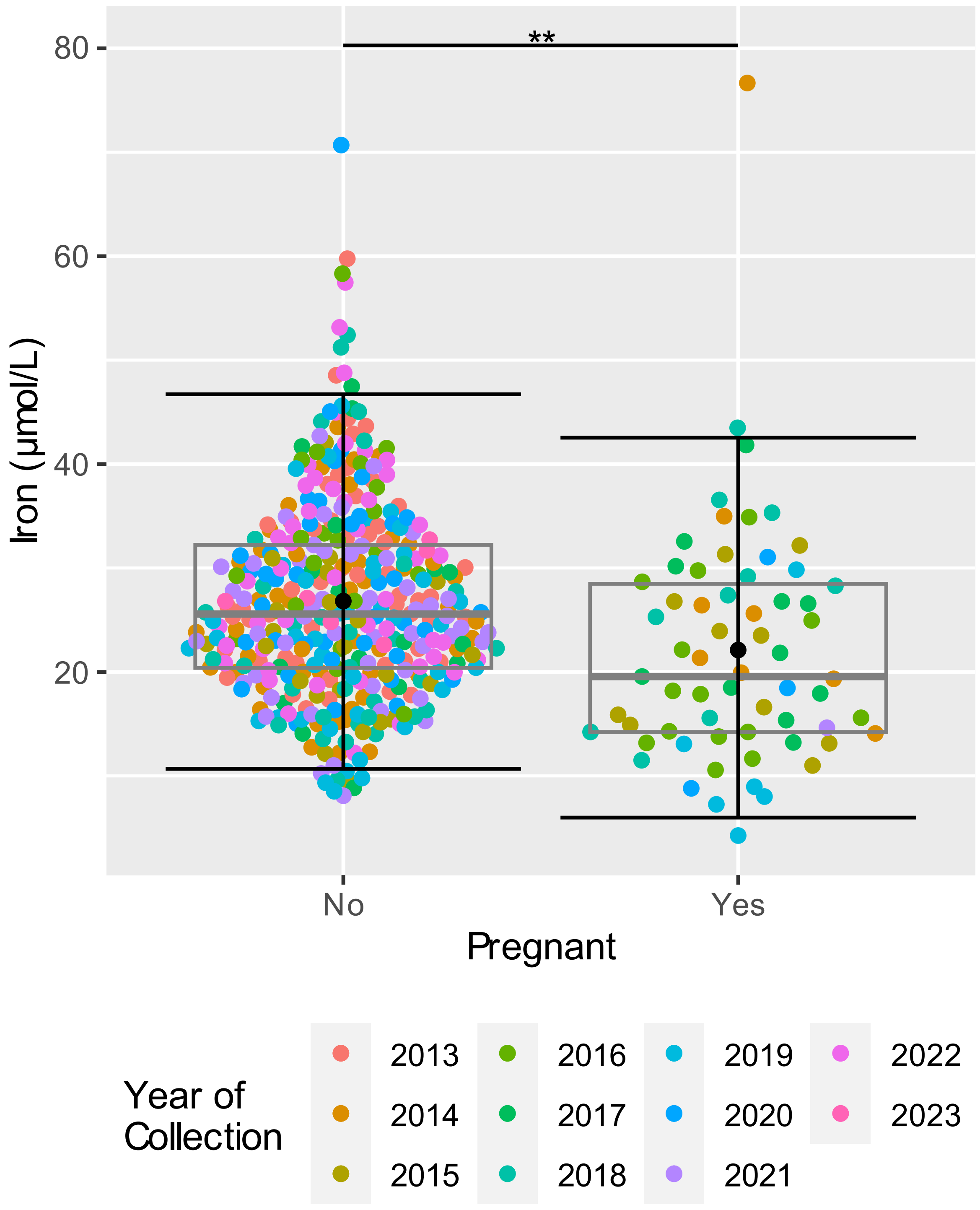

Figure S4 12. Iron levels according to pregnancy status in adult female common marmosets. Boxes indicate median and quartile ranges; black point indicates arithmetic mean and error bars indicate the normal range. Symbol colours indicate year of collection; NS = not significant, asterisks indicate statistical significance levels * < 0.001, ** < 0.0001, *** < 0.00001, **** < 0.000001

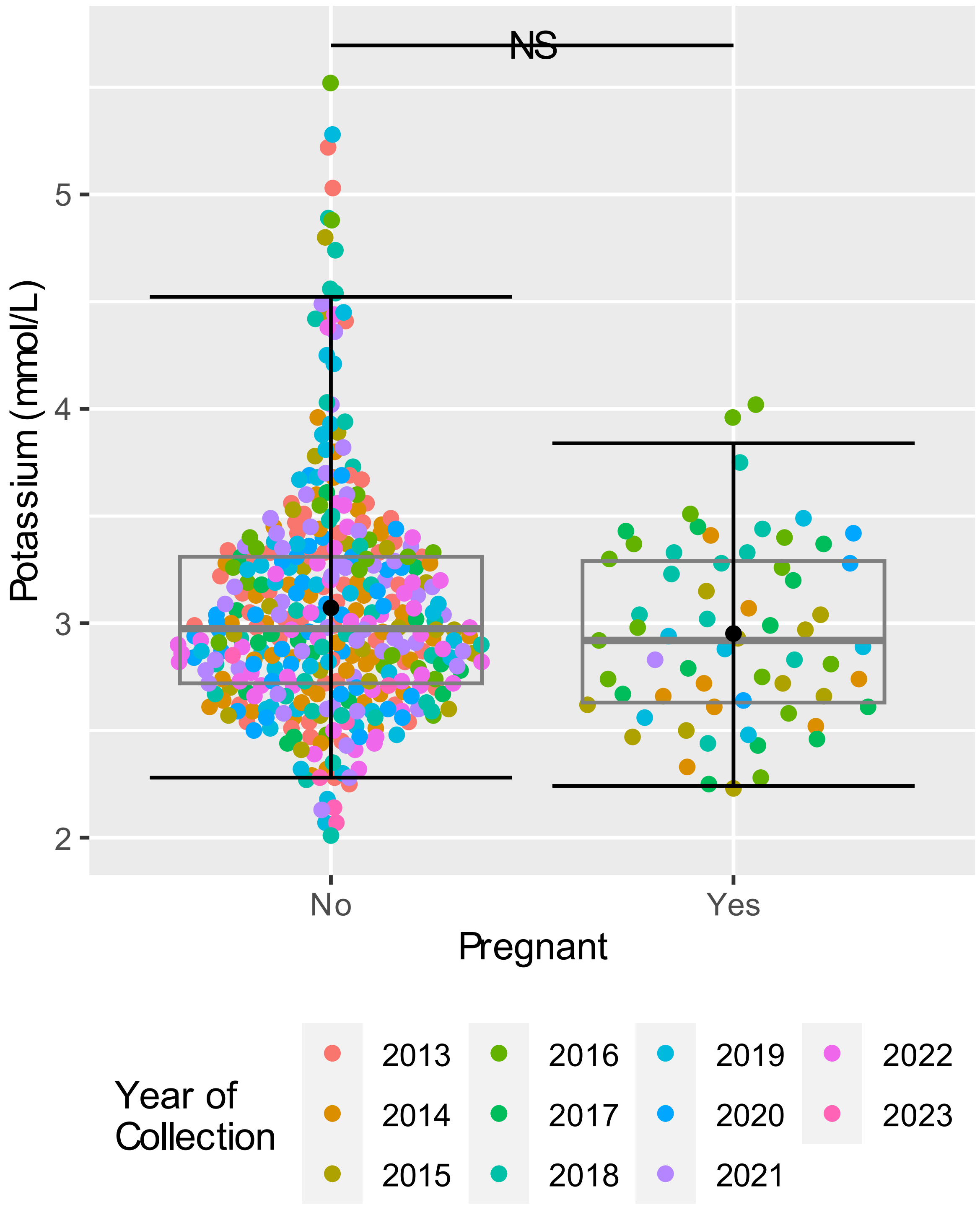

Figure S4 13. Potassium levels according to pregnancy status in adult female common marmosets. Boxes indicate median and quartile ranges; black point indicates arithmetic mean and error bars indicate the normal range. Symbol colours indicate year of collection; NS = not significant, asterisks indicate statistical significance levels * < 0.001, ** < 0.0001, *** < 0.00001, **** < 0.000001

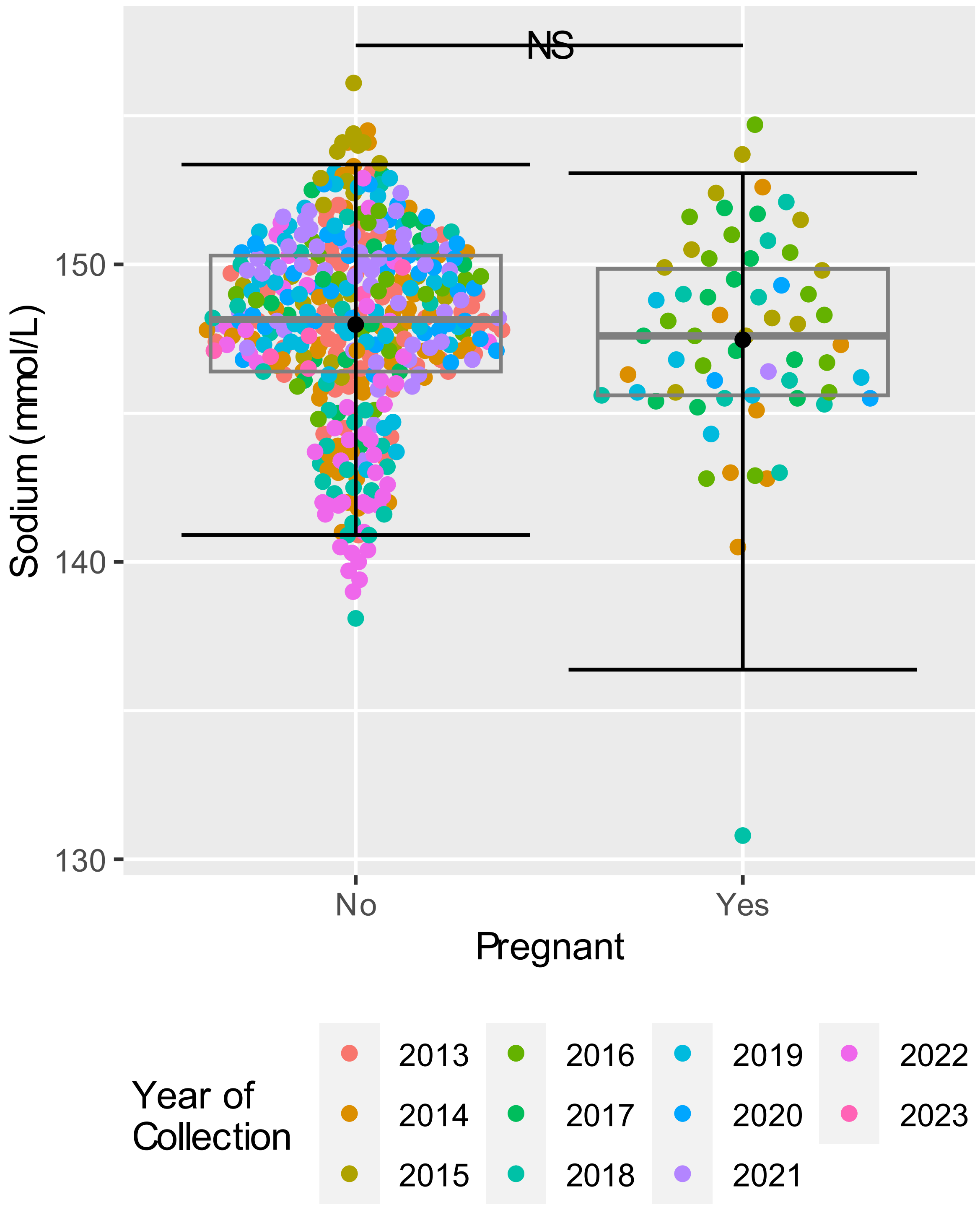

Figure S4 14. Sodium levels according to pregnancy status in adult female common marmosets. Boxes indicate median and quartile ranges; black point indicates arithmetic mean and error bars indicate the normal range. Symbol colours indicate year of collection; NS = not significant, asterisks indicate statistical significance levels * < 0.001, ** < 0.0001, *** < 0.00001, **** < 0.000001

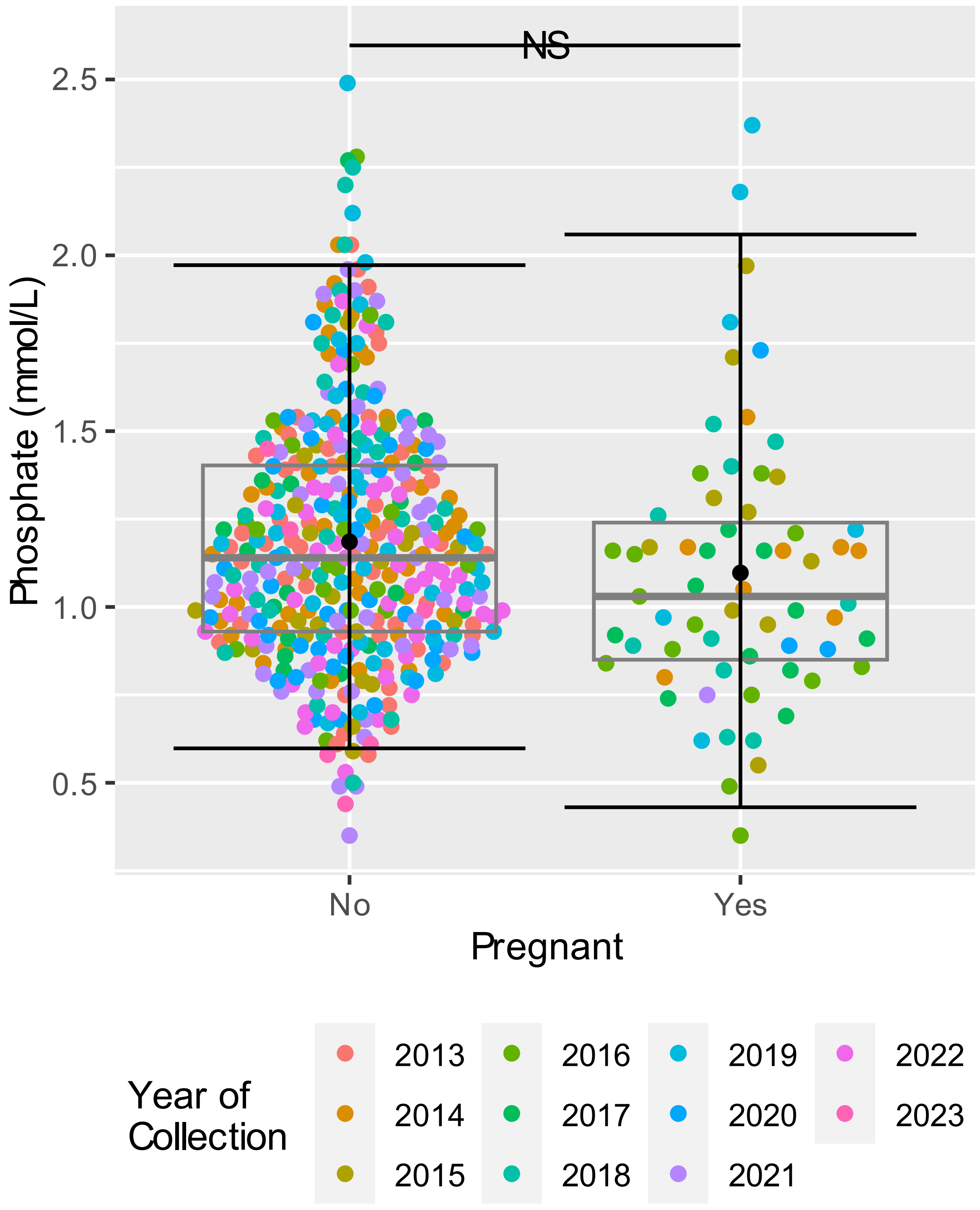

Figure S4 15. Phosphate levels according to pregnancy status in adult female common marmosets. Boxes indicate median and quartile ranges; black point indicates arithmetic mean and error bars indicate the normal range. Symbol colours indicate year of collection; NS = not significant, asterisks indicate statistical significance levels * < 0.001, ** < 0.0001, *** < 0.00001, **** < 0.000001

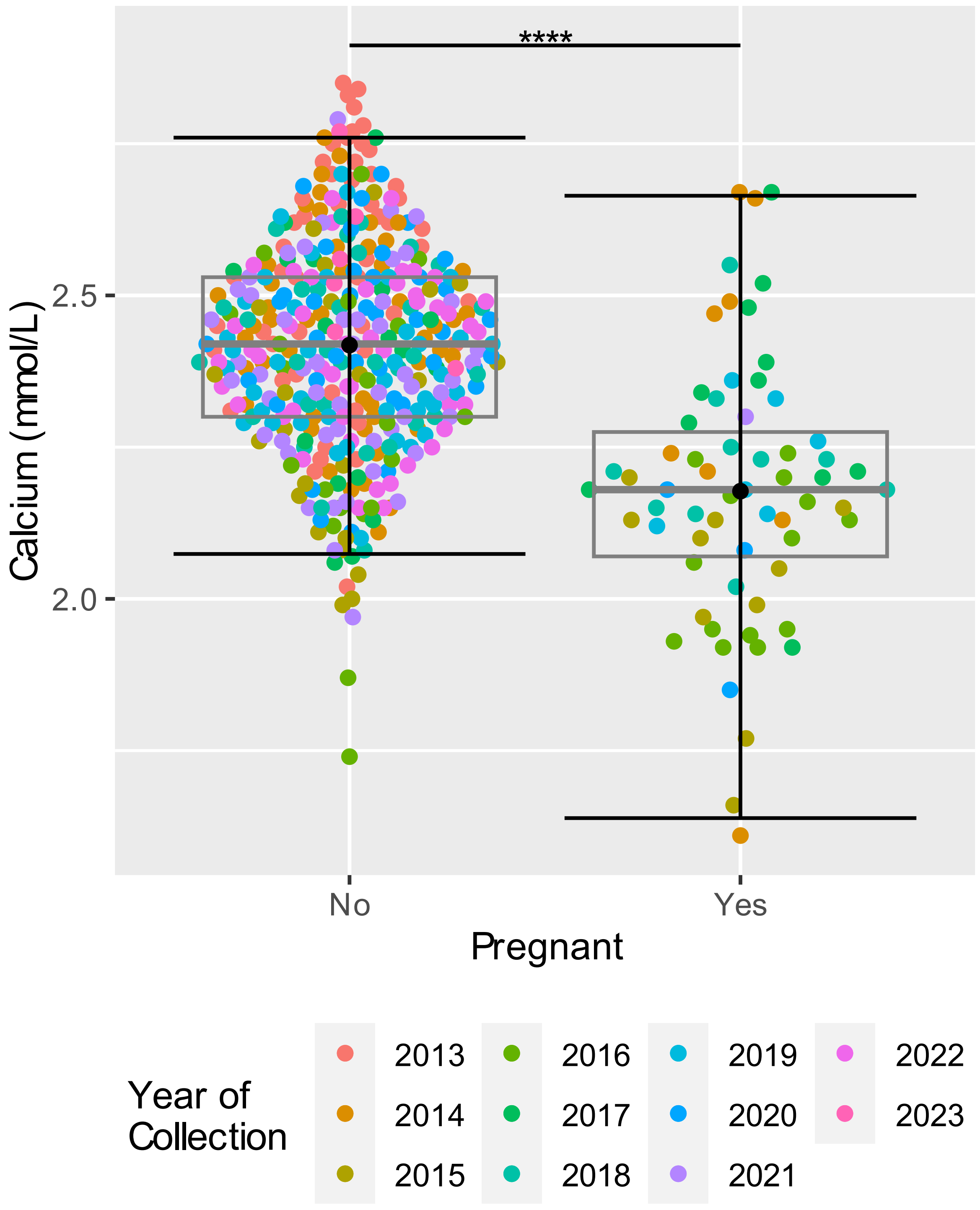

Figure S4 16. Calcium levels according to pregnancy status in adult female common marmosets. Boxes indicate median and quartile ranges; black point indicates arithmetic mean and error bars indicate the normal range. Symbol colours indicate year of collection; NS = not significant, asterisks indicate statistical significance levels * < 0.001, ** < 0.0001, *** < 0.00001, **** < 0.000001

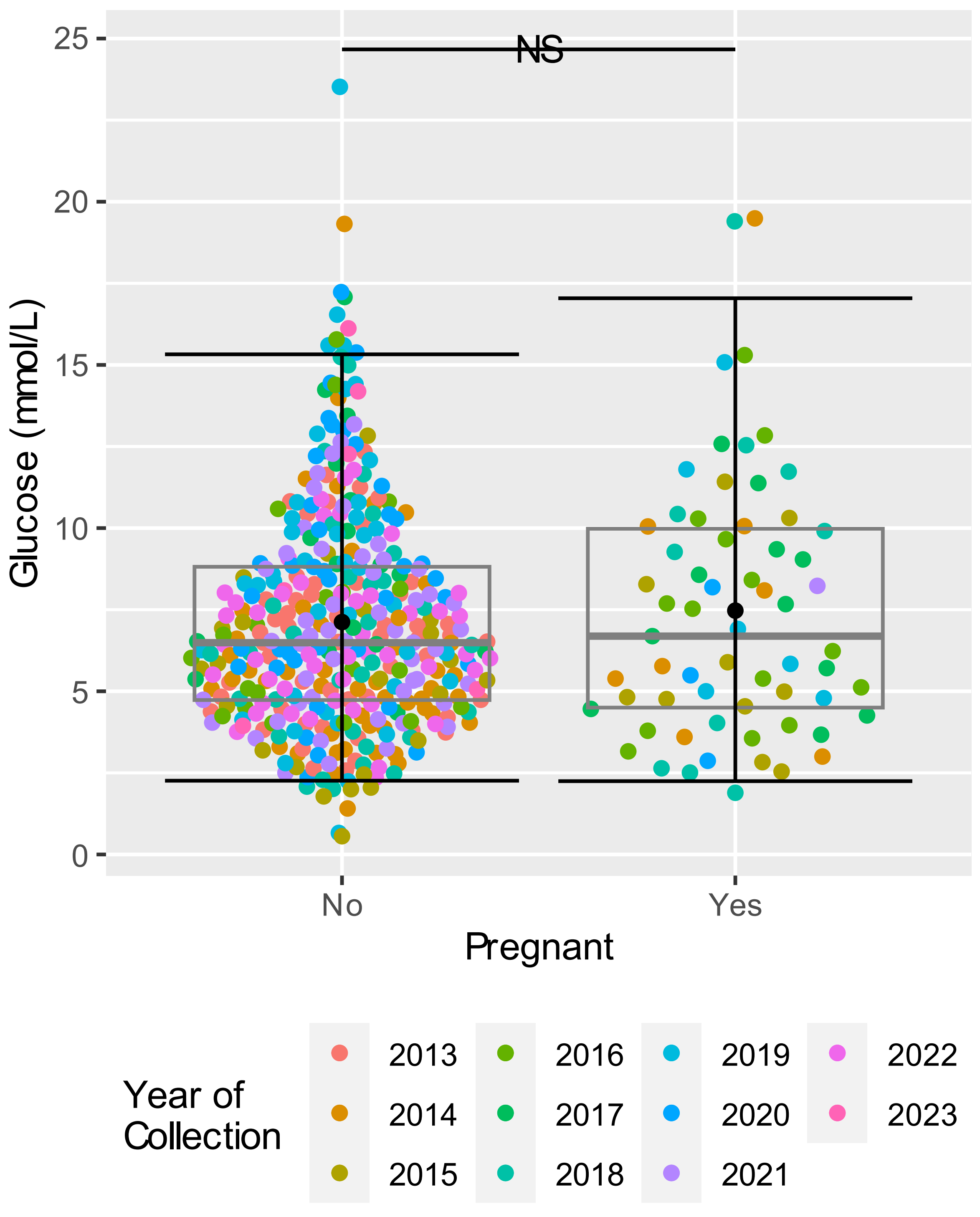

Figure S4 17. Glucose levels according to pregnancy status in adult female common marmosets. Boxes indicate median and quartile ranges; black point indicates arithmetic mean and error bars indicate the normal range. Symbol colours indicate year of collection; NS = not significant, asterisks indicate statistical significance levels * < 0.001, ** < 0.0001, *** < 0.00001, **** < 0.000001

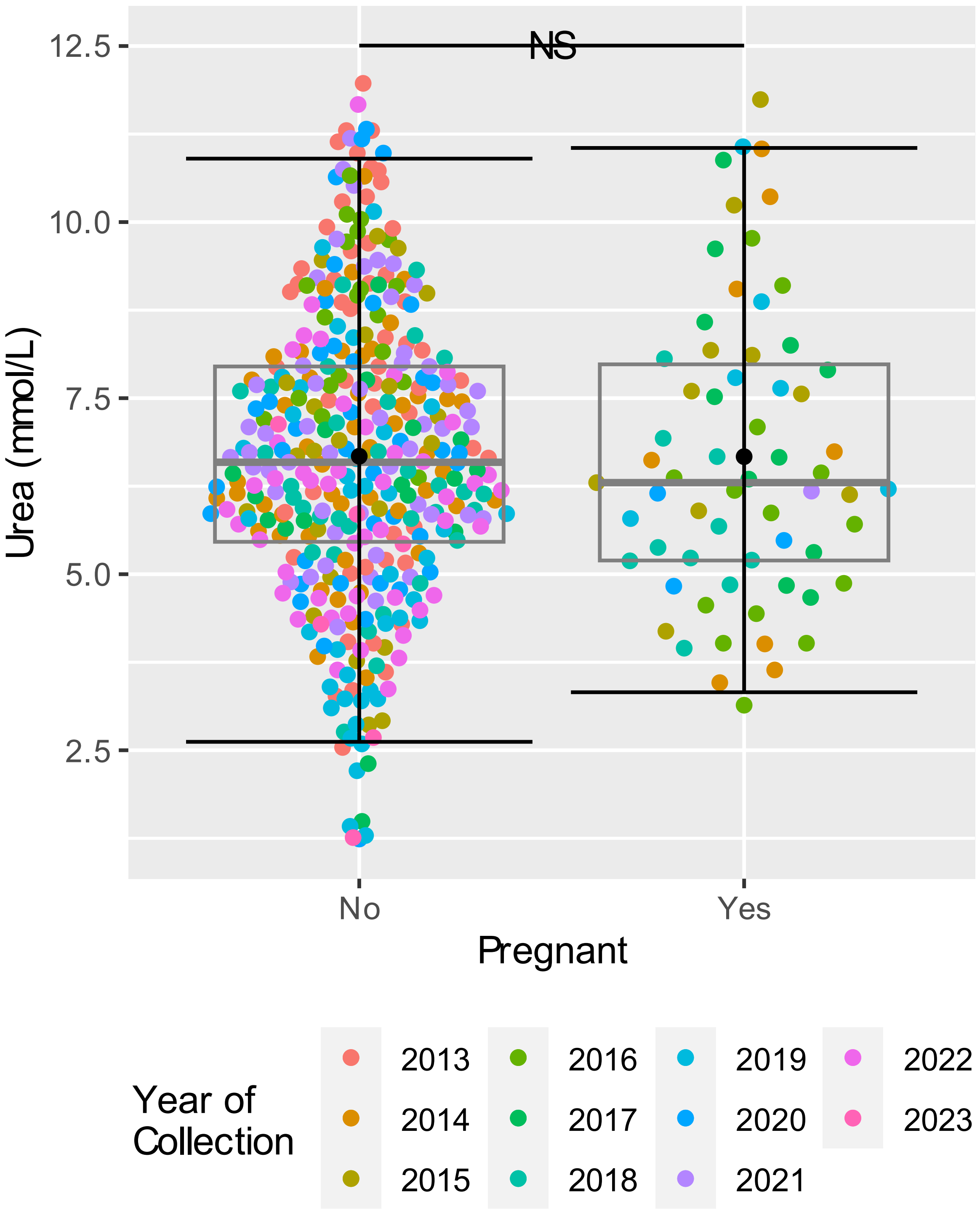

Figure S4 18. Urea levels according to pregnancy status in adult female common marmosets. Boxes indicate median and quartile ranges; black point indicates arithmetic mean and error bars indicate the normal range. Symbol colours indicate year of collection; NS = not significant, asterisks indicate statistical significance levels * < 0.001, ** < 0.0001, *** < 0.00001, **** < 0.000001

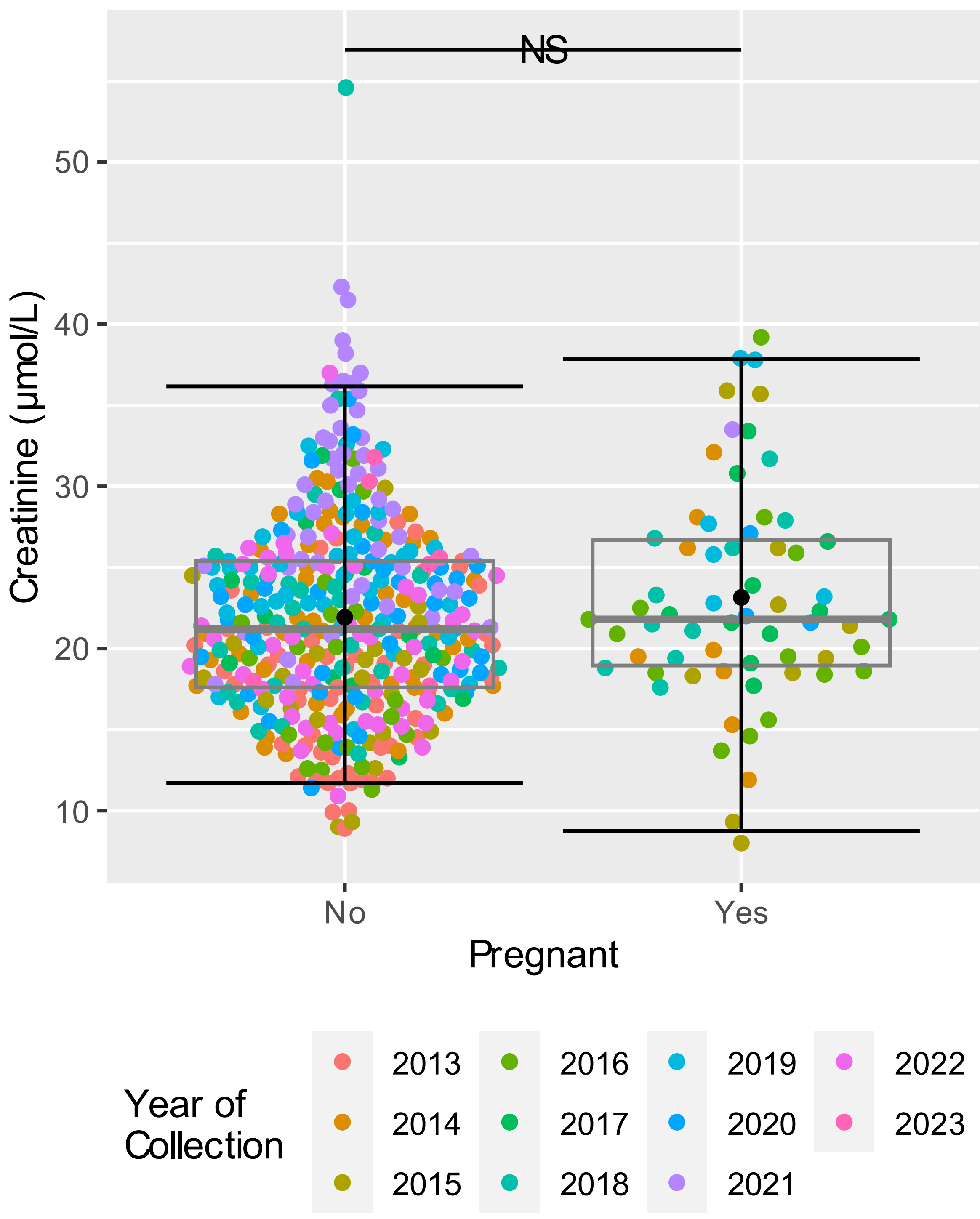

Figure S4 19. Creatinine levels according to pregnancy status in adult female common marmosets. Boxes indicate median and quartile ranges; black point indicates arithmetic mean and error bars indicate the normal range. Symbol colours indicate year of collection; NS = not significant, asterisks indicate statistical significance levels * < 0.001, ** < 0.0001, *** < 0.00001, **** < 0.000001

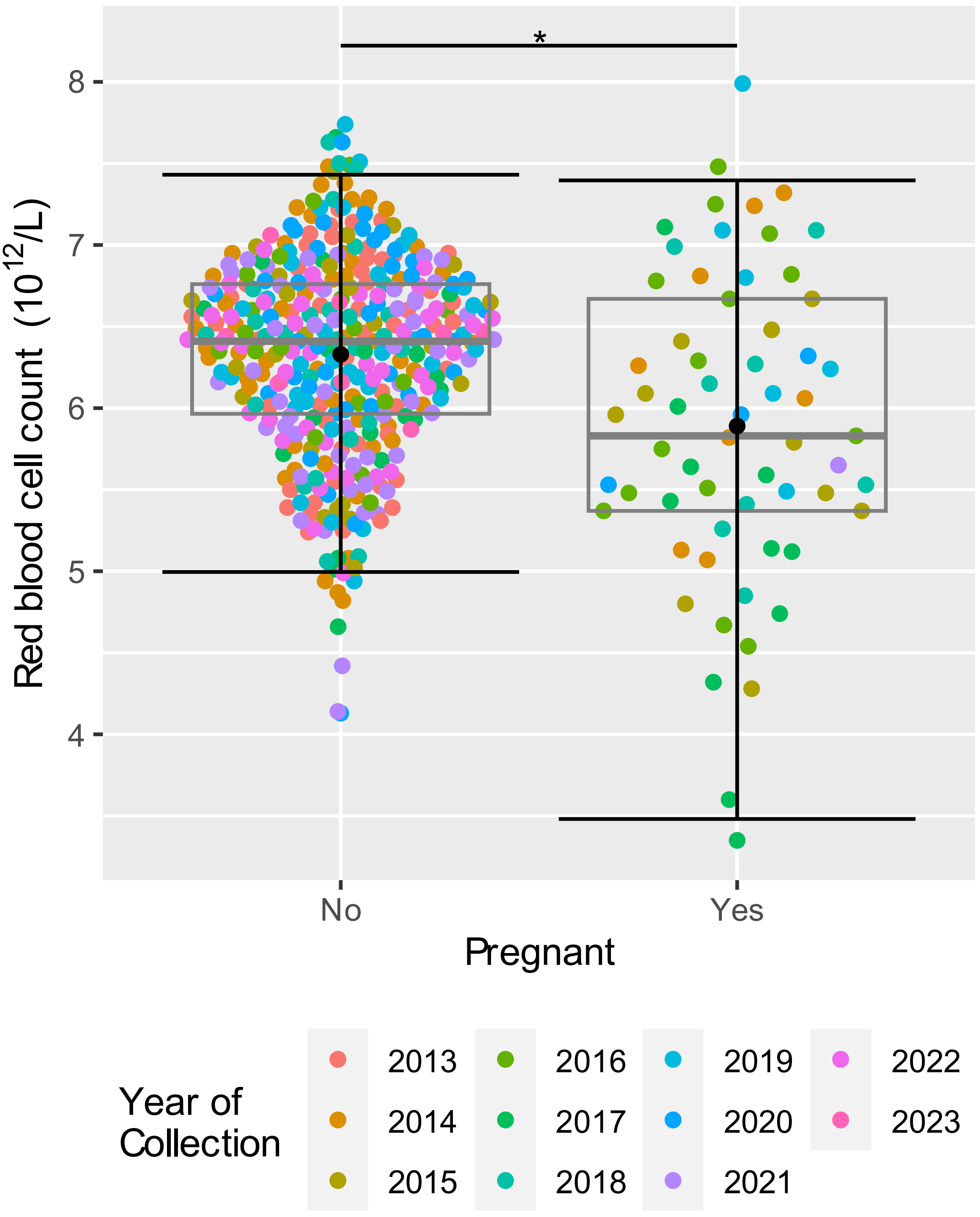

Figure S4 20. Red blood cell count according to pregnancy status in adult female common marmosets. Boxes indicate median and quartile ranges; black point indicates arithmetic mean and error bars indicate the normal range. Symbol colours indicate year of collection; NS = not significant, asterisks indicate statistical significance levels * < 0.001, ** < 0.0001, *** < 0.00001, **** < 0.000001

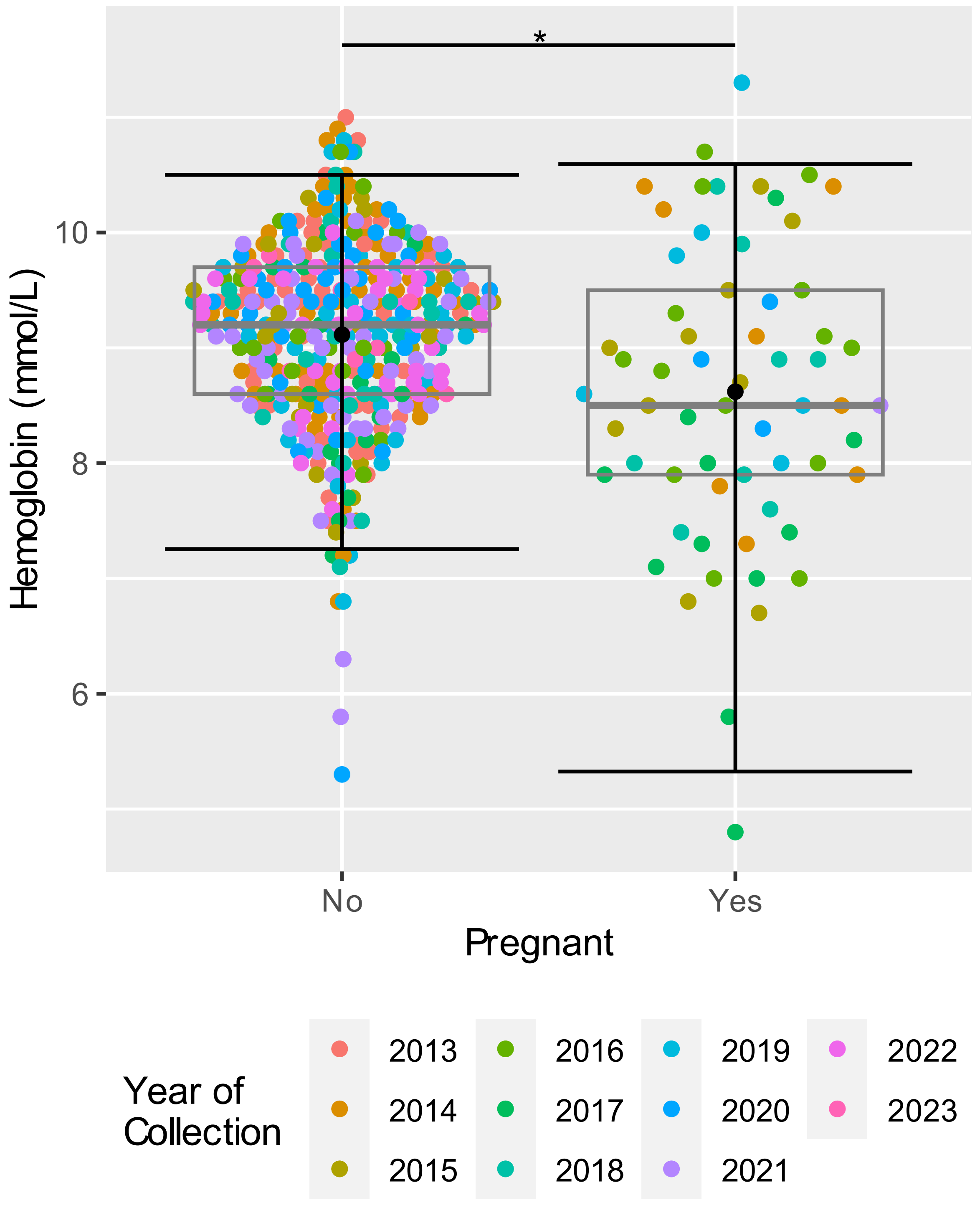

Figure S4 21. Haemoglobin levels according to pregnancy status in adult female common marmosets. Boxes indicate median and quartile ranges; black point indicates arithmetic mean and error bars indicate the normal range. Symbol colours indicate year of collection; NS = not significant, asterisks indicate statistical significance levels * < 0.001, ** < 0.0001, *** < 0.00001, **** < 0.000001

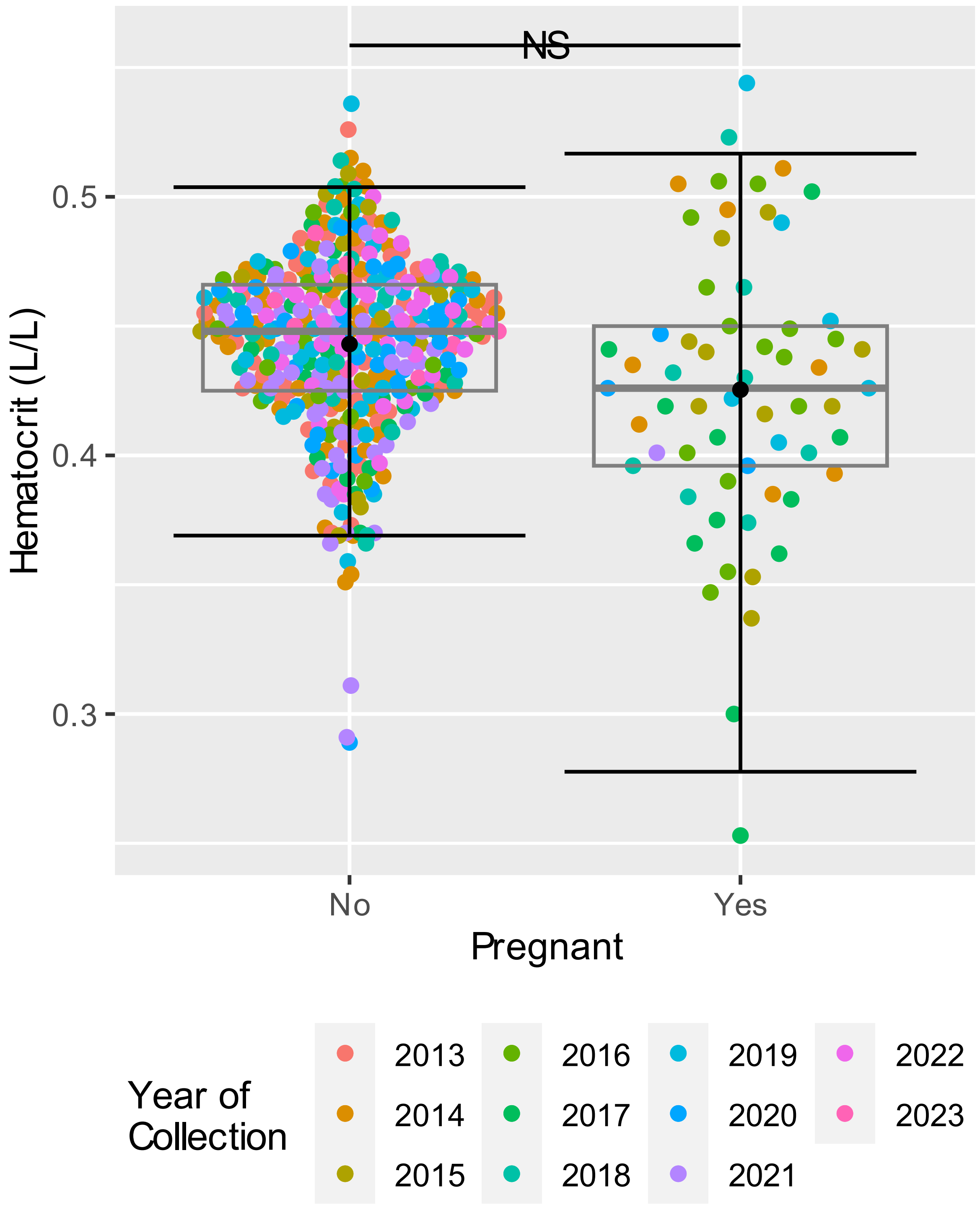

Figure S4 22. Haematocrit according to pregnancy status in adult female common marmosets. Boxes indicate median and quartile ranges; black point indicates arithmetic mean and error bars indicate the normal range. Symbol colours indicate year of collection; NS = not significant, asterisks indicate statistical significance levels * < 0.001, ** < 0.0001, *** < 0.00001, **** < 0.000001

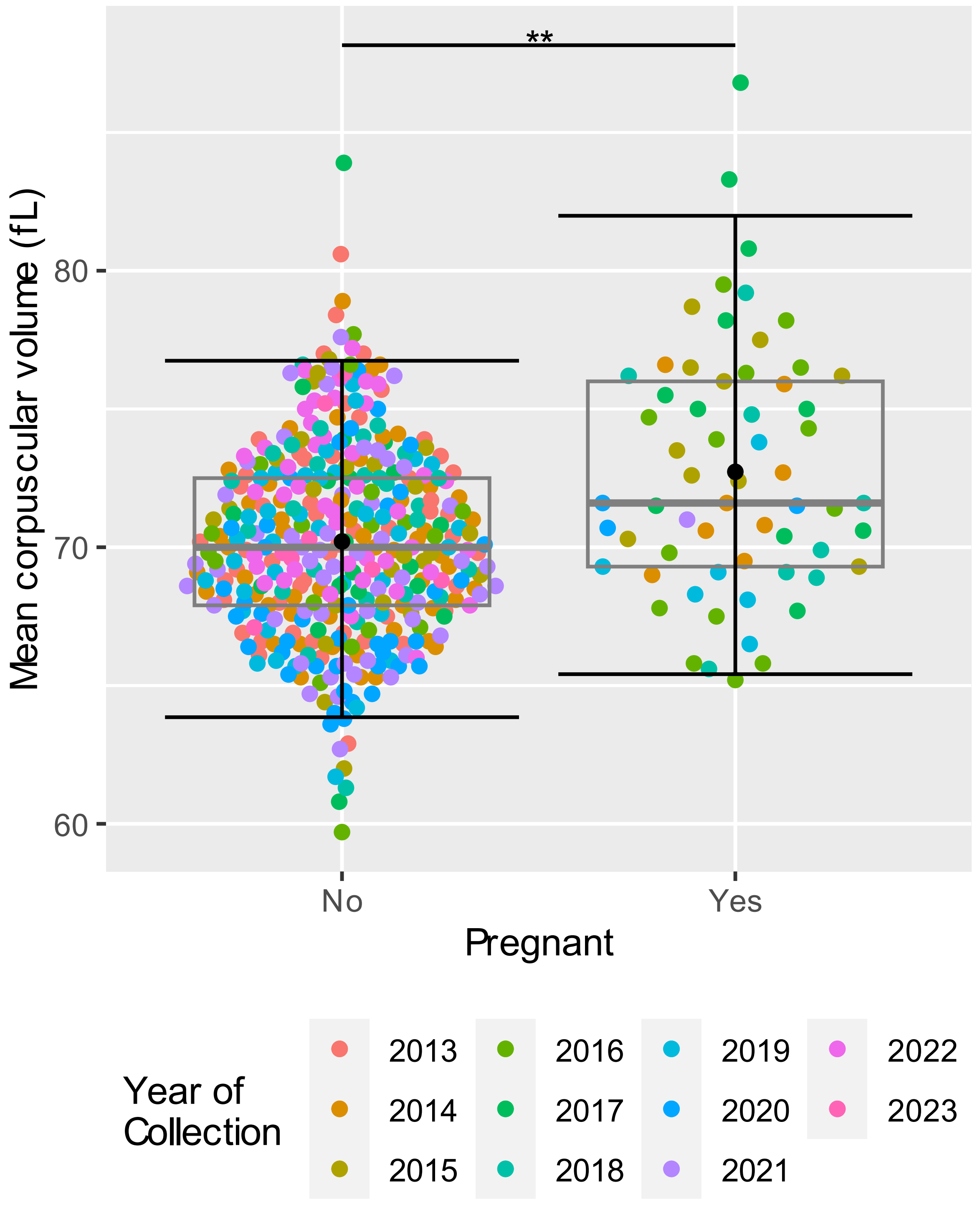

Figure S4 23. Mean Corpescular Volume according to pregnancy status in adult female common marmosets. Boxes indicate median and quartile ranges; black point indicates arithmetic mean and error bars indicate the normal range. Symbol colours indicate year of collection; NS = not significant, asterisks indicate statistical significance levels * < 0.001, ** < 0.0001, *** < 0.00001, **** < 0.000001

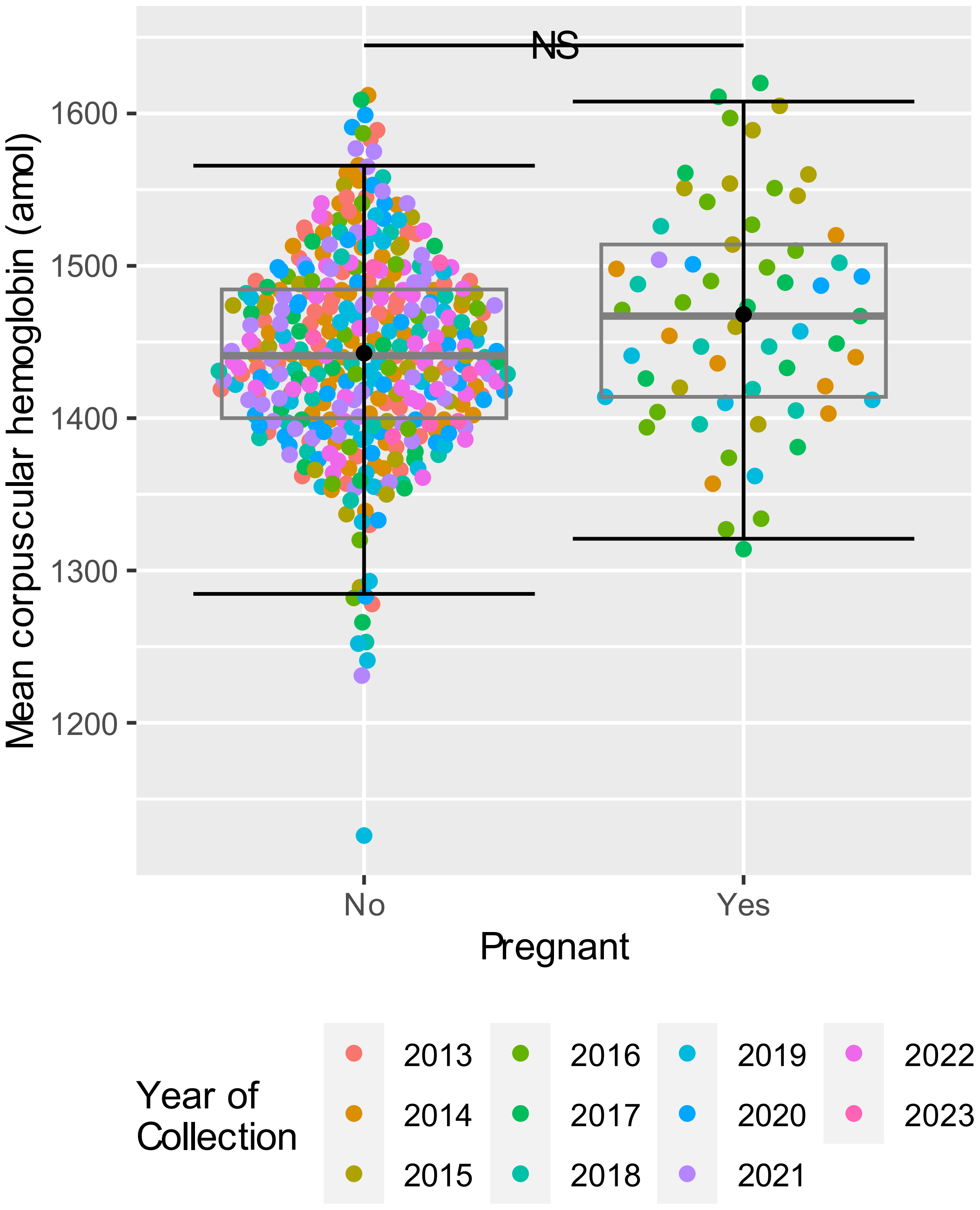

Figure S4 24. Mean Corpescular Haemoglobin according to pregnancy status in adult female common marmosets. Boxes indicate median and quartile ranges; black point indicates arithmetic mean and error bars indicate the normal range. Symbol colours indicate year of collection; NS = not significant, asterisks indicate statistical significance levels * < 0.001, ** < 0.0001, *** < 0.00001, **** < 0.000001

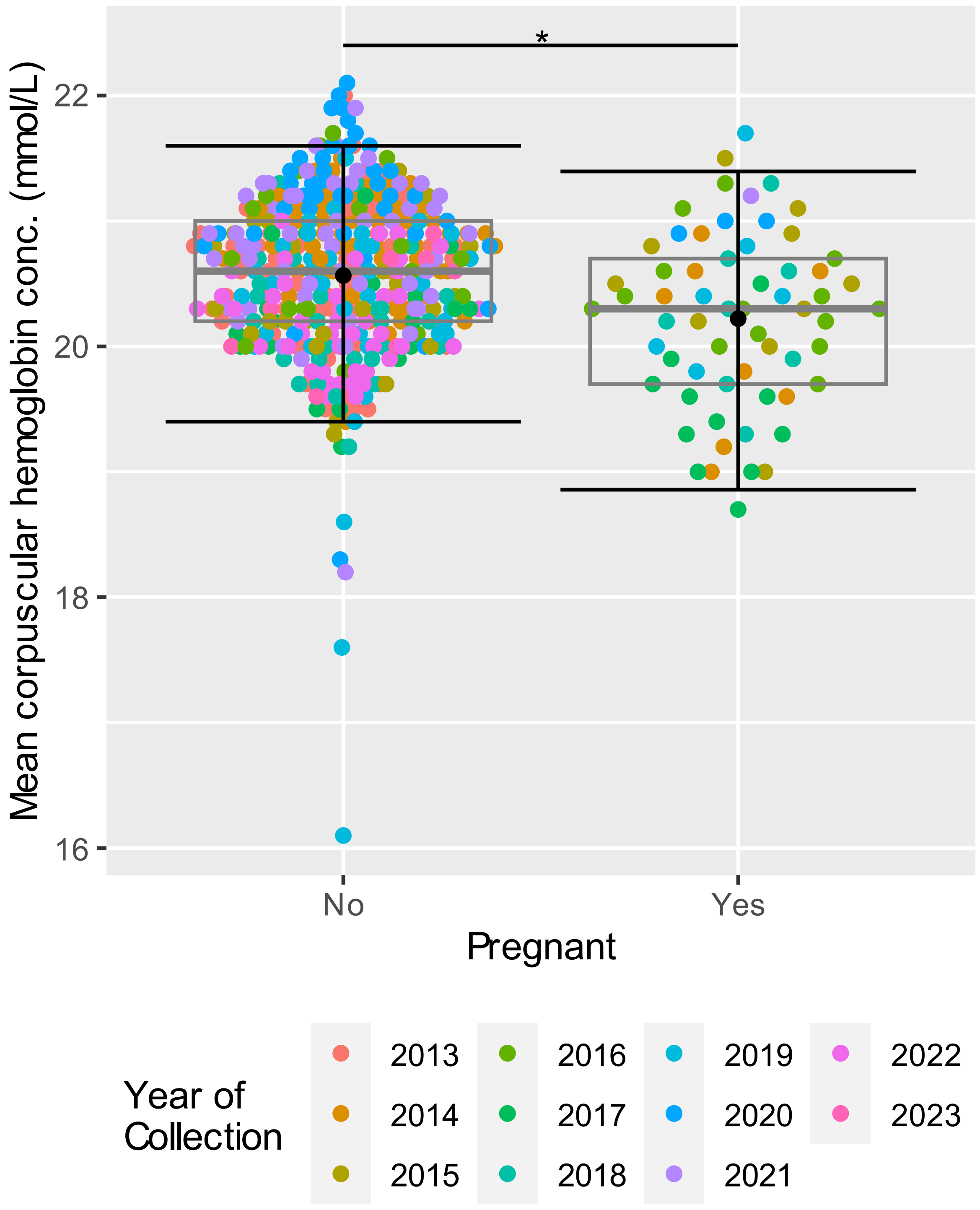

Figure S4 25. Mean Corpescular Haemoglobin Concentration according to pregnancy status in adult female common marmosets. Boxes indicate median and quartile ranges; black point indicates arithmetic mean and error bars indicate the normal range. Symbol colours indicate year of collection; NS = not significant, asterisks indicate statistical significance levels * < 0.001, ** < 0.0001, *** < 0.00001, **** < 0.000001

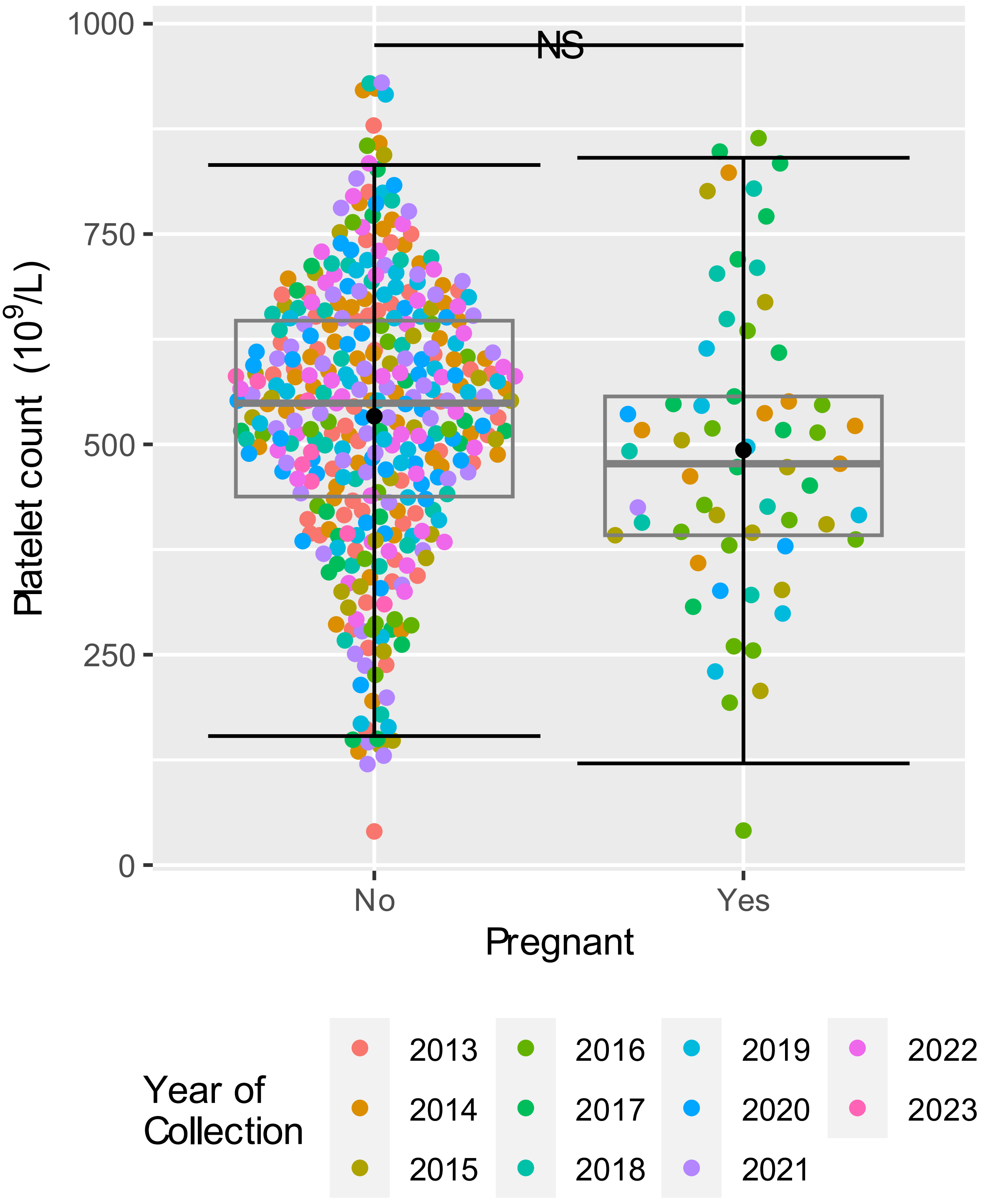

Figure S4 26. Platelet Count according to pregnancy status in adult female common marmosets. Boxes indicate median and quartile ranges; black point indicates arithmetic mean and error bars indicate the normal range. Symbol colours indicate year of collection; NS = not significant, asterisks indicate statistical significance levels * < 0.001, ** < 0.0001, *** < 0.00001, **** < 0.000001

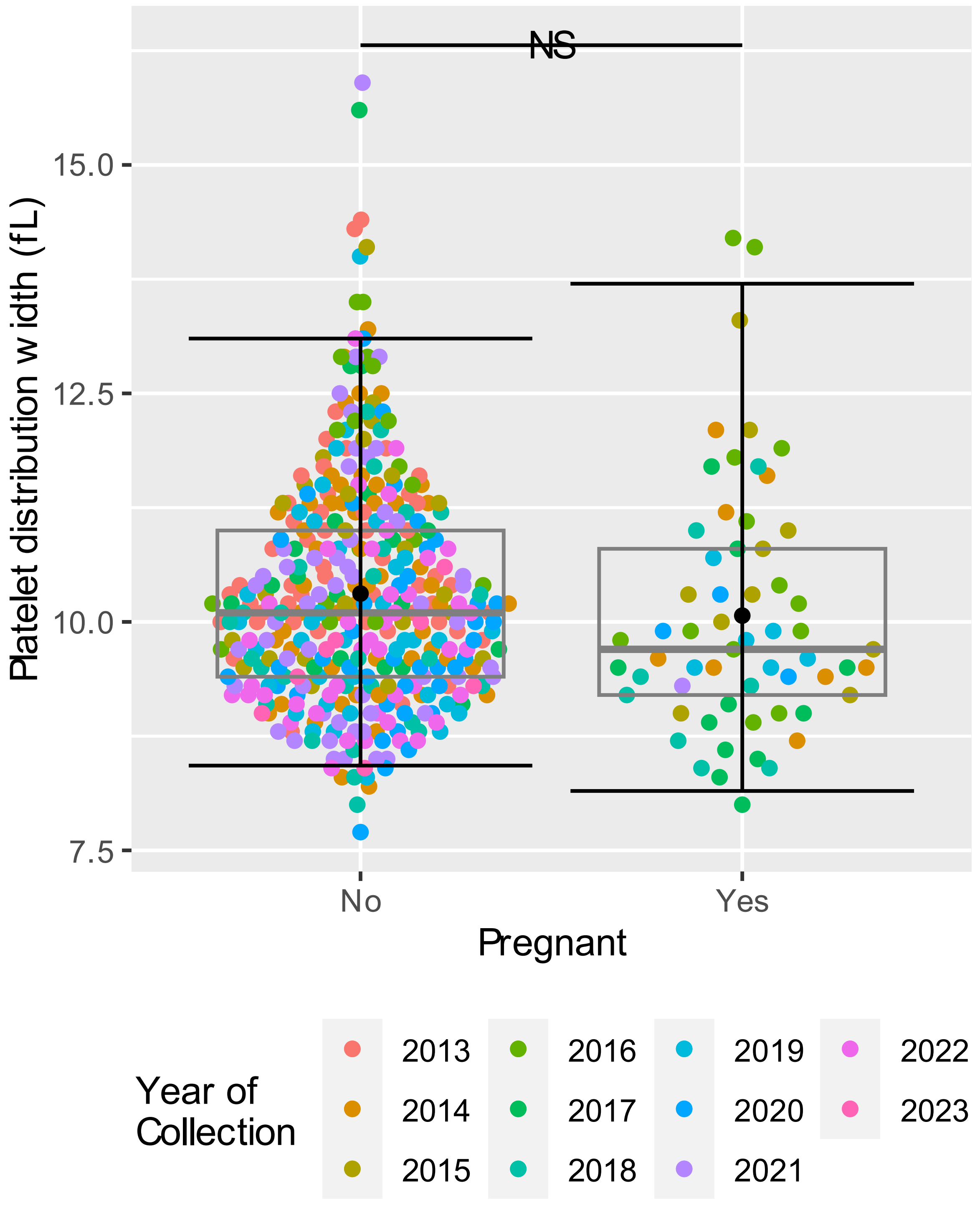

Figure S4 27. Platelet distribution width according to pregnancy status in adult female common marmosets. Boxes indicate median and quartile ranges; black point indicates arithmetic mean and error bars indicate the normal range. Symbol colours indicate year of collection; NS = not significant, asterisks indicate statistical significance levels * < 0.001, ** < 0.0001, *** < 0.00001, **** < 0.000001

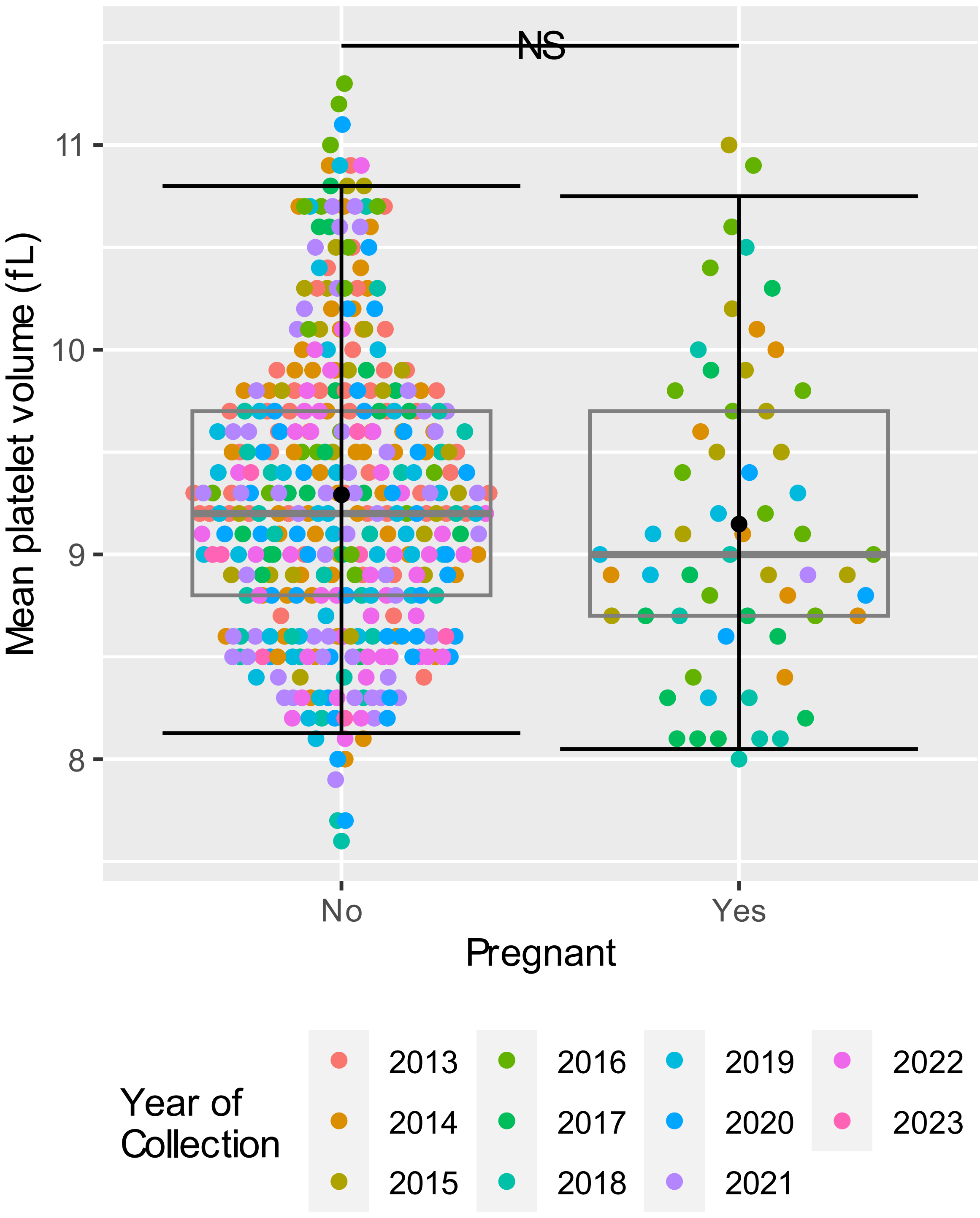

Figure S4 28. Mean Platelet Volume according to pregnancy status in adult female common marmosets. Boxes indicate median and quartile ranges; black point indicates arithmetic mean and error bars indicate the normal range. Symbol colours indicate year of collection; NS = not significant, asterisks indicate statistical significance levels * < 0.001, ** < 0.0001, *** < 0.00001, **** < 0.000001

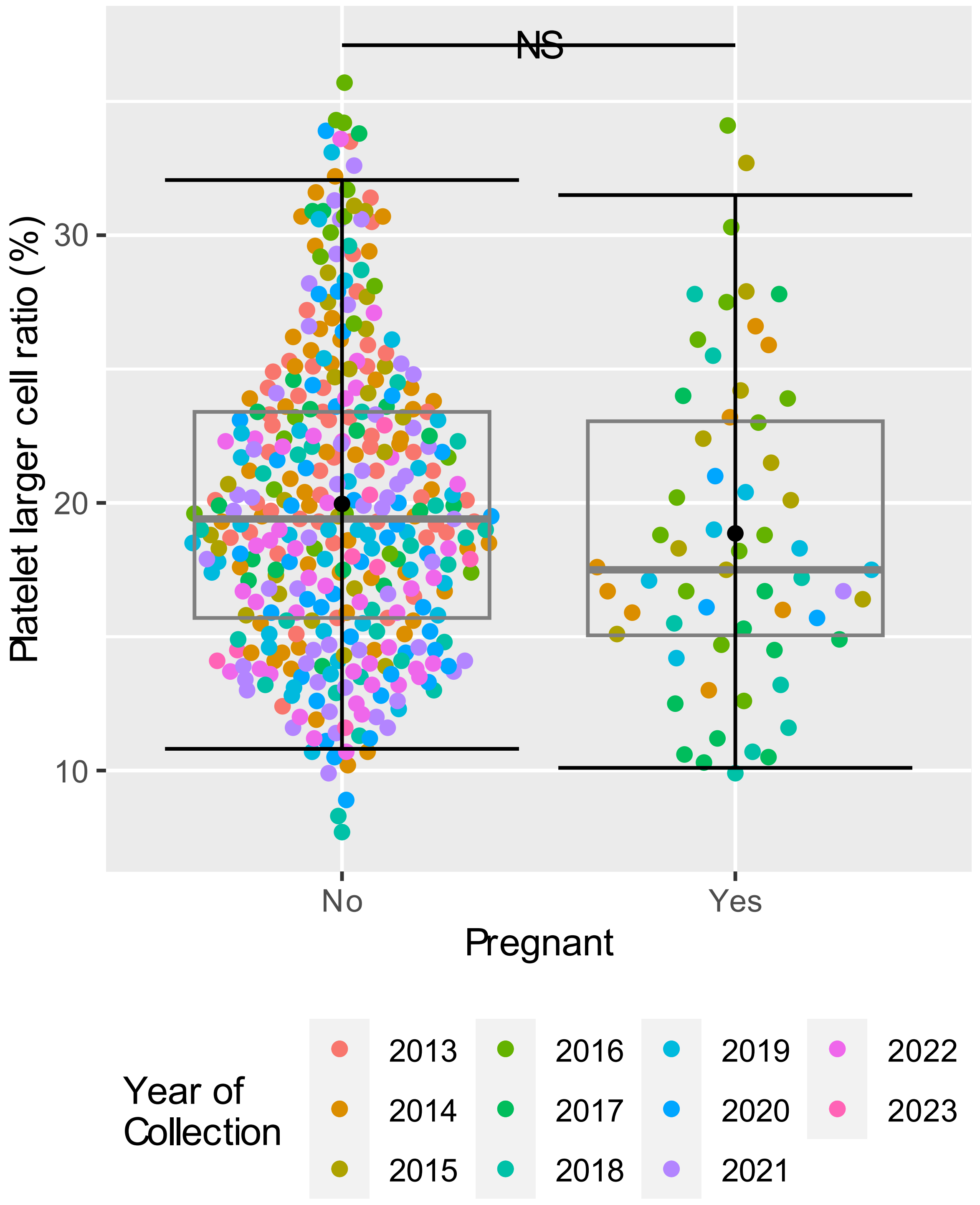

Figure S4 29. Platelet Larger Cell Ratio according to pregnancy status in adult female common marmosets. Boxes indicate median and quartile ranges; black point indicates arithmetic mean and error bars indicate the normal range. Symbol colours indicate year of collection; NS = not significant, asterisks indicate statistical significance levels * < 0.001, ** < 0.0001, *** < 0.00001, **** < 0.000001

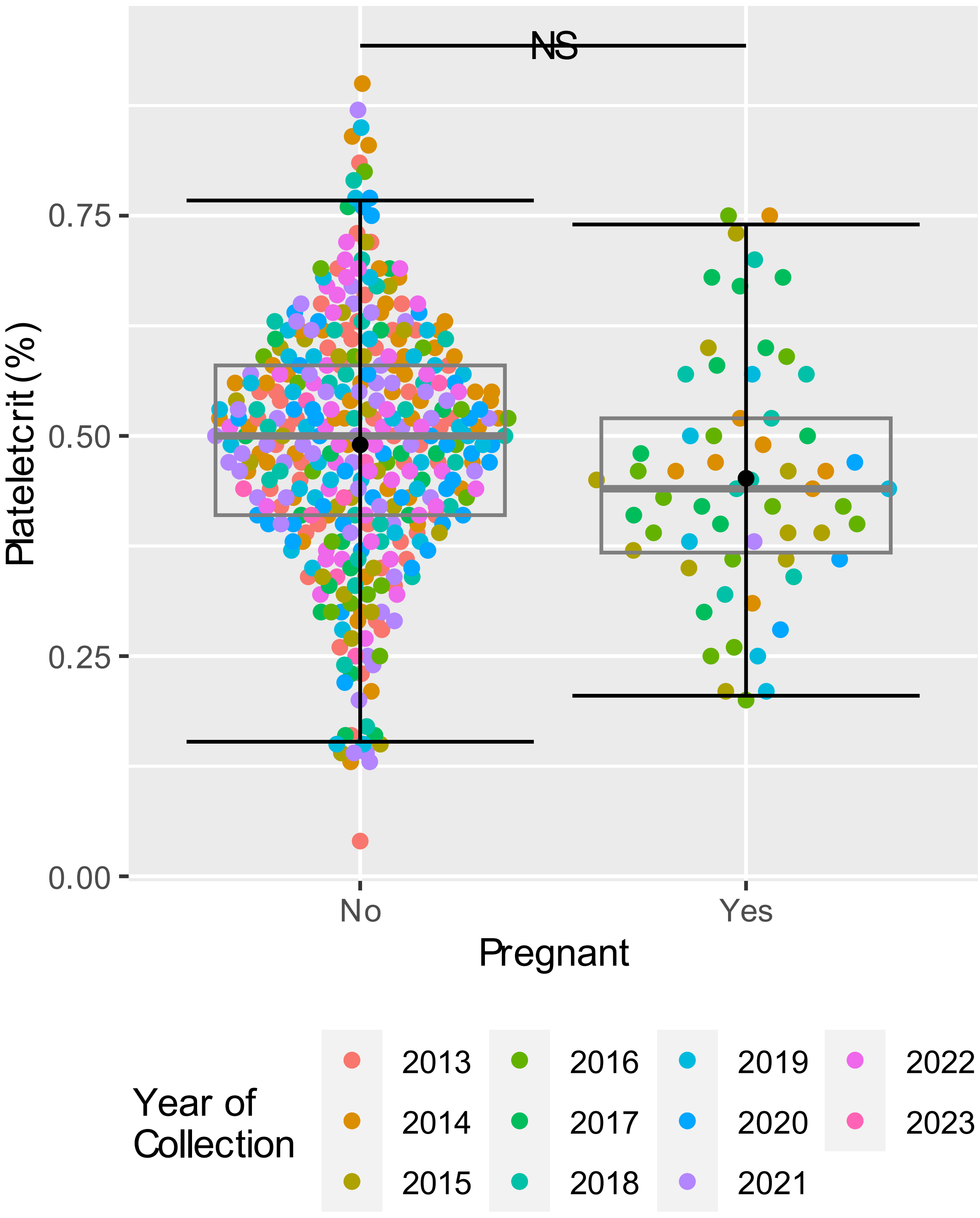

Figure S4 30. Plateletcrit according to pregnancy status in adult female common marmosets. Boxes indicate median and quartile ranges; black point indicates arithmetic mean and error bars indicate the normal range. Symbol colours indicate year of collection; NS = not significant, asterisks indicate statistical significance levels * < 0.001, ** < 0.0001, *** < 0.00001, **** < 0.000001

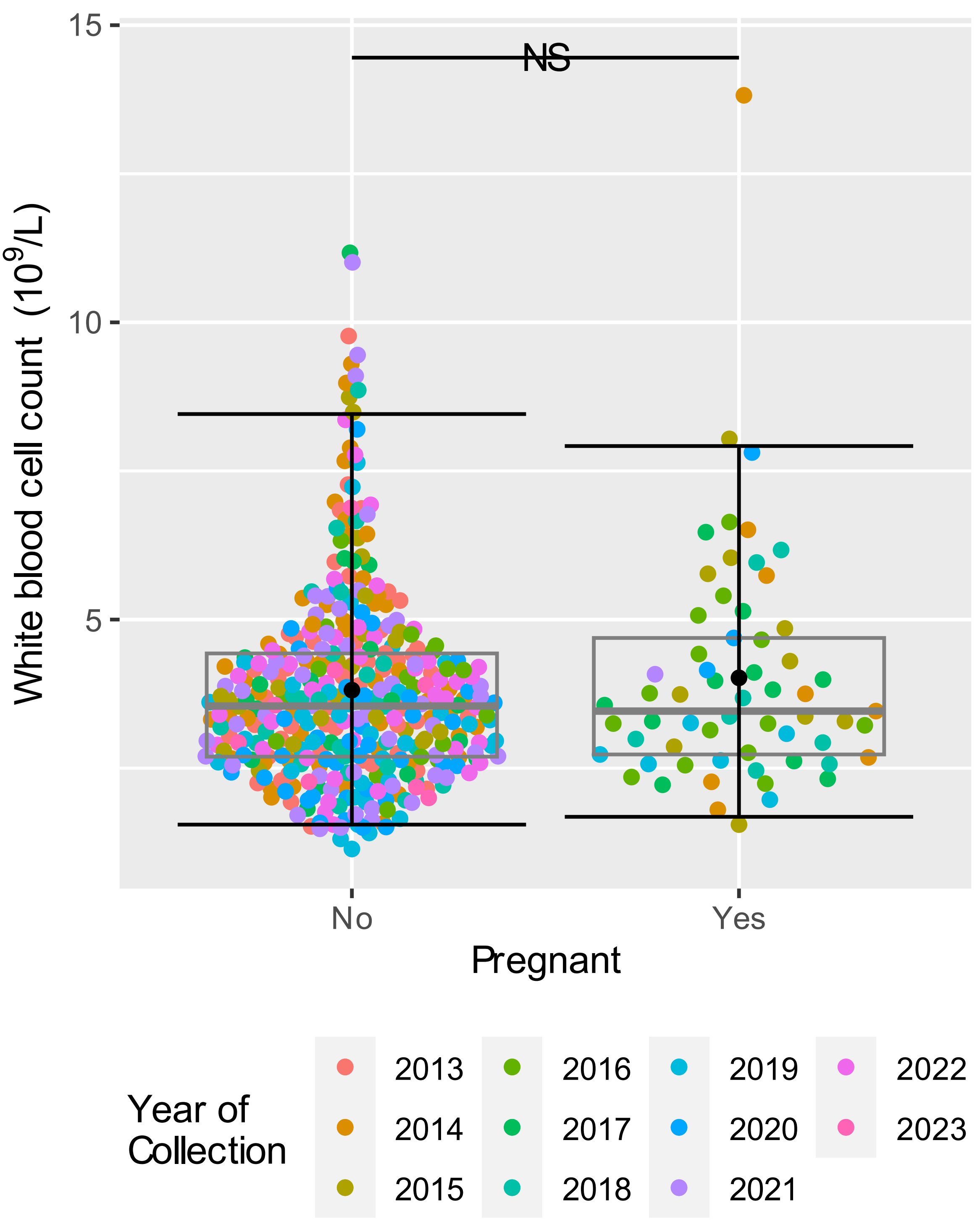

Figure S4 31. White Blood Cell count according to pregnancy status in adult female common marmosets. Boxes indicate median and quartile ranges; black point indicates arithmetic mean and error bars indicate the normal range. Symbol colours indicate year of collection; NS = not significant, asterisks indicate statistical significance levels * < 0.001, ** < 0.0001, *** < 0.00001, **** < 0.000001

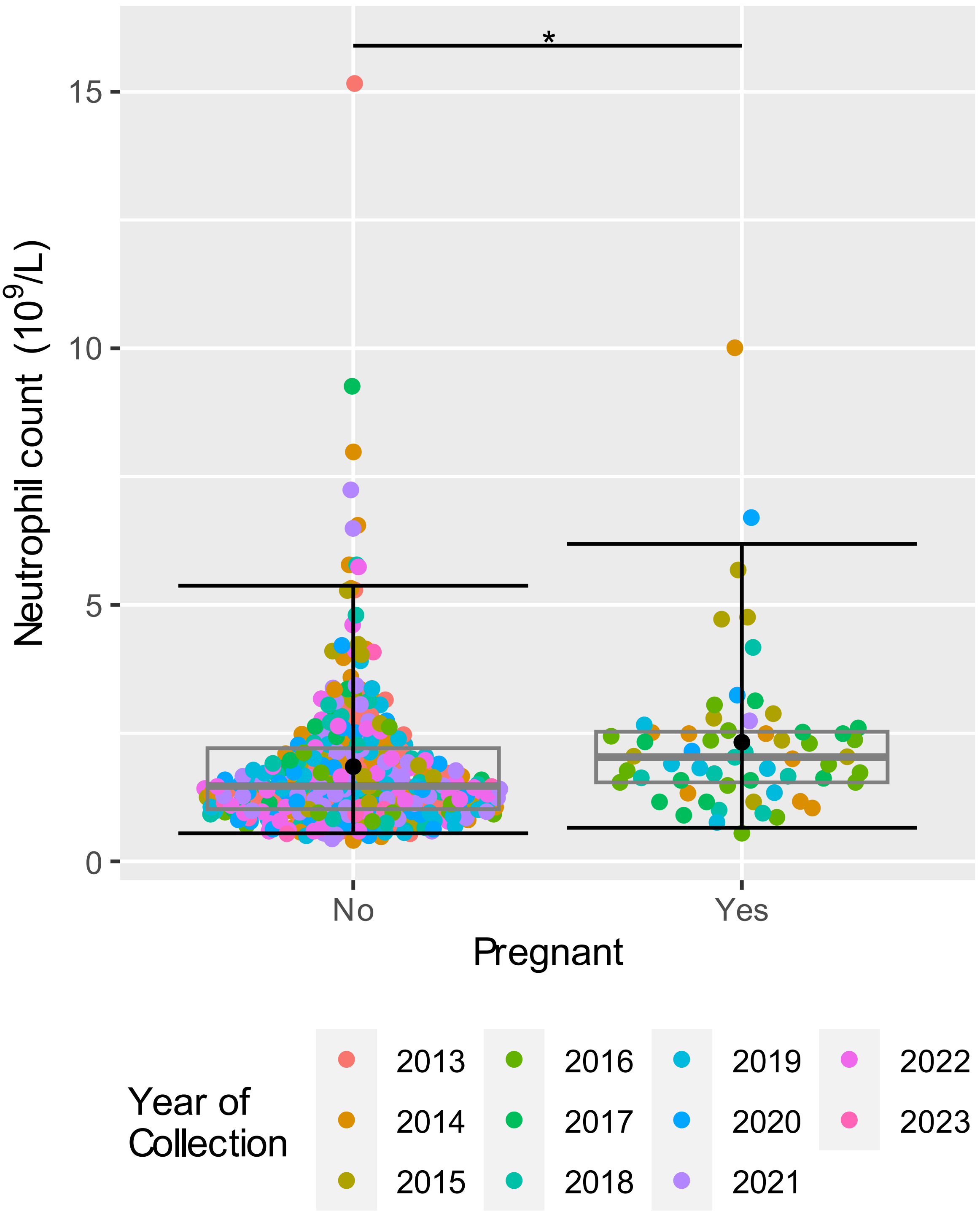

Figure S4 32. Neutrophil count according to pregnancy status in adult female common marmosets. Boxes indicate median and quartile ranges; black point indicates arithmetic mean and error bars indicate the normal range. Symbol colours indicate year of collection; NS = not significant, asterisks indicate statistical significance levels * < 0.001, ** < 0.0001, *** < 0.00001, **** < 0.000001

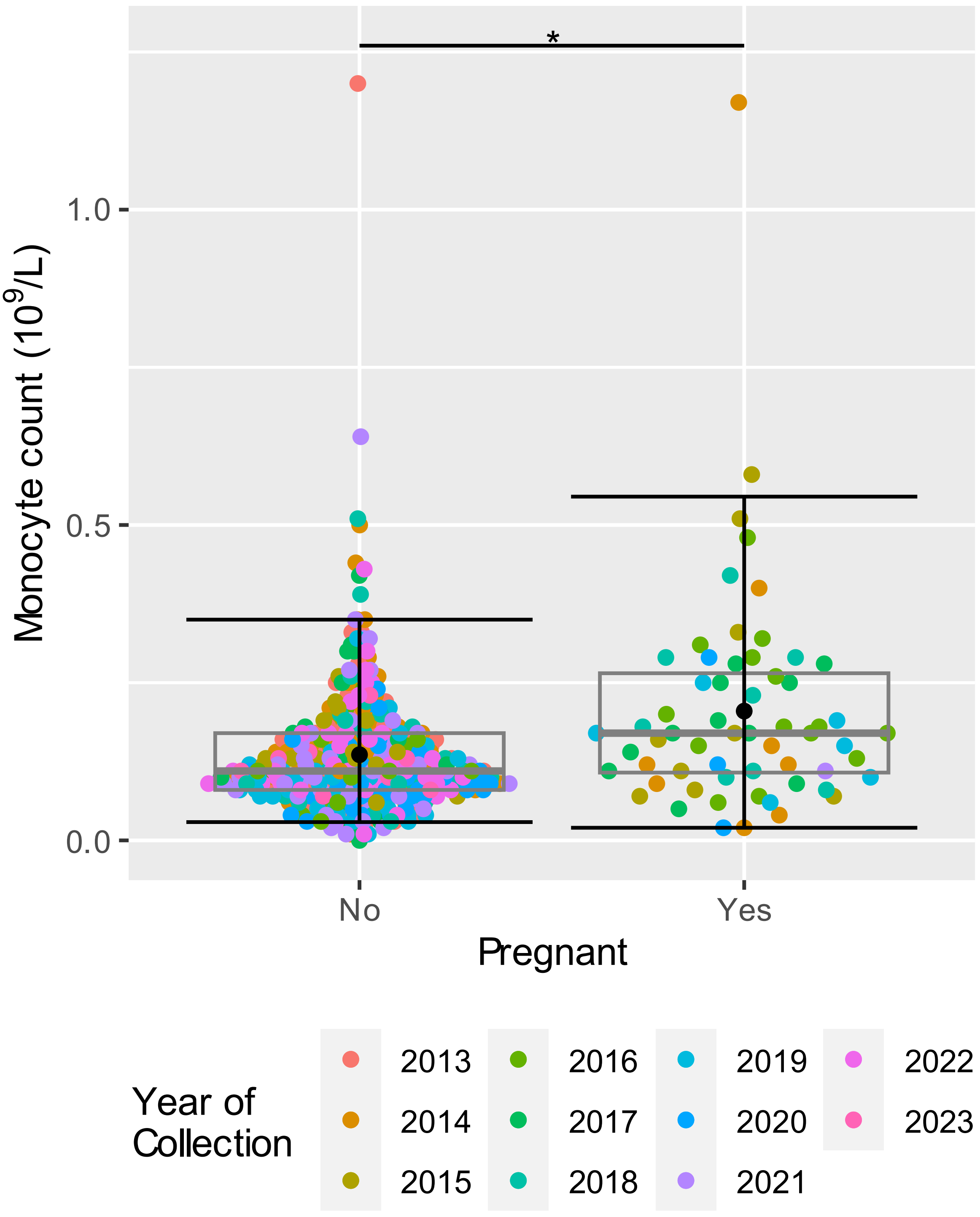

Figure S4 33. Monocyte count according to pregnancy status in adult female common marmosets. Boxes indicate median and quartile ranges; black point indicates arithmetic mean and error bars indicate the normal range. Symbol colours indicate year of collection; NS = not significant, asterisks indicate statistical significance levels * < 0.001, ** < 0.0001, *** < 0.00001, **** < 0.000001

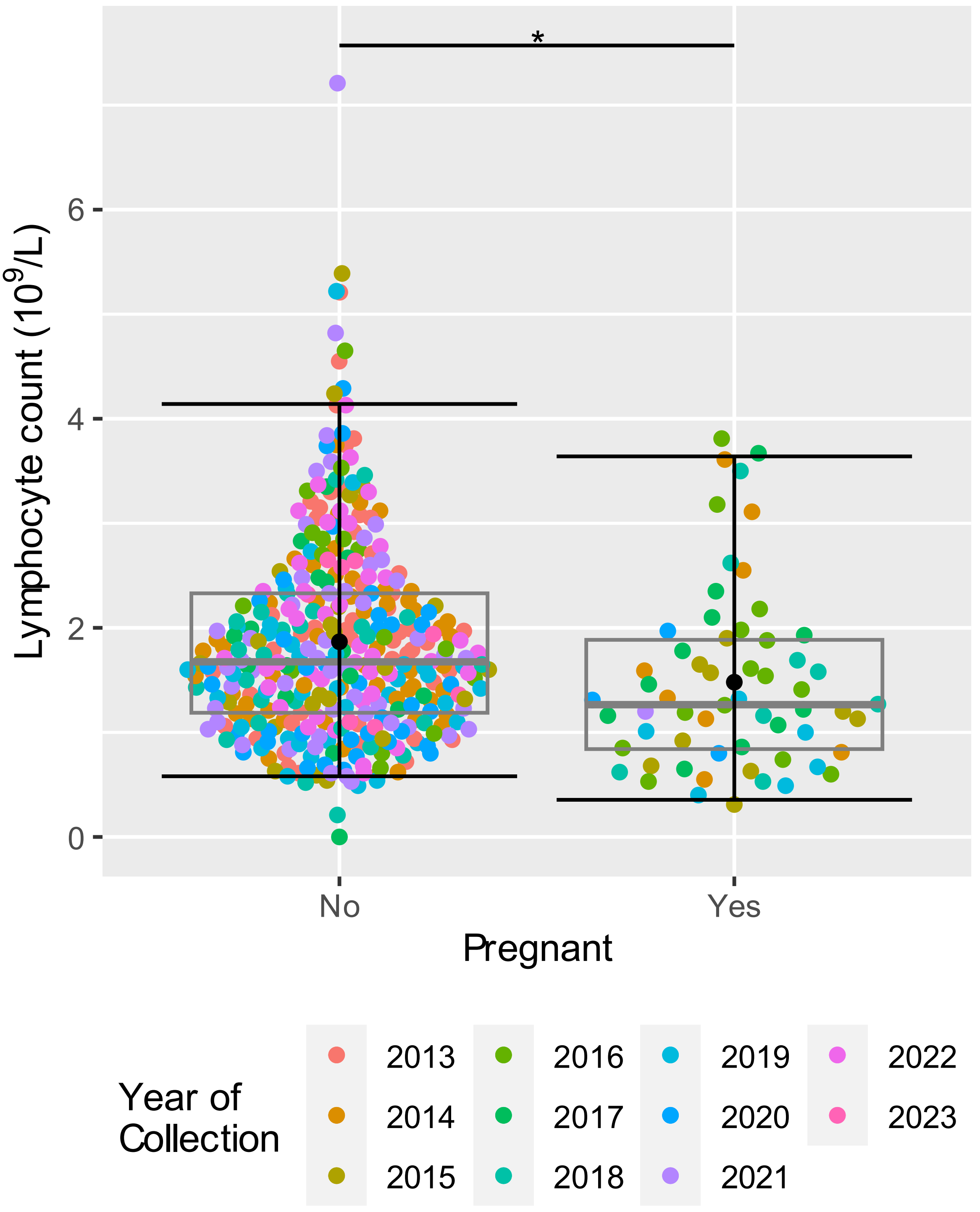

Figure S4 34. Lymphocyte count according to pregnancy status in adult female common marmosets. Boxes indicate median and quartile ranges; black point indicates arithmetic mean and error bars indicate the normal range. Symbol colours indicate year of collection; NS = not significant, asterisks indicate statistical significance levels * < 0.001, ** < 0.0001, *** < 0.00001, **** < 0.000001

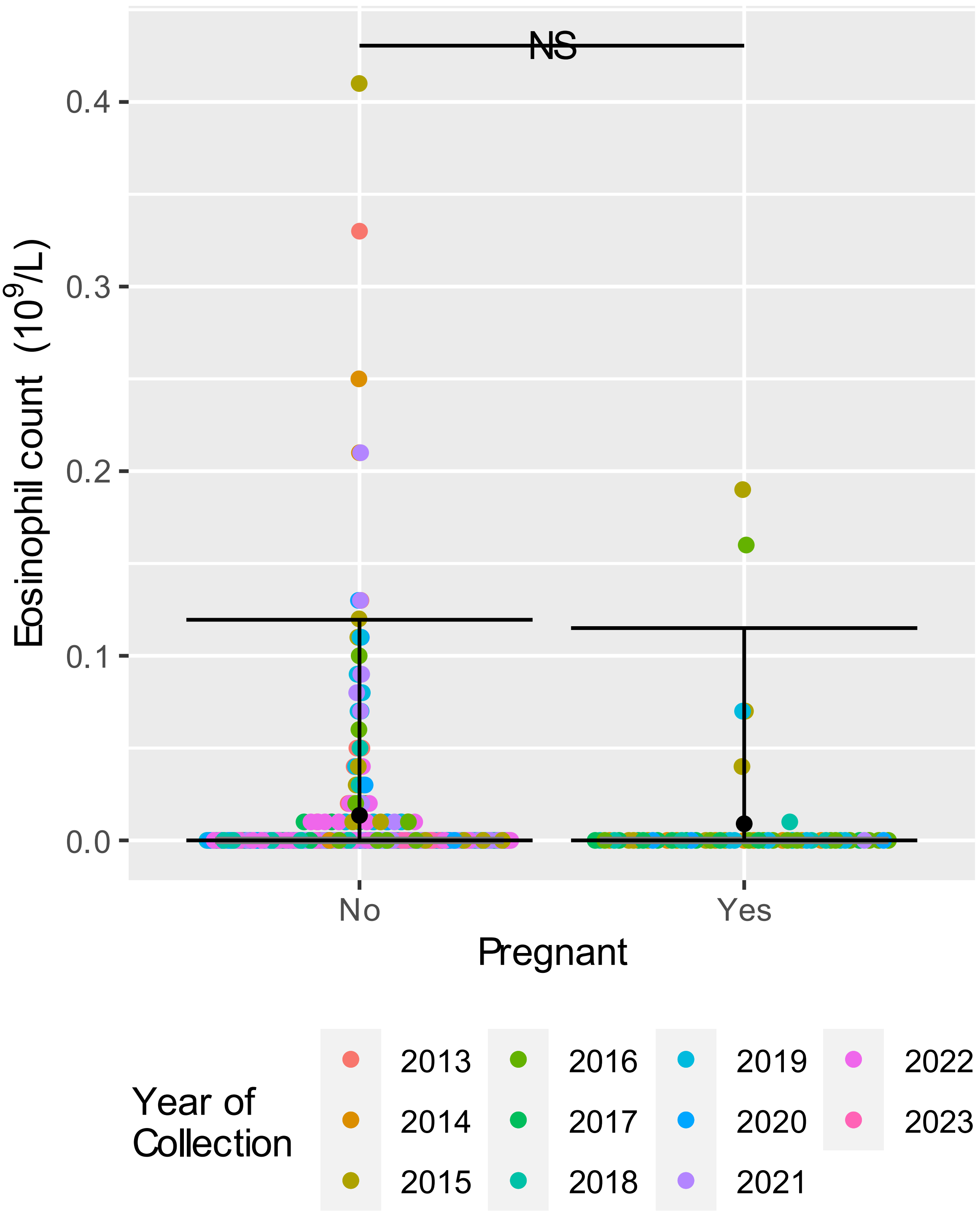

Figure S4 35. Eosinophil count according to pregnancy status in adult female common marmosets. Boxes indicate median and quartile ranges; black point indicates arithmetic mean and error bars indicate the normal range. Symbol colours indicate year of collection; NS = not significant, asterisks indicate statistical significance levels * < 0.001, ** < 0.0001, *** < 0.00001, **** < 0.000001

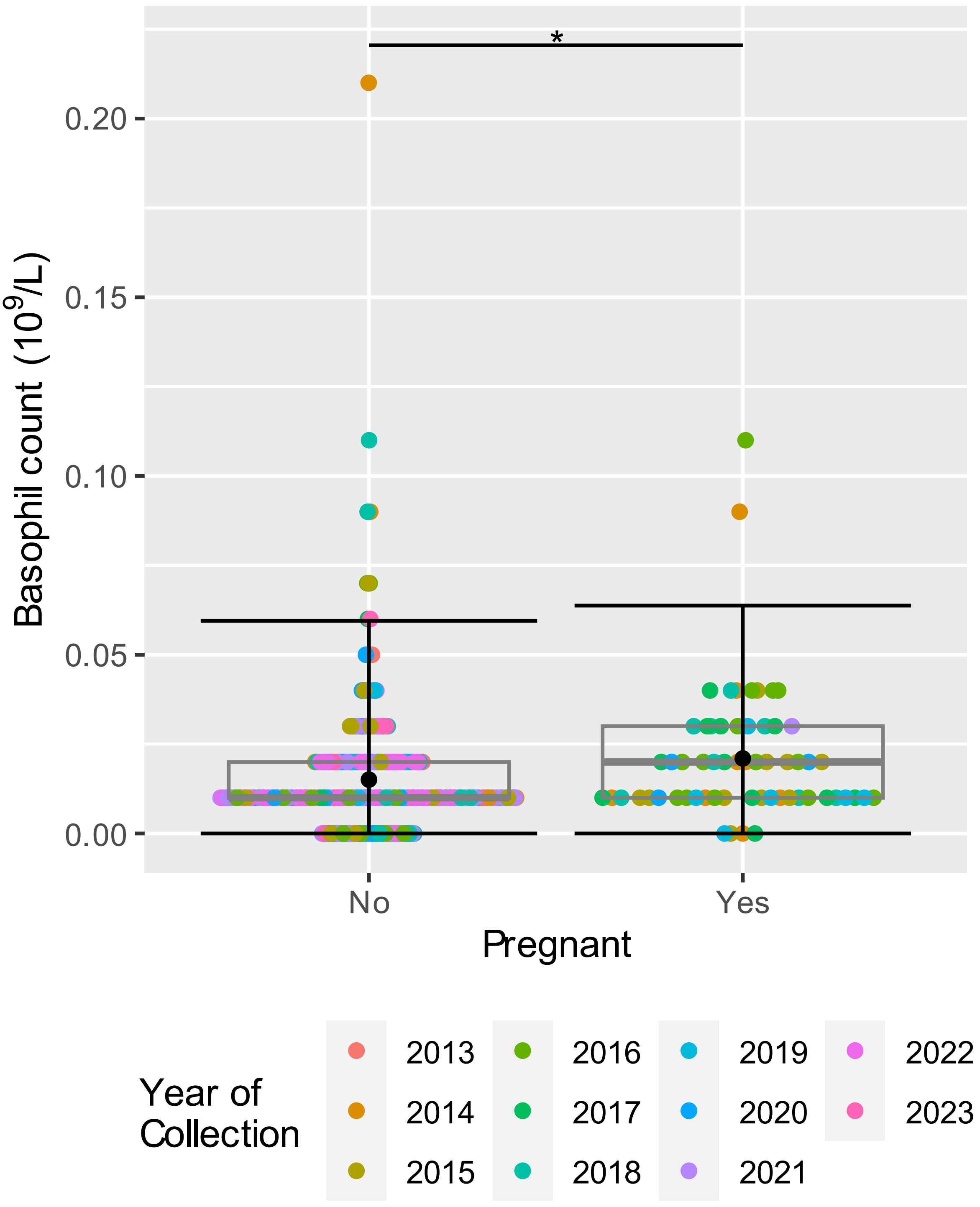

Figure S4 36. Basophil count according to pregnancy status in adult female common marmosets. Boxes indicate median and quartile ranges; black point indicates arithmetic mean and error bars indicate the normal range. Symbol colours indicate year of collection; NS = not significant, asterisks indicate statistical significance levels * < 0.001, ** < 0.0001, *** < 0.00001, **** < 0.000001
