# Supplementary material for: Hematological and Serum Biochemical Reference Intervals for Alphaxalone Sedated Common Marmosets (Callithrix jacchus)
Source: Animals (Basel). 2024 Mar 3;14(5):790. doi: 10.3390/ani14050790 (PMC10930924; doi:10.3390/ani14050790)
Supplement: Supplementary file 1 [file animals-14-00790-s001.zip › Figure S5.pdf]

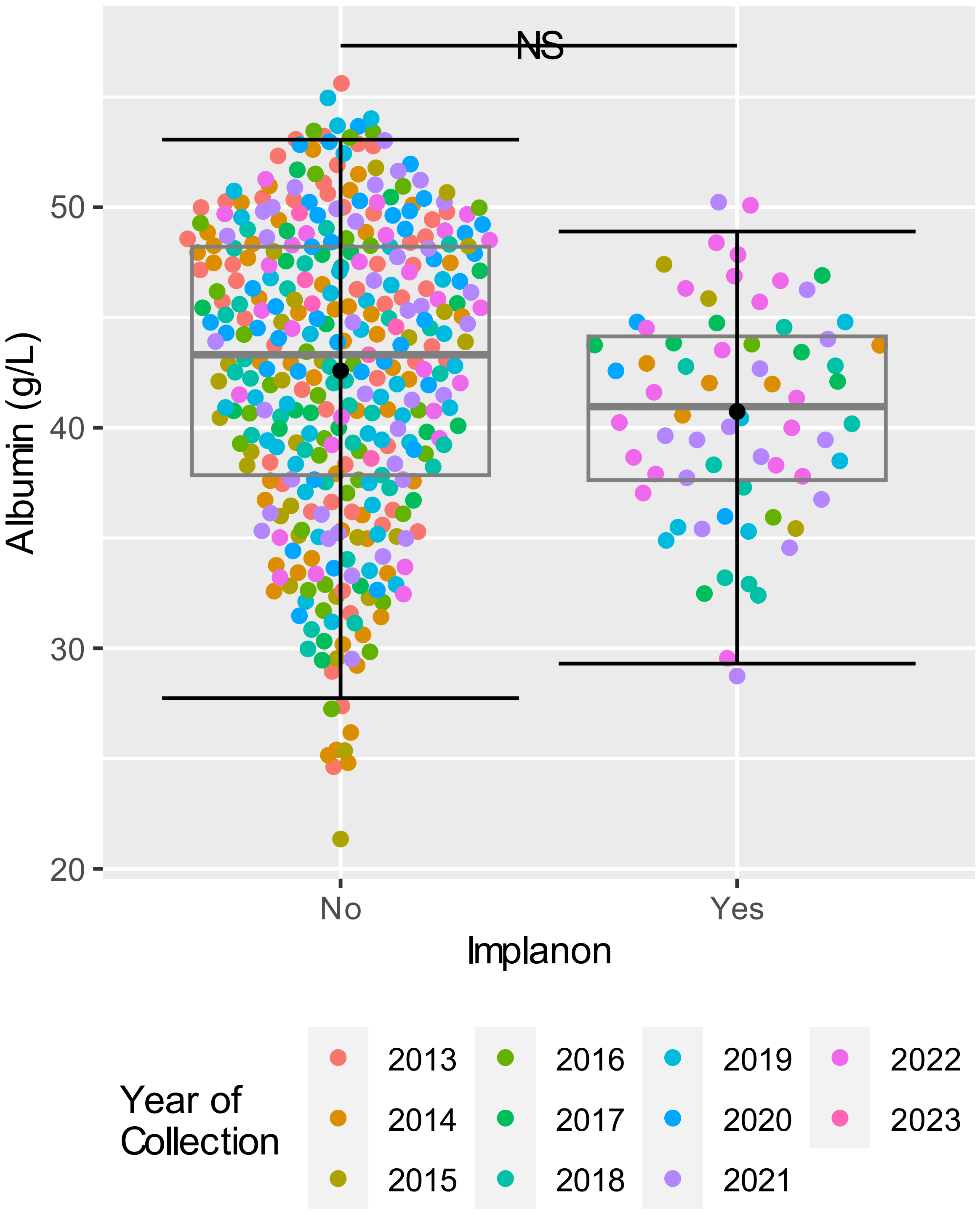

Figure S5 1. Albumin levels according to implanon status in adult female common marmosets. Boxes indicate median and quartile ranges; black point indicates arithmetic mean and error bars indicate the normal range. Symbol colours indicate year of collection; asterisks indicate statistical significance levels NS = not significant, * < 0.001, ** < 0.0001, *** < 0.00001, **** < 0.000001

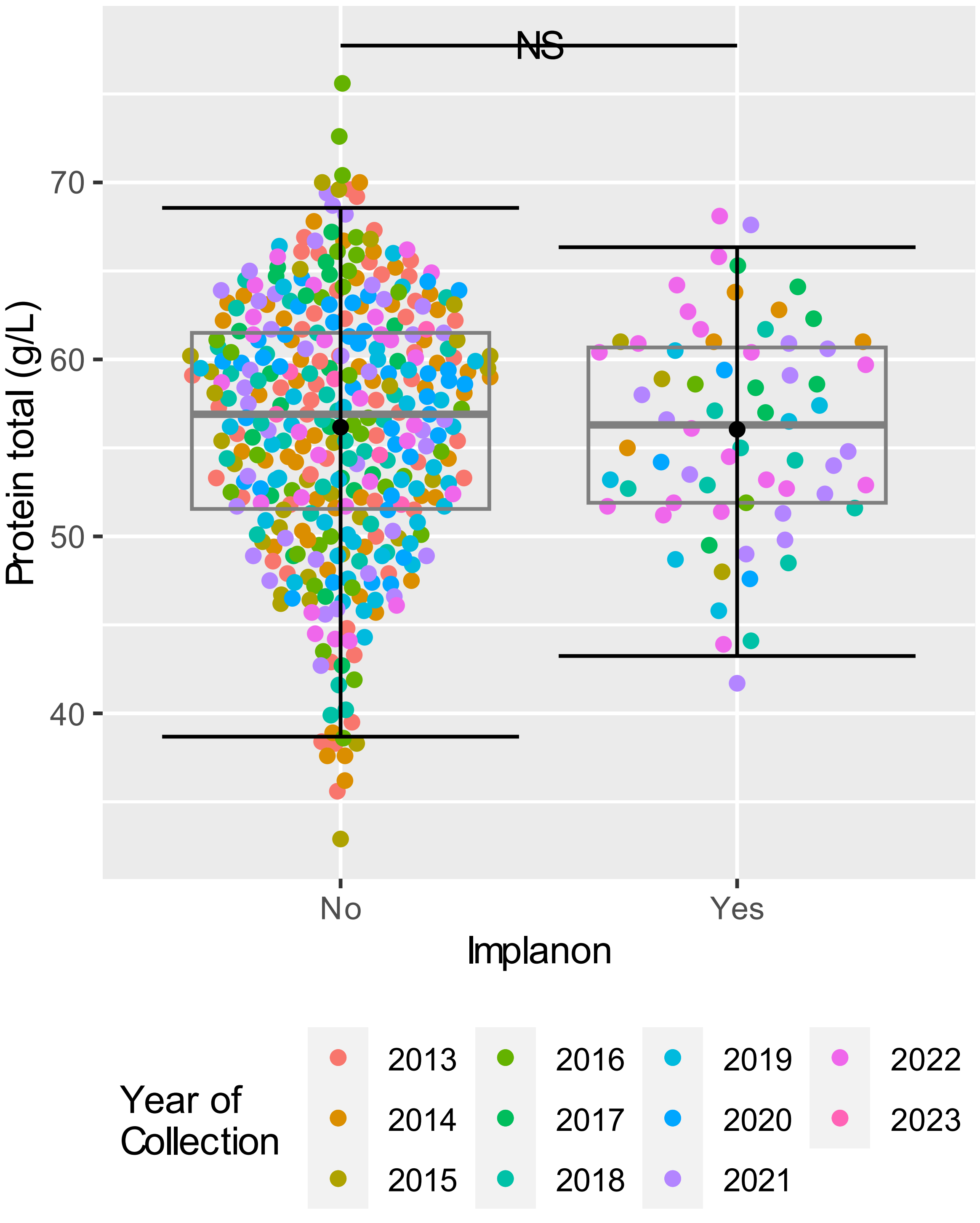

Figure S5 2. Total Protein levels according to implanon status in adult female common marmosets. Boxes indicate median and quartile ranges; black point indicates arithmetic mean and error bars indicate the normal range. Symbol colours indicate year of collection; asterisks indicate statistical significance levels NS = not significant, NS = not significant, * < 0.001, ** < 0.0001, *** < 0.00001, **** < 0.000001

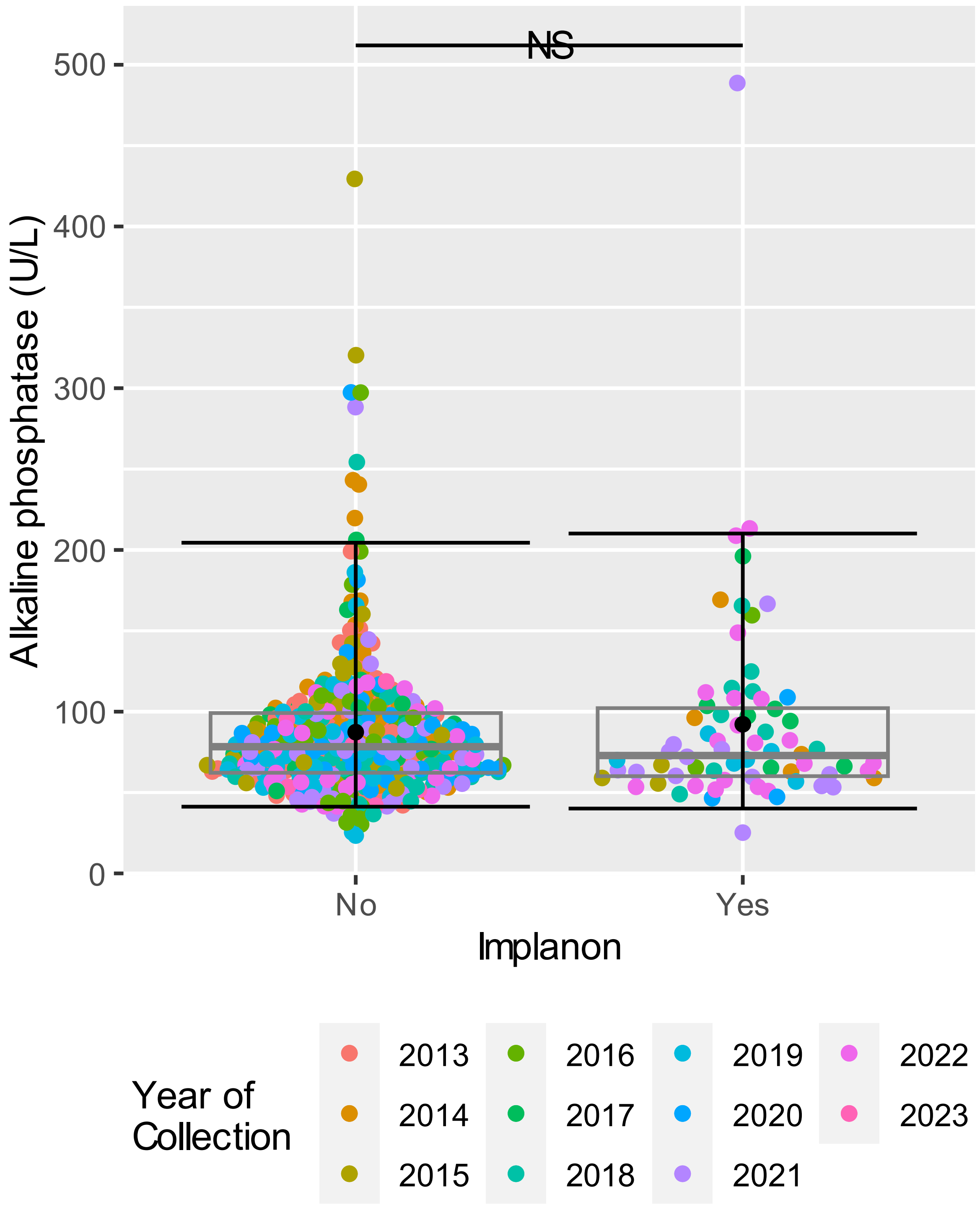

Figure S5 3. Alkaline Phosphatase levels according to implanon status in adult female common marmosets. Boxes indicate median and quartile ranges; black point indicates arithmetic mean and error bars indicate the normal range. Symbol colours indicate year of collection; asterisks indicate statistical significance levels NS = not significant, NS = not significant, * < 0.001, ** < 0.0001, *** < 0.00001, **** < 0.000001

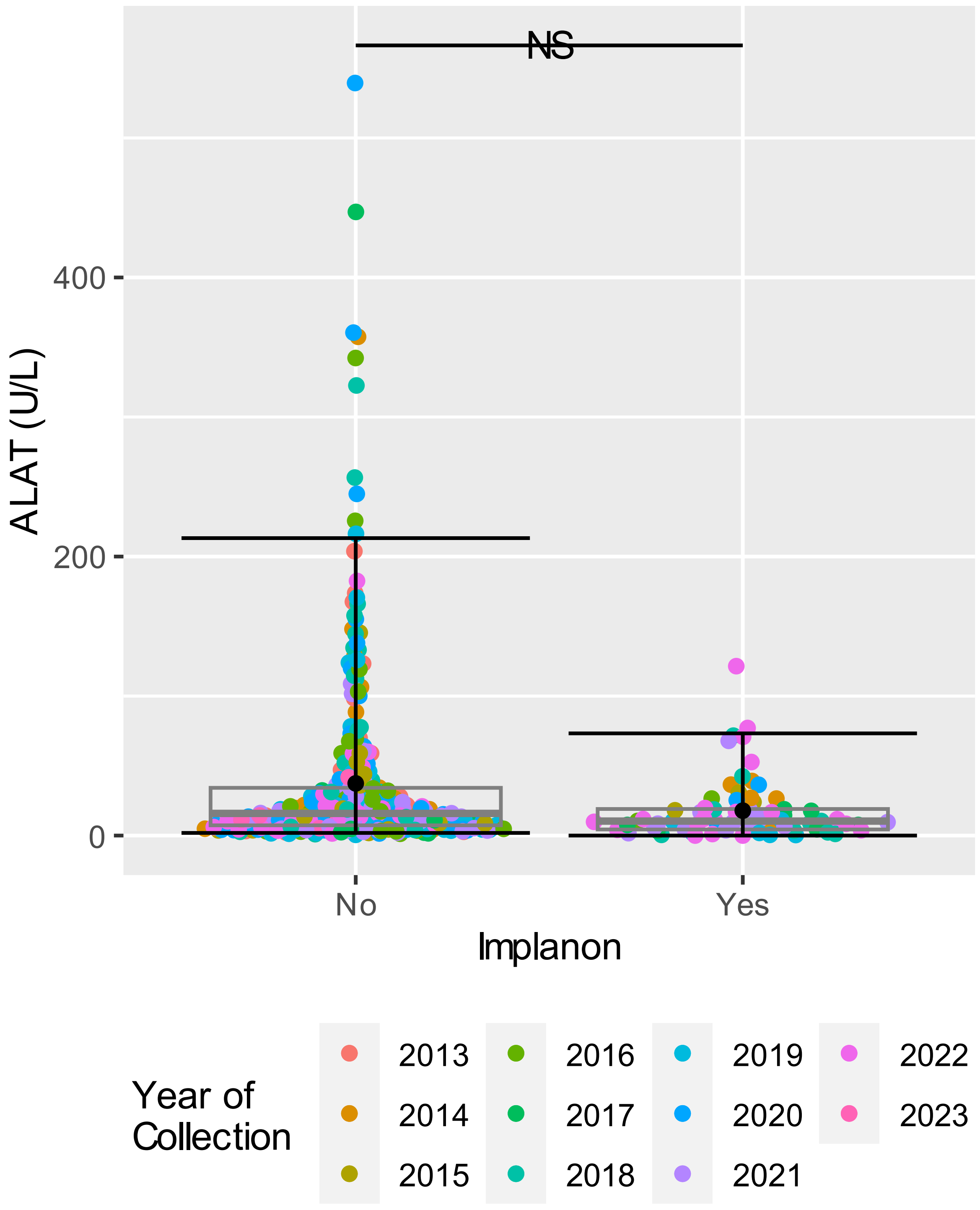

Figure S5 4. ALAT levels according to implanon status in adult female common marmosets. Boxes indicate median and quartile ranges; black point indicates arithmetic mean and error bars indicate the normal range. Symbol colours indicate year of collection; asterisks indicate statistical significance levels NS = not significant, NS = not significant, * < 0.001, ** < 0.0001, *** < 0.00001, **** < 0.000001

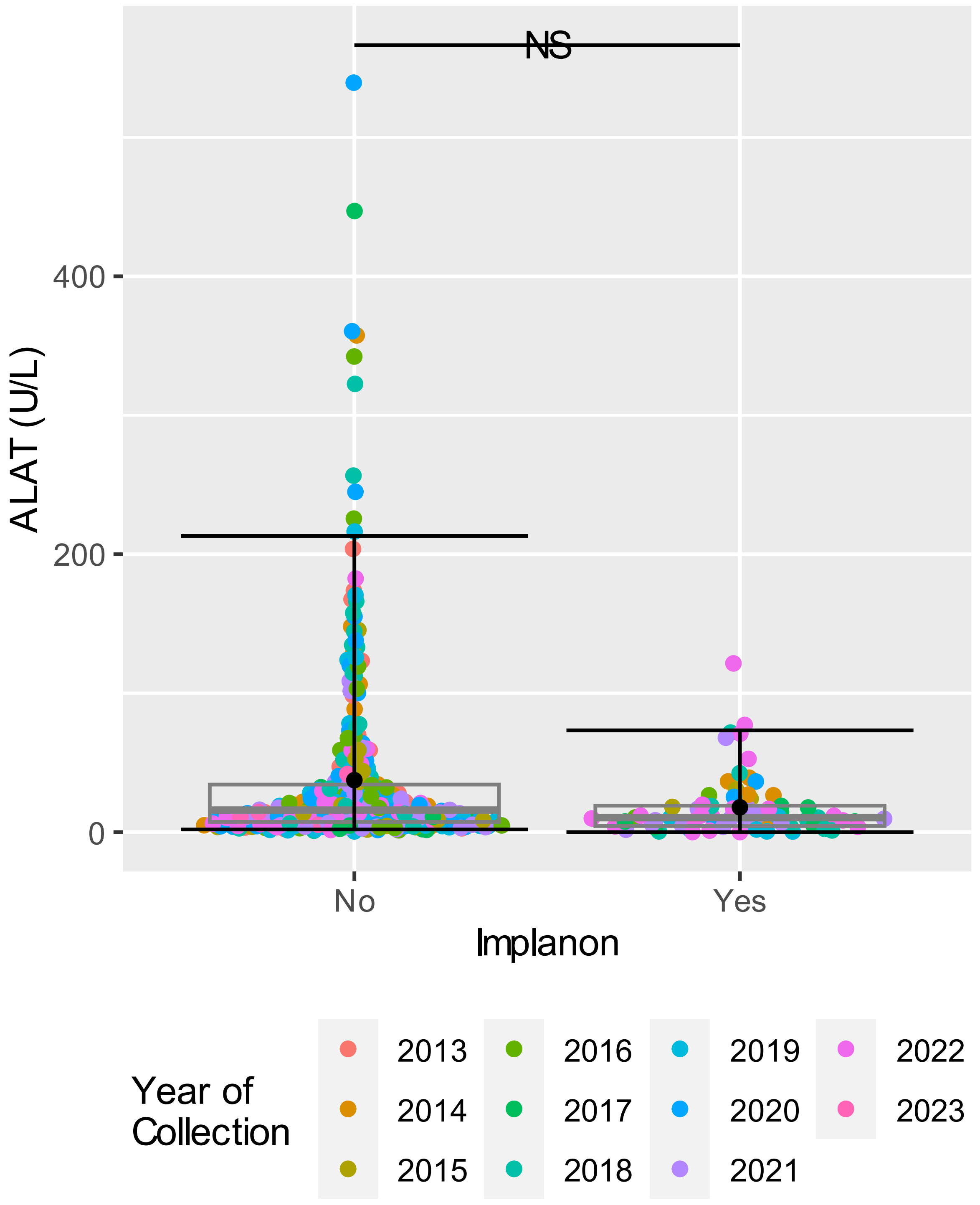

Figure S5 5. ASAT levels according to implanon status in adult female common marmosets. Boxes indicate median and quartile ranges; black point indicates arithmetic mean and error bars indicate the normal range. Symbol colours indicate year of collection; asterisks indicate statistical significance levels NS = not significant, NS = not significant, * < 0.001, ** < 0.0001, *** < 0.00001, **** < 0.000001

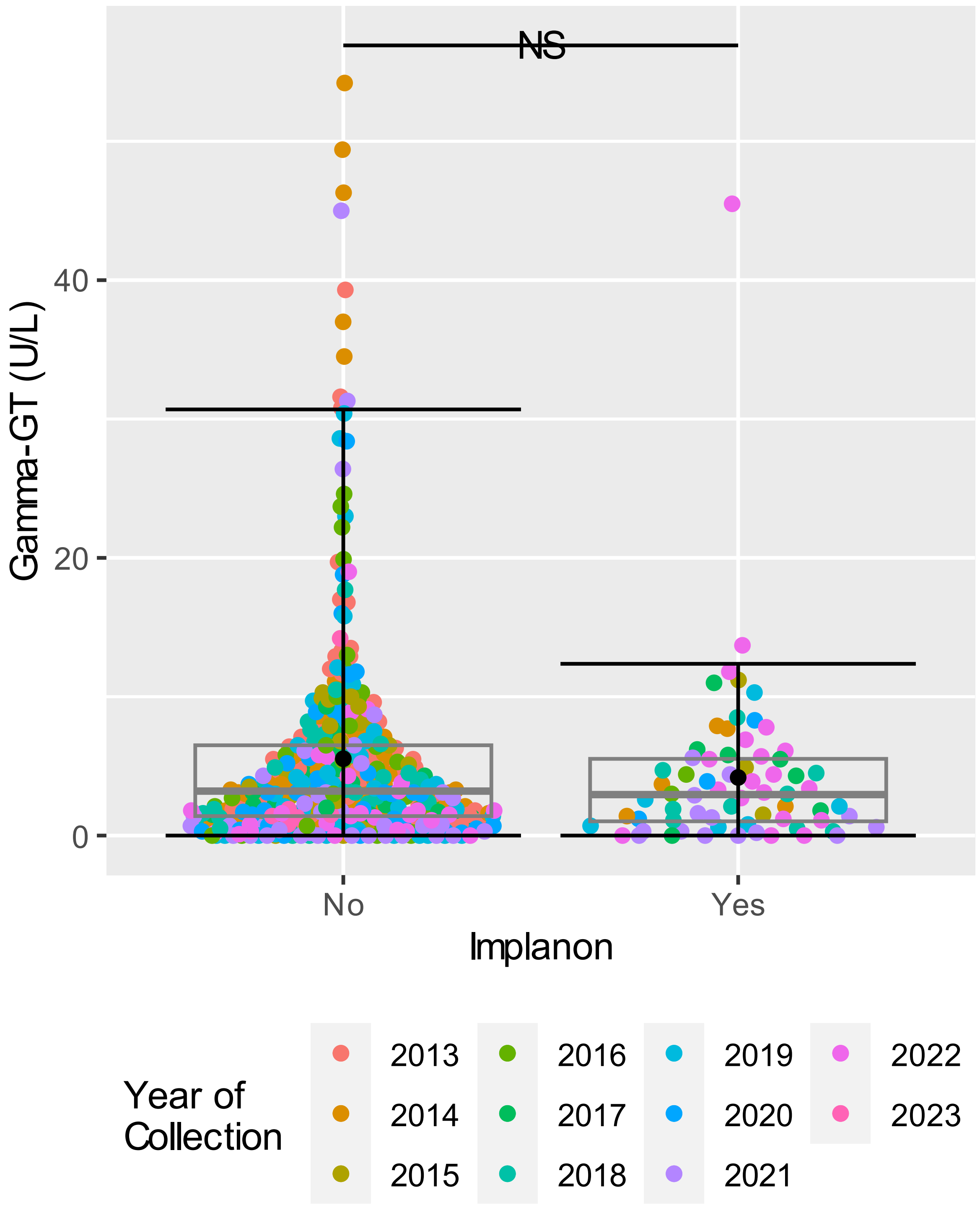

Figure S5 6. Gamma-GT levels according to implanon status in adult female common marmosets. Boxes indicate median and quartile ranges; black point indicates arithmetic mean and error bars indicate the normal range. Symbol colours indicate year of collection; asterisks indicate statistical significance levels NS = not significant, NS = not significant, * < 0.001, ** < 0.0001, *** < 0.00001, **** < 0.000001

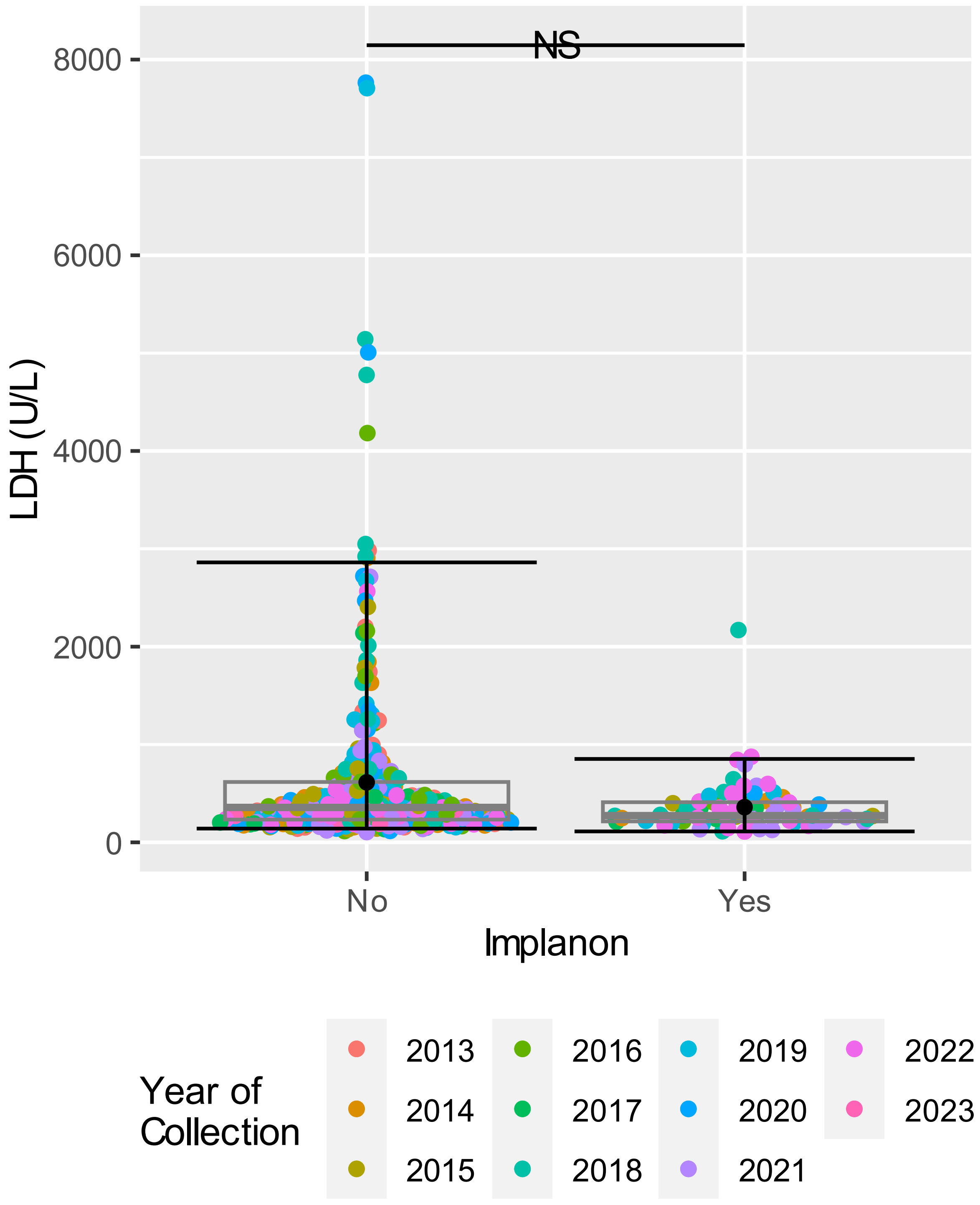

Figure S5 7. LDH levels according to implanon status in adult female common marmosets. Boxes indicate median and quartile ranges; black point indicates arithmetic mean and error bars indicate the normal range. Symbol colours indicate year of collection; asterisks indicate statistical significance levels NS = not significant, NS = not significant, * < 0.001, ** < 0.0001, *** < 0.00001, **** < 0.000001

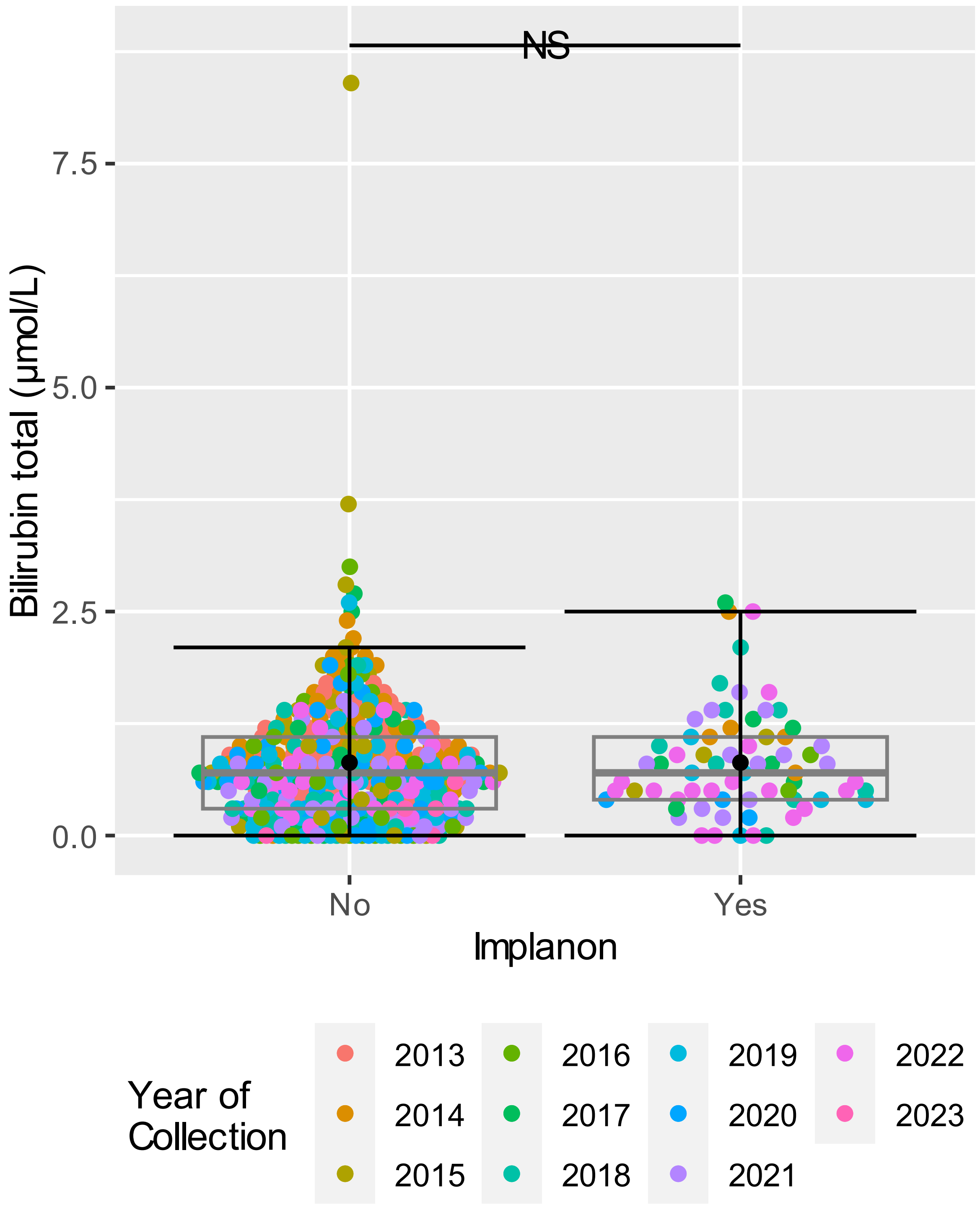

Figure S5 8. Total Bilirubin levels according to implanon status in adult female common marmosets. Boxes indicate median and quartile ranges; black point indicates arithmetic mean and error bars indicate the normal range. Symbol colours indicate year of collection; asterisks indicate statistical significance levels NS = not significant, NS = not significant, * < 0.001, ** < 0.0001, *** < 0.00001, **** < 0.000001

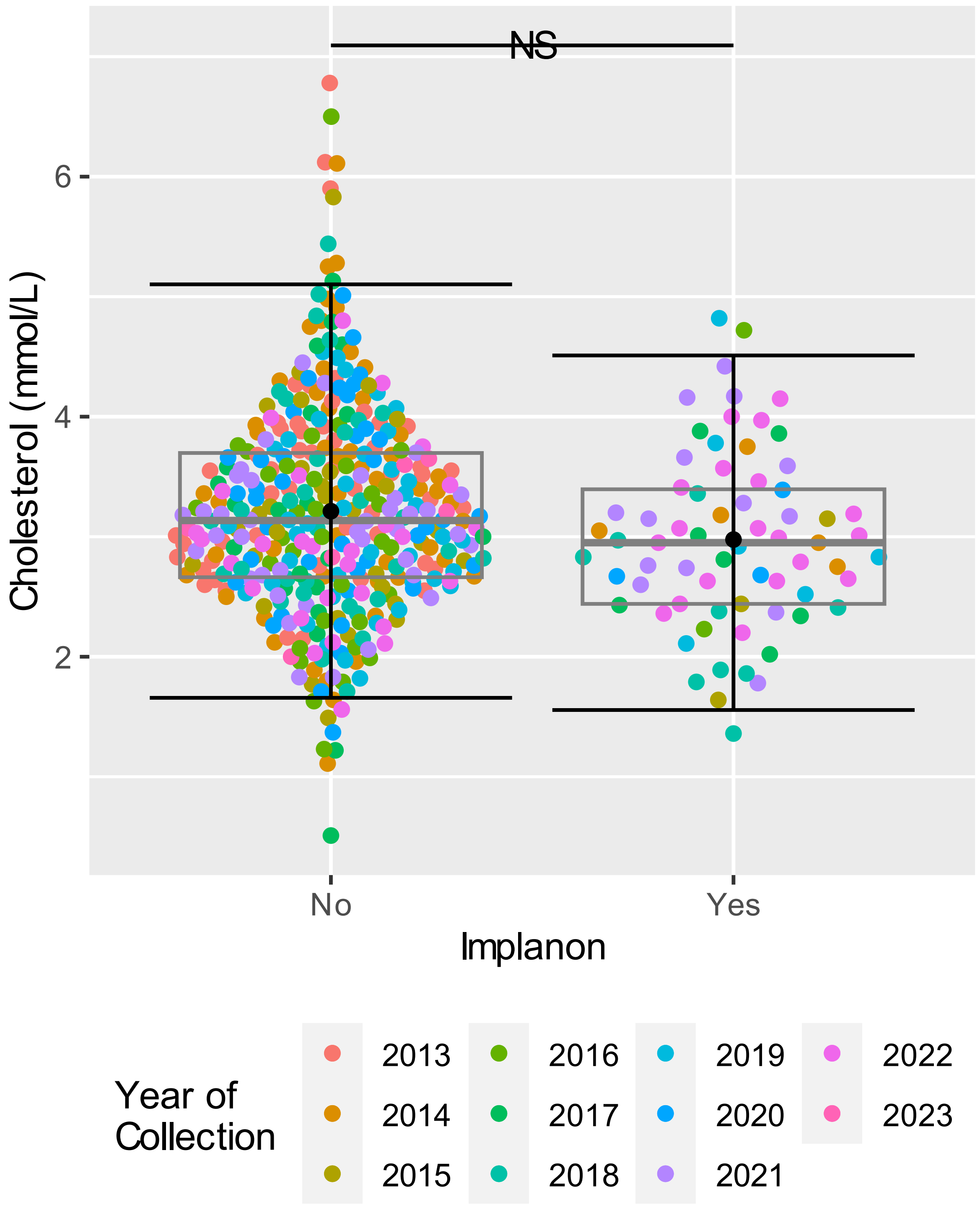

Figure S5 9. Cholesterol levels according to implanon status in adult female common marmosets. Boxes indicate median and quartile ranges; black point indicates arithmetic mean and error bars indicate the normal range. Symbol colours indicate year of collection; asterisks indicate statistical significance levels NS = not significant, NS = not significant, * < 0.001, ** < 0.0001, *** < 0.00001, **** < 0.000001

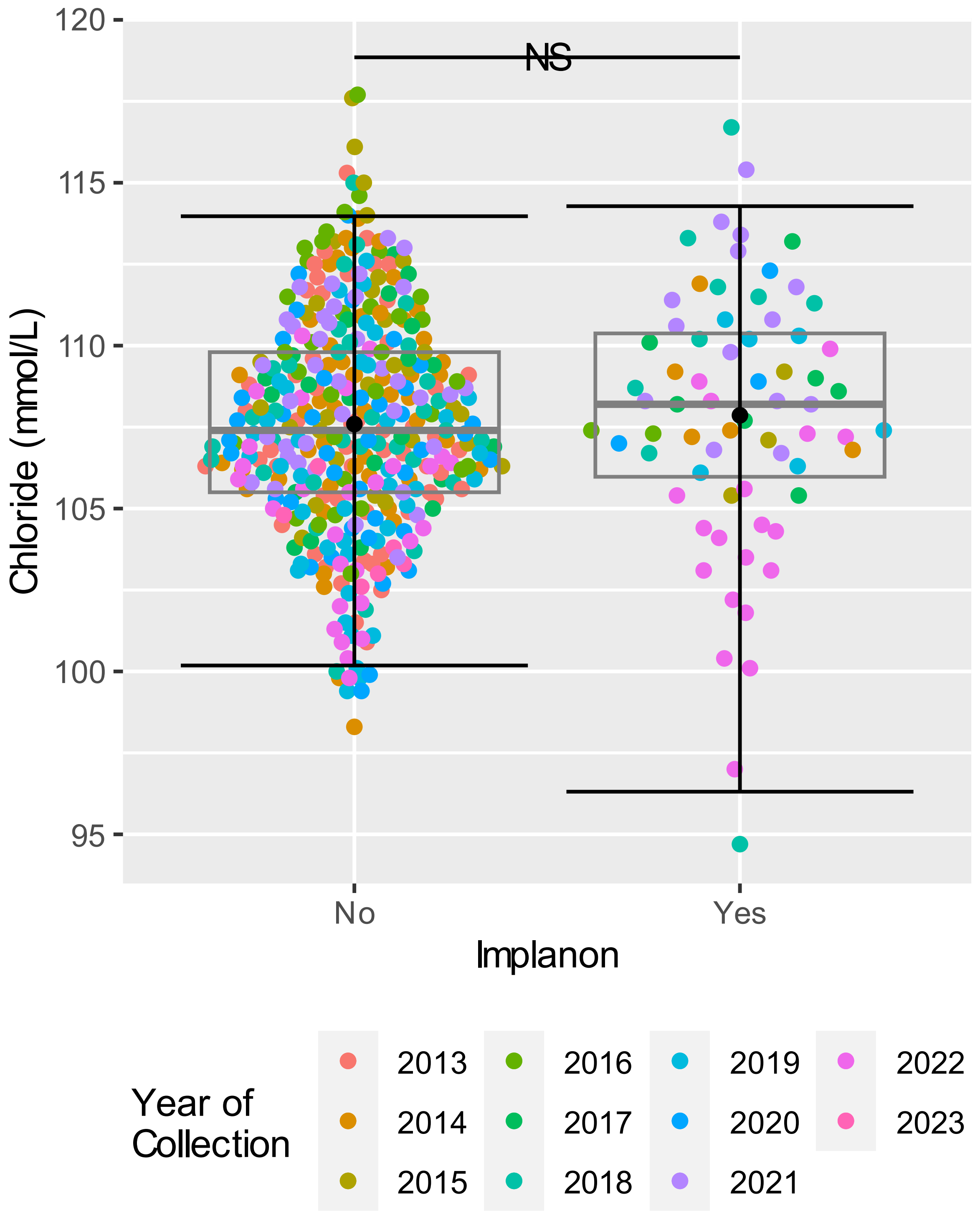

Figure S5 10. Chloride levels according to implanon status in adult female common marmosets. Boxes indicate median and quartile ranges; black point indicates arithmetic mean and error bars indicate the normal range. Symbol colours indicate year of collection; asterisks indicate statistical significance levels NS = not significant, NS = not significant, * < 0.001, ** < 0.0001, *** < 0.00001, **** < 0.000001

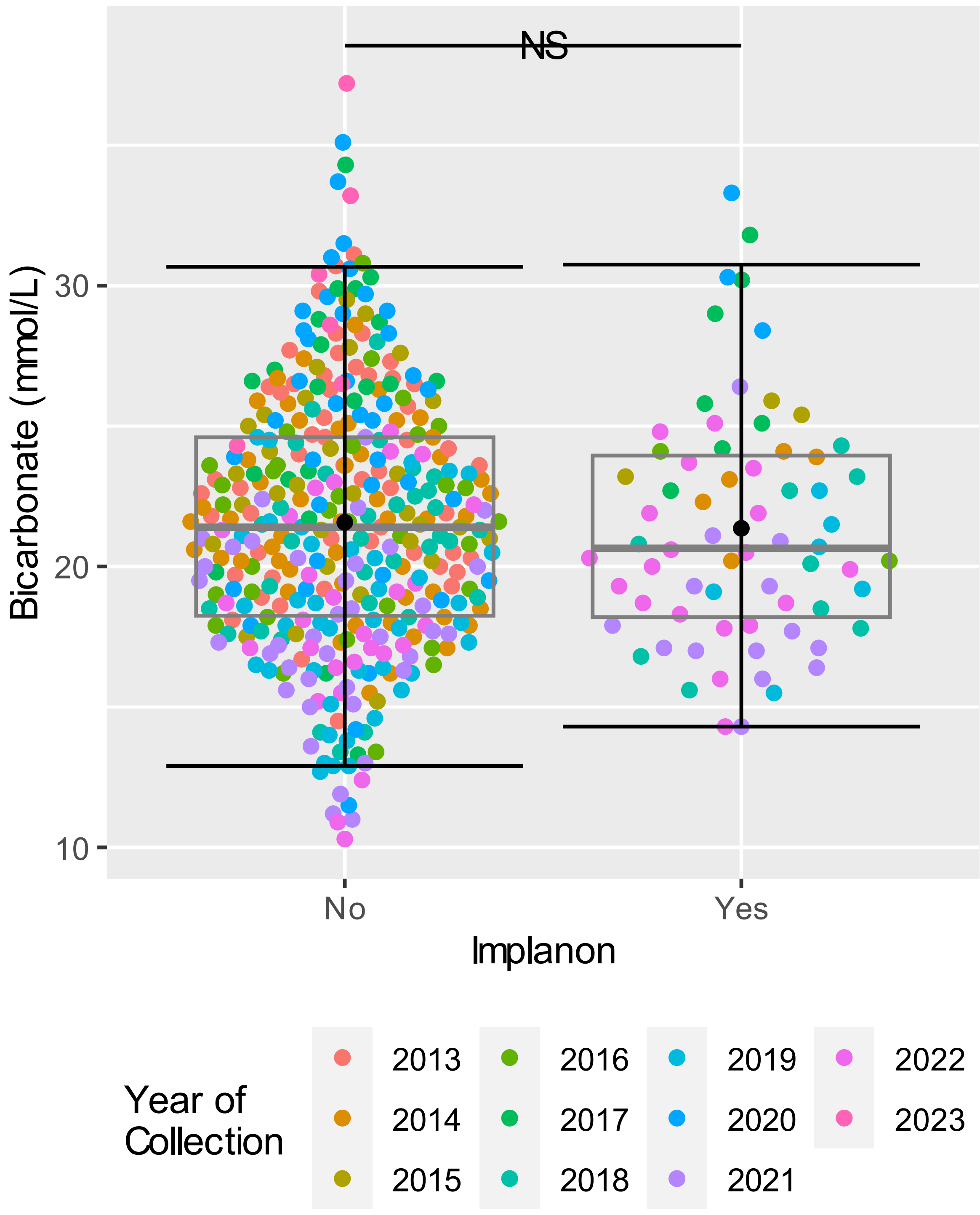

Figure S5 11. Bicarbonate levels according to implanon status in adult female common marmosets. Boxes indicate median and quartile ranges; black point indicates arithmetic mean and error bars indicate the normal range. Symbol colours indicate year of collection; asterisks indicate statistical significance levels NS = not significant, NS = not significant, * < 0.001, ** < 0.0001, *** < 0.00001, **** < 0.000001

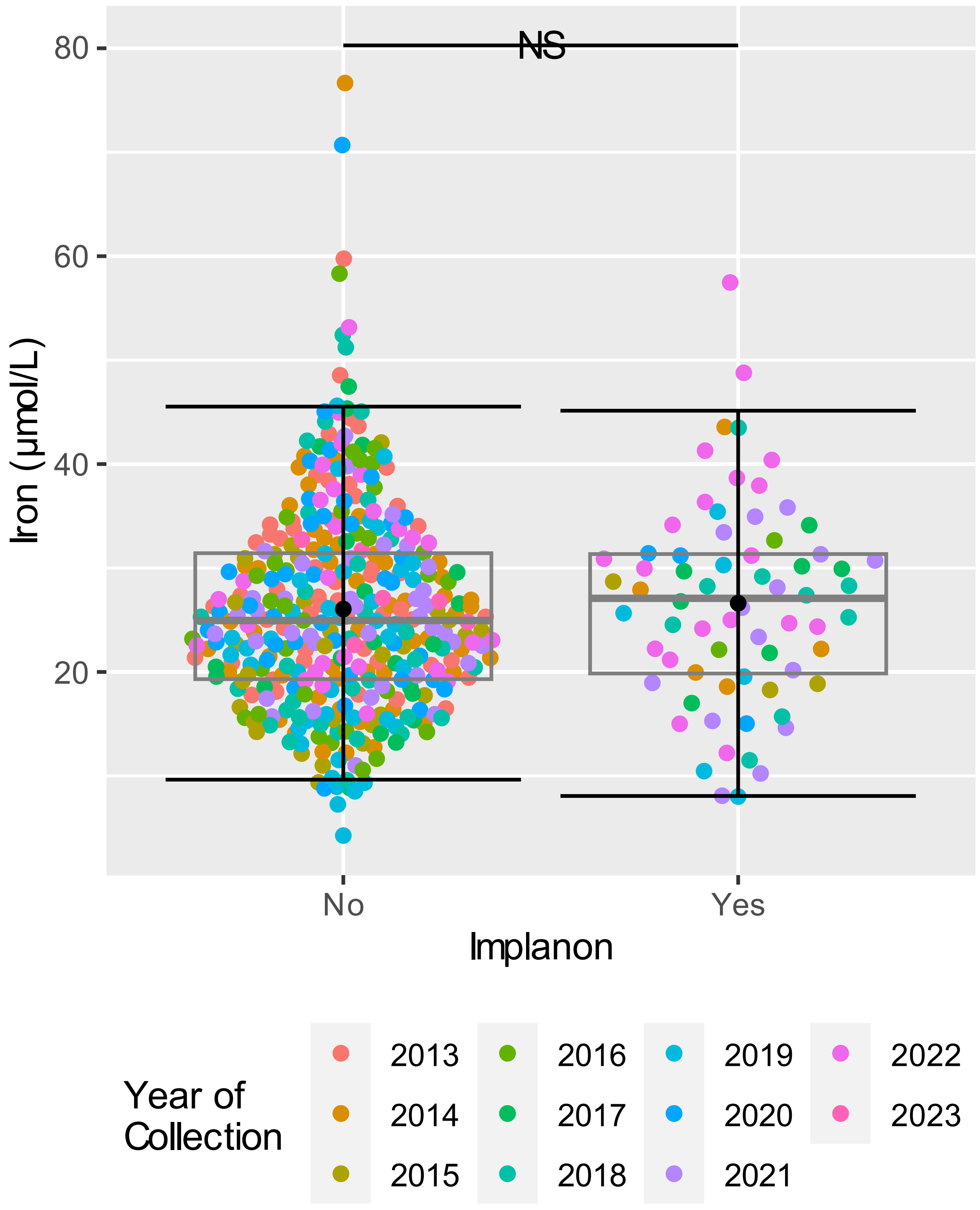

Figure S5 12. Iron levels according to implanon status in adult female common marmosets. Boxes indicate median and quartile ranges; black point indicates arithmetic mean and error bars indicate the normal range. Symbol colours indicate year of collection; asterisks indicate statistical significance levels NS = not significant, NS = not significant, * < 0.001, ** < 0.0001, *** < 0.00001, **** < 0.000001

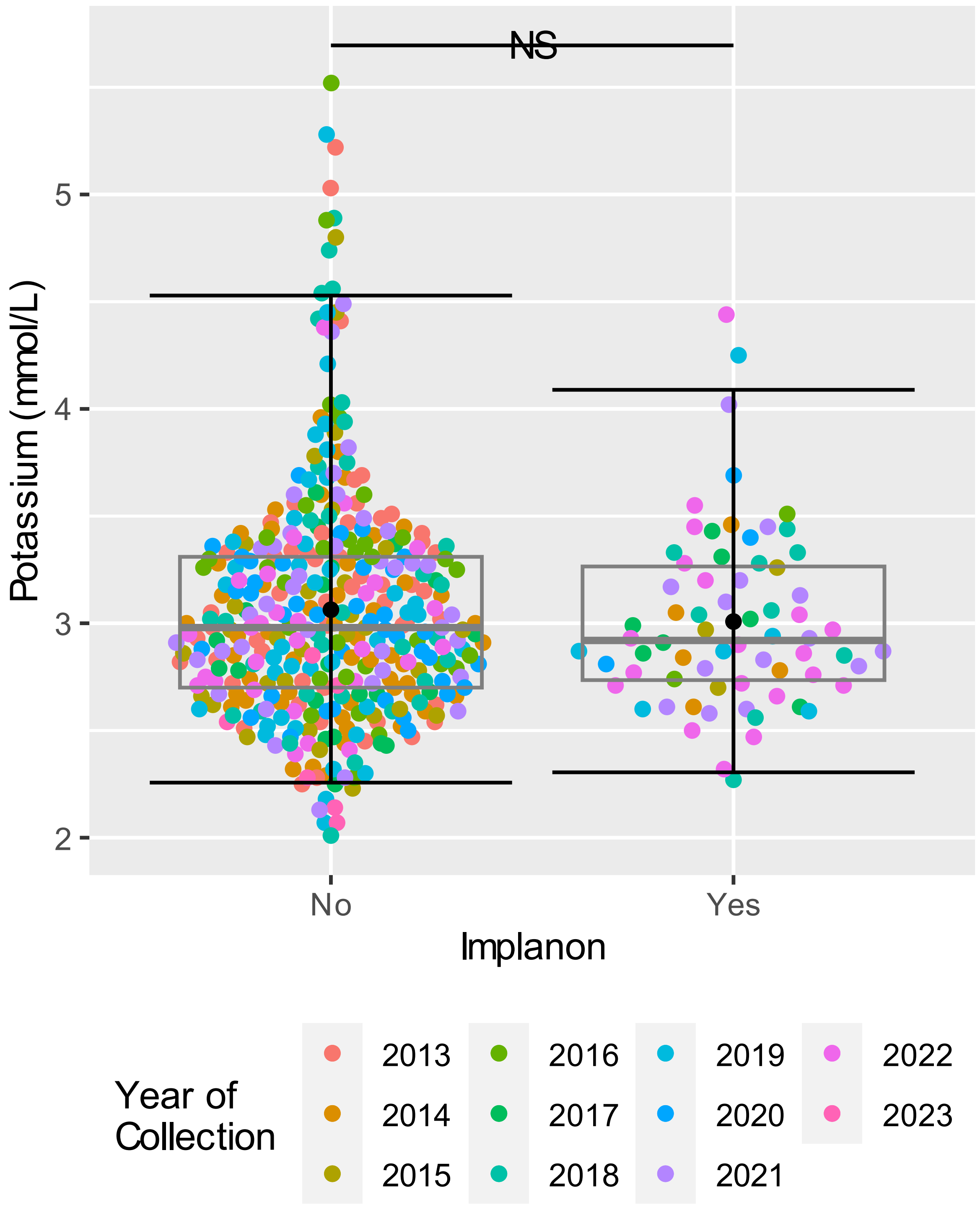

Figure S5 13. Potassium levels according to implanon status in adult female common marmosets. Boxes indicate median and quartile ranges; black point indicates arithmetic mean and error bars indicate the normal range. Symbol colours indicate year of collection; asterisks indicate statistical significance levels NS = not significant, NS = not significant, * < 0.001, ** < 0.0001, *** < 0.00001, **** < 0.000001

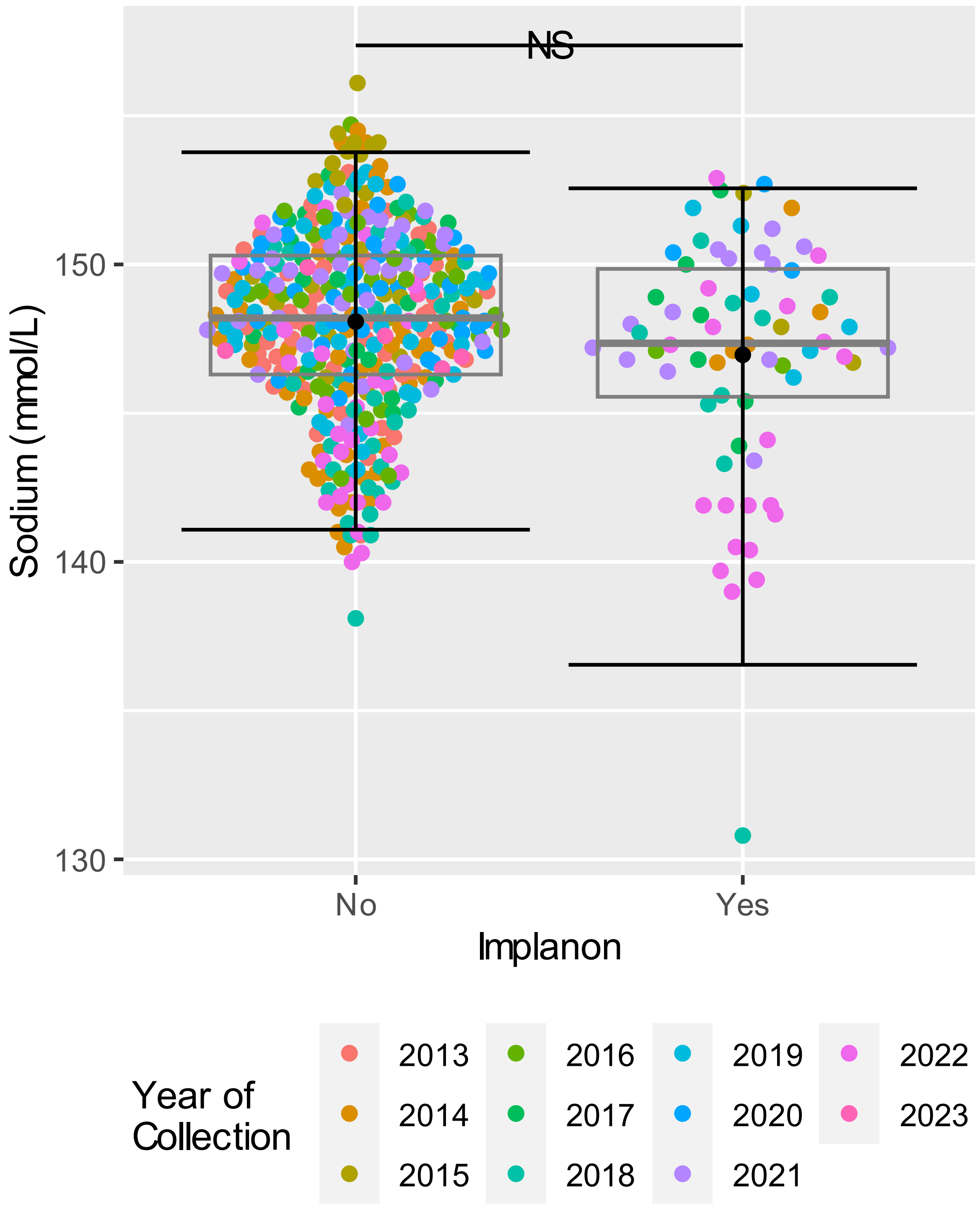

Figure S5 14. Sodium levels according to implanon status in adult female common marmosets. Boxes indicate median and quartile ranges; black point indicates arithmetic mean and error bars indicate the normal range. Symbol colours indicate year of collection; asterisks indicate statistical significance levels NS = not significant, NS = not significant, * < 0.001, ** < 0.0001, *** < 0.00001, **** < 0.000001

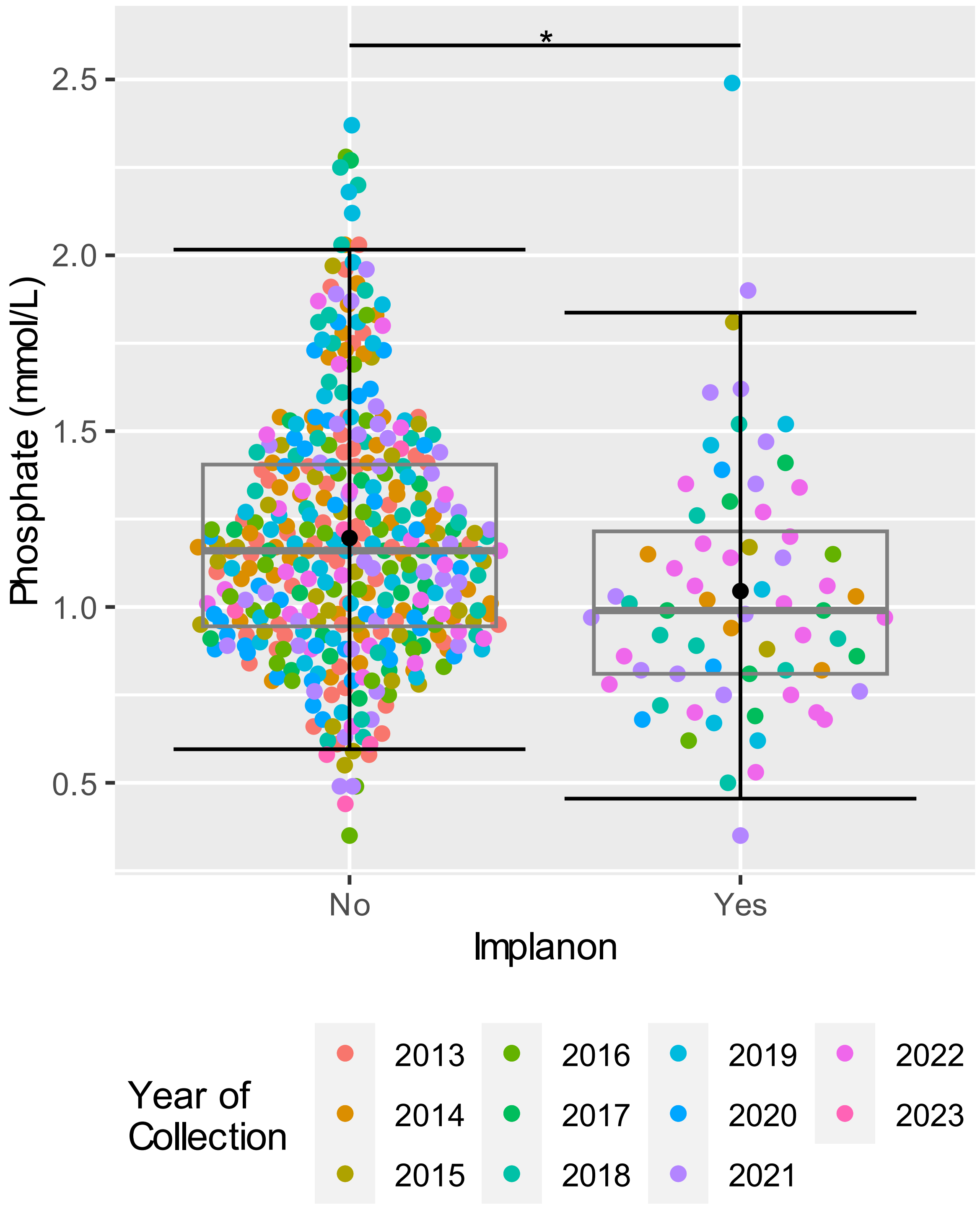

Figure S5 15. Phosphate levels according to implanon status in adult female common marmosets. Boxes indicate median and quartile ranges; black point indicates arithmetic mean and error bars indicate the normal range. Symbol colours indicate year of collection; asterisks indicate statistical significance levels NS = not significant, NS = not significant, * < 0.001, ** < 0.0001, *** < 0.00001, **** < 0.000001

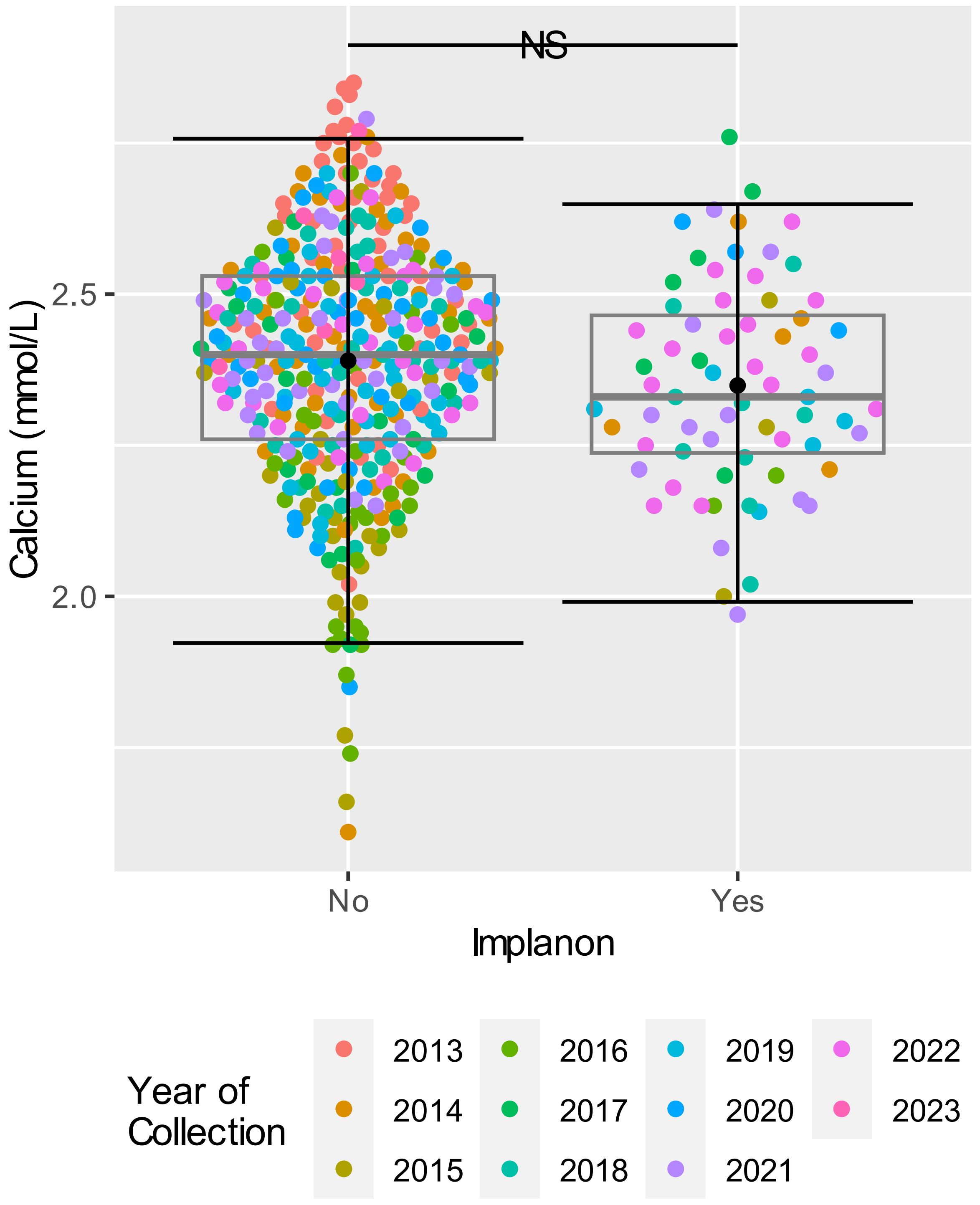

Figure S5 16. Calcium levels according to implanon status in adult female common marmosets. Boxes indicate median and quartile ranges; black point indicates arithmetic mean and error bars indicate the normal range. Symbol colours indicate year of collection; asterisks indicate statistical significance levels NS = not significant, * < 0.001, ** < 0.0001, *** < 0.00001, **** < 0.000001

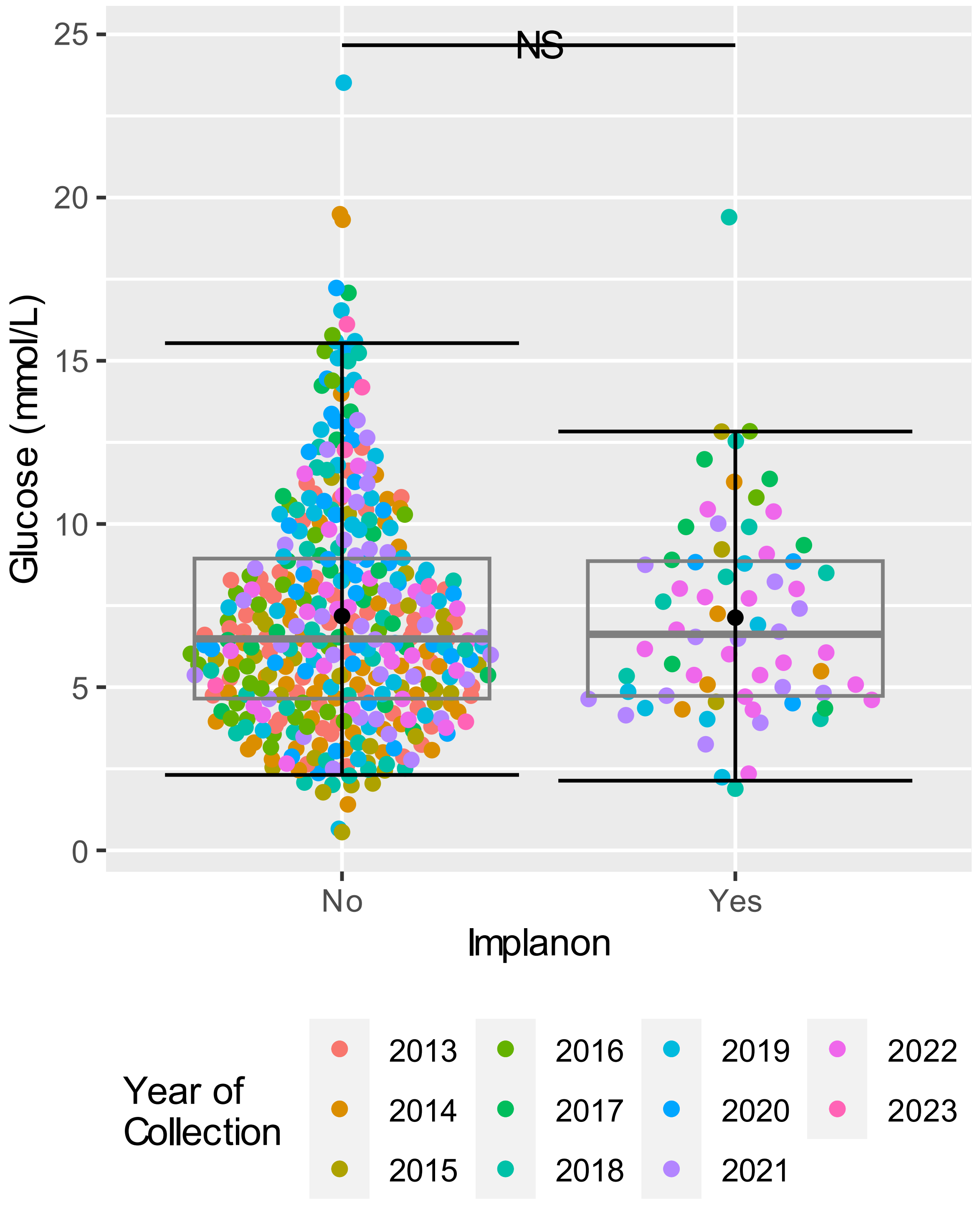

Figure S5 17. Glucose levels according to implanon status in adult female common marmosets. Boxes indicate median and quartile ranges; black point indicates arithmetic mean and error bars indicate the normal range. Symbol colours indicate year of collection; asterisks indicate statistical significance levels NS = not significant, * < 0.001, ** < 0.0001, *** < 0.00001, **** < 0.000001

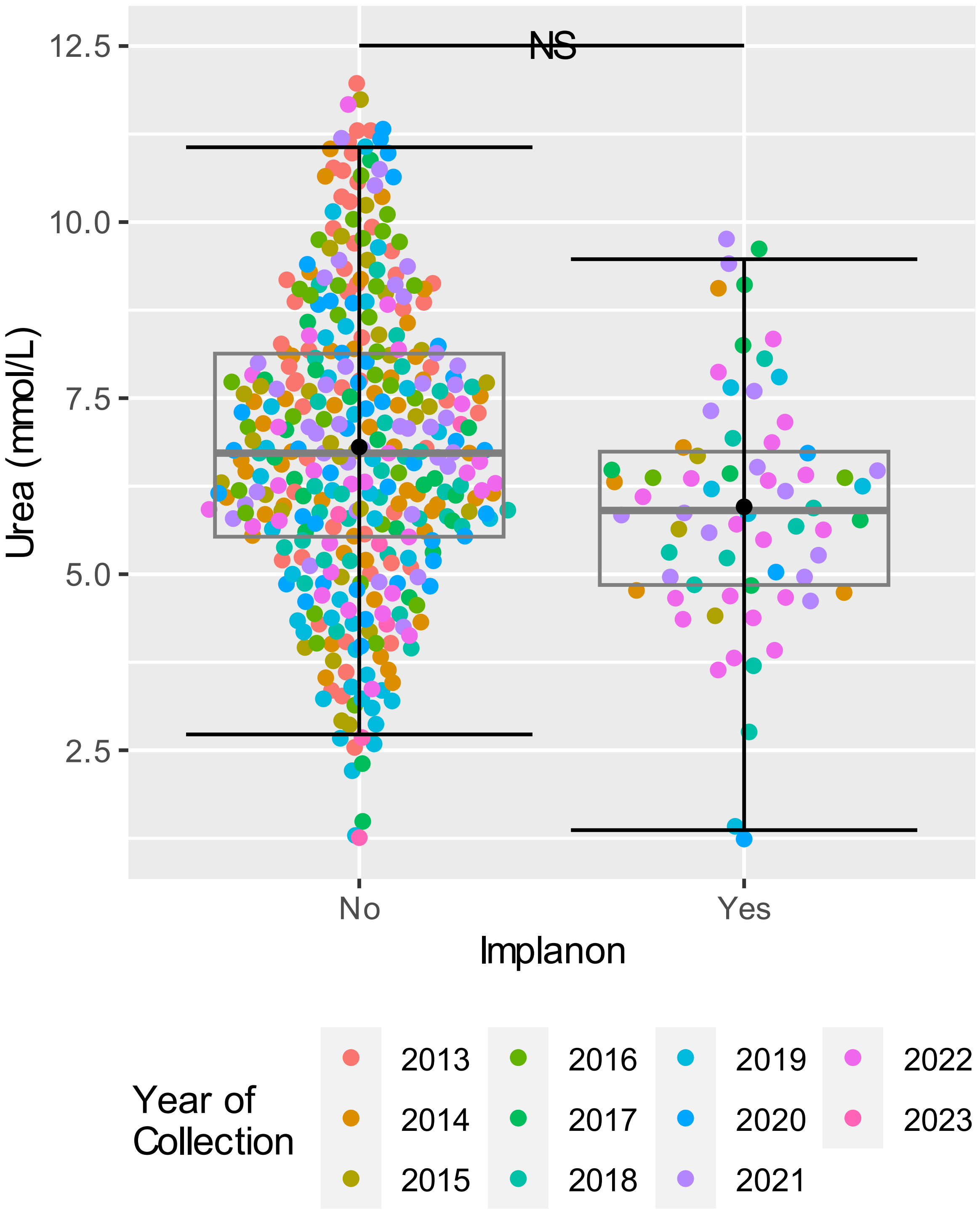

Figure S5 18. Urea levels according to implanon status in adult female common marmosets. Boxes indicate median and quartile ranges; black point indicates arithmetic mean and error bars indicate the normal range. Symbol colours indicate year of collection; asterisks indicate statistical significance levels NS = not significant, * < 0.001, ** < 0.0001, *** < 0.00001, **** < 0.000001

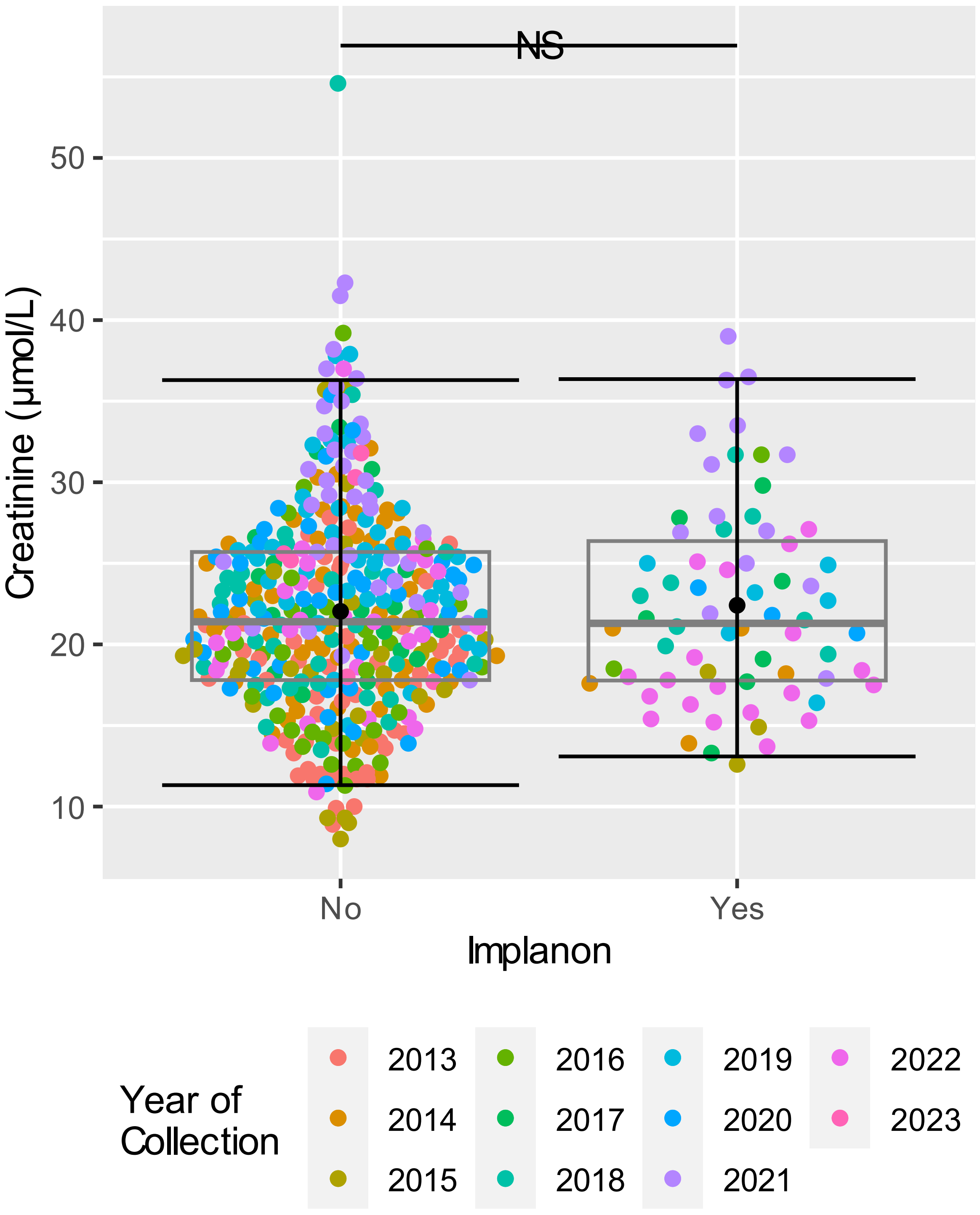

Figure S5 19. Creatinine levels according to implanon status in adult female common marmosets. Boxes indicate median and quartile ranges; black point indicates arithmetic mean and error bars indicate the normal range. Symbol colours indicate year of collection; asterisks indicate statistical significance levels NS = not significant, * < 0.001, ** < 0.0001, *** < 0.00001, **** < 0.000001

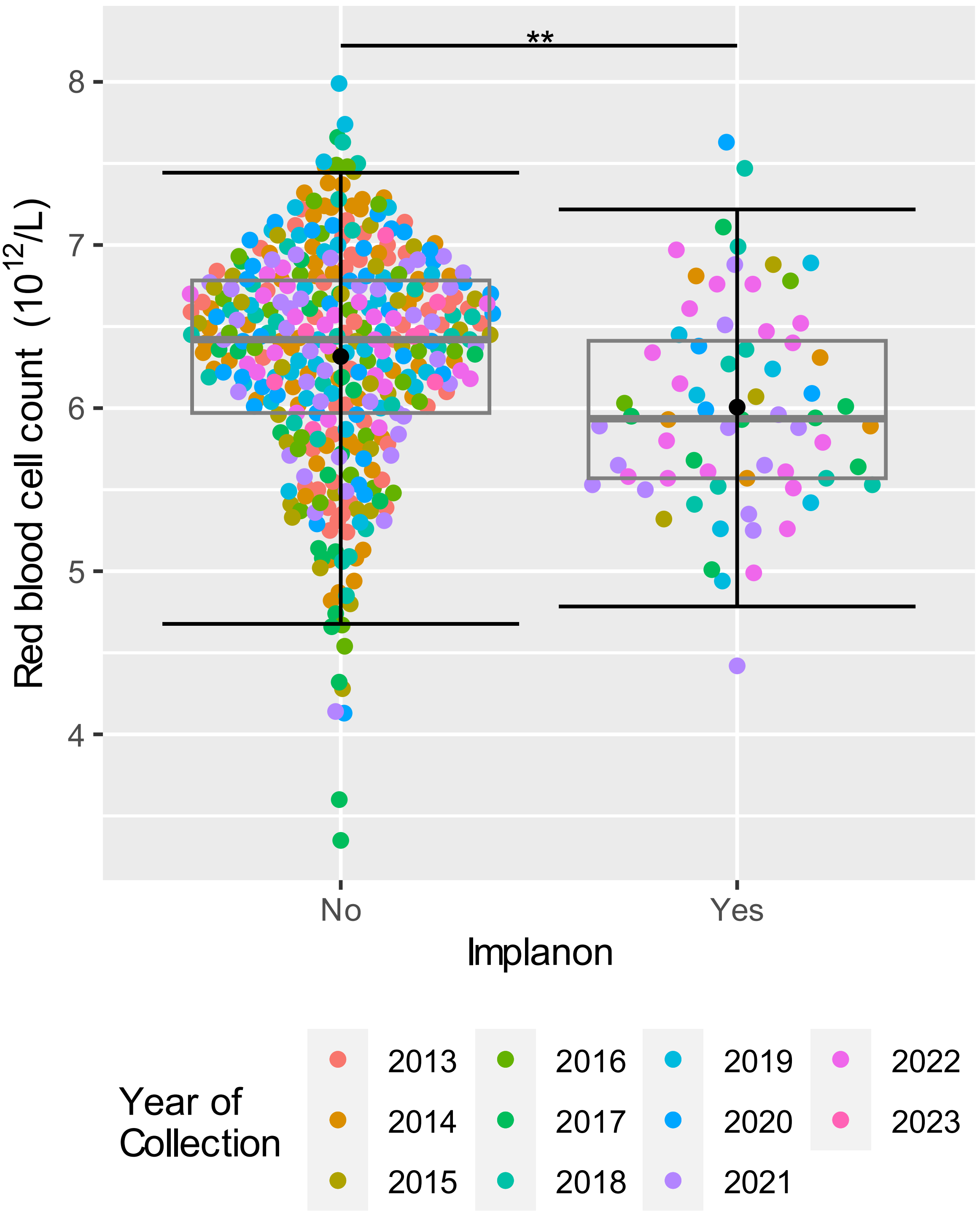

Figure S5 20. Red blood cell count according to implanon status in adult female common marmosets. Boxes indicate median and quartile ranges; black point indicates arithmetic mean and error bars indicate the normal range. Symbol colours indicate year of collection; asterisks indicate statistical significance levels NS = not significant, * < 0.001, ** < 0.0001, *** < 0.00001, **** < 0.000001

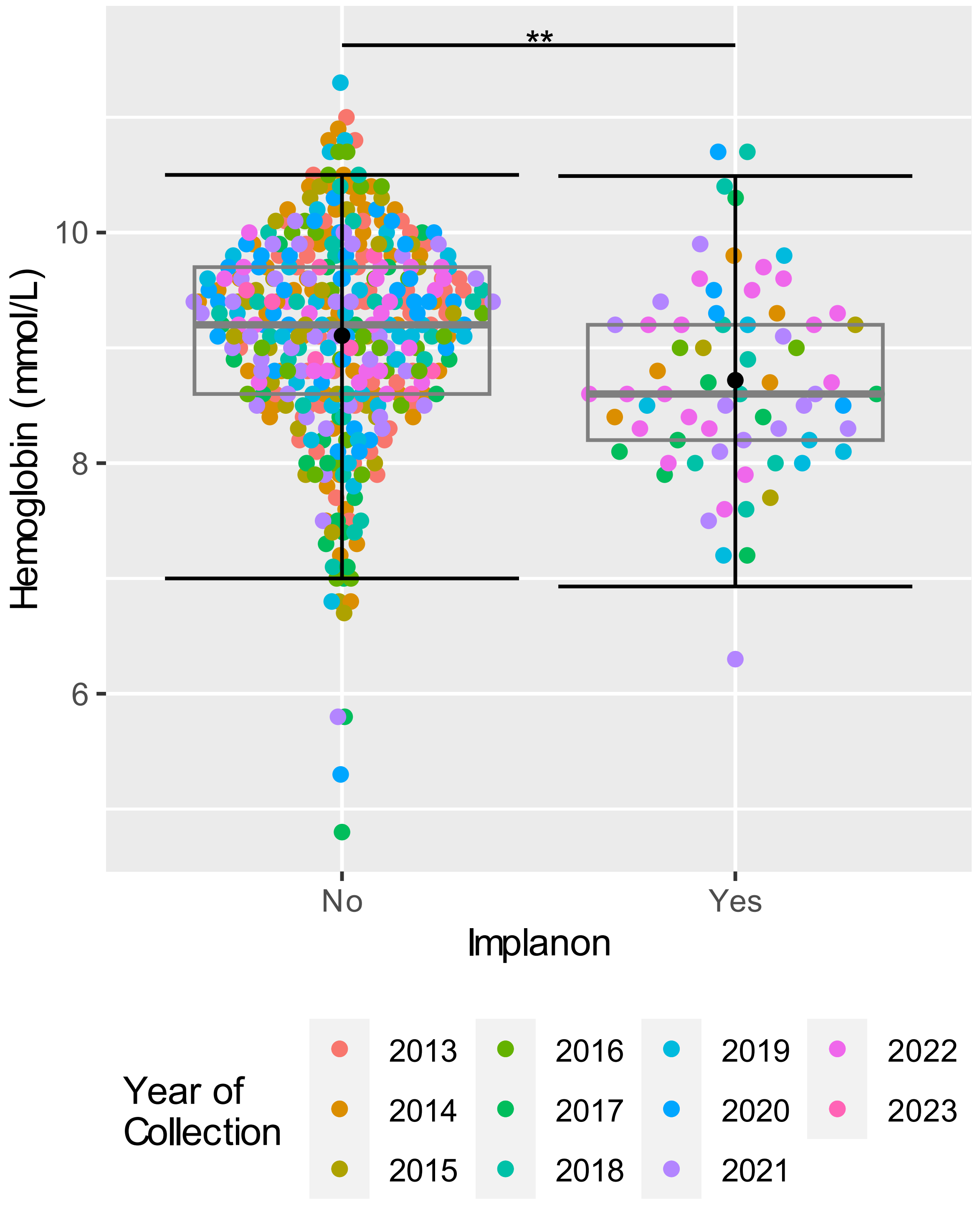

Figure S5 21. Haemoglobin levels according to implanon status in adult female common marmosets. Boxes indicate median and quartile ranges; black point indicates arithmetic mean and error bars indicate the normal range. Symbol colours indicate year of collection; asterisks indicate statistical significance levels NS = not significant, * < 0.001, ** < 0.0001, *** < 0.00001, **** < 0.000001

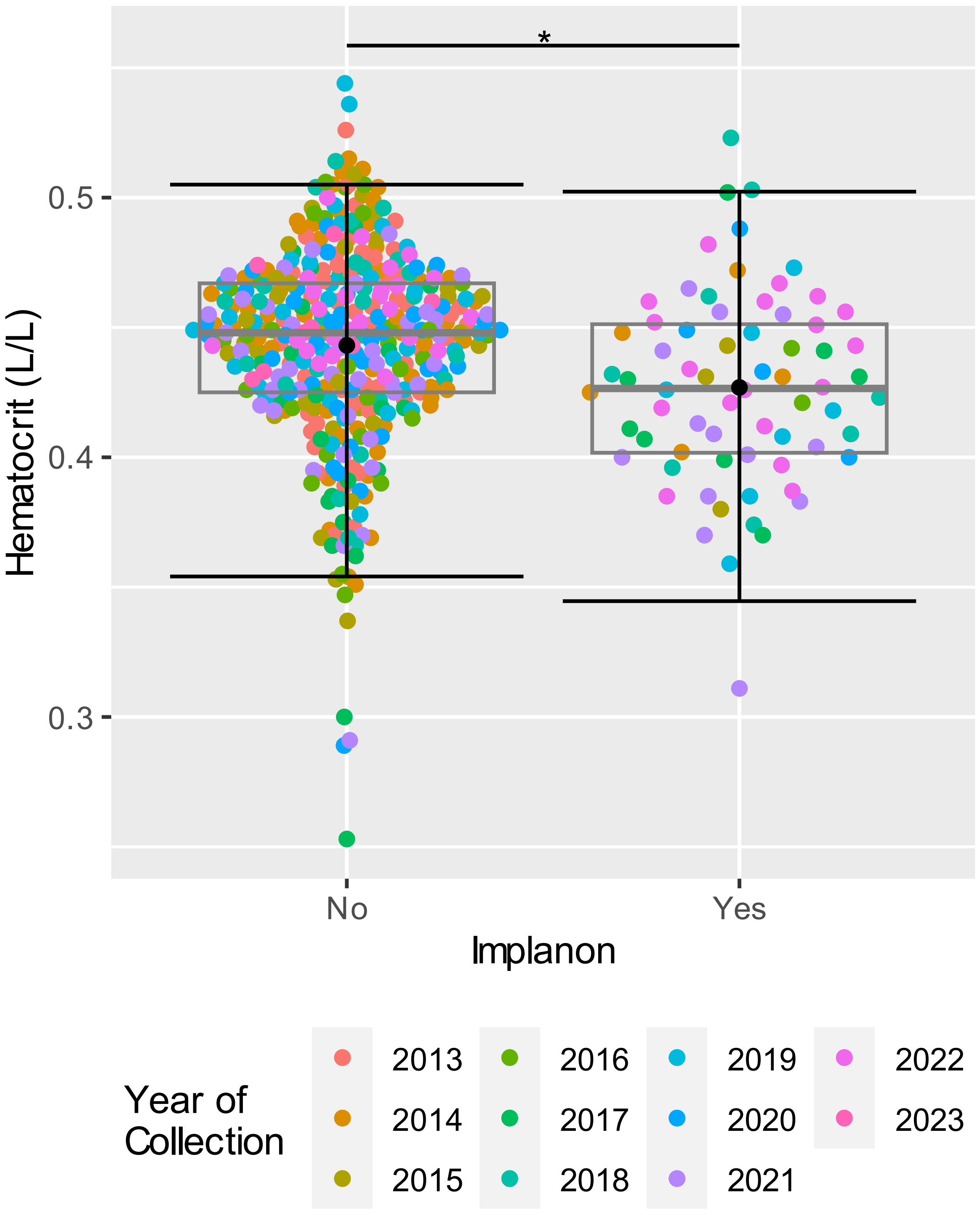

Figure S5 22. Haematocrit according to implanon status in adult female common marmosets. Boxes indicate median and quartile ranges; black point indicates arithmetic mean and error bars indicate the normal range. Symbol colours indicate year of collection; asterisks indicate statistical significance levels NS = not significant, * < 0.001, ** < 0.0001, *** < 0.00001, **** < 0.000001

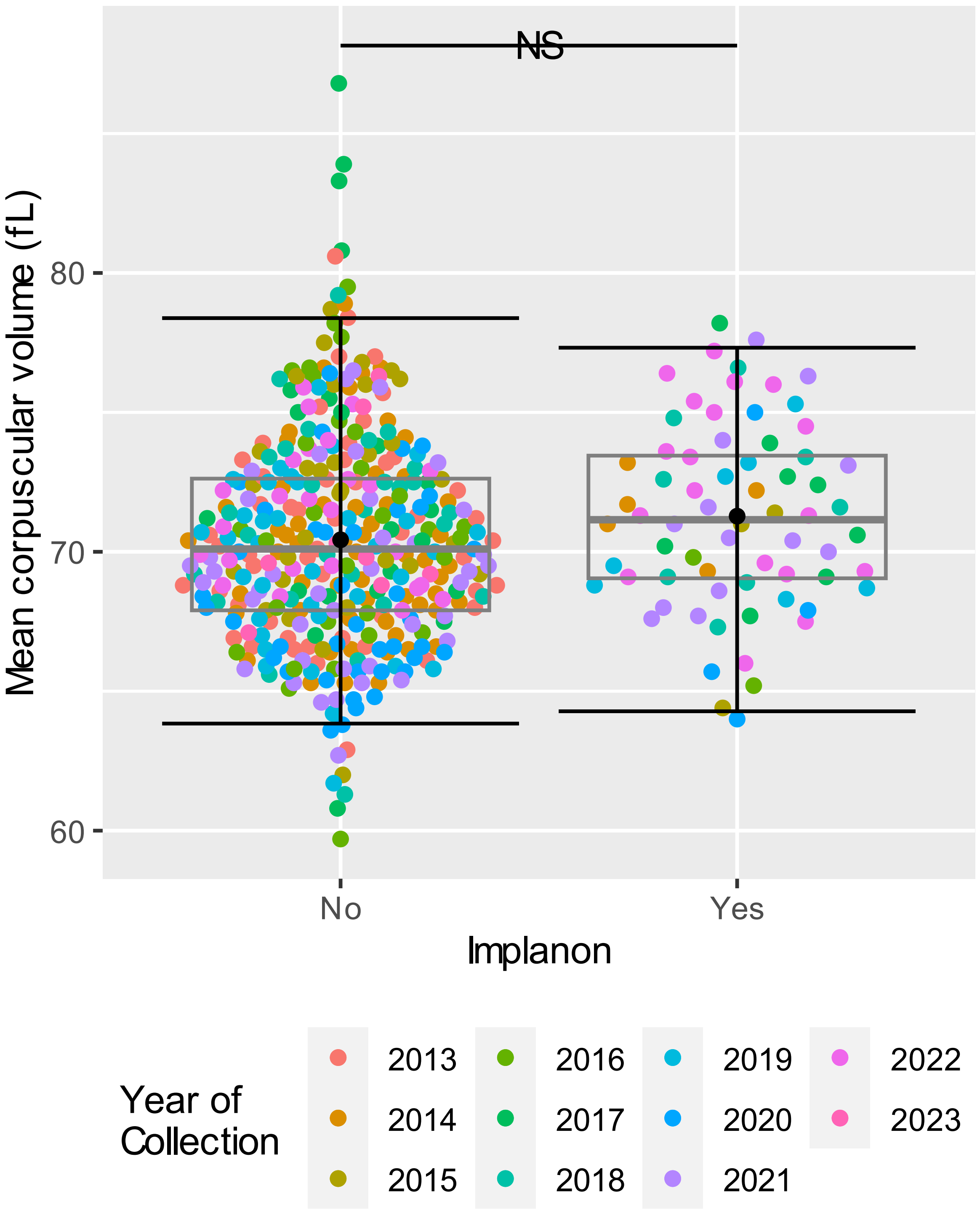

Figure S5 23. Mean Corpescular Volume according to implanon status in adult female common marmosets. Boxes indicate median and quartile ranges; black point indicates arithmetic mean and error bars indicate the normal range. Symbol colours indicate year of collection; asterisks indicate statistical significance levels NS = not significant, * < 0.001, ** < 0.0001, *** < 0.00001, **** < 0.000001

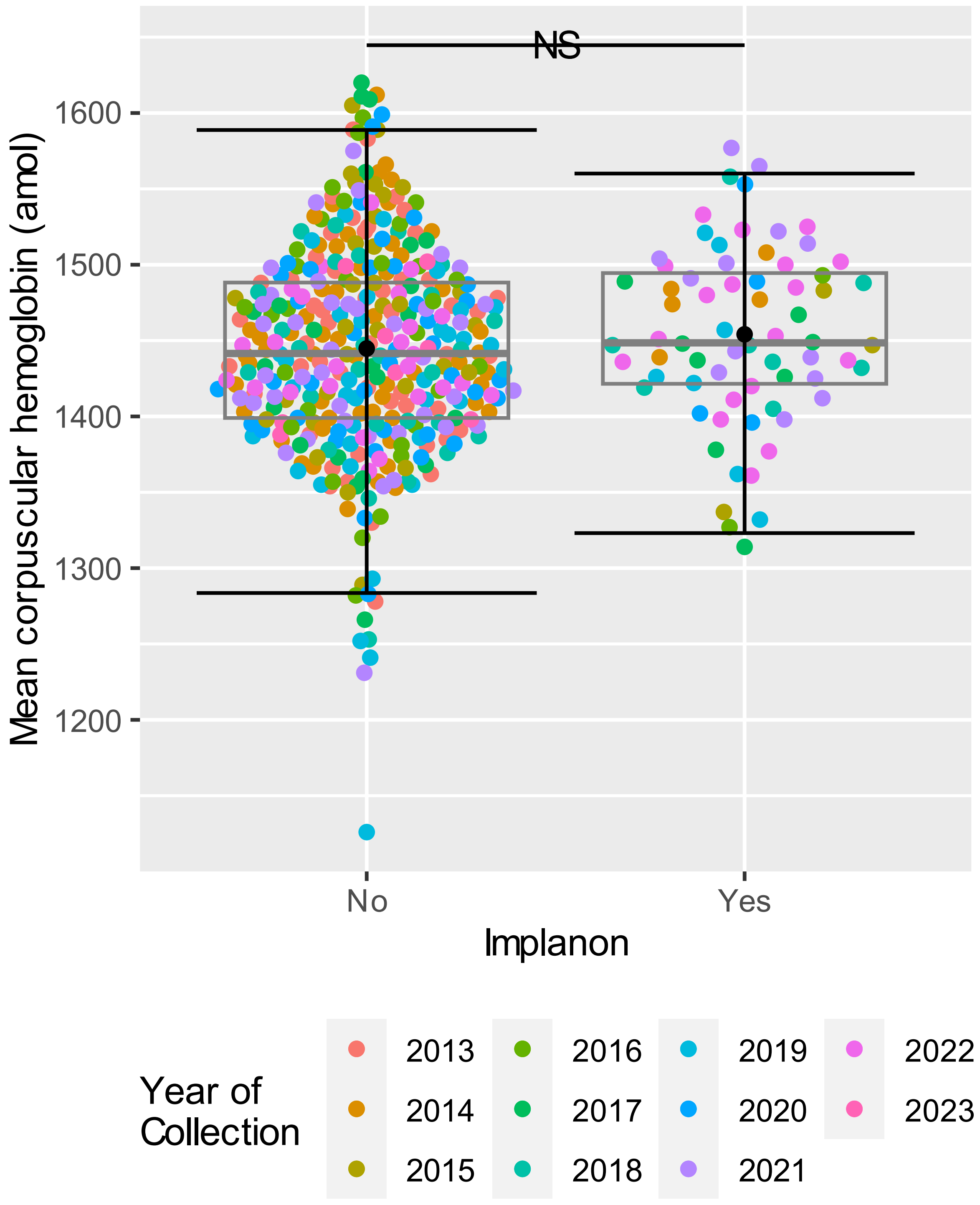

Figure S5 24. Mean Corpescular Haemoglobin according to implanon status in adult female common marmosets. Boxes indicate median and quartile ranges; black point indicates arithmetic mean and error bars indicate the normal range. Symbol colours indicate year of collection; asterisks indicate statistical significance levels NS = not significant, * < 0.001, ** < 0.0001, *** < 0.00001, **** < 0.000001

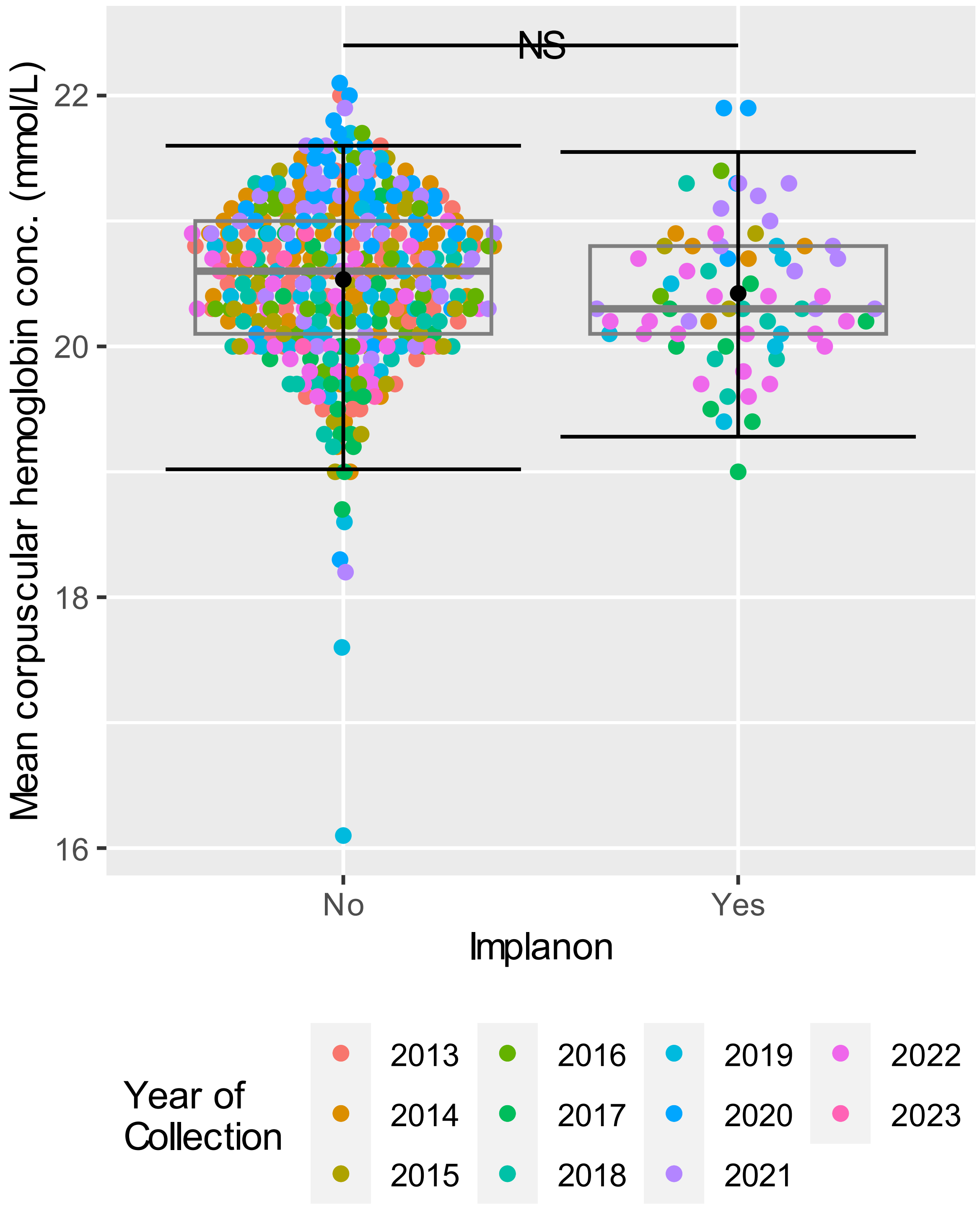

Figure S5 25. Mean Corpescular Haemoglobin Concentration according to implanon status in adult female common marmosets. Boxes indicate median and quartile ranges; black point indicates arithmetic mean and error bars indicate the normal range. Symbol colours indicate year of collection; asterisks indicate statistical significance levels NS = not significant, * < 0.001, ** < 0.0001, *** < 0.00001, **** < 0.000001

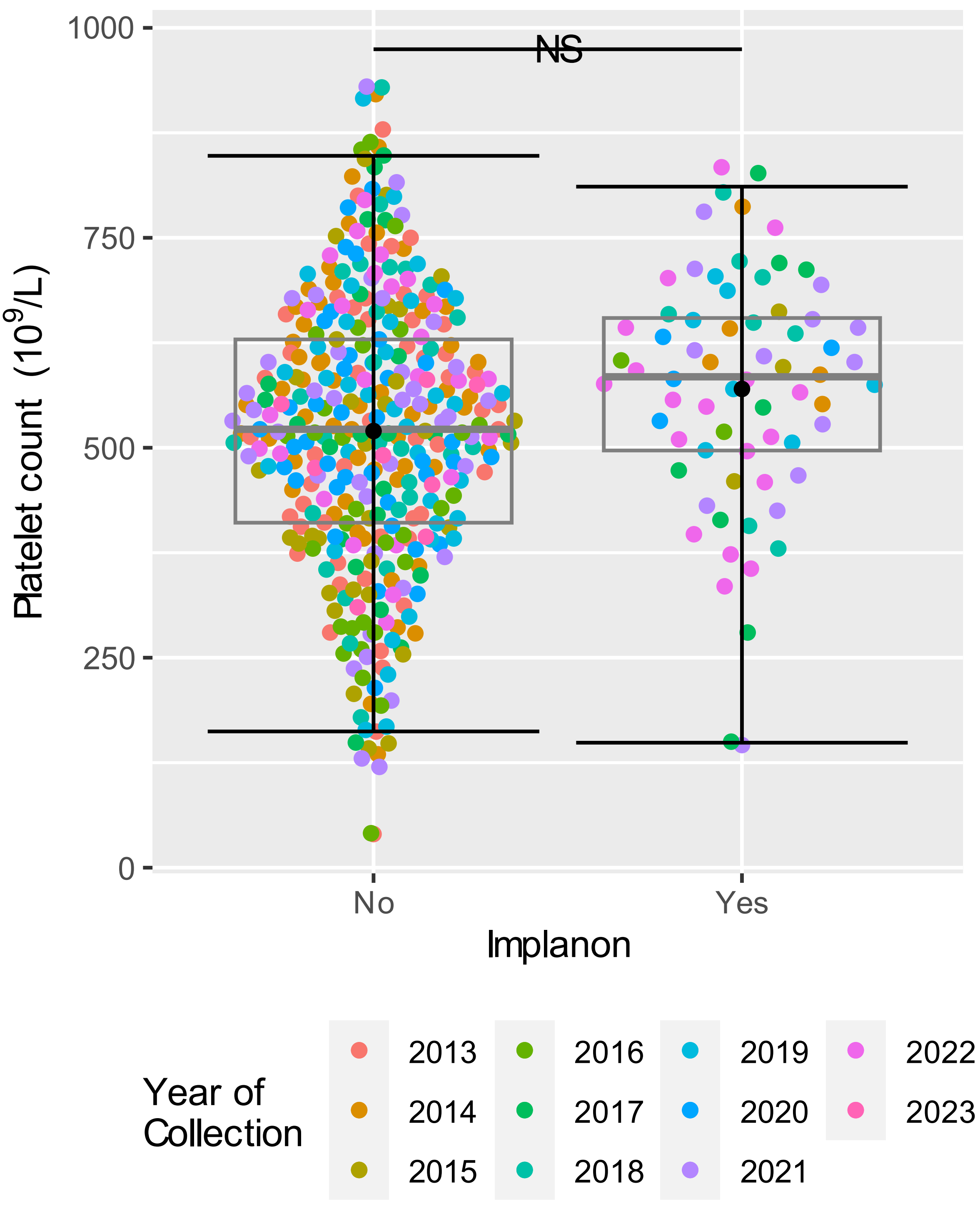

Figure S5 26. Platelet Count according to implanon status in adult female common marmosets. Boxes indicate median and quartile ranges; black point indicates arithmetic mean and error bars indicate the normal range. Symbol colours indicate year of collection; asterisks indicate statistical significance levels NS = not significant, * < 0.001, ** < 0.0001, *** < 0.00001, **** < 0.000001

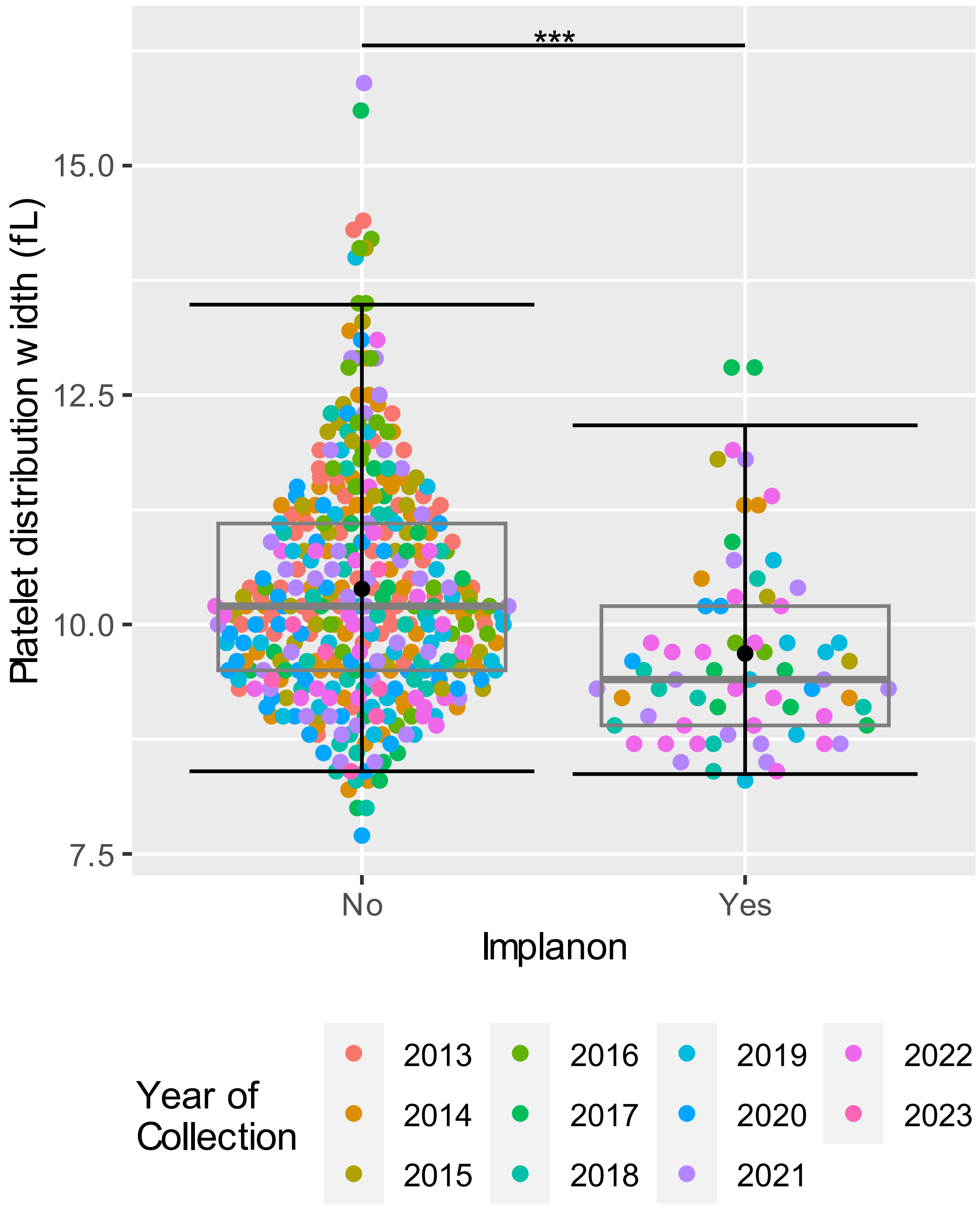

Figure S5 27. Platelet distribution width according to implanon status in adult female common marmosets. Boxes indicate median and quartile ranges; black point indicates arithmetic mean and error bars indicate the normal range. Symbol colours indicate year of collection; asterisks indicate statistical significance levels NS = not significant, * < 0.001, ** < 0.0001, *** < 0.00001, **** < 0.000001

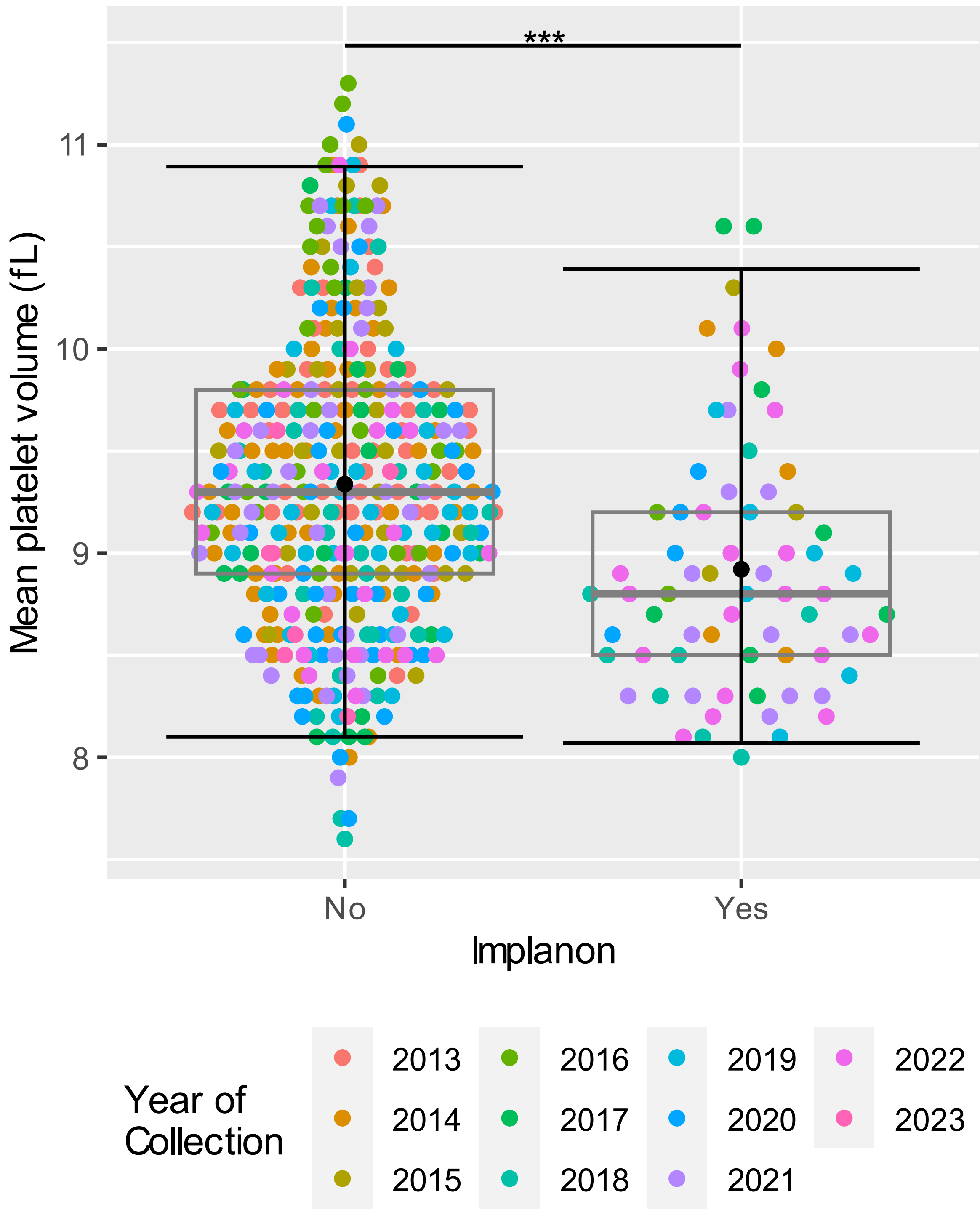

Figure S5 28. Mean Platelet Volume according to implanon status in adult female common marmosets. Boxes indicate median and quartile ranges; black point indicates arithmetic mean and error bars indicate the normal range. Symbol colours indicate year of collection; asterisks indicate statistical significance levels NS = not significant, * < 0.001, ** < 0.0001, *** < 0.00001, **** < 0.000001

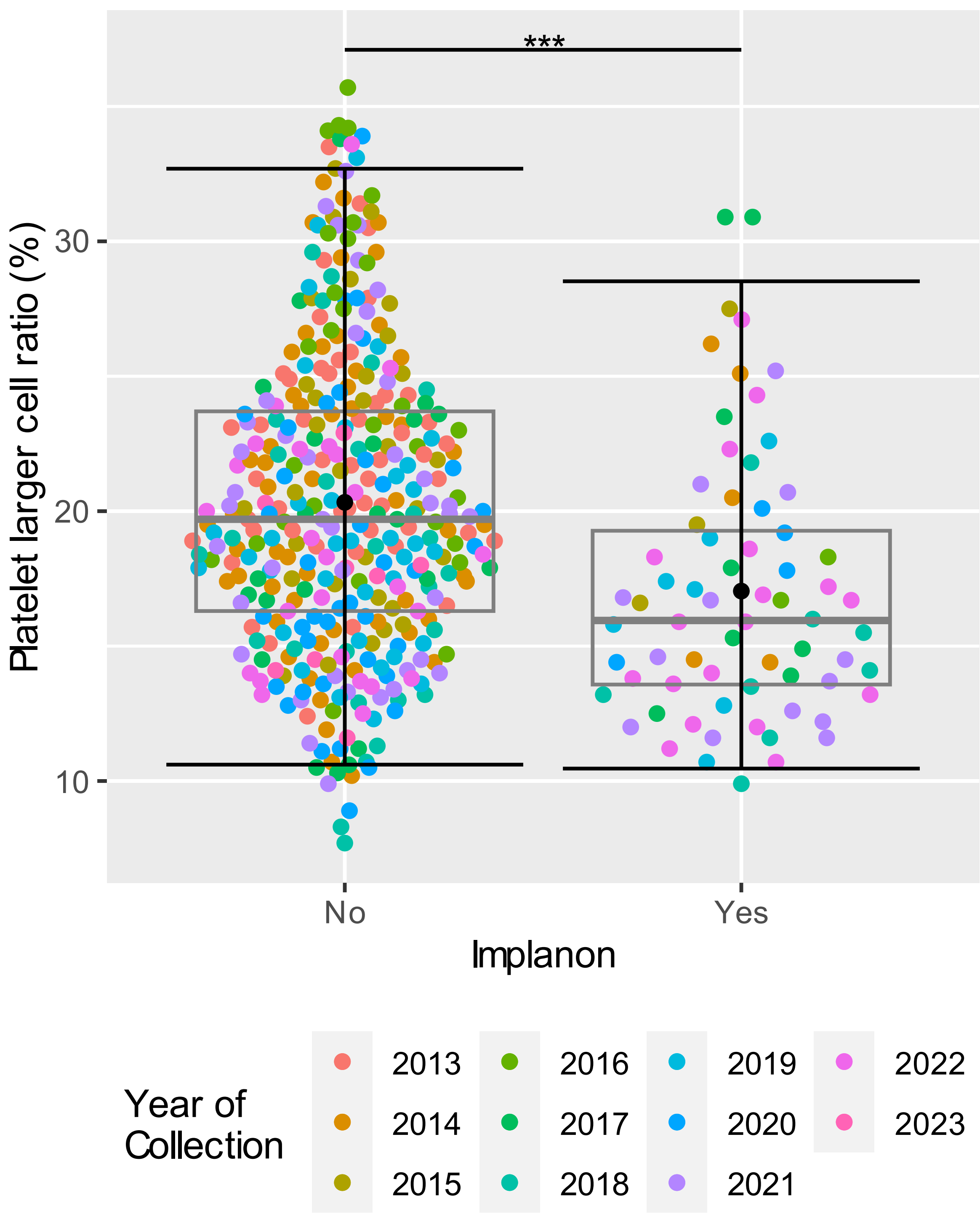

Figure S5 29. Platelet Larger Cell Ratio according to implanon status in adult female common marmosets. Boxes indicate median and quartile ranges; black point indicates arithmetic mean and error bars indicate the normal range. Symbol colours indicate year of collection; asterisks indicate statistical significance levels NS = not significant, * < 0.001, ** < 0.0001, *** < 0.00001, **** < 0.000001

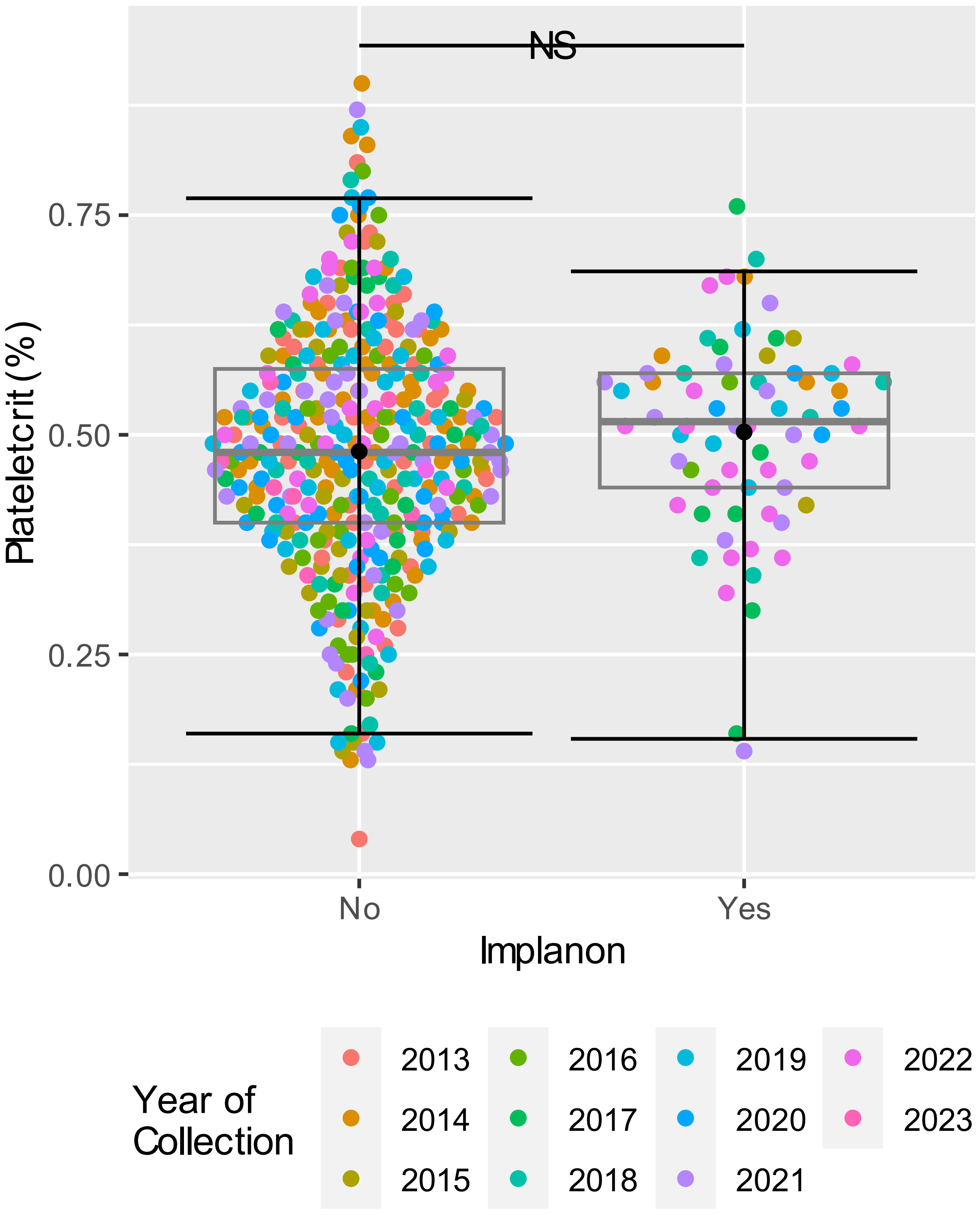

Figure S5 30. Plateletcrit according to implanon status in adult female common marmosets. Boxes indicate median and quartile ranges; black point indicates arithmetic mean and error bars indicate the normal range. Symbol colours indicate year of collection; asterisks indicate statistical significance levels NS = not significant, * < 0.001, ** < 0.0001, *** < 0.00001, **** < 0.000001

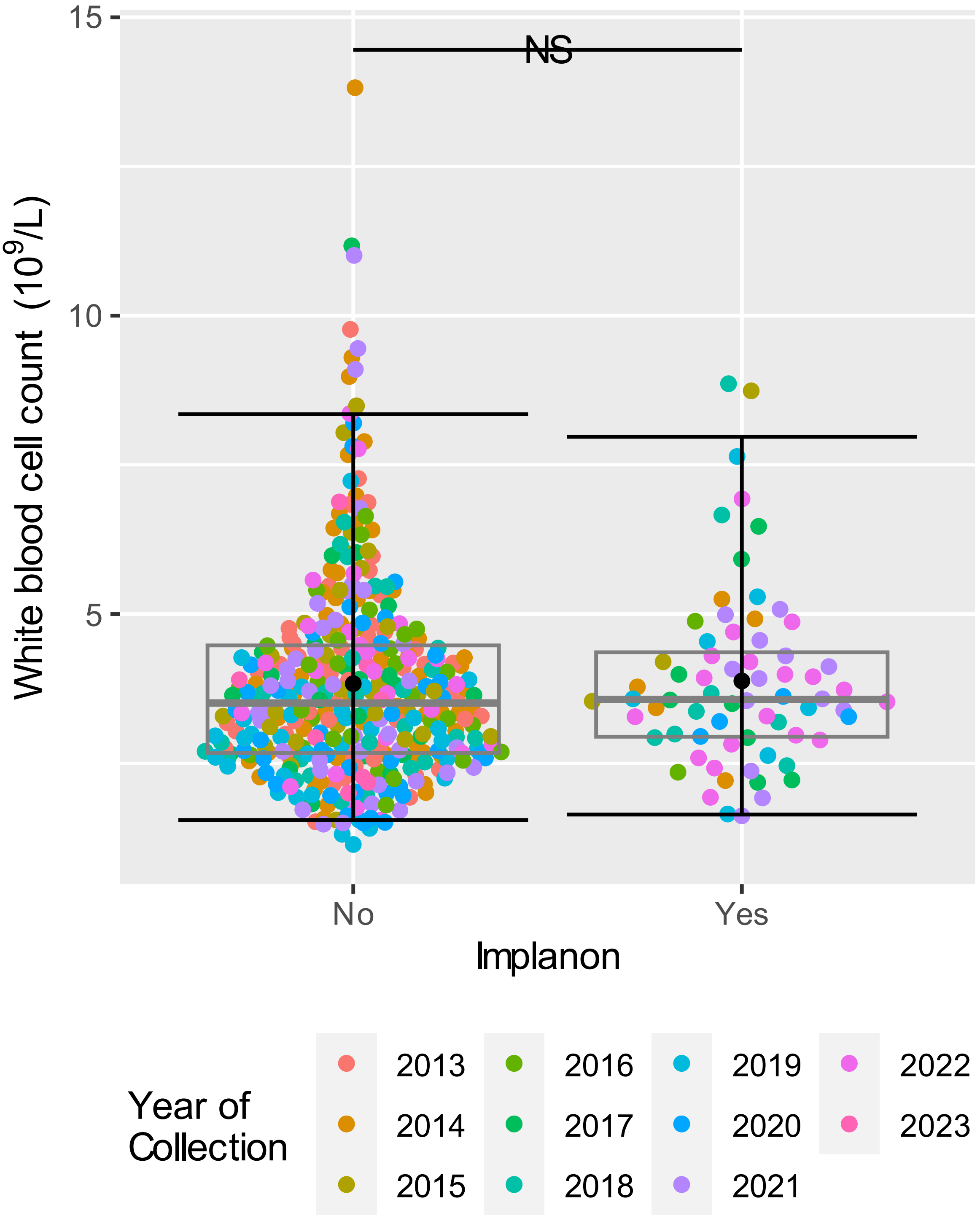

Figure S5 31. White Blood Cell count according to implanon status in adult female common marmosets. Boxes indicate median and quartile ranges; black point indicates arithmetic mean and error bars indicate the normal range. Symbol colours indicate year of collection; asterisks indicate statistical significance levels NS = not significant, * < 0.001, ** < 0.0001, *** < 0.00001, **** < 0.000001

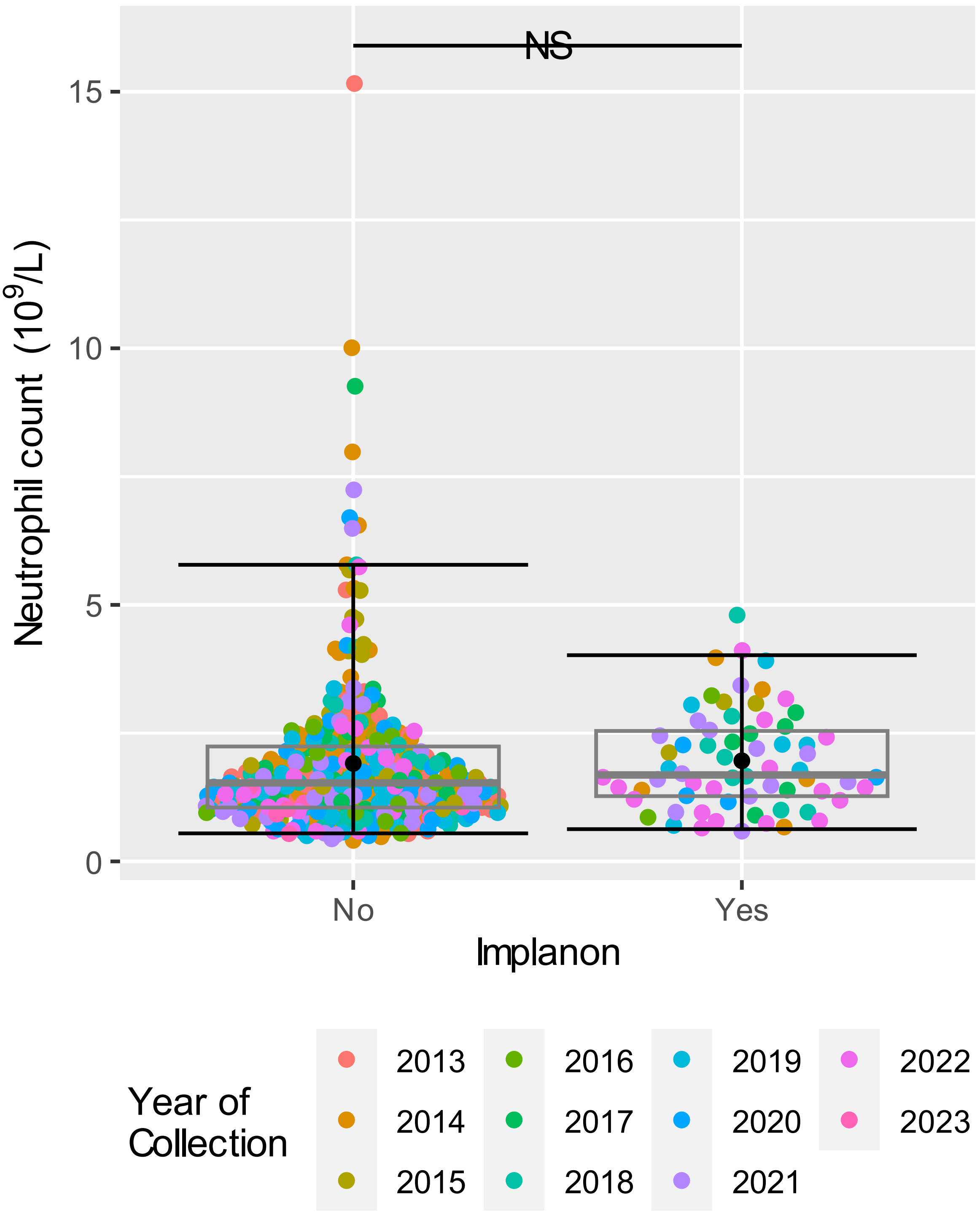

Figure S5 32. Neutrophil count according to implanon status in adult female common marmosets. Boxes indicate median and quartile ranges; black point indicates arithmetic mean and error bars indicate the normal range. Symbol colours indicate year of collection; asterisks indicate statistical significance levels NS = not significant, * < 0.001, ** < 0.0001, *** < 0.00001, **** < 0.000001

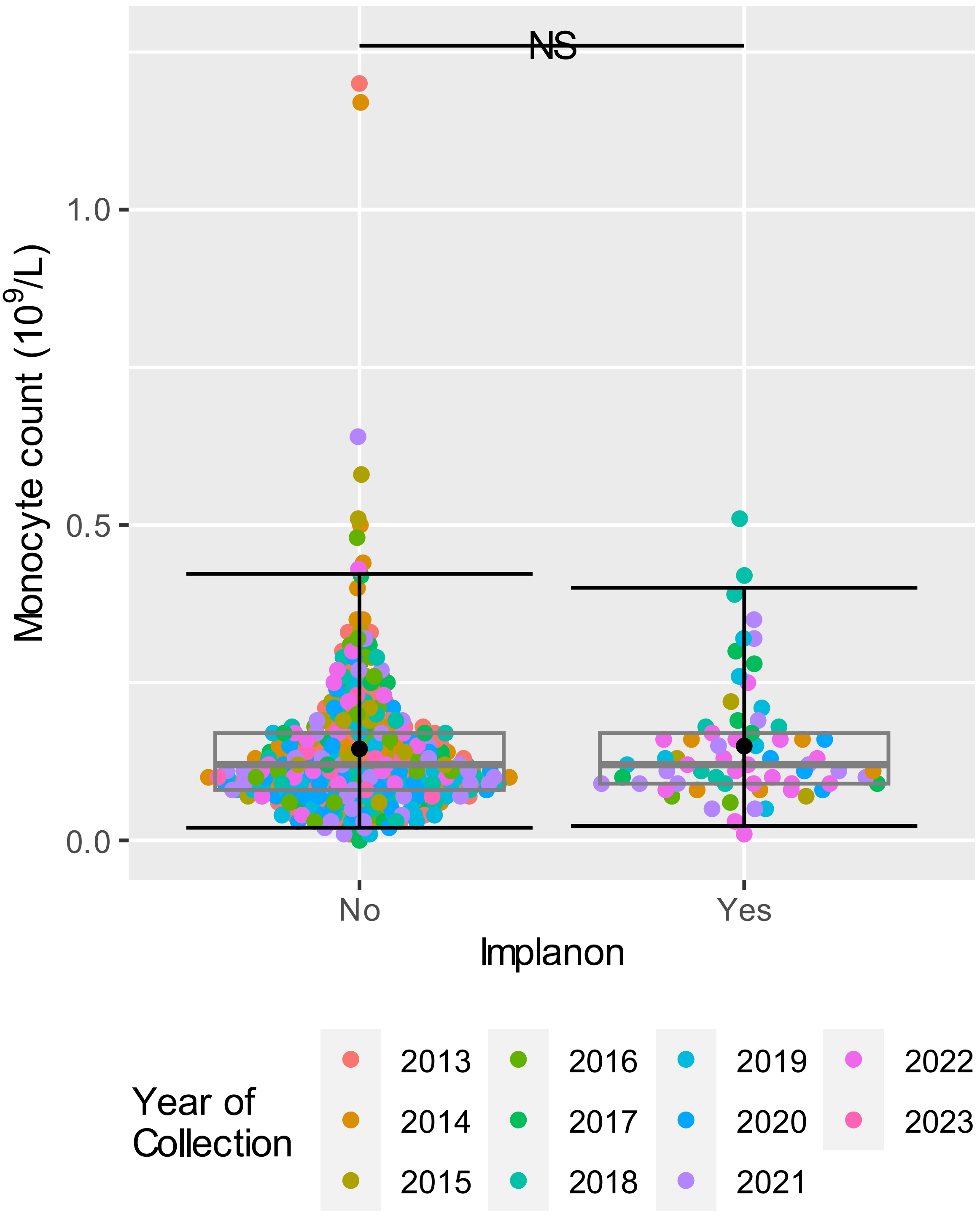

Figure S5 33. Monocyte count according to implanon status in adult female common marmosets. Boxes indicate median and quartile ranges; black point indicates arithmetic mean and error bars indicate the normal range. Symbol colours indicate year of collection; asterisks indicate statistical significance levels NS = not significant, * < 0.001, ** < 0.0001, *** < 0.00001, **** < 0.000001

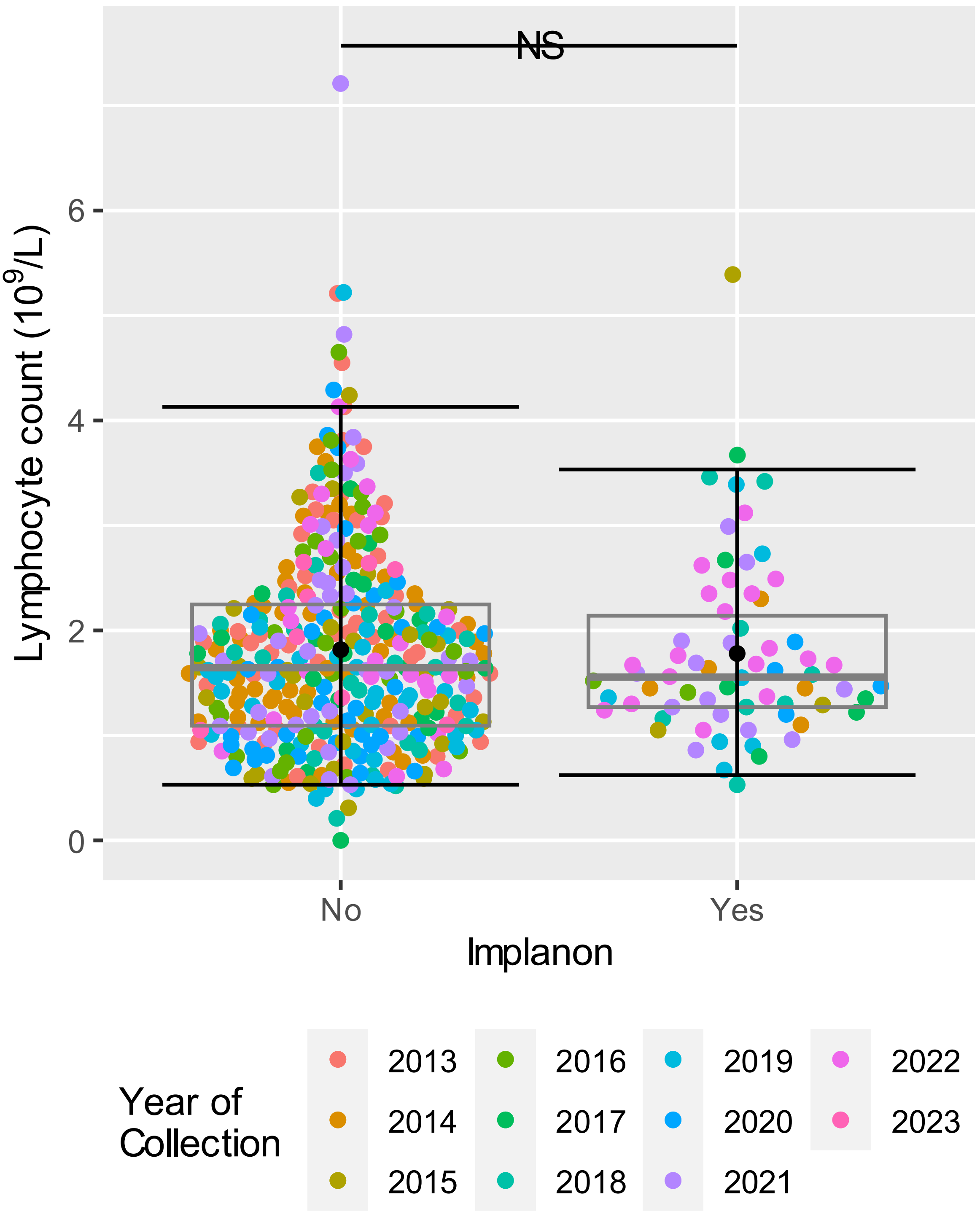

Figure S5 34. Lymphocyte count according to implanon status in adult female common marmosets. Boxes indicate median and quartile ranges; black point indicates arithmetic mean and error bars indicate the normal range. Symbol colours indicate year of collection; asterisks indicate statistical significance levels NS = not significant, * < 0.001, ** < 0.0001, *** < 0.00001, **** < 0.000001

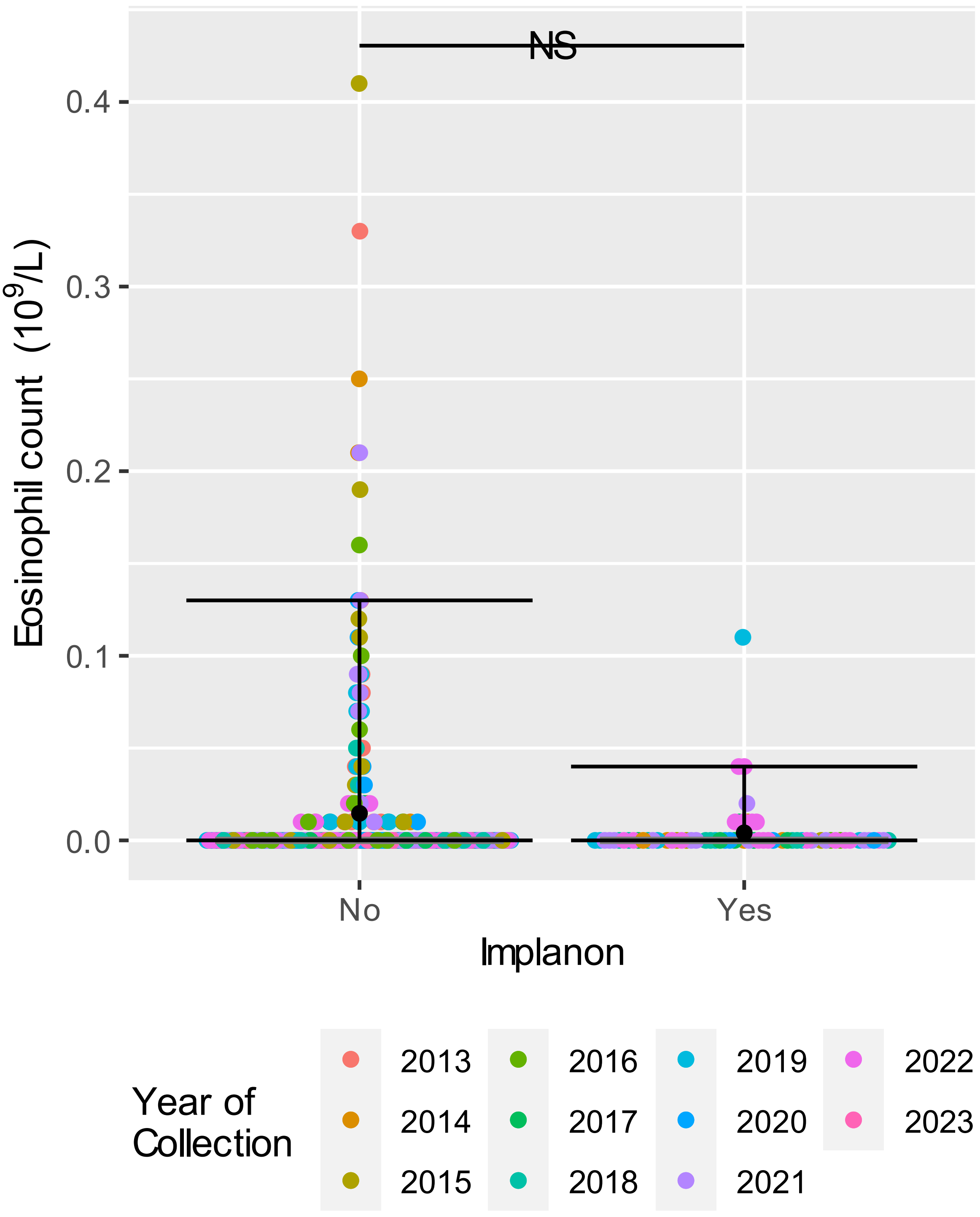

Figure S5 35. Eosinophil count according to implanon status in adult female common marmosets. Boxes indicate median and quartile ranges; black point indicates arithmetic mean and error bars indicate the normal range. Symbol colours indicate year of collection; asterisks indicate statistical significance levels NS = not significant, * < 0.001, ** < 0.0001, *** < 0.00001, **** < 0.000001

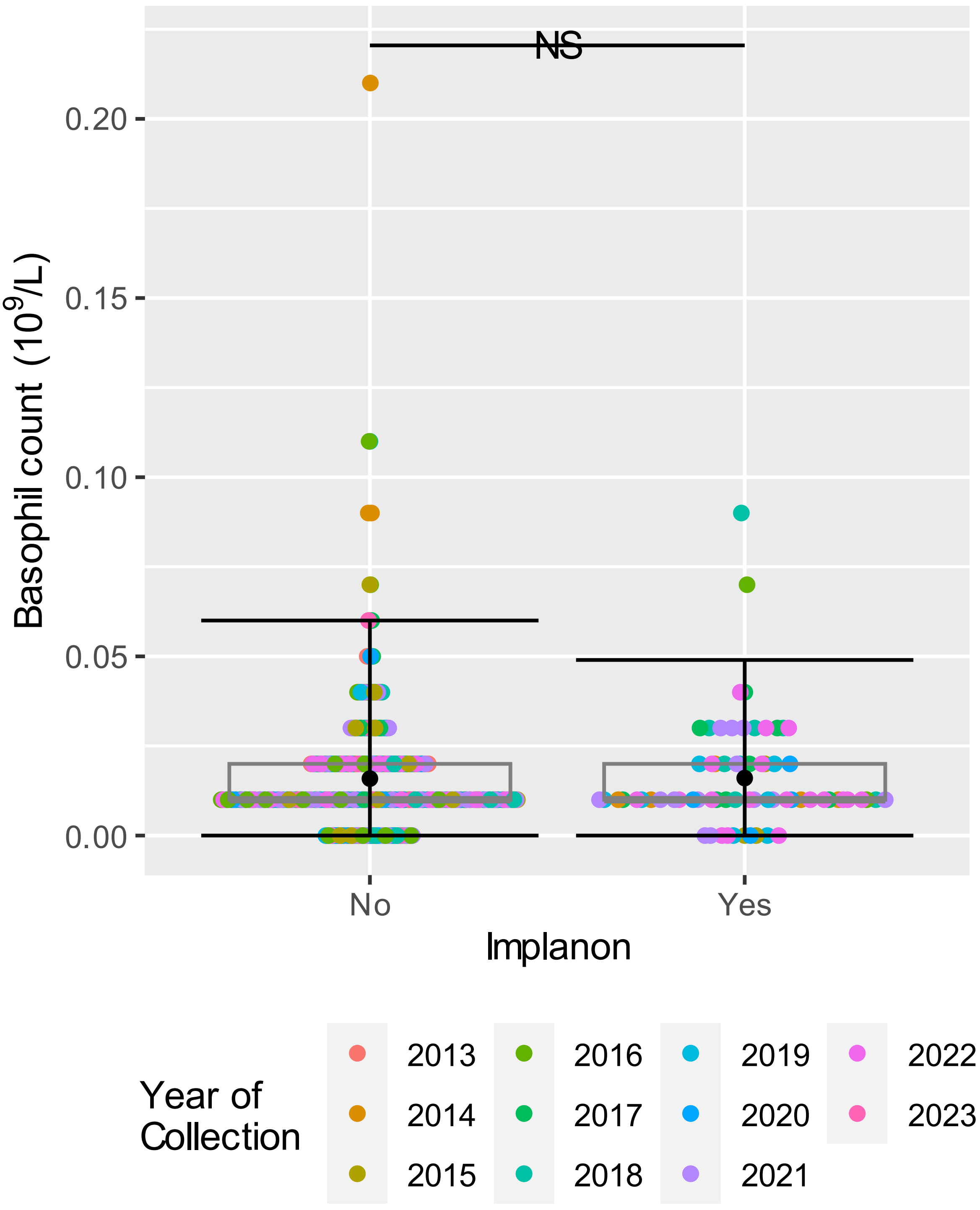

Figure S5 36. Basophil count according to implanon status in adult female common marmosets. Boxes indicate median and quartile ranges; black point indicates arithmetic mean and error bars indicate the normal range. Symbol colours indicate year of collection; asterisks indicate statistical significance levels NS = not significant, * < 0.001, ** < 0.0001, *** < 0.00001, **** < 0.000001
